# Supplementary material for: Bis-indole chiral architectures for asymmetric catalysis
Source: Nat Commun. 2025 Apr 17;16:3676. doi: 10.1038/s41467-025-58313-4 (PMC12006493; doi:10.1038/s41467-025-58313-4)
Supplement: Supplementary file 1 — Supplementary Information [file 41467_2025_58313_MOESM1_ESM.pdf]

Supplementary information

SPIROCYCLIC BIS-INDOLE CHIRAL ACHITECTURES FOR ASYMMETRIC CATALYSIS

Junshan Lai[1], Benjamin List[2]*, Jolene P. Reid[1]*

Affiliations:

[1]Department of Chemistry, University of British Columbia, Vancouver, British Columbia V6T 1Z1, Canada

[2]Max-Planck-Institut für Kohlenforschung, Kaiser-Wilhelm-Platz 1, 45470, Mülheim an der Ruhr, Germany

*Corresponding author. Email: list@kofo.mpg.de; jreid@chem.ubc.ca

Contents

1 General Information 1

2 Full List Of Authors In The Gaussian Reference 3

3 Computational Details 4

4 Reaction Optimization 9

5 Gram Scale Synthesis Of *R*-4a And Utility 14

6 Mechanism Studies 30

7 Application Of Spindole-Derived Catalysts And Ligands 38

8 Unreactive Indoles 38

9 Characterization Data 44

10 Crystal Data 58

11 NMR Spectra 63

12 SFC Chromatograms 130

13 References 178

1 General information

Method:

Unless otherwise stated, all reactions were magnetically stirred and were performed in standard, dry glassware, under a nitrogen atmosphere, applying standard Schlenk techniques. The reactions for preparation of **4a-4bb** were performed under air in vessels and sealed with Teflon caps, heating carried out with a metal bath. Cooling baths were prepared in Dewar vessels, filled with ice/water (0 °C) or dry ice/acetone (–78 °C). Reported concentrations refer to solution volumes at room temperature. Solvents and liquid reagents, as well as solutions of solid or liquid reagents were added via syringes, stainless steel or polyethylene cannulas through rubber septa or through a weak argon counter-flow. Solid reagents were added through a weak argon counter-flow. Concentration of organic solutions under reduced pressure was performed at 38 °C using a rotary evaporator unless otherwise indicated; the remaining compound was dried in high vacuum at ambient temperature. All given yields are isolated yields of chromatographically and nuclear magnetic resonance (NMR) spectroscopically pure materials. Flash chromatography was carried out using 230-400 mesh silica gel and dry-packed columns. Thin layer chromatography was carried out using Merck TLC Silica gel 60 F254 aluminum sheets. Components were visualized by UV light (λ = 254 nm) and stained with phosphomolybdic dip.

Chemicals:

Unless otherwise stated, chemicals were purchased from commercial suppliers (Sigma-Aldrich, Aaron Chemicals, AK Scientific) and used without further purification. Chiral Brønsted acids **A1-A13**,[1] **A14-A18**,[2] **B1-B18**,[3] **C1-C14**,[4] **D1-D4**,[5] **E1-E4**,[6] chemicals **9a** and **9b**,[7] **18**,[8] **20** [9] and **22**[10] were prepared following known literature procedures.

Solvent:

Solvents (dichloromethane, diethyl ether, tetrahydrofuran, toluene, benzene) are HPLC grade dried and degassed with Solvent Purification System (SPS). Other anhydrous solvents (1,4-dioxane, DMF, ethyl acetate, acetonitrile, methanol and hexane) were purchased from commercial suppliers

and dried over molecular sieves. Deuterated solvents were purchased from Cambridge Isotope Laboratories and were used as received, unless otherwise described.

Instrumentation:

All nuclear magnetic resonance (^{1}H, ^{13}C, ^{19}F, ^{31}P NMR) spectra were recorded at 298 K on a Bruker Avance 400dir Ultrashield apparatus in a suitable deuterated solvent. The solvent employed and respective measuring frequency are indicated for each experiment and were used as internal standard. Proton nuclear magnetic resonance (^{1}H NMR) spectra are referenced to the residual protium resonances of the NMR solvent ($CDCl_3$: 7.26 ppm; CD_2Cl_2: 5.32 ppm; $(CD_3)_2CO$: 2.09 ppm) and are referenced to the carbon resonances of the NMR solvent ($CDCl_3$: 77.16; CD_2Cl_2: 53.84 ppm; $(CD_3)_2CO$: 29.85 ppm, 206.25 ppm). Data are represented as follows: chemical shift, multiplicity (br = broad, s = singlet, d = doublet, t = triplet, q = quartet, p = pentet, sept = septet, m = multiplet), coupling constants in Hertz (Hz), integration. Mass spectral (MS) data were obtained by submission to the Mass Spectrometry Facility at UBC Chemistry Department. Optical rotation was measured using a 1 mL cell with a 0.5 dm path length on a Jasco P-2000 polarimeter at 589 nm (sodium D line) at room temperature. Chiral Supercritical Fluid Chromatography (SFC) was performed on Waters ACQUITY UPC2 instrument. Analysis was performed at 35°C, with the indicated APBR pressure, flow rate, column, solvent system and detector wavelength. In-situ IR experiments were carried out using a Perkin Elmer FTIR spectrometer.

Abbreviations:

ee = enantiomeric excess, de = diastereomeric excess, rt = room temperature, Me = methyl, Et = ethyl, Pr = propyl, Bu = butyl, MeOH = methanol, EtOAc = ethyl acetate, equiv. = equivalent, TLC = thin layer chromatography, min = minutes, h = hours, d = days, THF = tetrahydrofuran, DCM = dichloromethane, DMF = Dimethylformamide, TBS = tert-butyldimethylsilyl, TMS = trimethylsilyl, Tf = SO_2CF_3, MOM = methoxymethyl.

2 Full list of authors in the Gaussian reference

M. J. Frisch, G. W. Trucks, H. B. Schlegel, G. E. Scuseria, M. A. Robb, J. R. Cheeseman, G. Scalmani, V. Barone, G. A. Petersson, H. Nakatsuji, X. Li, M. Caricato, A. V. Marenich, J. Bloino, B. G. Janesko, R. Gomperts, B. Mennucci, H. P. Hratchian, J. V. Ortiz, A. F. Izmaylov, J. L. Sonnenberg, D. Williams-Young, F. Ding, F. Lipparini, F. Egidi, J. Goings, B. Peng, A. Petrone, T. Henderson, D. Ranasinghe, V. G. Zakrzewski, J. Gao, N. Rega, G. Zheng, W. Liang, M. Hada, M. Ehara, K. Toyota, R. Fukuda, J. Hasegawa, M. Ishida, T. Nakajima, Y. Honda, O. Kitao, H. Nakai, T. Vreven, K. Throssell, J. A. Montgomery, Jr., J. E. Peralta, F. Ogliaro, M. J. Bearpark, J. J. Heyd, E. N. Brothers, K. N. Kudin, V. N. Staroverov, T. A. Keith, R. Kobayashi, J. Normand, K. Raghavachari, A. P. Rendell, J. C. Burant, S. S. Iyengar, J. Tomasi, M. Cossi, J. M. Millam, M. Klene, C. Adamo, R. Cammi, J. W. Ochterski, R. L. Martin, K. Morokuma, O. Farkas, J. B. Foresman, and D. J. Fox, Gaussian, Inc., Wallingford CT, 2016.

3 Computational details

All calculations were performed using the Gaussian 16 (revision C.01).[11]

For calculating the thermodynamics of the uncatalyzed process, bis-indoles, indole, acetone, and water were computed using the ωB97XD density functional,[12] and split-valence polarized 6-31G(d,p) basis set.[13]

Calculations for the catalyst-product complexes involving substrate **4a** and catalyst **D4** were performed using the ωB97XD density functional and the split-valence polarized 6-31G(d) basis set. The free energy corrections were calculated using Grimme's quasiharmonic treatment[14] with GoodVibes [15]. Energies in solution were obtained from structures optimized in the gas phase through single-point calculations using ωB97XD/6-311G(d,p) with the polarizable continuum model (IEFPCM) in THF. These values were used to correct the Gibbs free energy derived from the gas-phase calculations. Conformational searches were performed with Macromodel version 11.7 [16] and the OPLS3 force field.[17] Structures were generated using the mixed torsional/Low-mode sampling method.

For quantum chemical calculations of bisphosphine-$PdCl_2$ complexes, all geometries of the stationary points were obtained via unconstrained optimizations using the ωB97XD density functional. [12] The SDD basis set was used for the Pd atoms, while 6-31G(d,p) was used on all other atoms. NBO version 3.1 was applied to obtain the natural atomic charge information using the same level of theory.

All relevant cartesian coordinates and energies for these structures are provided.

Structures relevant to the uncatalyzed process

Acetone
ωB97XD/6-31G(d,p) Free Energy = -193.044483
Number of Imaginary Frequencies = 0

H_2O
ωB97XD/6-31G(d,p) Free Energy = -76.393685
Number of Imaginary Frequencies = 0

Indole
ωB97XD/6-31G(d,p) Free Energy = -363.603616
Number of Imaginary Frequencies = 0

Bis-indole 2a
ωB97XD/6-31G(d,p) Free Energy = -843.858352
Number of Imaginary Frequencies = 0

Bis-indole 3a
ωB97XD/6-31G(d,p) Free Energy = -960.507841
Number of Imaginary Frequencies = 0

Bis-indole 4a
ωB97XD/6-31G(d,p) Free Energy = -1077.155185
Number of Imaginary Frequencies = 0

The lowest energy structure that led to major enantiomeric product (R) (GS-R)

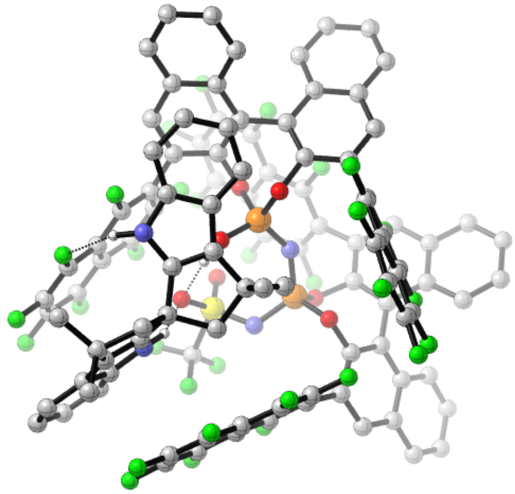

ωB97XD/6-31G(d) Energy = -8986.740561

ωB97XD/6-31G(d) Quasiharmonic Free Energy = -8985.534739
IEFPCM(THF)-ωB97XD/6-311G(d,p) Energy = -8988.791556
IEFPCM(THF)-ωB97XD/6-31G(d) Derived free energy in solution = -8987.585735
Number of Imaginary Frequencies = 0

The lowest energy structure that led to minor enantiomeric product (S) (GS-S)

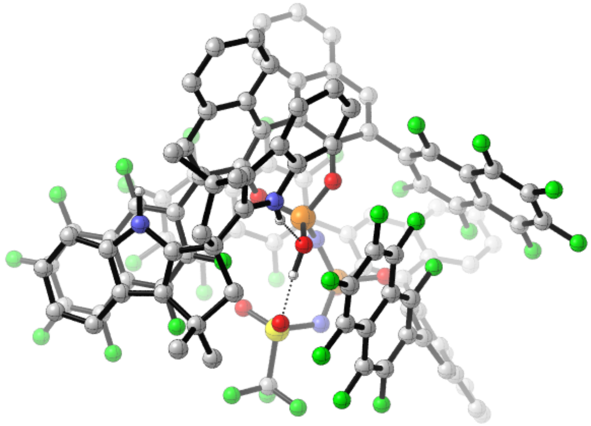

ωB97XD/6-31G(d) Energy = -8986.731999
ωB97XD/6-31G(d) Quasiharmonic Free Energy = -8985.529027
IEFPCM(THF)-ωB97XD/6-311G(d,p) Energy = -8988.784103
IEFPCM(THF)-ωB97XD/6-31G(d) Derived free energy in solution = -8987.581131
Number of Imaginary Frequencies = 0

BINAP-$PdCl_2$

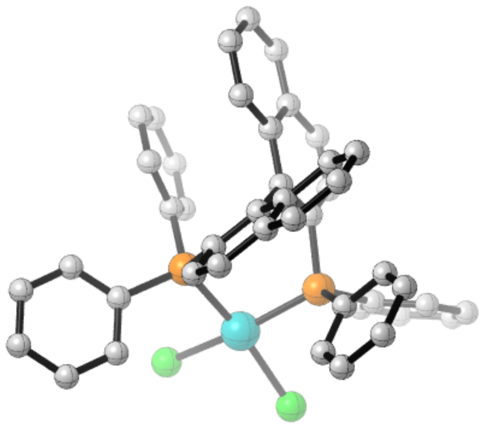

ωB97XD/6-31G(d,p)-SDD(Pd) Energy = -3426.550954
ωB97XD/6-31G(d,p)-SDD(Pd) Free Energy = -3425.990482

Number of Imaginary Frequencies = 0

SDP-PdCl$_2$

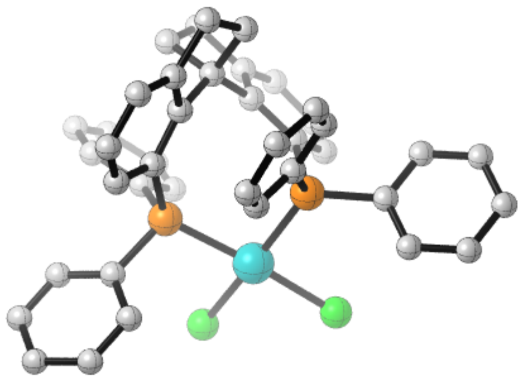

ωB97XD/6-31G(d,p)-SDD(Pd) Energy = -3313.472581
ωB97XD/6-31G(d,p)-SDD(Pd) Free Energy = -3312.902464
Number of Imaginary Frequencies = 0

SIDP-PdCl$_2$

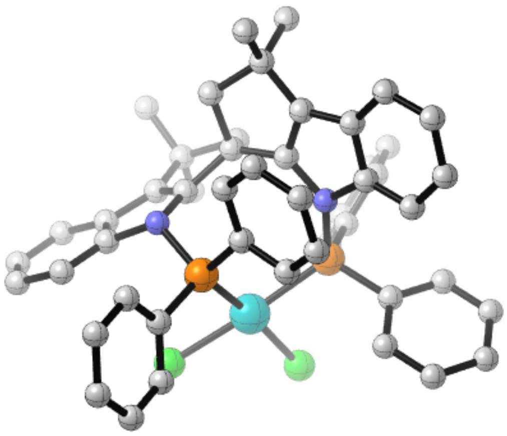

ωB97XD /6-31G(d,p)-SDD(Pd) Energy = -3733.751325
ωB97XD /6-31G(d,p))-SDD(Pd) Free Energy = -3733.018886
Number of Imaginary Frequencies = 0

Distortion-interaction analysis

Both ground state structures exhibit similar interactions, including an intramolecular hydrogen bond between the OH and the SO_2CF_3 group, a hydrogen bond between the SPINDOLE and the SO_2CF_3 group, and stacking interactions between the product and the aromatic group of the catalyst. Notably, the lowest-energy complex reveals an additional hydrogen bond between a fluorine atom on one of the aromatic groups and the N-H group on the product. The reasons for the energy differences between **GS-R** and **GS-S** were further investigated using distortion-interaction analysis. These confirmed that stronger attractive interactions in GS-R compared to GS-S contribute to the stabilization of this structure.

Basically, the equation $\Delta\Delta E^{\ddagger} = \Delta\Delta E_{int} - \Delta\Delta E_{distort\mathbf{Cat}} - \Delta\Delta E_{distort\mathbf{Pdt}}$ allows the relative energy difference between the two GS complexes to be understood partially on the basis of the energy required to distort the structures of the catalyst and product to the GS geometry. The energy required for this process is usually positive but the interactions between the catalyst and product, appearing as $\Delta\Delta E_{int}$ in the equation, offsets this energetically costly distortion process, shown as the $\Delta\Delta E_{distort}$ term. $\Delta\Delta E^{\ddagger}$ corresponds to the difference in IEFPCM(THF)-ωB97XD/6-311G(d,p) energy between **GS-R** and **GS-S**. To determine the differences in catalyst and product distortion energies the catalyst and product portions of the two GS are separated and a single point energy taken in the solution-phase at the ωB97XD/6-311G(d,p) level. The differences in energy of the distorted catalysts and substrates in **GS-R** and **GS-S** correspond to $\Delta\Delta E_{distort\mathbf{Cat}}$ and $\Delta\Delta E_{distort\mathbf{Pdt}}$**.** This information can then be used to calculate $\Delta\Delta E_{int}$.

As part of this we determined that

Distorted product **GS-R**
IEFPCM(THF)-ωB97XD/6-311G(d,p) energy = -1077.769063

Distorted catalyst **GS-R**
IEFPCM(THF)-ωB97XD/6-311G(d,p) energy = -7910.942235

Distorted product **GS-S**
IEFPCM(THF)-ωB97XD/6-311G(d,p) energy = -1077.768699

Distorted catalyst **GS-S**
IEFPCM(THF)-ωB97XD/6-311G(d,p) energy = -7910.945802

4 Reaction Optimization

4.1 General procedure for the optimization of the enantioselective synthesis of 4a with cat A-E

A 4 mL vessel was charged with 0.5 mL of toluene, **1a** (11.7 mg, 0.1 mmol, 1.0 equiv.), acetone (29 mg, 0.5 mmol, 5.0 equiv.), and the stated amount of catalyst. The vessel was sealed and stirred at the specified temperature for 3 days. The reaction mixture was then directly subjected to silica gel column chromatography (petroleum ether:AcOEt = 20:1) to afford compound **4a**.

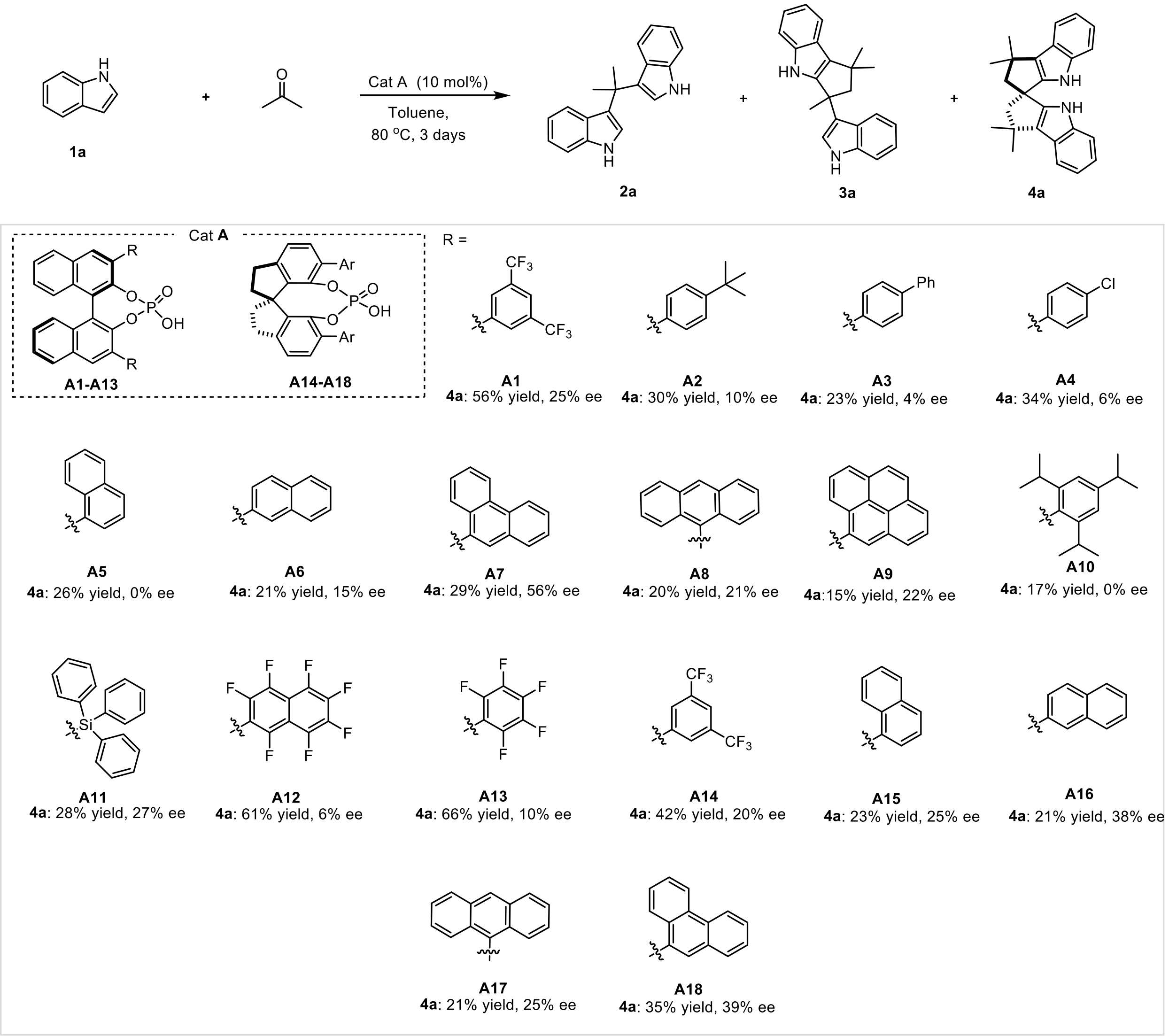

Figure S1: The optimization of the enantioselective synthesis of **4a** with cat. **A**.

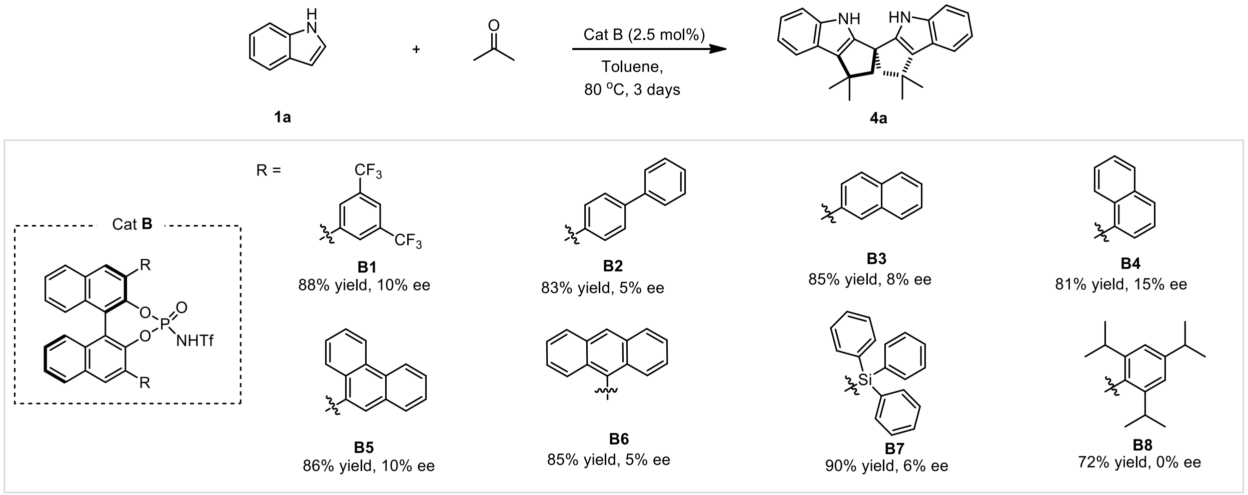

Figure S2: The optimization of the enantioselective synthesis of **4a** with cat. **B**.

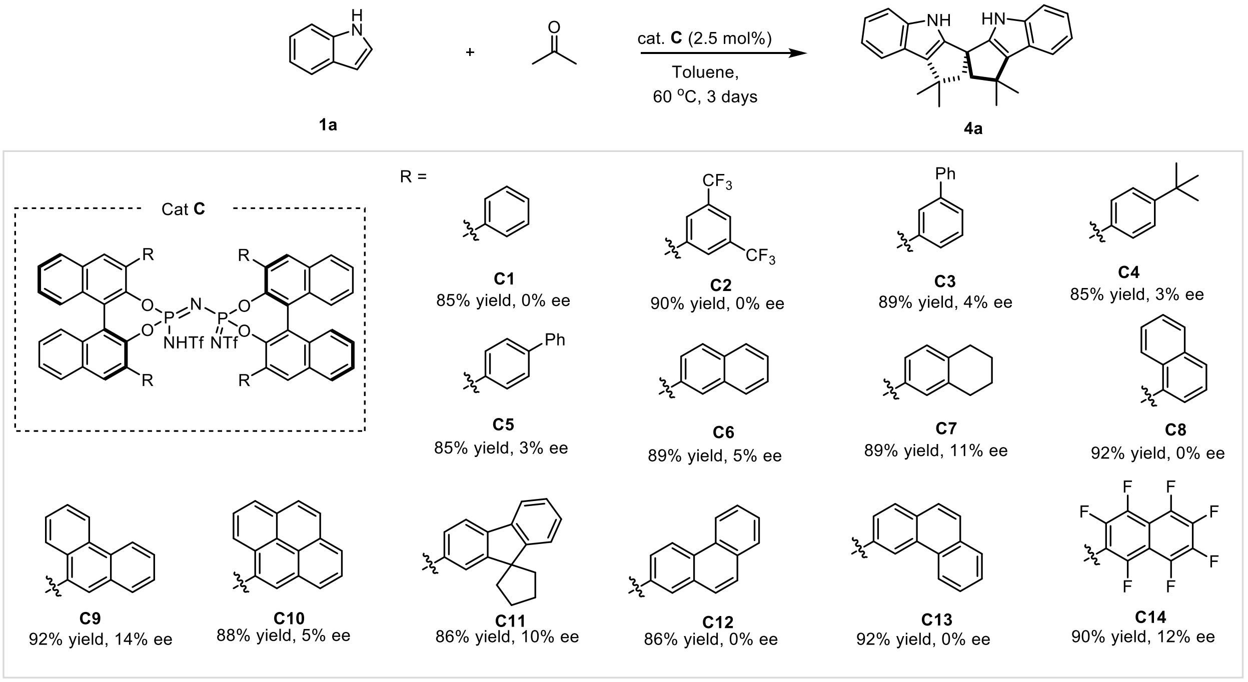

Figure S3: The optimization of the enantioselective synthesis of **4a** with cat. **C**.

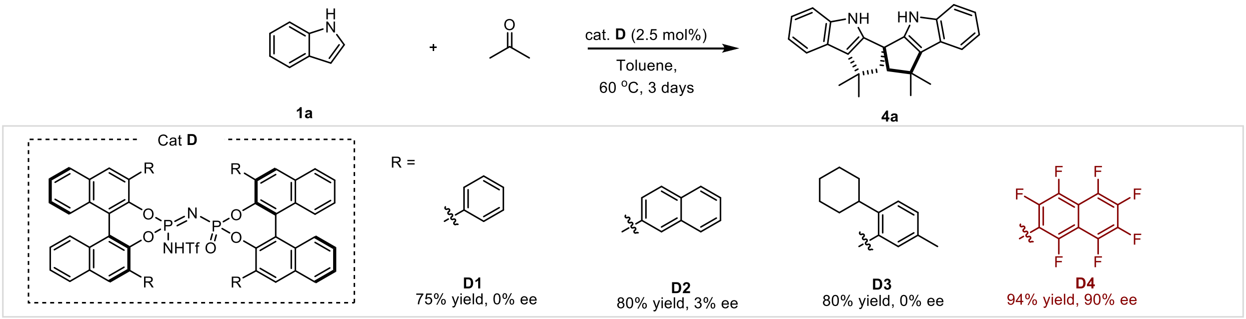

Figure S4: The optimization of the enantioselective synthesis of **4a** with cat. **D**.

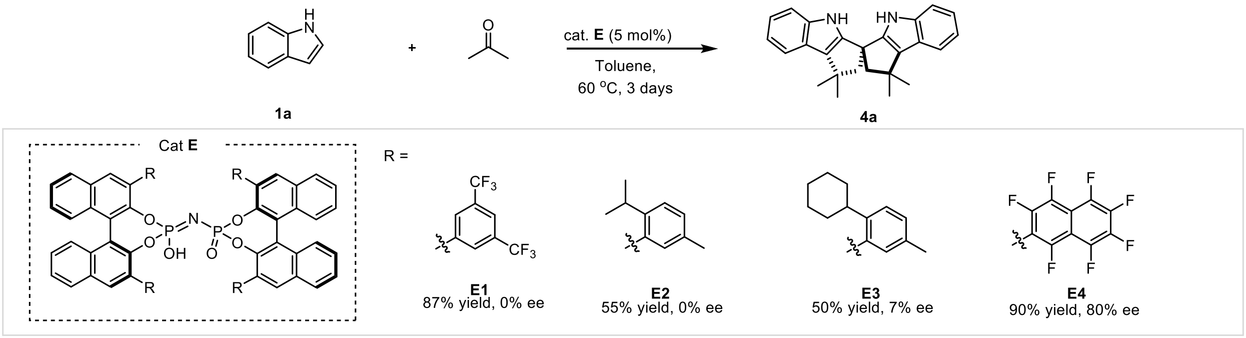

Figure S5: The optimization of the enantioselective synthesis of **4a** with cat. **E**.

4.2 General procedure for the optimization of solvent for the enantioselective synthesis of 4a with cat D4

A 4 mL vessel was charged with 0.5 mL of solvent, 1 (11.7 mg, 0.1 mmol, 1.0 equiv.), acetone (29 mg, 0.5 mmol, 5.0 equiv.), and catalyst (*R,R*) -**D4** (4.5 mg, 0.0025 mmol, 2.5 mol%). The vessel was sealed and stirred at 60 °C for 3–5 days. The reaction mixture was then directly subjected to silica gel column chromatography (petroleum ether:AcOEt = 20:1) to afford compound **4a**. Note: we swapped enantiomers at some point in the optimization process because of availability.

Table S1: the optimization of solvent for the enantioselective synthesis of 4a with cat D4

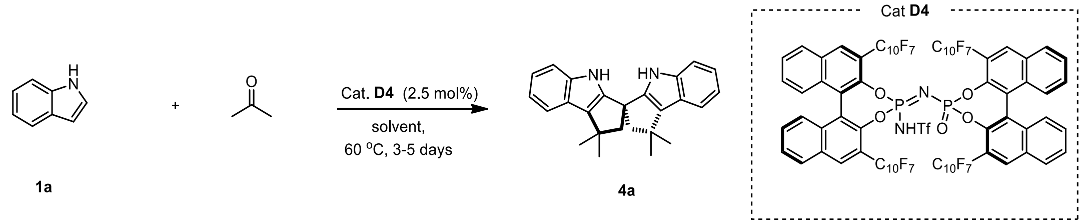

Entry	solvent	Time/day	Yield(%)	ee(%)
1	toluene	3	94	90
2	benzene	3	94	90
**3**	**THF**	**5**	**86**	**95**
4	1,4-dioxane	5	62	93
5	MeCN	5	67	88
6	DCE	3	95	88
7	$PhCF_3$	3	81	79
8	*c*-Hexane	3	79	70
9	$CHCl_3$	5	77	21

4.3 General procedure for synthesis of *rac*-4a-4bb

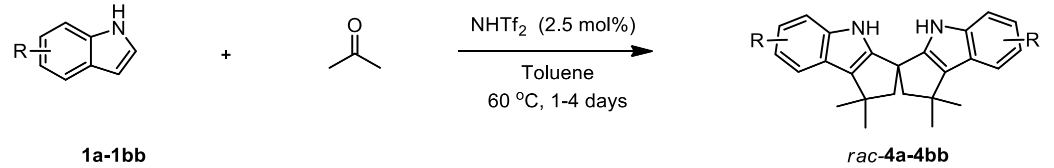

A 4 mL was charged with 0.5 mL of toluene, 1 (0.1 mmol, 1.0 equiv.), acetone (29 mg, 0.5 mmol, 5.0 equiv.), and Tf_2NH (0.25 mL, 0.0025 mmol, 2.5 mol%, 0.01 M solution in toluene). The vessel was sealed and stirred at 60 °C for 1–4 days. The reaction mixture was then directly subjected to silica gel column chromatography (petroleum ether:AcOEt = 20:1 to 5:1) to afford compound *rac*-**4a-4bb**.

4.4 General procedure for enantioselective reactions

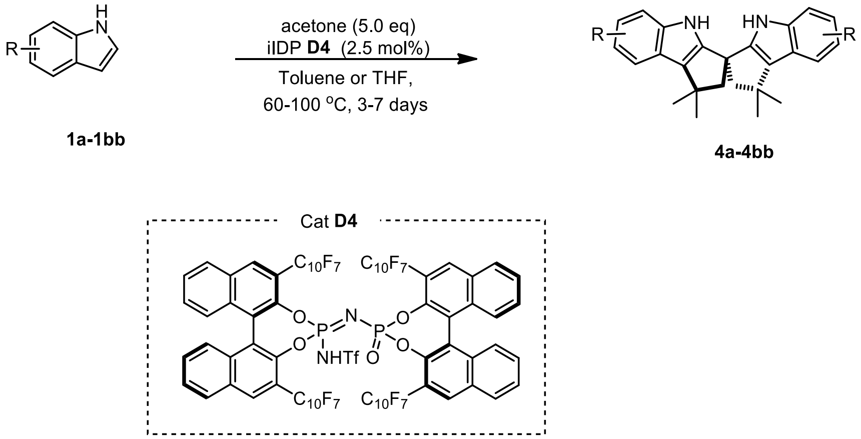

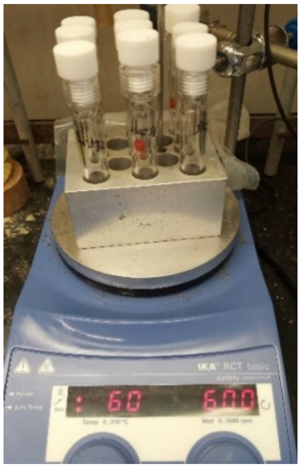

A 4 mL was charged with 1.0 mL of toluene or THF, **1a-1bb** (0.2 mmol, 1.0 eq), acetone (58.0 mg, 1.0 mmol, 5.0 eq), and iIDP **D4** (9.1 mg, 0.005 mmol, 2.5 mol%) were added. The vessel was sealed and stirred at 60 °C to 100 °C for 4-7 days. The reaction mixture was then subjected to silica gel column chromatography (Petroleum ether: AcOEt = 20:1 to 5:1) to afford compound **4a-4bb**.

5 Gram scale synthesis of *R*-4a and utility

5.1 General procedure for enantioselective gram scale synthesis of *R*-4a

A 200 mL heavy-wall flask was charged with 100 mL of toluene, **1** (4.68 g, 40 mmol, 1.0 eq), acetone (11.62 g, 7.41 mL, 200 mmol, 5.0 eq), and iIDP **D4** (1.819 g, 1 mmol, 2.5 mol%). The flask was sealed and stirred at 60 ^{o}C for 5 days. The solvents were removed and the reaction mixture was then subjected to silica gel column chromatography (Petroleum ether: AcOEt = 10:1) to afford compound **4a** (5.94 g, 84% yield, 90 % ee). The reaction also was performed with THF as solvent at 60 ^{o}C in 10 mmol (1.17 g) scale, 1.08 g (61% yield) of **4a** was obtained with 95% ee after 8 days' stirring.

5.2 Recrystallization of *R*-2a and *S*-4a

5.94 g of **R-4a** (90% ee) was dissolved in 20 mL of Et_2O, followed by the addition of 200 mL of hexane. The solution was evaporated to approximately 50 mL under reduced pressure, during which white precipitates formed. The precipitates were collected by filtration and washed with 10 mL of hexane, yielding 5.22 g of **R-4a** (88% recovery, >99% ee).

S-4a was obtained using **S,S-iIDP D4** under the same conditions. 500 mg of **S-4a** (90% ee) was dissolved in 4 mL of Et_2O, followed by the addition of 20 mL of hexane. The solution was evaporated to approximately 5 mL under reduced pressure, during which white precipitates formed. The precipitates were collected by filtration and washed with 1 mL of hexane, yielding 445.0 mg of **S-4a** (89% recovery, >99% ee).

SFC: (analytical SFC, Trefoil AMY-1 column, MeOH : CO_2 = 10 : 90, flow rate 0.6 mL/min, 2000psi, PDA wavelength: 280 nm) t_{R1}= 4.15 min ((*R*)-**4a**); t_{R2}= 5.73 min ((*S*)-**4a**).

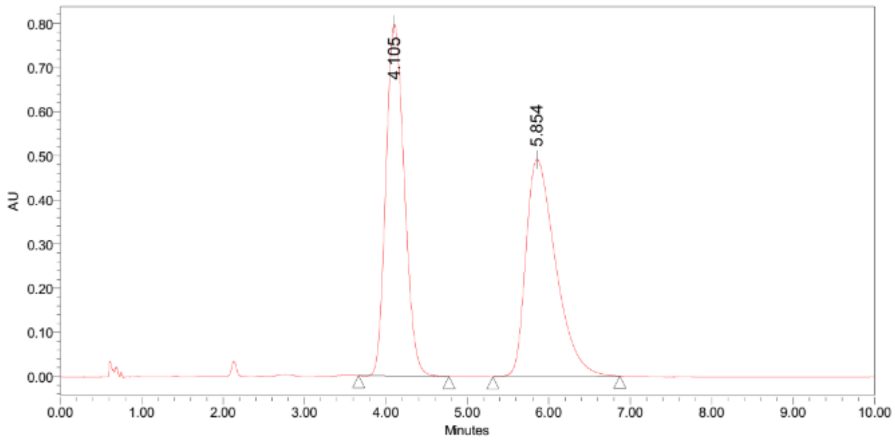

Peak Results

	Retention Time (min)	Area	% Area	Height	Int Type
1	4.105	12538715	50.06	796151	bb
2	5.854	12506892	49.94	491859	bb

SFC trace of (*R*)-**4a**

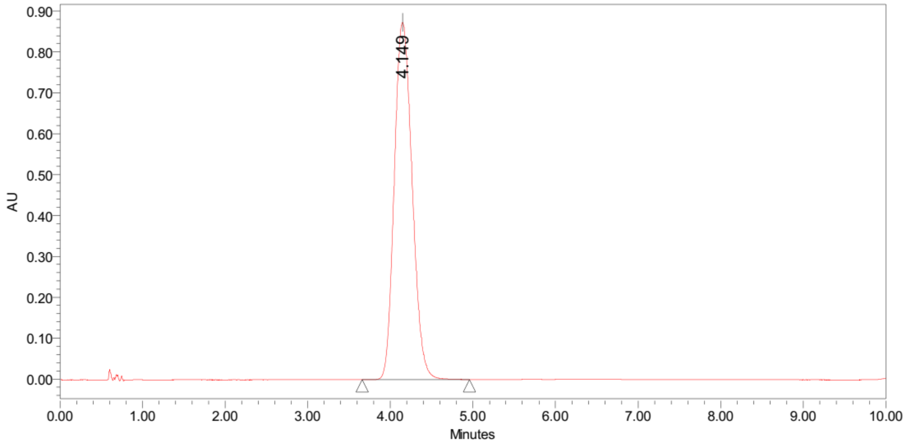

Peak Results

	Retention Time (min)	Area	% Area	Height	Int Type
1	4.149	13119384	100.00	873347	bb

SFC trace of (*R*)-**4a**

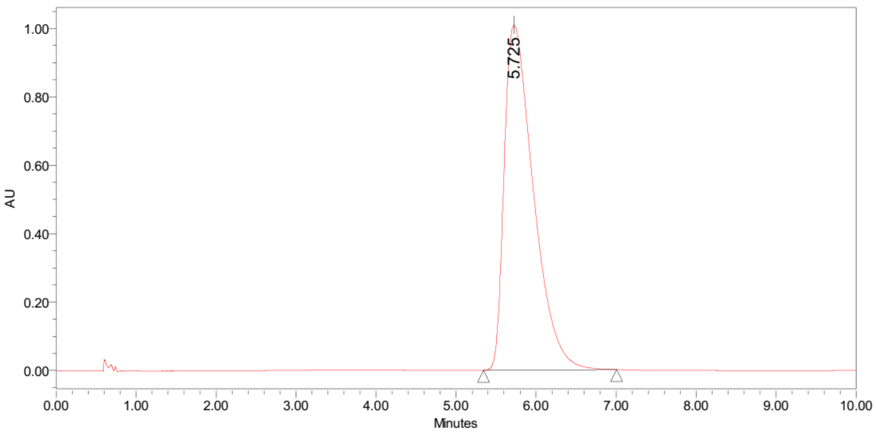

Peak Results

	Retention Time (min)	Area	% Area	Height	Int Type
1	5.725	25586534	100.00	1008792	bb

SFC trace of (*S*)-**4a**

5.3 Synthesis of 6a, 6b, and 6c

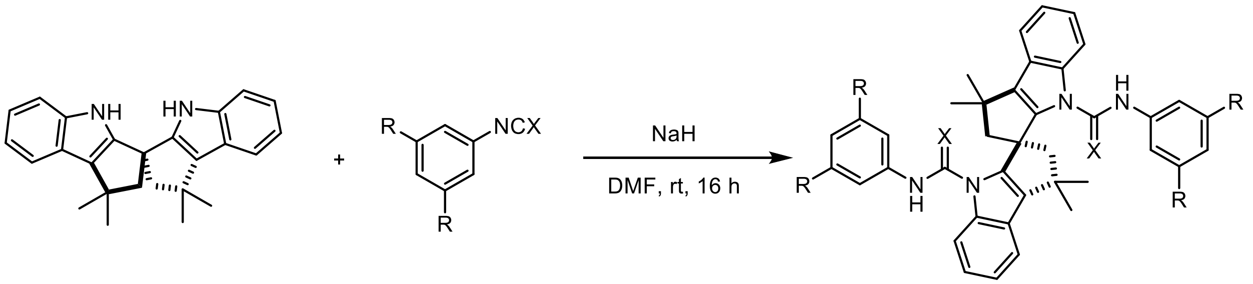

4a

5a: R = H, X = O
5b: R = H, X = S
5c: R = CF_3, X = S

6a: R = H, X = O
6b: R = H, X = S
6c: R = CF_3, X = S

In an oven-dried 5 mL flask under N_2, NaH (a mixture of 60% sodium hydride (w/w) in mineral oil, 16 mg, 0.4 mmol, 4 eq) was suspended in 0.5 mL of anhydrous DMF. A solution of **4a** (0.1 mmol, 1.0 eq) in 1 mL of dry DMF was added at 0 °C using a dry ice bath, and the mixture was stirred for 1 hour. Subsequently, **5a, 5b,** or **5c** (3.0 eq) in 0.5 mL of dry DMF was added dropwise. The reaction mixture was stirred at room temperature for 16 hours and then quenched with 5 mL of saturated $NaHCO_3$. The resulting mixture was extracted with ethyl acetate (5 mL × 3). After removal of the solvent by rotary evaporation, the residue was purified by silica gel flash column chromatography (PE:EtOAc = 10:1 to 3:1), affording the expected compound **6a, 6b,** or **6c.**

(*R*)-1,1,1',1'-tetramethyl-N4,N4'-diphenyl-1H,1'H-3,3'-spirobi[cyclopenta[b]indole]-4,4'(2H,2'H)-dicarboxamide (6a)

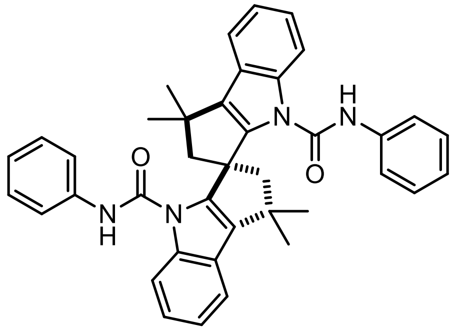

6a

Light yellow solid, m. p.237.0-238.8 °C. 44.4 mg, 89% yield. **[α]$_D^{25}$** = -131.2 (*c* = 0.5, CH_2Cl_2). **SFC**: ee >99% (analytical SFC, Trefoil AMY-1 column, MeOH : CO_2 = 20 : 80, flow rate 0.6 mL/min, 2000psi, PDA wavelength: 280 nm): minor isomer: t_R = 2.54 min; major isomer: t_R = 3.62 min. **^{1}H NMR** (400 MHz, $CDCl_3$) δ 8.27 – 8.05 (m, 4H), 7.54 (d, *J* = 7.7 Hz, 2H), 7.43 – 7.35 (m, 2H), 7.28 (t, *J* = 3.7 Hz, 2H), 7.07 (dd, *J* = 10.7, 4.7 Hz, 4H), 6.98 (dt, *J* = 7.0, 2.8 Hz, 6H), 2.84 (d, *J* = 13.4 Hz, 2H), 2.63 (d, *J* = 13.4 Hz, 2H), 1.50 (s, 6H), 1.34 (s, 6H). **^{13}C NMR** (101 MHz, $CDCl_3$) δ 149.53, 141.45, 140.40, 136.48, 135.00, 128.79, 124.28, 122.52, 119.35, 119.13, 114.93, 60.13, 53.96, 37.88, 29.58, 29.29. **IR** (neat) 3354, 3057, 2958, 2926, 2864, 1703, 1678, 1600, 1533, 1314, 1443, 1403, 1347, 1310, 1290, 1240, 1178, 1155, 1127, 1078, 881, 743, 687 cm^{-1}. **HRMS** (ESI) m/z $[M+Na]^+$ ($C_{39}H_{36}N_4O_2Na$), calcd.: 615.2736; found: 615.2728.

(*R*)-1,1,1',1'-tetramethyl-N4,N4'-diphenyl-1H,1'H-3,3'-spirobi[cyclopenta[b]indole]-4,4'(2H,2'H)-bis(carbothioamide) (6b)

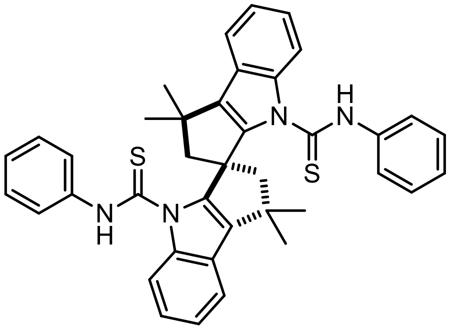

6b

Light yellow solid, m. p.240.4-241.7 °C. 43.0 mg, 69% yield. **[α]$_D^{25}$** = 109.2 (*c* = 0.5, CH_2Cl_2). **SFC**: ee >99% (analytical SFC, Trefoil IH-3 column, MeOH : CO_2 = 10 : 90, flow rate 0.6 mL/min, 2000psi, PDA wavelength: 280 nm): minor isomer: t_R = 4.58 min; major isomer: t_R = 5.39 min. **^{1}H NMR** (400 MHz, $CDCl_3$) δ 10.00 (s, 2H), 7.95 (d, *J* = 7.6 Hz, 2H), 7.58 (d, *J* = 7.0 Hz, 4H), 7.41 (d, *J* = 7.1 Hz, 2H), 7.31 – 6.97 (m, 10H), 2.83 (d, *J* = 13.2 Hz, 2H), 2.53 (d, *J* = 13.3 Hz, 2H), 1.45 (d, *J* = 7.4 Hz, 12H). **^{13}C NMR** (101 MHz, $CDCl_3$) δ 175.69, 143.18, 140.73, 137.37, 128.52, 126.26, 124.34, 123.04, 122.07, 121.66, 118.96, 113.40, 60.25, 53.45, 37.63, 29.86, 29.43. **IR** (neat) cm^{-1}. 3308, 3049, 2953, 2924, 2859, 1598, 1542, 1497, 1443, 1404, 1344, 1322, 1248, 1215, 1170, 1150, 1112, 1066, 1028, 955, 934, 901, 738, 687 **HRMS** (ESI) m/z $[M+H]^+$ ($C_{39}H_{37}N_4S_2$), calcd.: 625.2460; found: 625.2454.

(*R*)-N4,N4'-bis(3,5-bis(trifluoromethyl) phenyl)-1,1,1',1'-tetramethyl-1H,1'H-3,3'-spirobi [cyclopenta[b]indole]-4,4'(2H,2'H)-bis(carbothioamide) (6c)

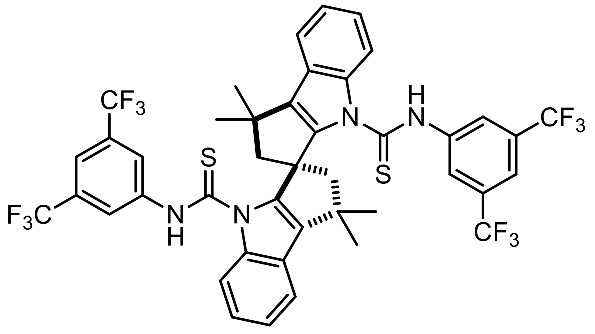

6c

Yellow solid, m. p.225.2-226.7 °C. 69.0 mg, 77%. $[\alpha]_D^{25}$ = 51.9 (*c* = 0.5, CH_2Cl_2). **SFC**: ee >99% (analytical SFC, Trefoil IJ-3 column, MeCN (contains 0.05% TFA) : CO_2= 3 : 97, flow rate 0.6 mL/min, 2000psi, PDA wavelength: 280 nm): minor isomer: t_R = 6.04 min; major isomer: t_R = 7.36 min. **^{1}H NMR** (400 MHz, Acetone-D$_6$) δ 11.67 (s, 2H), 8.35 (s, 4H), 7.99 (s, 2H), 7.71 (s, 2H), 7.47 (s, 2H), 7.18 (t, *J* = 17.9 Hz, 4H), 2.89 (d, *J* = 12.4 Hz, 2H), 2.65 (d, *J* = 12.9 Hz, 2H), 1.54 (s, 6H), 1.47 (s, 6H). **^{13}C NMR** (101 MHz, Acetone-D$_6$) δ 177.61, 143.25, 141.26, 139.99, 133.29, 132.19, 131.85, 131.52, 131.19, 127.25, 124.54, 124.25, 123.57, 122.69, 121.83, 119.13, 118.98, 114.16, 78.45, 60.12, 53.77, 37.78. **^{19}F NMR** (282 MHz, Acetone) δ 114.05. **IR** (neat) 3300, 2959, 1706, 1546, 1471, 1446, 1380, 1345, 1317, 1304, 1273, 1170, 1129, 1109, 1067,986, 967, 886, 847 cm^{-1}. **HRMS** (ESI) m/z $[M+H]^+$ ($C_{43}H_{33}F_{12}N_4S_2$), calcd.: 897.1955; found: 897.1954.

5.4 Synthesis of 7a and 7b

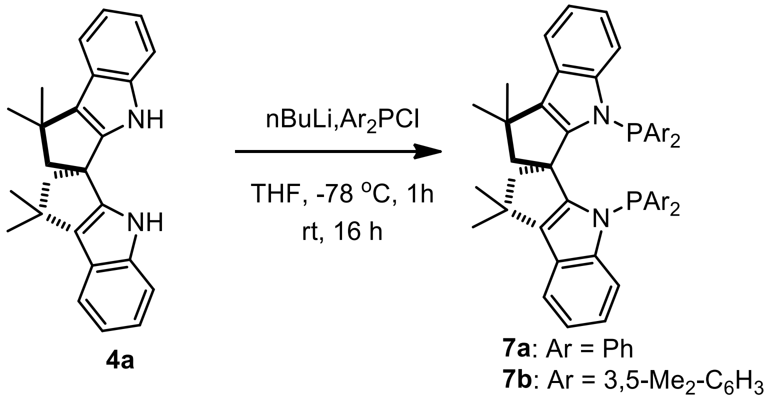

4a

7a: Ar = Ph
7b: Ar = 3,5-Me$_2$-C$_6$H$_3$

In an oven-dried 25 mL flask under N_2, **4a** (1.0 eq) and 5 mL of dry THF were added, then cooled to 0 °C using an ice bath. nBuLi (1.6 M in hexanes, 2.2 eq) was added dropwise. The mixture was stirred at room temperature for 1 hour, and Ar_2PCl (2.2 eq) in 2 mL of dry THF was added dropwise at 0 °C. The mixture was stirred overnight at room temperature and then quenched with 5 mL of saturated NH_4Cl. The reaction mixture was extracted with CH_2Cl_2. After removal of the solvent

by rotary evaporation, the residue was purified by silica gel flash column chromatography (DCM:Petroleum ether = 1:10 to 1:3), affording the desired compounds **7a** and **7b** as a white solid.

(*R*)-4,4'-bis(diphenylphosphino)-1,1,1',1'-tetramethyl-2,2',4,4'-tetrahydro-1H,1'H-3,3'-spirobi[cyclopenta[b]indole] (7a)

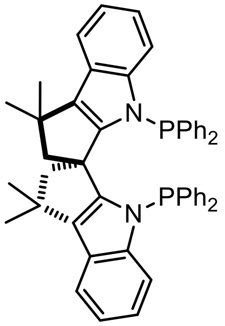

7a

The reaction was performed at 0.5 mmol scale and affording the expected compound **7a** as a white solid (317.7 mg, 88% yield). m. p. >300 °C. $[\alpha]_D^{25}$ = 29.0 (c = 0.5, CH_2Cl_2). **SFC**: ee > 99% (ee was determined by the recovered SPINDOLE **4a** from treating **7a** with 2.0 eq TBAF in THF at rt.) **^{1}H NMR** (400 MHz, $CDCl_3$) δ 7.59 (d, J = 7.6 Hz, 2H), 7.56 – 7.45 (m, 4H), 7.39 (dd, J = 4.2, 2.1 Hz, 6H), 7.08 (dt, J = 7.4, 3.8 Hz, 8H), 7.01 – 6.92 (m, 4H), 6.80 – 6.72 (m, 2H), 6.59 (d, J = 8.4 Hz, 2H), 2.88 (d, J = 13.1 Hz, 2H), 2.53 (d, J = 13.1 Hz, 2H), 1.50 (s, 6H), 1.43 (s, 6H). **^{13}C NMR** (101 MHz, $CDCl_3$) δ 149.70, 149.49, 144.24, 144.13, 136.26, 136.06, 135.01, 134.87, 131.65, 131.43, 130.97, 130.94, 130.91, 130.54, 130.35, 129.14, 128.55, 128.52, 128.48, 128.06, 127.95, 127.90, 127.01, 120.37, 120.20, 118.45, 116.06, 61.43, 52.31, 38.23, 30.25, 29.28. **^{31}P NMR** (121 MHz, $CDCl_3$) δ 35.25. **IR** (neat) 3055, 2947, 2925, 2859, 1606, 1586, 1481, 1444, 1433, 1380, 1359, 1279, 1228,1186, 1151, 1121, 1108, 1094, 1021, 963, 945, 742, 692 cm^{-1}. **HRMS** (ESI) m/z $[M+H]^+$ ($C_{49}H_{45}N_2P_2$), calcd.: 723.3058; found: 723.3048.

(*R*)-4,4'-bis(bis(3,5-dimethylphenyl)phosphino)-1,1,1',1'-tetramethyl-2,2',4,4'-tetrahydro-1H,1'H-3,3'-spirobi[cyclopenta[b]indole] (7b)

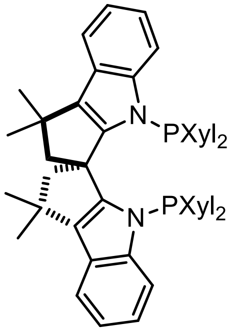

7b

The reaction was performed at 0.2 mmol scale and affording the expected compound **7b** as a white solid (136.8 mg, 82% yield). m. p. >300 °C. $[\alpha]_D^{25}$ = 39.2 (c = 0.5, CH_2Cl_2). **SFC**: ee > 99% (analytical SFC, Trefoil AMY-1 column, IPA: CO_2 = 10 : 90, flow rate 0.6 mL/min, 2000psi, PDA wavelength: 280 nm): major isomer: t_R = 1.84 min; minor isomer: t_R = 2.13 min. **^{1}H NMR** (400 MHz, $CDCl_3$) δ 7.56 (d, J = 7.7 Hz, 2H), 7.21 (d, J = 8.4 Hz, 4H), 7.05 (ddd, J = 9.7, 5.7, 2.4 Hz, 4H), 6.84 – 6.73 (m, 4H), 6.69 (d, J = 6.4 Hz, 6H), 2.81 (d, J = 13.0 Hz, 2H), 2.46 (d, J = 13.0 Hz, 2H), 2.29 (s, 12H), 1.87 (s, 12H), 1.46 (d, J = 11.8 Hz, 12H). **^{13}C NMR** (101 MHz, $CDCl_3$) δ 149.97, 149.76, 144.35, 144.24, 137.79, 137.71, 137.18,

137.14, 136.32, 136.12, 135.15, 135.03, 131.15, 130.45, 130.34, 130.10, 129.55, 128.34, 128.15, 126.97, 120.06, 119.77, 118.06, 116.32, 61.03, 52.41, 38.15, 30.25, 29.33, 21.41, 21.31, 21.05. **^{31}P NMR** (121 MHz, $CDCl_3$) δ 37.32. **IR** (neat) 3024, 2950, 2920, 2859, 1713, 1684, 1600, 1583, 1443, 1358, 1330, 1279, 1228, 1187, 1150, 1122, 1108, 1072, 1020, 964, 845, 741, 691 cm^{-1}. **HRMS** (ESI) m/z [M+H]$^+$ ($C_{57}H_{61}N_2P_2$), calcd.: 835.4310; found: 835.4315.

5.5 Synthesis of (*R*)-4-(diphenylphosphino)-1,1,1',1'-tetramethyl-2,2',4,4'-tetrahydro-1H,1'H-3,3'-spirobi[cyclopenta[b]indole] (8)

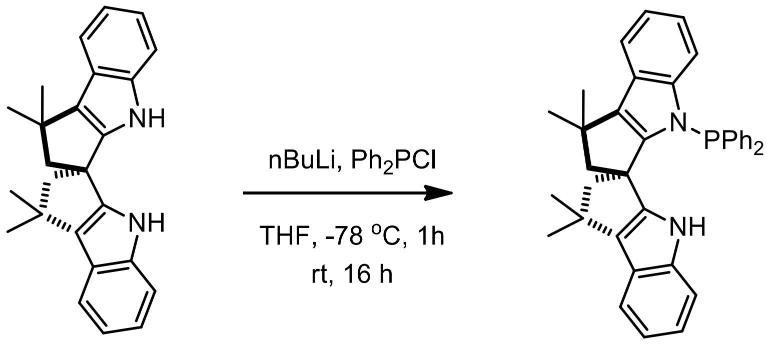

4a　　　　8

In an oven-dried 25 mL flask under N_2, **4a** (177.0 mg, 0.5 mmol, 1.0 eq) and 5 mL of dry THF were added, then cooled to -78 °C using a dry ice/acetone bath. nBuLi (1.6 M in hexanes, 0.35 mL, 0.55 mmol, 1.1 eq) was added dropwise. The mixture was stirred at -78 °C for 1 hour, then at room temperature for 30 minutes. Ph_2PCl (121 mg, 0.55 mmol, 1.1 eq) in 1 mL of dry THF was added dropwise at -78 °C and stirred at this temperature for 1 hour. The mixture was warmed to room temperature, stirred for 2 hours, and then quenched with 5 mL of saturated NH_4Cl. The reaction mixture was extracted with CH_2Cl_2 (10 mL × 3). After removal of the solvent by rotary evaporation, the residue was purified by silica gel flash column chromatography (DCM:Petroleum ether = 1:10 to 1:2), affording the expected compound **8** as a white solid (239.4 mg, 89% yield).White solid, m. p.212.1-213.9 °C. **$[\alpha]_D^{25}$** = -1.1 (*c* = 0.5, CH_2Cl_2). **SFC**: ee > 99% (analytical SFC, Trefoil AMY-1 column, IPA : CO_2 = 10 : 90, flow rate 0.6 mL/min, 2000psi, PDA wavelength: 280 nm): minor isomer: t_R = 4.48 min; major isomer: t_R = 5.17 min. **^{1}H NMR** (400 MHz, $CDCl_3$) δ 7.69 (dd, *J* = 8.3, 3.2 Hz, 2H), 7.46 – 7.34 (m, 6H), 7.32 – 7.25 (m, 3H), 7.24 – 7.14 (m, 6H), 6.92 (ddd, *J* = 22.9, 12.1, 4.7 Hz, 2H), 3.46 (dd, *J* = 13.0, 2.2 Hz, 1H), 2.86 (q, *J* = 13.1 Hz, 2H), 2.76 (d, *J* = 13.0 Hz, 1H), 1.69 (dd, *J* = 19.4, 11.8 Hz, 12H). **^{13}C NMR** (101 MHz,

CDCl3) δ 148.98, 148.79, 145.28, 145.26, 144.30, 144.18, 141.09, 135.74, 135.66, 135.54, 135.52, 132.48, 132.25, 131.10, 131.05, 130.87, 130.68, 129.45, 128.64, 128.52, 128.47, 128.37, 128.30, 127.54, 127.52, 126.57, 126.55, 123.84, 120.85, 120.58, 120.47, 119.31, 118.58, 118.27, 115.70, 111.76, 62.71, 61.72, 61.66, 50.46, 50.42, 39.42, 38.54, 31.03, 31.00, 30.32, 30.12, 28.87. **^{31}P NMR** (121 MHz, $CDCl_3$) δ 37.12. **IR** (neat) 3362, 3054, 2953, 2940, 2914, 1603, 1563, 1480, 1452, 1433, 1371, 1343, 1326, 1289, 1277, 1227, 1183, 1157, 1103, 1061, 1017, 969, 745, 694 cm^{-1}. **HRMS** (ESI) m/z [M+H]$^+$ ($C_{37}H_{36}N_2P$), calcd.: 539.2616; found: 539.2612.

5.6 Synthesis of 10a and 10b

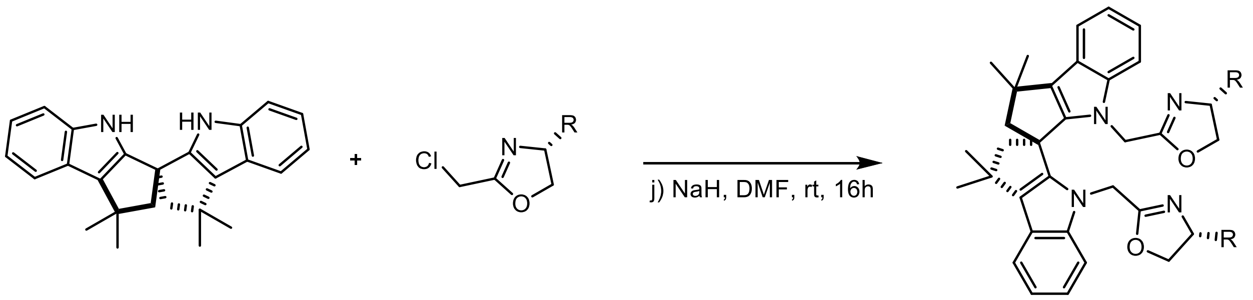

4a **9a**: R = H **9b**: R = Et **10a**: R = H **10b**: R = Et

In an oven-dried 5 mL flask under N_2, NaH (a mixture of 60% sodium hydride (w/w) in mineral oil, 16 mg, 0.4 mmol, 4 eq) was suspended in 0.5 mL of anhydrous DMF. **4a** (0.1 mmol, 1.0 eq) in 1 mL of dry DMF was added at 0 °C using a dry ice bath, and the mixture was stirred for 1 hour. Then, **9a** or **9b** (0.3 mmol, 3.0 eq) in 0.5 mL of dry DMF was added dropwise. The mixture was stirred at room temperature for 16 hours and quenched with 5 mL of saturated $NaHCO_3$. The reaction mixture was then extracted with EA (5 mL × 3). After removal of the solvent by rotary evaporation, the residue was purified by silica gel flash column chromatography (DCM:MeOH = 100:0 to 10:1), affording the expected compounds **10a** and **10b** as white solids.

(*R*)-2,2'-((1,1,1',1'-tetramethyl-1H,1'H-3,3'-spirobi[cyclopenta[b] indol]-4,4'(2H,2'H)-diyl)bis(methylene)) bis(4,5-dihydrooxazole) (10a)

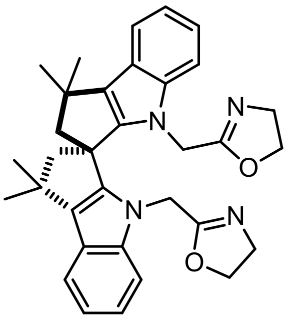

10a

Light yellow solid, m. p.235.5-236.9 °C. 42.7 mg, 82% yield. **$[\alpha]_D^{25}$** = 55.9 (*c* = 1.0, CH_2Cl_2). **SFC**: ee >99% (analytical SFC, Trefoil AMY-1 column, IPA : CO_2 = 10 : 90, flow rate 0.6 mL/min, 2000psi, PDA wavelength: 280 nm): major isomer: t_R = 9.24 min; minor isomer: t_R = 9.94 min. **^{1}H NMR** (400 MHz, $CDCl_3$) δ 7.62 (dd, *J* = 6.9, 1.5 Hz, 2H), 7.42 – 7.34 (m, 2H), 7.27 – 7.13 (m, 4H), 4.60 (s, 4H), 4.21 – 4.05 (m, 4H), 3.95 – 3.67 (m, 4H), 3.08 (d, *J* = 13.4 Hz, 2H), 2.64 (d, *J* = 13.4 Hz, 2H), 1.61 (s, 6H), 1.55 (s, 6H). **^{13}C NMR** (101 MHz, $CDCl_3$) δ 163.49, 143.18, 141.90, 127.55, 123.09, 121.13, 119.59, 118.59, 110.26, 67.79, 61.25, 54.29, 49.05, 40.92, 38.79, 30.94, 29.68. **IR** (neat) 3053, 2951, 2931, 2862, 1668, 1456, 1378, 1358, 1301, 1181, 1165, 1114, 1047, 1004, 980, 953, 914, 841, 793, 739 cm^{-1}. **HRMS** (ESI) m/z $[M+H]^+$ ($C_{33}H_{37}N_4O_2$), calcd.: 521.2917; found: 521.2911.

(*4R,4'R*)-2,2'-(((R)-1,1,1',1'-tetramethyl-1H,1'H-3,3'-spirobi [cyclopenta[b]indol] -4,4'(2H,2'H)-diyl)bis(methylene)) bis(4-ethyl-4,5-dihydrooxazole) (10b)

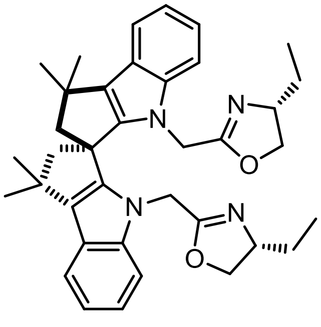

10b

Light yellow solid, m. p.95.0-96.4 °C. 48.4 mg, 84% yield. **$[\alpha]_D^{25}$** = 47.7 (*c* = 1.0, CH_2Cl_2). **SFC**: de > 99% (analytical SFC, Trefoil CEL-1 column, MeOH : CO_2 = 5 : 95, flow rate 0.6 mL/min, 2000psi, PDA wavelength: 220 nm): major isomer: t_R = 5.55 min; minor isomer: t_R = 5.96 min. **^{1}H NMR** (400 MHz, $CDCl_3$) δ 7.58 (d, *J* = 6.9 Hz, 2H), 7.43 – 7.31 (m, 2H), 7.20 – 7.09 (m, 4H), 4.67 – 4.44 (m, 4H), 4.17 (t, *J* = 8.9 Hz, 2H), 4.06 – 3.90 (m, 2H), 3.76 (t, *J* = 7.9 Hz, 2H), 3.05 (d, *J* = 13.4 Hz, 2H), 2.59 (d, *J* = 13.4 Hz, 2H), 1.65 – 1.54 (m, 8H), 1.51 (s, 6H), 1.46 – 1.37 (m, 2H), 0.87 (t, *J* = 7.4 Hz, 6H). **^{13}C NMR** (101 MHz, $CDCl_3$) δ 162.36, 143.25, 141.94, 127.53, 123.13, 121.07, 119.58, 118.54, 110.43, 72.44, 67.59, 61.10, 49.11, 41.10, 38.82, 30.89, 29.79, 28.26, 9.98. **IR** (neat) 3053, 2955, 2931, 2862, 1669, 1456, 1376, 1360, 1299, 1187, 1165, 1113, 1044, 970, 842, 792, 738 cm^{-1}. **HRMS** (ESI) m/z $[M+H]^+$ ($C_{37}H_{45}N_4O_2$), calcd.: 577.3543; found: 577.3538.

5.7 Synthesis of 11a and 11b

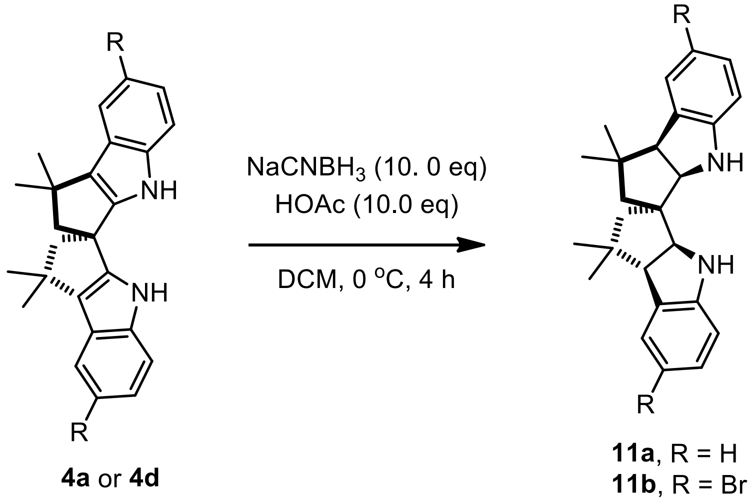

4a or **4d**

11a, R = H
11b, R = Br

A 4 mL vial was charged with **4a** or **4d** (1.0 eq), $NaCNBH_3$ (10.0 eq), and DCM (0.1 M). The mixture was cooled to 0 °C, and HOAc (10.0 eq) was added dropwise. The reaction mixture was stirred for 4 hours at this temperature, then quenched with saturated $NaHCO_3$ and extracted with DCM (15 mL × 3). After removal of the solvent by rotary evaporation, the residue was purified by silica gel flash column chromatography (DCM:PE = 1:3), affording the expected compounds **11a** and **11b**.

(*3R,3aR,3a'S,8bS,8b'R*)-1,1,1',1'-tetramethyl-2,2',3a,3a',4,4',8b,8b'-octahydro -1H,1'H-3,3'-spirobi[cyclopenta[b]indole] (11a)

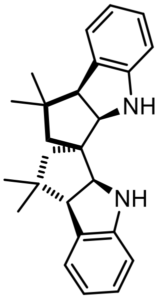

11a

The reaction was performed with 1 mmol **4a**, **11a** was obtained as white solid (236.1 mg, 66% yield). m. p.141.2-142.7 °C. $[\alpha]_D^{25}$ = -24.7 (*c* = 0.52, CH_2Cl_2). **SFC**: ee >99% (analytical SFC, Trefoil CEL-1 column, MeOH : CO_2 = 10 : 90, flow rate 0.6 mL/min, 2000psi, PDA wavelength: 220 nm): major isomer: t_R = 4.68 min; minor isomer: t_R = 6.22 min. **^{1}H NMR** (400 MHz, $CDCl_3$) δ 7.05 (d, *J* = 7.6 Hz, 4H), 6.68 (dd, *J* = 29.9, 7.5 Hz, 4H), 4.24 (d, *J* = 7.7 Hz, 2H), 3.42 (d, *J* = 7.7 Hz, 2H), 1.84 (dd, *J* = 31.9, 13.6 Hz, 4H), 1.29 (s, 6H), 0.73 (s, 6H). **^{13}C NMR** (101 MHz, $CDCl_3$) δ 150.98, 131.23, 127.46, 125.77, 117.91, 108.59, 77.32, 77.00, 76.68, 74.54, 57.99, 57.48, 48.76, 40.51, 33.58, 29.02. **IR** (neat) 3403, 3050, 2913, 2858, 1717, 1607, 1483, 1464, 1398,

1386, 1362, 1326, 1305, 1245, 1184, 1167, 1152, 1047, 1027, 928, 845, 810, 739, 711 cm^{-1}. **HRMS** (ESI) m/z [M+H]$^+$ ($C_{25}H_{31}N_2$), calcd.: 359.2487; found: 359.2482.

(*3R,3aR,3a'S,8bS,8b'R*)-7,7'-dibromo-1,1,1',1'-tetramethyl-2,2',3a,3a',4,4',8b,8b'-octahydro-1H,1'H-3,3'-spirobi[cyclopenta[b]indole (11b)

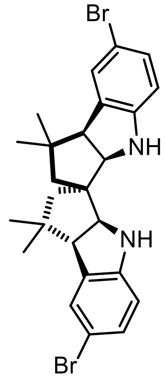

11b

The reaction was performed at 0.1 mmol of **4d** (77% ee), **11b** was obtained as light yellow solid (32.2 mg, 62% yield). m. p.122.2-123.5 °C. $[\alpha]_D^{25}$ = -46.0 (*c* = 0.45, CH_2Cl_2). **SFC**: ee = 77% (analytical SFC, Trefoil AMY-1 column, IPA : CO_2 = 20 : 80, flow rate 0.6 mL/min, 2000psi, PDA wavelength: 220 nm): major isomer: t_R = 3.45 min; minor isomer: t_R = 4.08 min. **^{1}H NMR** (400 MHz, $CDCl_3$) δ 7.19 – 7.01 (m, 4H), 6.48 (d, *J* = 8.0 Hz, 2H), 4.20 (d, *J* = 7.7 Hz, 2H), 3.35 (d, *J* = 7.7 Hz, 2H), 1.79 (dd, *J* = 33.3, 13.6 Hz, 4H), 1.24 (s, 6H), 0.72 (s, 6H). **^{13}C NMR** (101 MHz, $CDCl_3$) δ 149.93, 133.51, 130.12, 128.47, 109.91, 109.39, 77.32, 77.00, 76.68, 74.75, 57.90, 57.61, 48.66, 40.60, 33.45, 28.95. **IR** (neat) 3396, 2954, 2863, 1602, 1477, 1457, 1425, 1384, 1363, 1287,1245, 1167, 1120, 1107, 1048, 924, 874, 808, 758, 715 cm^{-1}. **HRMS** (ESI) m/z [M+H]$^+$ ($C_{25}H_{29}Br_2N_2$), calcd.: 515.0697; found: 515.0684.

5.8 Synthesis of (*R*)-1,1,1',1'-tetramethyl-5,5'-bis(4,4,5,5-tetramethyl-1,3,2-dioxaborolan-2-yl)-2,2',4,4'-tetrahydro-1H,1'H-3,3'-spirobi [cyclopenta[b]indole] (12)

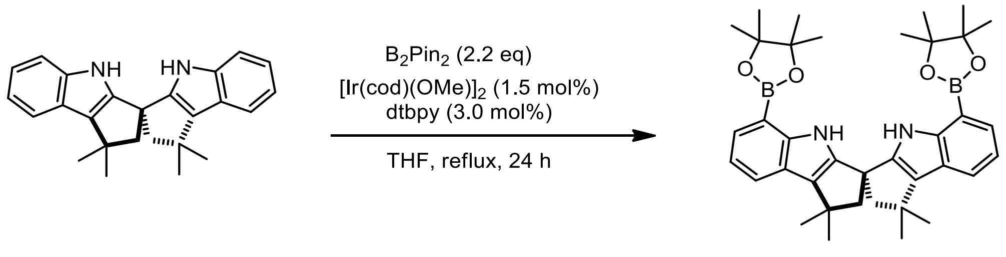

Charge a 4.0 mL vessel with $[Ir(cod)(OMe)]_2$ (1.98 mg, 1.5 mol%), 4,4'-bis(1,1-di-tert-butyl)-2,2'-bipyridine (1.01 mg, 3.0 mol%), B_2pin_2 (56.2 mg, 0.22 mmol, 2.2 equiv.), and dry THF (1.0 mL) in an N_2-filled glove box. Stir the reaction mixture for 2 minutes at room temperature. Add **4a** (35.4 mg, 0.1 mmol) to the reaction mixture. Seal the vessel and place it into a pre-heated

aluminum block at 70 °C. Stir the reaction mixture for 24 hours. After cooling to room temperature, remove the solvent by rotary evaporation. Purify the residue by silica gel flash column chromatography (petroleum ether:ethyl acetate = 10:1 to 5:1), affording the expected compound **12** as a white solid (40.0 mg, 66% yield). Light yellow solid, m.p. 289.0–290.8 °C. **$[\alpha]_D^{25}$** = 134.4 (c = 1.0, CH_2Cl_2). **SFC**: ee > 99% (analytical SFC, Trefoil CEL-1 column, MeOH : CO_2 = 20 : 80, flow rate 0.6 mL/min, 2000psi, PDA wavelength: 280 nm): major isomer: t_R = 2.48 min; minor isomer: t_R = 4.36 min. **^{1}H NMR** (400 MHz, CD_2Cl_2) δ 8.73 (s, 2H), 7.75 (d, J = 7.7 Hz, 2H), 7.56 (d, J = 6.9 Hz, 2H), 7.14 (t, J = 7.3 Hz, 2H), 2.88 (d, J = 13.1 Hz, 2H), 2.68 (d, J = 13.1 Hz, 2H), 1.68 (s, 6H), 1.57 (s, 6H), 1.36 (s, 24H). **^{13}C NMR** (101 MHz, CD_2Cl_2) δ 146.65, 144.56, 128.20, 127.12, 122.70, 121.88, 118.96, 84.00, 62.38, 49.77, 39.04, 30.50, 30.18, 24.93, 24.68. **IR** (neat) 3450, 2974, 2932, 2864, 1588, 1566, 1491, 1453, 1411, 1374, 1345, 1312, 1267, 1213, 1190, 1169, 1130, 1108, 1047, 964, 862, 846, 797 cm^{-1}. **HRMS** (ESI) m/z $[M+H]^+$ ($C_{37}H_{49}B_2N_2O_4$), calcd.: 607.3878; found: 607.3891.

5.9 Synthesis of [(*R*)-4,4'-bis(diphenylphosphino)-1,1,1',1'-tetramethyl-2,2',4,4'-tetrahydro-1H,1'H-3,3'-spirobi[cyclopenta[b]indole]]palladium(II) chloride (13)

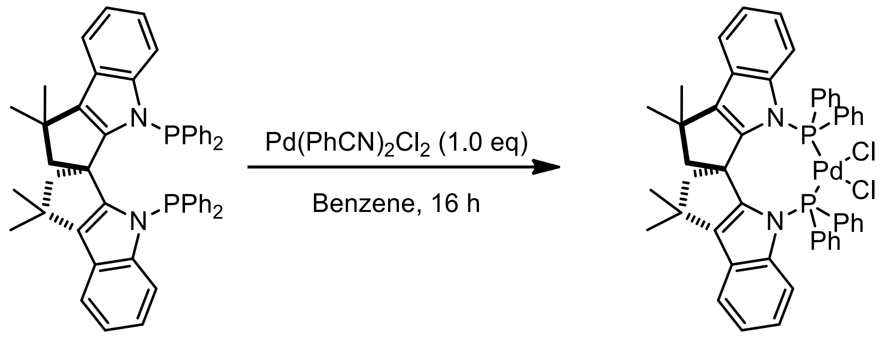

7a **13**

In an oven-dried 10 mL flask, diphosphine **7a** (13.0 mg, 0.018 mmol, 1.0 equiv.), bis(benzonitrile)palladium(II) chloride (7.1 mg, 0.018 mmol), and benzene (1.2 mL) were stirred at room temperature. After 16 hours, the complex was precipitated upon the addition of hexanes (5 mL) as an orange solid, which was collected by filtration and washed with hexanes. The solid was redissolved in dichloromethane (DCM), and the volatiles were evaporated in vacuo to yield complex **13** as an orange solid (14.6 mg, 90% yield). Yellow solid, m.p. > 300 °C. **$[\alpha]_D^{25}$** = 17.4 (c = 0.5, CH_2Cl_2). **^{1}H NMR** (400 MHz, $CDCl_3$) δ 8.25 (dd, J = 12.3, 7.4 Hz, 4H), 7.78 (dd, J = 11.6,

7.5 Hz, 4H), 7.49 (dt, J = 14.6, 7.1 Hz, 6H), 7.28 (s, 2H), 7.04 (dt, J = 20.9, 7.2 Hz, 8H), 6.82 (t, J = 7.9 Hz, 2H), 6.36 (d, J = 8.4 Hz, 2H), 2.23 (d, J = 12.6 Hz, 2H), 2.07 (d, J = 12.5 Hz, 2H), 1.39 (s, 6H), 1.16 (s, 6H). **^{13}C NMR** (101 MHz, $CDCl_3$) δ 147.08, 145.80, 139.66, 135.13, 133.30, 132.95, 132.70, 132.36, 132.01, 131.02, 128.48, 128.36, 128.23, 127.05, 122.89, 122.28, 118.42, 117.44, 57.98, 54.98, 36.60, 29.35, 27.93. **^{31}P NMR** (121 MHz, $CDCl_3$) δ 77.33. **IR** (neat) 3058, 2953, 2927, 2864, 1585, 1479, 1435, 1361, 1309, 1280, 1240, 1186, 1149, 1093, 1028, 998, 978, 943, 889, 841, 818, 774 cm^{-1}. **HRMS** (ESI) m/z $[M-Cl]^+$ ($C_{49}H_{44}N_2P_2PdCl$), calcd.: 863.1703; found: 863.1705.

5.10 Synthesis of (R)-(1,1,1',1'-tetramethyl-1H,1'H-3,3'-spirobi[cyclopenta[b]indol]-4,4'(2H,2'H)-diyl)bis(diphenylphosphine oxide) (14)

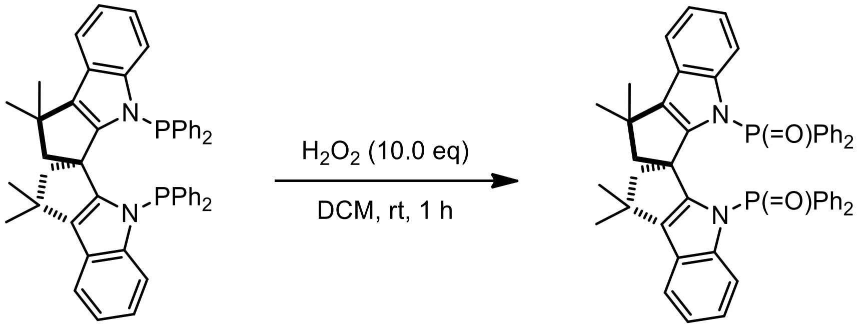

7a **14**

A 2 mL vial was charged with 1 mL of DCM, **7a** (0.1 mmol, 1.0 equiv.), and 50 μL of H_2O_2 (30% in water, approximately 0.5 mmol, 5 equiv.). The mixture was stirred for 1 hour at room temperature. The reaction mixture was then directly subjected onto a silica gel column for chromatography (petroleum ether:acetate = 5:1) to afford compound **14** (75.4 mg, 99% yield). White solid, m.p. > 300 °C. **$[\alpha]_D^{25}$** = −83.3 (c = 1.0, CH_2Cl_2). SFC: ee > 99% (ee was determined by the recovered SPINDOLE **4a** from treating **14** with 2.0 equiv. TBAF in THF at rt). **^{1}H NMR** (400 MHz, $CDCl_3$) δ 7.84 (dd, J = 12.4, 7.3 Hz, 4H), 7.66 – 7.42 (m, 12H), 7.34 (t, J = 7.5 Hz, 2H), 7.11 (td, J = 7.7, 3.3 Hz, 4H), 7.01 (t, J = 7.5 Hz, 2H), 6.70 (t, J = 7.8 Hz, 2H), 6.43 (d, J = 8.4 Hz, 2H), 2.78 (d, J = 12.7 Hz, 2H), 2.43 (d, J = 12.7 Hz, 2H), 1.46 (d, J = 6.9 Hz, 12H). **^{13}C NMR** (101 MHz, $CDCl_3$) δ 151.06, 151.03, 142.44, 142.39, 133.15, 132.28, 132.23, 132.18, 131.99, 131.96, 131.92, 131.73, 131.57, 131.51, 130.50, 128.48, 128.35, 128.28, 128.15, 126.58, 126.52, 120.85, 120.81, 118.47, 115.09, 60.90, 54.35, 37.45, 30.17, 28.79. **^{31}P NMR** (121 MHz,

CDCl3) δ 25.32. **IR** (neat) 3190, 2947, 2923, 2859, 1579, 1448, 1437, 1359, 1312, 1284, 1223, 1182, 1152, 1123, 1105, 1080, 1022, 968, 845, 820, 749, 729, 693 cm^{-1}. **HRMS** (ESI) m/z $[M+H]^+$ ($C_{49}H_{45}N_2O_2P_2$), calcd.: 755.2956; found: 755.2953.

5.11 Synthesis of (*R*)-N-(3,5-bis(trifluoromethyl)phenyl)-4'-(diphenylphosph ino)-1,1,1',1'-tetramethyl-2',4'-dihydro-1H,1'H-3,3'-spirobi [cyclo penta[b]indole]-4(2H)-carbothioamide (15)

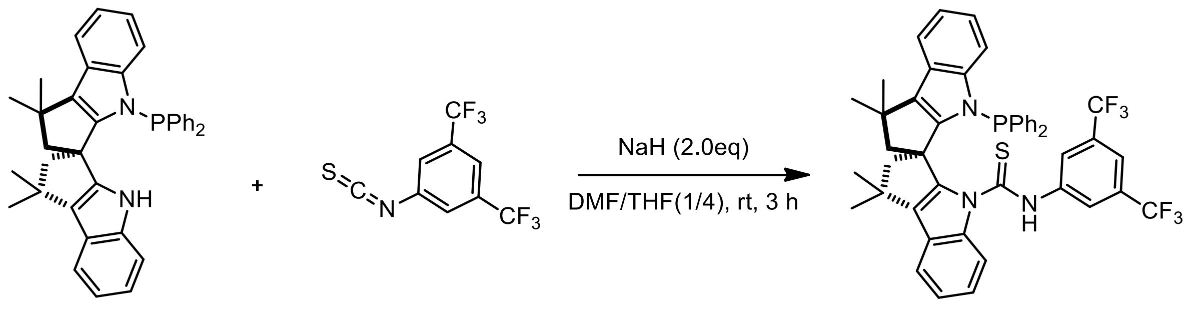

8 **5c** **15**

In an oven-dried 5 mL flask under N_2, NaH (a mixture of 60% sodium hydride (w/w) in mineral oil, 16 mg, 0.4 mmol, 2 equiv.) was suspended in 1 mL of anhydrous DMF/THF (v/v = 1/4). **8** (0.2 mmol, 1.0 equiv.) in 1 mL of anhydrous DMF/THF (v/v = 1/4) was added at 0 °C with an ice bath, and the mixture was stirred for 0.5 hours. Then, **5c** (0.4 mmol, 2.0 equiv.) in 0.5 mL of anhydrous DMF/THF (v/v = 1/4) was added dropwise. The mixture was stirred at room temperature for 3 hours and quenched with 15 mL of saturated $NaHCO_3$. The reaction mixture was then extracted with ethyl acetate (EA, 5 mL × 3). After removal of solvent by rotary evaporation, the residue was purified by silica gel flash column chromatography (DCM:petroleum ether = 1:5 to 1:3), affording the expected compound **15** as a yellow solid (131.1 mg, 81% yield). Yellow solid, m.p. 132.5–133.8 °C. $[\alpha]_D^{25}$ = 19.6 (c = 0.58, CH_2Cl_2). **SFC**: ee > 99% (analytical SFC, Trefoil CEL-1 column, IPA : CO_2 = 10 : 90, flow rate 0.6 mL/min, 2000psi, PDA wavelength: 280 nm): major isomer: t_R = 4.96 min; minor isomer: t_R = 5.42 min. **^{1}H NMR** (400 MHz, CDCl3) δ 8.61 (d, J = 6.2 Hz, 1H), 8.03 (s, 1H), 7.72 (d, J = 7.1 Hz, 1H), 7.50 (d, J = 8.6 Hz, 3H), 7.45 – 7.23 (m, 13H), 7.07 (dd, J = 6.4, 4.2 Hz, 1H), 6.95 (s, 2H), 3.28 (d, J = 13.0 Hz, 1H), 3.09 (d, J = 12.8 Hz, 1H), 2.90 (d, J = 13.1 Hz, 1H), 2.69 (d, J = 13.2 Hz, 1H), 1.70 (s, 3H), 1.64 (s, 3H), 1.57 (s, 3H), 1.32 (s, 3H). **^{13}C NMR** (101 MHz, CDCl3) δ 176.09, 148.25, 148.06, 143.58, 143.46, 142.37, 141.87, 138.51,

135.24, 135.13, 135.05, 134.40, 134.30, 134.03, 133.78, 131.99, 131.95, 131.37, 131.24, 131.04, 130.70, 130.52, 129.07, 129.03, 128.99, 128.76, 128.71, 126.26, 125.64, 123.91, 123.46, 122.95, 122.58, 121.96, 121.47, 121.19, 118.93, 118.85, 117.12, 115.70, 61.58, 61.54, 59.99, 52.96, 52.92, 38.56, 37.96, 30.53, 28.44, 27.96. **^{19}F NMR** (282 MHz, $CDCl_3$) δ -62.86. **^{31}P NMR** (121 MHz, $CDCl_3$) δ 40.21. **IR** (neat) 3333, 3057, 2952, 2923, 1711, 1588, 1527, 1471, 1441, 1381, 1345, 1301, 1275, 1169, 1132, 1090, 1026, 987, 967, 884, 849, 740, 696, 679 cm^{-1}. **HRMS** (ESI) m/z $[M+H]^+$ ($C_{46}H_{39}F_6N_3PS$), calcd.: 810.2506; found: 810.2511.

5.12 Synthesis of (*R*)-diphenyl(1,1,1',1'-tetramethyl-2',4'-dihydro-1H,1'H-3,3'-spiro bi[cyclopenta[b]indol]-4(2H)-yl)phosphine oxide (16)

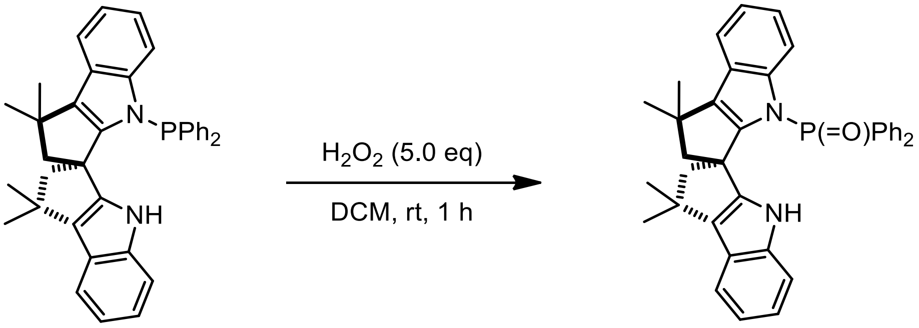

8 **16**

A 2 mL vial was charged with 1 mL of DCM, **8** (0.1 mmol, 1.0 equiv.), and 50 µL of H_2O_2 (30% in water, approximately 0.5 mmol, 5 equiv.). The mixture was stirred for 1 hour at room temperature. The reaction mixture was then directly subjected onto a silica gel column for chromatography (petroleum ether:acetate = 5:1) to afford compound **16** (55.3 mg, 99% yield). Light yellow solid, m.p. 230.7–231.7 °C. **$[\alpha]_D^{25}$** = 113.7 (*c* = 1.0, CH_2Cl_2). **SFC**: ee > 99% (analytical SFC, Trefoil CEL-1 column, MeOH : CO_2 = 10 : 90, flow rate 0.6 mL/min, 2000psi, PDA wavelength: 280 nm): minor isomer: t_R = 5.33 min; major isomer: t_R = 5.88 min. **^{1}H NMR** (300 MHz, $CDCl_3$) δ 7.72 – 7.40 (m, 8H), 7.30 (dd, *J* = 7.1, 5.1 Hz, 4H), 7.17 (t, *J* = 7.5 Hz, 1H), 7.10 – 6.97 (m, 2H), 6.88 (dd, *J* = 10.4, 5.6 Hz, 2H), 6.49 (s, 1H), 6.24 (d, *J* = 8.6 Hz, 1H), 3.71 (d, *J* = 12.3 Hz, 1H), 2.66 (q, *J* = 13.0 Hz, 2H), 2.44 (d, *J* = 12.3 Hz, 1H), 1.68 (s, 3H), 1.61 – 1.46 (m, 9H). **^{13}C NMR** (101 MHz, $CDCl_3$) δ 147.86, 147.83, 144.86, 143.79, 143.75, 140.95, 136.04, 135.99, 132.97, 132.93, 132.90, 132.38, 132.35, 131.91, 131.80, 131.74, 131.55, 131.45, 131.10, 129.85, 128.96, 128.83, 128.65, 128.52, 127.82, 126.27, 126.21, 123.83, 122.14, 121.40, 120.02,

118.86, 118.68, 118.34, 114.79, 111.85, 62.54, 59.58, 52.46, 39.16, 37.85, 30.78, 29.78, 29.67, 28.67. **^{31}P NMR** (121 MHz, $CDCl_3$) δ 23.90. **IR** (neat) 3645, 3192, 2949, 2922, 2858, 1622, 1588, 1481, 1446, 1437, 1357, 1312, 1212, 1189, 1155, 1125, 1105, 1063, 1026, 973, 747, 729, 693 cm^{-1}. **HRMS** (ESI) m/z $[M+Na]^+$ ($C_{37}H_{35}N_2OPNa$), calcd.: 577.2385; found: 577.2380.

5.13 Synthesis of (*R*)-(4'-(diphenylphosphino)-1,1,1',1'-tetramethyl-2',4'-dihydro-1H, 1'H-3,3'-spirobi[cyclopenta[b]indol]-4(2H)-yl)diphenylphosphine oxide (17)

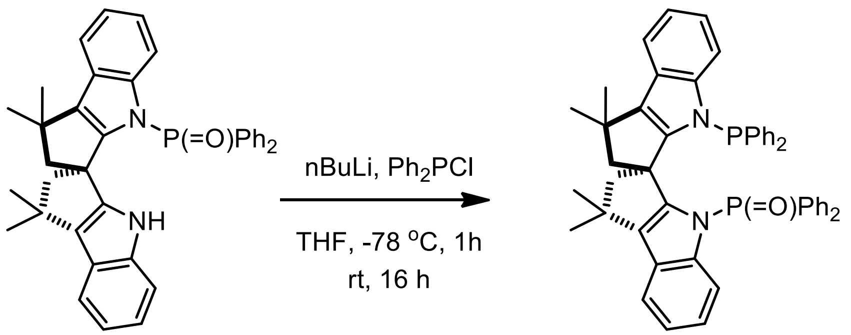

16 **17**

In an oven-dried 5 mL flask under N_2, **16** (0.1 mmol, 1.0 equiv.) and 1 mL of dry THF were added, then the mixture was cooled to 0 °C with an ice bath. nBuLi (1.6 M in hexanes, 69 µL, 0.11 mmol, 1.1 equiv.) was added dropwise. The mixture was stirred at 0 °C for 30 minutes, then Ph_2PCl (24.2 mg, 0.11 mmol, 1.1 equiv.) in 0.5 mL of dry THF was added dropwise at 0 °C. The mixture was warmed to room temperature and stirred for 16 hours, then quenched with 2 mL of saturated NH_4Cl. The reaction mixture was then extracted with CH_2Cl_2 (5 mL × 3). After removal of the solvent by rotary evaporation, the residue was purified by silica gel flash column chromatography (petroleum ether:acetate = 5:1), affording the expected compound **17** as a white solid (62.7 mg, 85% yield). Light yellow solid, m.p. > 300 °C. **$[\alpha]_D^{25}$** = -14.8 (*c* = 1.0, CH_2Cl_2). **SFC**: ee = 100% (ee was determined by the recovered SPINDOLE **4a** from treating **17** with 2.0 equiv. TBAF in THF at room temperature). **^{1}H NMR** (300 MHz, $CDCl_3$) δ 7.79 (dd, *J* = 12.5, 7.9 Hz, 2H), 7.66 – 7.30 (m, 15H), 7.18 – 6.91 (m, 7H), 6.83 (t, *J* = 7.8 Hz, 1H), 6.66 (t, *J* = 7.7 Hz, 1H), 6.55 (d, *J* = 8.3 Hz, 1H), 6.43 (d, *J* = 8.5 Hz, 1H), 2.82 (dd, *J* = 12.9, 4.1 Hz, 2H), 2.58 (d, *J* = 13.3 Hz, 1H), 2.33 (d, *J* = 12.6 Hz, 1H), 1.57 – 1.41 (m, 9H), 1.34 (s, 3H). **^{13}C NMR** (101 MHz, $CDCl_3$) δ 152.20, 151.99, 148.91, 143.55, 143.44, 143.19, 143.14, 136.48, 136.29, 135.64, 135.51, 134.30, 132.96, 132.46, 132.43, 132.30, 132.27, 132.11, 132.01, 131.89, 131.67, 131.46, 130.86, 130.69, 128.84,

128.62, 128.49, 128.41, 128.39, 128.35, 128.26, 127.94, 127.90, 127.86, 127.20, 126.52, 126.46, 121.53, 121.05, 119.99, 119.72, 118.61, 118.42, 115.73, 115.06, 61.97, 60.34, 53.37, 53.32, 38.28, 37.52, 30.31, 30.23, 29.07, 28.77. **^{31}P NMR** (121 MHz, $CDCl_3$) δ 33.80, 25.37. **IR** (neat) 3054, 2951, 2924, 2857, 1607, 1589, 1446, 1435, 1358, 1308, 1281, 1228, 1186, 1156, 1124, 1107, 1076, 1025, 966, 739, 692 cm^{-1}. **HRMS** (ESI) m/z $[M+H]^+$ ($C_{49}H_{45}N_2OP_2$), calcd.: 739.3007; found: 739.3001.

6 Mechanism studies

6.1 General procedure for collection of intermediate 2a and 3a

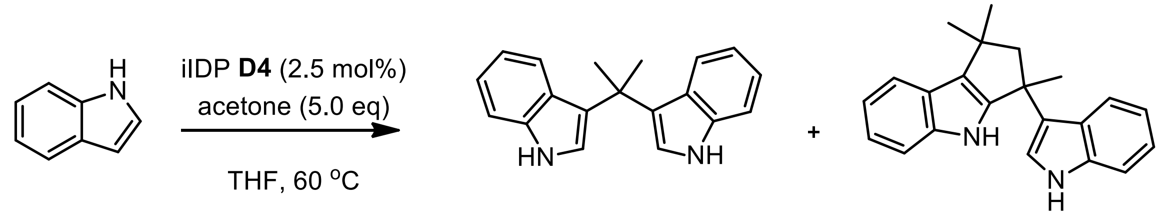

1a **2a** **3a**, 0% ee

A 20 mL heavy-wall vessel was charged with 5 mL of THF, **1** (117 mg, 1 mmol, 1.0 equiv.), acetone (290 mg, 5 mmol, 5.0 equiv.), and iIDP D4 (45 mg, 0.025 mmol, 2.5 mol%) were added. The vessel was sealed and stirred at 60 °C. After the specified time, the solvent of the reaction mixture was removed and subjected onto a silica gel column for chromatography (petroleum ether:acetate = 10:1) to afford compounds **2a** and **3a**, respectively.

3,3'-(propane-2,2-diyl)bis(1H-indole) (2a)

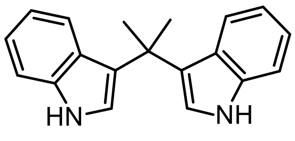

2a

2a was obtained after 2 hours of stirring, yielding 126.1 mg (92% yield). ^{1}H NMR (400 MHz, $CDCl_3$) δ 7.71 (s, 2H), 7.54 (d, J = 8.0 Hz, 2H), 7.34 (d, J = 8.1 Hz, 2H), 7.19 (t, J = 7.5 Hz, 2H), 7.08 – 6.93 (m, 4H), 2.02 (s, 6H). ^{13}C NMR (101 MHz, $CDCl_3$) δ 136.95, 126.19, 125.25, 121.26, 121.16, 120.54, 118.55, 111.06, 34.82, 29.90. The NMR spectroscopic data are in accordance with those reported. [18]

3-(1H-indol-3-yl)-1,1,3-trimethyl-1,2,3,4-tetrahydrocyclopenta[b]indole (3a)

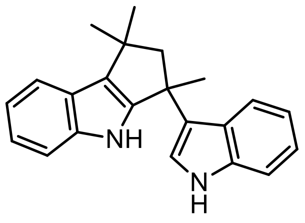

3a

3a was obtained after 20 hours of stirring, yielding 63.8 mg (41% yield). **$[\alpha]_D^{25}$** = 0.0 (c = 0.50, CH_2Cl_2). **SFC**: ee= 0% (analytical SFC, Trefoil AMY-1 column, MeOH : CO_2 = 30 : 70, flow rate 0.6 mL/min, 2000psi, PDA wavelength: 280 nm): major isomer: t_R = 5.01 min; minor isomer: t_R = 5.83 min. **^{1}H NMR** (400 MHz, $CDCl_3$) δ 7.81 (s, 1H), 7.61 (s, 1H), 7.37 (dd, J = 10.9, 7.6 Hz, 2H), 7.32 – 7.19 (m, 4H), 7.15 (d, J = 7.9 Hz, 1H), 7.04 (t, J = 6.2 Hz, 1H), 6.89 (d, J = 2.2 Hz, 1H), 3.08 (d, J = 12.9 Hz, 1H), 2.71 – 2.57 (m, 1H), 1.98 (s, 3H), 1.70 (d, J = 21.5 Hz, 6H). **^{13}C NMR** (101 MHz, $CDCl_3$) δ 147.35, 140.68, 136.79, 125.88, 125.59, 123.66, 123.48, 121.75, 120.71, 120.45, 120.36, 119.27, 118.32, 111.89, 111.18, 62.58, 42.04, 38.98, 30.62, 30.13, 28.46. The NMR spectroscopic data are in accordance with those reported. [19]

6.2 Reactions of intermediates of 2a, 3a under optimal condition

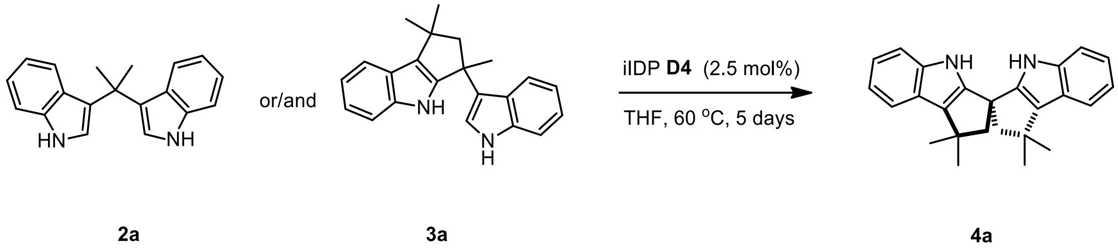

Scheme S3. Synthesis of **2a** using (R, R)-IDPi **D4** from intermediates **I** and **3a**

A 4 mL heavy-wall vessel was charged with 0.5 mL of THF, **2a** or **3a** (0.1 mmol, 1.0 equiv.), with or without acetone (29 mg, 0.5 mmol, 5.0 equiv.), and iIDP **D4** (4.5 mg, 0.0025 mmol, 2.5 mol%) was added. The vessel was sealed and stirred at 60 °C for 5 days. The reaction mixture was then directly subjected onto a silica gel column for chromatography (petroleum ether:acetate = 10:1) to afford compound **4a**.

Table S2: Reaction of intermediates 2a and 3a to afford 4a

Conditions	Yield, ee (%)
**2a**, acetone	88% yield, 96%
**3a**, acetone	89% yield, 96%
**2a**, **3a**, acetone	85% yield, 96%

6.3 Reaction of intermediates of *rac*-3f and 2a under optimal condition

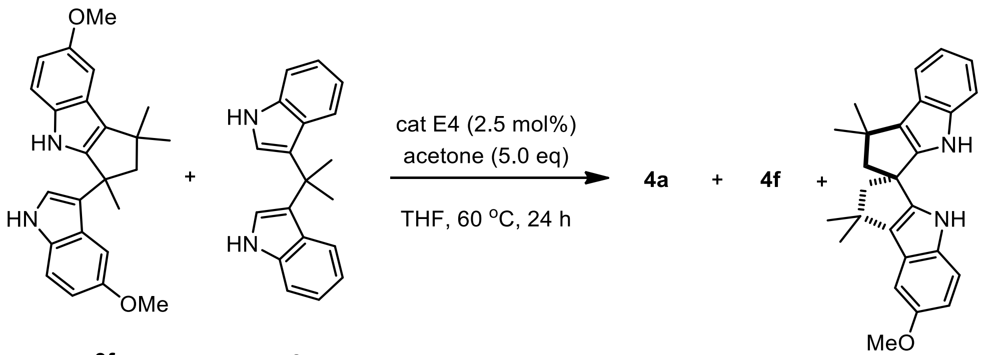

rac-3f **2a** **4cc**

(*R*)-7-methoxy-1,1,1',1'-tetramethyl-2,2',4,4'-tetrahydro-1H,1'H-3,3'-spirobi [cyclopenta[b] indole]

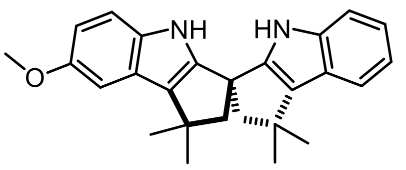

4cc

rac-3f was synthesized as **3a**. A 4 mL heavy-wall vessel was charged with 0.5 mL of THF, **rac-3f** (0.1 mmol, 1.0 equiv.), **3a** (0.1 mmol, 1.0 equiv.), acetone (29 mg, 0.5 mmol, 5.0 equiv.), and iIDP D4 (4.5 mg, 0.0025 mmol, 2.5 mol%) was added. The vessel was sealed and stirred at 60 °C for 5 days. The reaction mixture was then directly subjected onto a silica gel column for chromatography (petroleum ether:acetate = 10:1) to afford compound **4cc** (22.8 mg, 29% yield). Light yellow solid, m.p. 145.2–147.5 °C. $[\alpha]_D^{25}$ = -79.7 (c = 0.55, CH_2Cl_2). **SFC**: ee = 93% (analytical SFC, Trefoil CEL-1 column, IPA : CO_2 = 20 : 80, flow rate 0.6 mL/min, 2000psi, PDA wavelength: 220 nm): major isomer: t_R = 4.71 min; minor isomer: t_R = 7.48 min. ^{1}**H NMR** (400 MHz, CD_2Cl_2) δ 7.72 (s, 1H), 7.59 (dd, J = 8.9, 5.0 Hz, 2H), 7.26 (d,

J = 5.0 Hz, 1H), 7.15 – 7.04 (m, 4H), 6.76 (dd, *J* = 8.8, 2.3 Hz, 1H), 3.87 (s, 3H), 2.83 (d, *J* = 13.1 Hz, 2H), 2.67 (dd, *J* = 13.1, 3.8 Hz, 2H), 1.63 (s, 6H), 1.54 (s, 6H). **^{13}C NMR** (101 MHz, CD_2Cl_2) δ 153.57, 145.07, 144.00, 140.80, 135.86, 127.17, 126.98, 123.47, 123.11, 120.60, 119.03, 117.91, 111.81, 111.33, 109.92, 100.61, 61.69, 61.63, 55.39, 48.91, 38.74, 38.63, 29.67, 29.63, 29.52, 29.49. **IR** (neat) 3393, 3326, 2950, 2918, 2863, 1720, 1622, 1582, 1452, 1360, 1291, 1258, 1207, 1156, 1104, 1026, 832, 795, 745 cm^{-1}. **HRMS** (ESI) m/z [M+H]$^+$ ($C_{26}H_{29}N_2O$), calcd.: 385.2280; found: 385.2270.

6.4 Stacked ^{1}H NMR spectra of the process with toluene-D8 as solvent and plot of yields of 2a, 3a and 4a over time

1a (11.8 mg, 0.1 mmol, 1.0 eq) in 0.3 mL toluene-D8; iIDP **D4** (4.5 mg, 2.5 mol%), acetone 2 (29.0 mg, 0.5 mmol, 5.0 eq) and 1,3,5-Trimethylbenzene (1.05 mg, 6.25x10^{-3} mmol) in 0.2 mL toluene-D8 were mixed in the J-Young NMR tubes. Then the sample was placed inside an NMR machine, which was preheated to 60 ^{o}C and measurements were taken every 1h for the next 72 h.

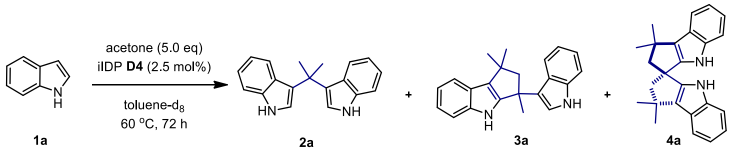

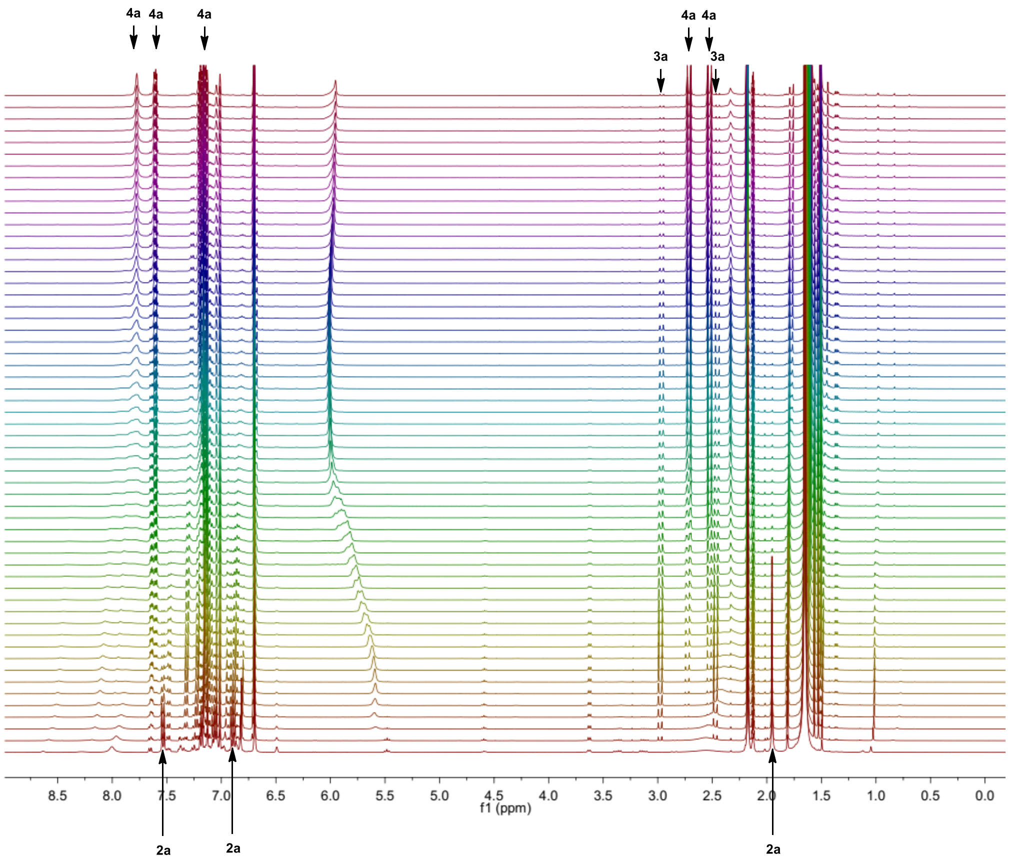

Figure S6: Stacked ^{1}H NMR spectra of the process with toluene-D8 as solvent

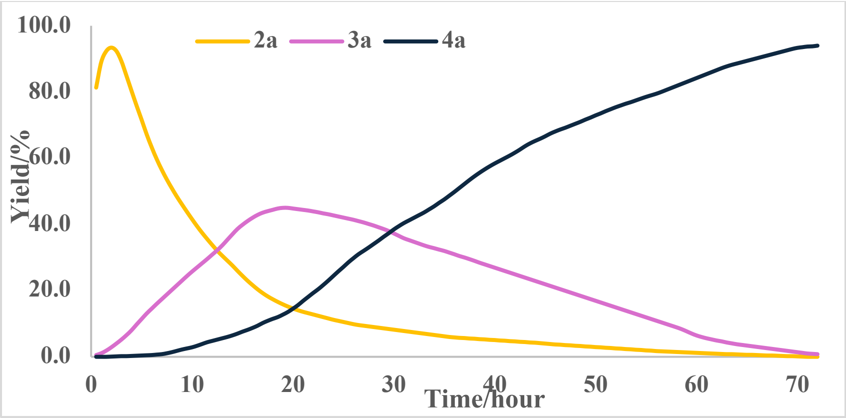

Figure S7: Plot of yields of 2a, 3a, and 4a over time

6.5 KIE study

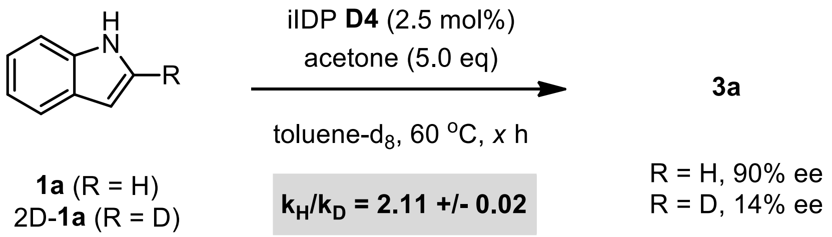

1a or 2D-**1a** (11.8 mg, 0.1 mmol, 1.0 eq) in 0.3 mL toluene-d_8; iIDP **D4** (4.5 mg, 2.5 mol%), acetone 2 (29.0 mg, 0.5 mmol, 5.0 eq) and 1,3,5-Trimethylbenzene (1.05 mg, 6.25x10^{-3} mmol) in 0.2 mL toluene-D8 were mixed in the NMR tubes and heated at 60 °C. Then the sample was placed inside an NMR machine, which was preheated to 60 °C and measurements were taken every 1h for the next 72 h. The signal s for desired products **3a** started appearing after 3 hours stirring. Each reaction was repeated three times.

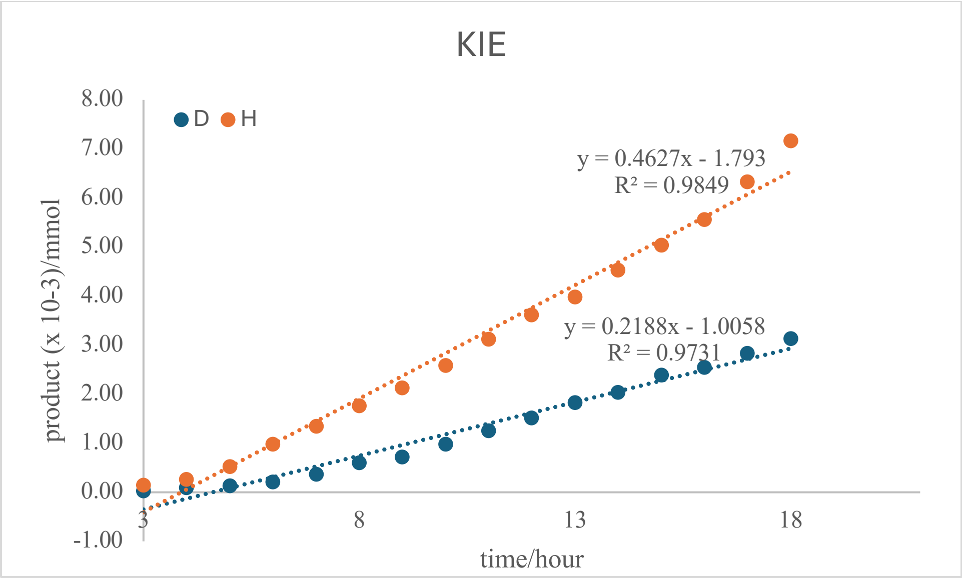

Figure S8: KIE study

6.6 Exploring the reaction with (R)-SPINDOLE as catalyst

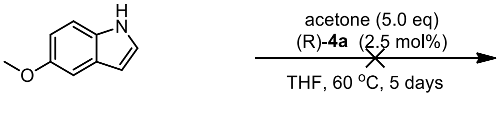

1f

A 4 mL reaction vessel was charged with 1.0 mL of THF, **1f** (0.2 mmol, 1.0 equiv), acetone (58.0 mg, 1.0 mmol, 5.0 equiv), and (R)-**4a** (1.8 mg, 0.005 mmol, 2.5 mol%). The vessel was sealed and the reaction mixture was stirred at 60 °C for 5 days, monitored by TLC. No reaction was observed, and both (R)-**4a** and the starting material **1f** were recovered unchanged.

6.7 Nonlinear effect studies

The specified ee values of iIDP **D4** were made by combining the certain amounts of optically pure(*R,R*)- iIDP **D4** with optically pure (*S,S*)- iIDP **D4**. Six reactions containing iIDP **D4** of racemic, 20%, 40%, 60%,80%, and 100% ee optical purity were run in parallel.

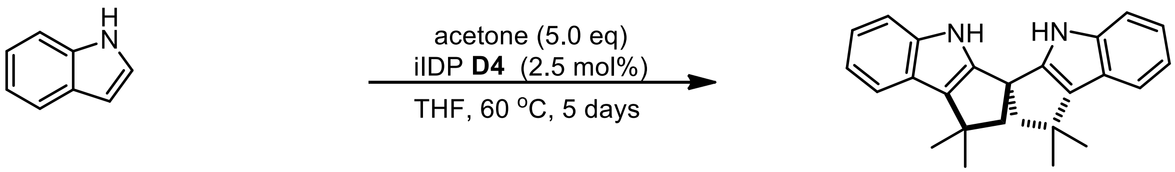

1a **4a**

Entry	ee of iIDP **D4** (%)	ee of **4a** (%)
1	0	0
2	20	18
3	40	37
4	60	59
5	80	77
6	100	96

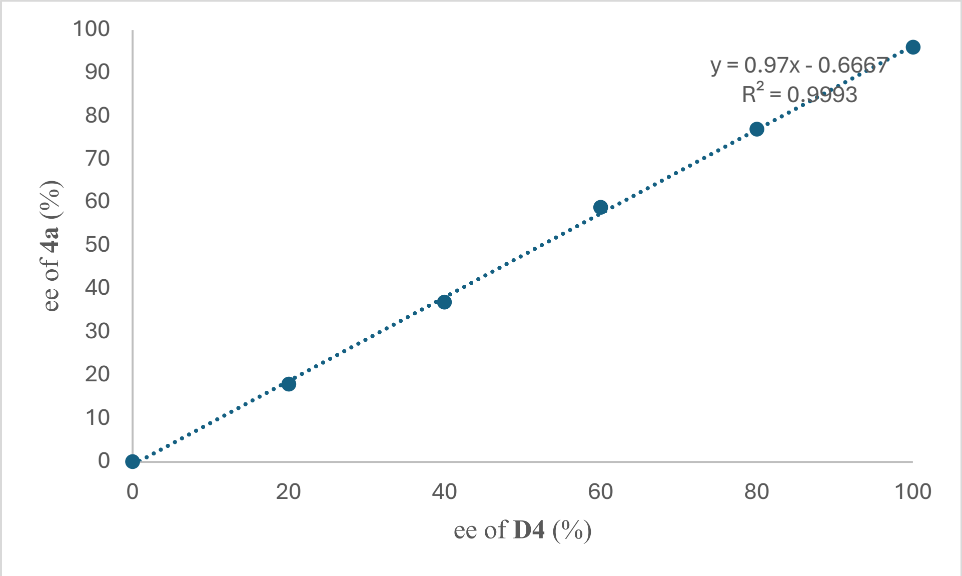

Figure S9: Nonlinear effect studies

7 Application of SPINDOLE-derived catalysts and ligands

7.1 Comparison of structure properties of Pd(II) complexes (full information)

Table S3: Mapping molecular properties of bisphosphine ligands

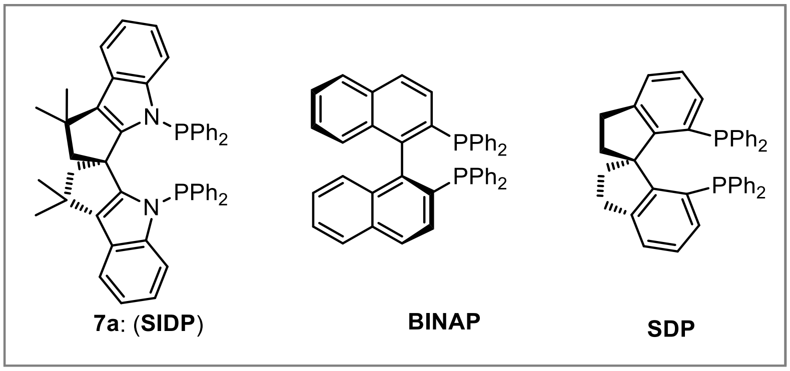

$PdCl_2$[**L**]	Natural charge (Pd^{II})	P-P distance (Å)	P-Pd distance (Å)	T_4	Bite angle (°)	Dihedral angle (°)	^{31}P NMR (L, ppm)*
$PdCl_2$[**BINAP**]	-0.191	3.25	2.25	0.215	92.7	68.4	-14.76
$PdCl_2$[SDP]	-0.181	3.36	2.26	0.116	96.0	61.4	-17.6
$PdCl_2$[SIDP] (**13**)	-0.175	3.49	2.27	0.107	100.4	68.1	35.25

* ^{31}P NMR based on the ligands.

7.2 General procedure for asymmetric Hydroboration/cyclization of 1,6-enynes synthesis of (*S,E*)-3-methyl-4-((4,4,5,5-tetramethyl-1,3,2-dioxaborolan-2-yl)methylene)-1-tosylpyrrolidine (19)

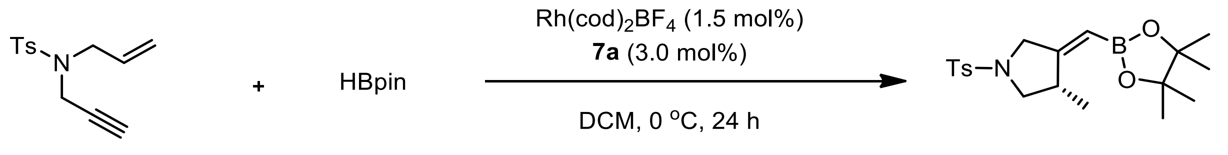

A mixture of [Rh(cod)$_2$]BF$_4$ (1.2 mg, 0.003 mmol, 1.5 mol%) and (R)-**7a** (4.3 mg, 0.006 mmol, 3.0 mol%) in DCM (1 mL) was stirred under nitrogen at 25 °C for 30 min. HBpin (38.4 mg, 0.3 mmol) was added, and the mixture was stirred at 25 °C for another 20 min. 1,6-enyne **18** (50.0 mg, 0.2 mmol) in 1.0 mL of DCM was then added and stirred at 0 °C for 24 h. The reaction mixture was concentrated under reduced pressure. The crude product was purified by silica gel column chromatography using petroleum ether/ethyl acetate (5:1) as the eluent to give **19** (colorless oil, 71.6 mg, 95% yield). **$[\alpha]_D^{25}$** = -43.9 (*c* = 1.0, $CHCl_3$). **SFC**: ee = 90% (analytical SFC, Trefoil AMY-1 column, IPA : CO_2 = 20 : 80, flow rate 0.6 mL/min, 2000psi, PDA wavelength: 220 nm): minor isomer: t_R = 2.77 min; major isomer: t_R = 3.41 min. **^{1}H NMR** (400 MHz, $CDCl_3$) δ 7.67 (d, *J* = 8.1 Hz, 2H), 7.30 (d, *J* = 8.0 Hz, 2H), 5.16 (s, 1H), 4.08 (d, *J* = 15.4 Hz, 1H), 3.55 (d, *J* = 15.4 Hz, 1H), 3.22 (dd, *J* = 13.0, 7.9 Hz, 2H), 3.07 (dd, *J* = 9.0, 6.4 Hz, 1H), 2.40 (s, 3H), 1.20 (s, 12H), 1.11 (d, *J* = 7.0 Hz, 3H). **^{13}C NMR** (101 MHz, $CDCl_3$) δ 165.34, 143.46, 132.12, 129.47, 127.72, 82.90, 77.32, 77.00, 76.68, 55.20, 53.33, 37.61, 24.75, 24.46, 21.35, 20.94. The data is coincided with the previous literature data.[20]

7.3 General procedure for Rh-Catalyzed Asymmetric Hydrogenation to afford (*S*)-methyl 2-acetamido-3-phenylpropanoate (21)

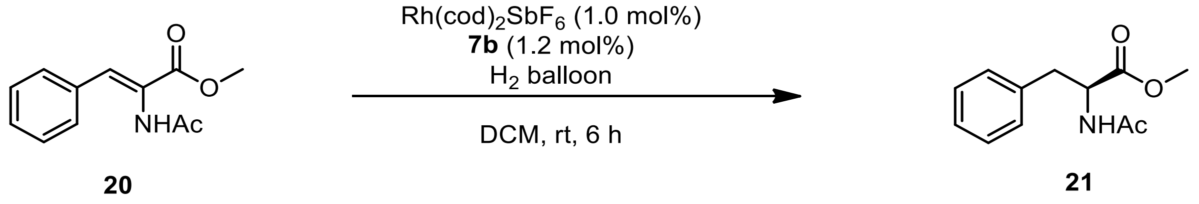

In a nitrogen-filled glove box, **Rh(cod)$_2$SbF$_6$** (1.1 mg, 0.002 mmol, 1.0 mol%), **R-7b** (2.0 mg, 0.0024 mmol, 1.2 mol%), and substrate **20** (43.8 mg, 0.2 mmol, 1.0 equiv.) were charged into a 10 mL Schlenk tube. The tube was sealed and moved outside of the glove box, followed by the addition of anhydrous DCM (2.0 mL). After stirring for 30 min at room temperature, the reaction system was flushed with H_2 three times and then stirred under an H_2 balloon for 6 hours at room temperature. The solution was then concentrated under reduced pressure, and the reaction mixture was purified by silica gel column chromatography (PE: EtOAc = 3:1 to 1:1), affording the expected compound **21** (44.0 mg, 99% yield) as a light yellow liquid. $[\alpha]_D^{25}$ = 108.5 (*c* = 1.0, $CHCl_3$). **SFC**: ee = 99% (analytical SFC, Trefoil AMY-1 column, IPA : CO_2 = 10 : 90, flow rate 0.6 mL/min, 2000psi, PDA wavelength: 220 nm): minor isomer: t_R = 2.12 min; major isomer: t_R = 2.88 min. **^{1}H NMR** (400 MHz, $CDCl_3$) δ 7.28 (ddd, *J* = 8.2, 7.6, 6.1 Hz, 3H), 7.14 – 7.05 (m, 2H), 5.96 (s, 1H), 4.89 (dt, *J* = 7.8, 5.8 Hz, 1H), 3.73 (s, 3H), 3.12 (qd, *J* = 13.9, 5.8 Hz, 2H), 1.98 (s, 3H). **^{13}C NMR** (101 MHz, $CDCl_3$) δ 172.07, 169.55, 135.80, 129.19, 128.54, 127.09, 53.08, 52.27, 37.81, 23.09. The data is coincided with the previous literature data. [21]

7.4 General procedure for Pd-catalyzed asymmetric allylic alkylation in the presence of Pd[SIDP]Cl_2 ligand to afford (*R*,*E*)-dimethyl 2-(1,3-diphenylallyl)malonate (23)

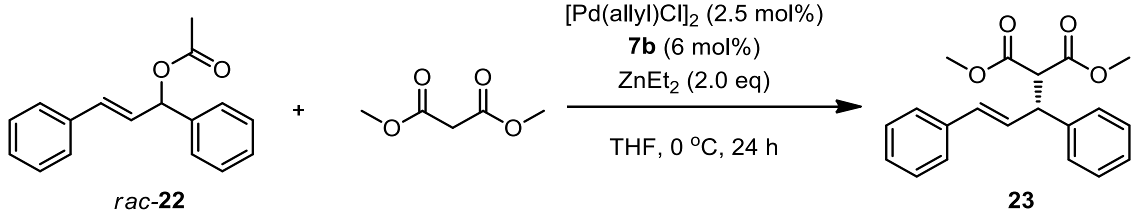

A 4.0 mL vial was charged with **SIDP 7b** (5.0 mg, 0.006 mmol, 6 mol%), **[Pd(allyl)Cl]$_2$** (0.9 mg, 0.0025 mmol, 2.5 mol%), and 0.5 mL of THF under N_2, followed by the injection of a THF solution (0.5 mL) of rac-**22** (28.2 mg, 0.1 mmol, 1.0 equiv). After stirring for another 10 min, a THF solution (1.0 mL) of dimethyl malonate (26.4 mg, 0.2 mmol, 2.0 equiv) was added, and the reaction mixture was cooled to 0 °C. Then, $ZnEt_2$ (1.0 M in hexane, 0.2 mL, 0.2 mmol, 2.0 equiv) was added dropwise. The reaction mixture was then stirred at 0 °C for 48 h. Upon completion, the reaction mixture was diluted with EtOAc (10 mL), washed with saturated NH_4Cl (10 mL) and brine (10 mL). The solvent was then removed, and the reaction mixture was purified by silica gel column chromatography (PE: EtOAc = 20:1 to 10:1), affording the expected compound **23** as a white solid (22.7 mg, 70% yield). Light yellow liquid. **$[\alpha]_D^{25}$** = 20.7 (*c* = 1.0, $CHCl_3$). **SFC**: ee = 91% (analytical SFC, Trefoil AMY-1 column, IPA : CO_2 = 20 : 80, flow rate 0.6 mL/min, 2000psi, PDA wavelength: 220 nm): major isomer: t_R = 1.78 min; minor isomer: t_R = 1.99 min. **^{1}H NMR** (400 MHz, $CDCl_3$) δ 7.33 – 7.09 (m, 10H), 6.41 (d, *J* = 15.8 Hz, 1H), 6.26 (dd, *J* = 15.7, 8.6 Hz, 1H), 4.19 (dd, *J* = 10.7, 8.7 Hz, 1H), 3.88 (d, *J* = 10.9 Hz, 1H), 3.63 (s, 3H), 3.44 (s, 3H). **^{13}C NMR** (101 MHz, $CDCl_3$) δ 168.18, 167.76, 140.12, 136.78, 131.80, 129.06, 128.70, 128.45, 127.84, 127.55, 127.15, 126.36, 57.61, 52.63, 52.45, 49.17. The data is coincided with the previous literature data.[22]

7.5 General procedure for 15a-Catalyzed asymmetric Michael addition of cinnaldehyde and N-Me indole to generate (*S*)-3-(1-methyl-1H-indol-3-yl)-3-phenylpropan-1-ol (26)

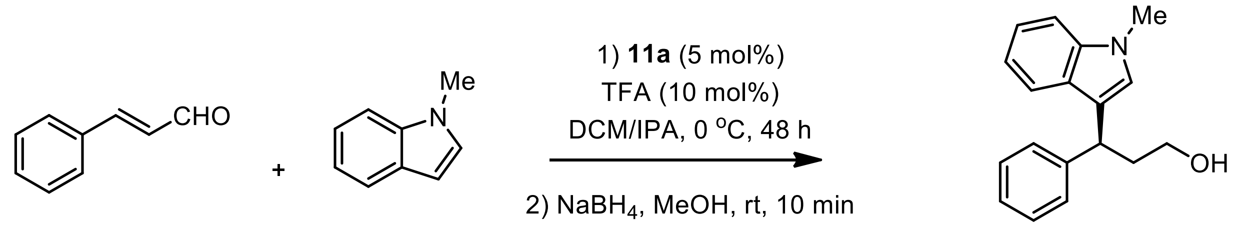

Prepare a mixture of substituted spindoline **11a** (1.8 mg, 0.005 mmol, 5 mol%), **TFA** (1.14 mg, 0.01 mmol, 10 mol%), and **24** (13.2 mg, 0.1 mmol) in a mixture of DCM (1.6 mL) and i-PrOH (0.4 mL), and stir for 15 min at room temperature. Then, add 1-methylindole **25** (26.2 mg, 0.2 mmol). Stir the reaction mixture at 0 °C for 48 h (monitor by TLC). Afterward, add 2 mL of EtOH, an excess of $NaBH_4$ (19 mg, 0.5 mmol), and stir the resulting solution for 10 min. Quench the reaction mixture with saturated aqueous $NaHCO_3$, extract with DCM, and dry over anhydrous Na_2SO_4. Evaporate the solvent under vacuum and purify the residue by flash column chromatography over silica gel (AcOEt/petroleum ether 1:10) to obtain **26** (20.9 mg, 79% yield) as a light yellow liquid. $[\alpha]_D^{25}$ = 29.1 (*c* = 1.0, $CHCl_3$). **SFC**: ee = 91% (analytical SFC, Trefoil CEL-1 column, MeOH : CO_2 = 20 : 80, flow rate 0.6 mL/min, 2000psi, PDA wavelength: 220 nm): minor isomer: t_R = 4.40 min; major isomer: t_R = 6.89 min. **^{1}H NMR** (400 MHz, $CDCl_3$) δ 7.52 (d, *J* = 8.0 Hz, 1H), 7.43 – 7.35 (m, 2H), 7.35 – 7.26 (m, 3H), 7.22 (ddd, *J* = 13.5, 6.2, 1.0 Hz, 2H), 7.12 – 7.02 (m, 1H), 6.93 (s, 1H), 4.42 (t, *J* = 7.7 Hz, 1H), 3.77 (s, 3H), 3.69 (td, *J* = 6.4, 3.3 Hz, 2H), 2.49 (dt, *J* = 13.6, 6.8 Hz, 1H), 2.32 (ddd, *J* = 13.6, 8.3, 4.2 Hz, 1H). **^{13}C NMR** (101 MHz, $CDCl_3$) δ 144.89, 137.18, 128.37, 127.78, 127.24, 126.05, 125.87, 121.56, 119.50, 118.71, 118.13, 109.10, 77.32, 77.00, 76.68, 61.30, 39.12, 38.77, 32.62. The data is coincided with the previous literature data.[23]

8 Unreactive indoles

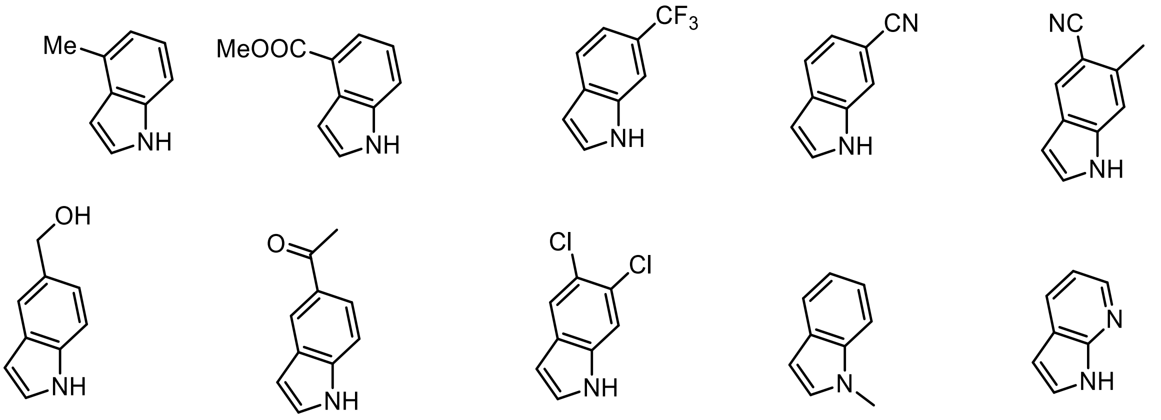

Figure S10: Unreactive indoles and analogs

9 Characterization data

(*R*)-1,1,1',1'-tetramethyl-2,2',4,4'-tetrahydro-1H,1'H-3,3'-spirobi [cyclopenta[b]indole]

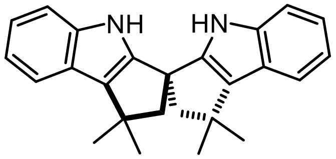

4a

Light yellow solid, m. p. 249.5-250.4 °C. 30.4 mg, 86% yield. **[α]$_D^{25}$** = -34.3 (*c* = 1.0, CH_2Cl_2). **SFC**: ee = 96% (analytical SFC, Trefoil AMY-1 column, MeOH : CO_2 = 10 : 90, flow rate 0.6 mL/min, 2000psi, PDA wavelength: 280 nm): major isomer: t_R = 4.04 min; minor isomer: t_R = 5.88 min. **^{1}H NMR** (400 MHz, $CDCl_3$) δ 7.64 – 7.57 (m, 2H), 7.53 (s, 2H), 7.25 (dd, *J* = 6.2, 2.9 Hz, 2H), 7.18 – 7.09 (m, 4H), 2.81 (d, *J* = 13.1 Hz, 2H), 2.65 (d, *J* = 13.1 Hz, 2H), 1.62 (s, 6H), 1.53 (s, 6H). **^{13}C NMR** (101 MHz, $CDCl_3$) δ 144.31, 141.05, 127.70, 123.47, 121.21, 119.63, 118.47, 111.81, 62.15, 49.28, 39.26, 30.35, 30.32. **IR** (neat) 3453, 3065, 2975, 2946,2922,2869,1578, 1446, 1369, 1312, 1285, 1250, 1220, 1177, 1149, 1103, 1067, 1009, 963, 923, 749, 731 cm^{-1} **HRMS** (ESI) m/z [M+H]$^+$ ($C_{25}H_{27}N_2$), calcd.: 355.2174; found: 355.2178.

(*R*)-7,7'-difluoro-1,1,1',1'-tetramethyl-2,2',4,4'-tetrahydro-1H,1'H-3,3'-spirobi[cyclopenta [b]indole]

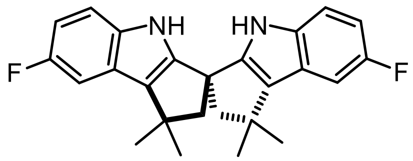

4b

Light yellow solid, m. p. 187.8-189.7 °C. 30.0 mg, 77% yield. **[α]$_D^{25}$** = -34.6 (*c* = 0.5, CH_2Cl_2). **SFC**: ee = 95% (analytical SFC, Trefoil AMY-1 column, IPA : CO_2 = 15 : 85, flow rate 0.6 mL/min, 2000psi, PDA wavelength: 280 nm): major isomer: t_R = 1.25 min; minor isomer: t_R = 1.73 min. **^{1}H NMR** (400 MHz, CD_2Cl_2) δ 7.72 (s, 2H), 7.18 (dt, *J* = 6.0, 3.3 Hz, 4H), 6.84 (td, *J* = 9.2, 2.5 Hz, 2H), 2.77 (d, *J* = 13.2 Hz, 2H), 2.62 (d, *J* = 13.2 Hz, 2H), 1.55 (s, 6H), 1.47 (s, 6H). **^{13}C NMR** (101 MHz, CD_2Cl_2) δ 158.91, 156.59, 146.40, 137.77, 128.01, 127.96, 123.74, 123.64, 112.36, 112.27, 109.15, 108.89, 103.57, 103.34, 62.00, 49.46, 39.14, 29.97, 29.92. **^{19}F NMR** (282 MHz, CD_2Cl_2) δ -125.10, -125.12, -125.13, -125.15, -125.17, -125.18. **IR** (neat) 3431, 2957, 2923, 2863, 1579, 1481, 1448, 1362, 1287, 1249, 1218, 1193, 1157, 1142, 1106, 1048, 1034, 979, 957, 926, 860, 850, 795, 733 cm^{-1} **HRMS** (ESI) m/z [M+H]$^+$ ($C_{25}H_{25}F_2N_2$), calcd.: 391.1986; found: 391.1979.

(*R*)-7,7'-dichloro-1,1,1',1'-tetramethyl-2,2',4,4'-tetrahydro-1H,1'H-3,3'-spirobi[cyclopenta[b]indole]

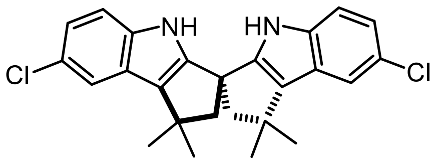

4c

Light yellow solid, m. p. 267.0-268.9 °C. 30.0 mg, 72% yield. **[α]$_D$^{25}** = -25.2 (*c* = 0.5, CH_2Cl_2). **SFC**: ee = 90% (analytical SFC, Trefoil AMY-1 column, IPA : CO_2 = 30 : 70, flow rate 0.6 mL/min, 2000psi, PDA wavelength: 280 nm): major isomer: t_R = 2.28 min; minor isomer: t_R = 4.20 min. **^{1}H NMR** (400 MHz, CD_2Cl_2) δ 7.77 (s, 2H), 7.51 (d, *J* = 1.8 Hz, 2H), 7.19 (d, *J* = 8.6 Hz, 2H), 7.04 (dd, *J* = 8.6, 2.0 Hz, 2H), 2.77 (d, *J* = 13.2 Hz, 2H), 2.62 (d, *J* = 13.2 Hz, 2H), 1.55 (s, 6H), 1.47 (s, 6H). **^{13}C NMR** (101 MHz, CD_2Cl_2) δ 145.85, 139.60, 127.63, 125.03, 124.46, 121.24, 117.94, 112.81, 61.94, 49.39, 39.17, 30.01, 29.96. **IR** (neat) 3431, 2951, 2922, 2860, 1574, 1450, 1436, 1380, 1362, 1338, 1286, 1220, 1186, 1124, 1135, 1108, 1064, 1009, 989, 914, 861, 799, 727 cm^{-1} **HRMS** (ESI) m/z [M+H]$^+$ ($C_{25}H_{25}Cl_2N_2$), calcd.:423.1395; found:423.1396.

(*R*)-7,7'-dibromo-1,1,1',1'-tetramethyl-2,2',4,4'-tetrahydro-1H,1'H-3,3'-spirobi[cyclopenta[b]indole]

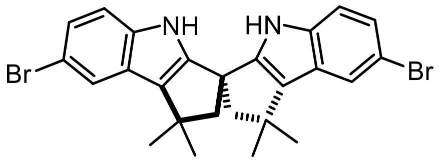

4d

Light yellow solid, m. p. 232.2-234.0 °C. 34.8 mg, 68% yield. **[α]$_D$^{25}** = -81.1 (*c* = 0.5, CH_2Cl_2). **SFC**: ee = 77% (analytical SFC, Trefoil AMY-1 column, IPA : CO_2 = 15 : 85, flow rate 0.6 mL/min, 2000psi, PDA wavelength: 300 nm): major isomer: t_R = 5.55 min; minor isomer: t_R = 11.28 min. **^{1}H NMR** (400 MHz, CD_2Cl_2) δ 7.76 (s, 2H), 7.70 – 7.62 (m, 2H), 7.23 – 7.11 (m, 4H), 2.76 (d, J = 13.2 Hz, 2H), 2.62 (d, J = 13.2 Hz, 2H), 1.54 (s, 6H), 1.46 (s, 6H). **^{13}C NMR** (101 MHz, CD_2Cl_2) δ 145.66, 139.87, 127.54, 125.12, 123.84, 121.02, 113.28, 112.64, 61.93, 49.34, 39.19, 30.03, 29.97. **IR** (neat) 3431, 2949, 2920, 2858, 1577, 1450, 1434, 1379, 1361, 1337,1286, 1219, 1185, 1135, 1108, 1066, 1052, 1008, 988, 910, 870, 860, 797, 731, 716, 702 cm^{-1} **HRMS** (ESI) m/z [M+H]$^+$ ($C_{25}H_{25}Br_2N_2$), calcd.: 511.0384; found: 511.0382.

(*R*)-7,7'-diiodo-1,1,1',1'-tetramethyl-2,2',4,4'-tetrahydro-1H,1'H-3,3'-spirobi[cyclopenta [b]indole]

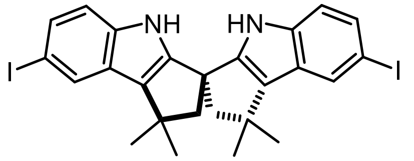

4e

Yellow solid, m. p. 155.6-156.9 °C. 42.4 mg, 75% yield. **$[\alpha]_D^{25}$** = -70.1 (*c* = 0.52, CH_2Cl_2). **SFC**: ee = 80% (analytical SFC, Trefoil AMY-1 column, MeOH : CO_2 = 20 : 80, flow rate 0.6 mL/min, 2000psi, PDA wavelength: 280 nm): major isomer: t_R = 16.29 min; minor isomer: t_R = 28.14 min. **^{1}H NMR** (400 MHz, CD_2Cl_2) δ 7.89 (s, 2H), 7.78 (s, 2H), 7.36 (dd, *J* = 8.5, 1.5 Hz, 2H), 7.07 (d, *J* = 8.5 Hz, 2H), 2.77 (d, *J* = 13.2 Hz, 2H), 2.63 (d, *J* = 13.2 Hz, 2H), 1.55 (s, 6H), 1.47 (s, 6H). **^{13}C NMR** (101 MHz, CD_2Cl_2) δ 145.18, 140.33, 129.45, 127.31, 127.26, 125.97, 113.84, 82.90, 61.93, 49.23, 39.21, 30.04, 30.00. **IR** (neat) 3389, 2952, 2923, 2861, 1577, 1450, 1362, 1286, 1245, 1204, 1188, 1169, 1071,944, 862, 809, 791, 724 cm^{-1} **HRMS** (ESI) m/z [M+H]$^+$ ($C_{25}H_{25}I_2N_2$), calcd.: 607.0107; found: 607.0100.

(*R*)-7,7'-dimethoxy-1,1,1',1'-tetramethyl-2,2',4,4'-tetrahydro-1H,1'H-3,3'-spirobi [cyclopenta [b] indole]

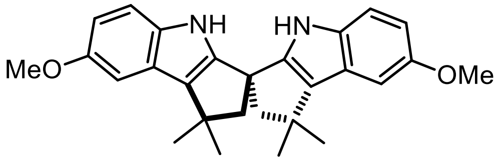

4f

Yellow solid, m. p. 138.9-140.6 °C. 25.9 mg, 63% yield. **$[\alpha]_D^{25}$** = -85.1 (*c* = 1.0, CH_2Cl_2). **SFC**: ee = 90% (analytical SFC, Trefoil CEL-1 column, MeOH : CO_2 = 20 : 80, flow rate 0.6 mL/min, 2000psi, PDA wavelength: 280 nm): major isomer: t_R = 6.25 min; minor isomer: t_R = 11.59 min. **^{1}H NMR** (400 MHz, CD_2Cl_2) δ 7.60 (s, 2H), 7.10 (d, *J* = 8.8 Hz, 2H), 7.01 (d, *J* = 2.4 Hz, 2H), 6.71 (dd, *J* = 8.8, 2.5 Hz, 2H), 3.82 (s, 6H), 2.77 (d, *J* = 13.1 Hz, 2H), 2.61 (d, *J* = 13.1 Hz, 2H), 1.57 (s, 6H), 1.49 (s, 6H). **^{13}C NMR** (101 MHz, CD_2Cl_2) δ 154.05, 145.56, 136.35, 127.43, 123.95, 112.30, 110.39, 101.08, 62.15, 55.88, 49.38, 39.11, 30.00, 29.98. **IR** (neat) 3388, 2953, 2861, 1705, 1651, 1583, 1487, 1455, 1439, 1385, 1362, 1293, 1257, 1207, 1178, 1158, 1079, 1031, 987, 852, 797, 732, cm^{-1} **HRMS** (ESI) m/z [M+H]$^+$ ($C_{27}H_{31}N_2O_2$), calcd.: 415.2386; found: 415.2388.

(*R*)-7,7'-diethoxy-1,1,1',1'-tetramethyl-2,2',4,4'-tetrahydro-1H,1'H-3,3'-spirobi[cyclopenta [b]indole]

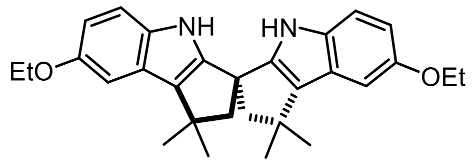

4g

Yellow solid, m. p. 140.0-141.5 °C. 33.2 mg, 75% yield. **[α]$_D$^{25}** = -81.8 (*c* = 0.5, CH_2Cl_2). **SFC**: ee = 91% (analytical SFC, Trefoil AMY-1 column, IPA : CO_2 = 20 : 80, flow rate 0.6 mL/min, 2000psi, PDA wavelength: 280 nm): major isomer: t_R = 2.10 min; minor isomer: t_R = 2.96 min. **^{1}H NMR** (400 MHz, CD_2Cl_2) δ 7.56 (s, 2H), 7.10 (d, *J* = 8.8 Hz, 2H), 7.00 (d, *J* = 2.3 Hz, 2H), 6.71 (dd, *J* = 8.8, 2.5 Hz, 2H), 4.05 (q, *J* = 7.0 Hz, 4H), 2.76 (d, *J* = 13.1 Hz, 2H), 2.60 (d, *J* = 13.1 Hz, 2H), 1.56 (s, 6H), 1.48 (s, 6H), 1.40 (t, *J* = 7.0 Hz, 6H). **^{13}C NMR** (101 MHz, CD_2Cl_2) δ 153.28, 145.53, 136.38, 127.40, 123.99, 112.23, 110.94, 102.29, 64.32, 62.15, 49.38, 39.10, 30.00, 29.99, 14.97. **IR** (neat) 3400, 2953, 2922, 2861, 1704, 1622, 1584, 1452, 1392, 1360, 1289, 1199, 1164, 1111, 1044, 989, 933, 853, 795 cm^{-1} **HRMS** (ESI) m/z [M-H]$^-$ ($C_{29}H_{33}N_2O_2$), calcd.: 441.2542; found: 441.2552.

(*R*)-7,7'-bis(benzyloxy)-1,1,1',1'-tetramethyl-2,2',4,4'-tetrahydro-1H,1'H-3,3'-spirobi [cyclopenta[b]indole]

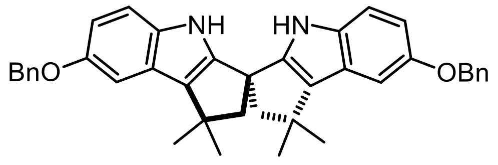

4h

Yellow solid, m. p. 105.5-107.6 °C. 45.9 mg, 81% yield. **[α]$_D$^{25}** = -40.2 (*c* = 1.0, CH_2Cl_2). **SFC**: ee = 96% (analytical SFC, Trefoil AMY-1 column, MeOH : CO_2 = 30 : 70, flow rate 0.6 mL/min, 2000psi, PDA wavelength: 280 nm): minor isomer: t_R = 5.04 min; major isomer: t_R = 9.49 min. **^{1}H NMR** (400 MHz, CD_2Cl_2) δ 7.56 (s, 2H), 7.48 (d, *J* = 7.2 Hz, 4H), 7.39 (t, *J* = 7.4 Hz, 4H), 7.33 (d, *J* = 7.1 Hz, 2H), 7.12 (dd, *J* = 12.6, 5.4 Hz, 4H), 6.80 (dd, *J* = 8.8, 2.4 Hz, 2H), 5.09 (s, 4H), 2.76 (d, *J* = 13.1 Hz, 2H), 2.60 (d, *J* = 13.1 Hz, 2H), 1.56 (s, 6H), 1.48 (s, 6H). **^{13}C NMR** (101 MHz, CD_2Cl_2) δ 153.11, 145.62, 137.98, 136.56, 128.49, 127.80, 127.71, 127.47, 123.93, 112.28, 111.11, 102.87, 70.99, 62.12, 49.40, 39.09, 30.00, 29.98. **IR** (neat) 3416, 3032, 2951, 2840, 1698, 1622, 1582, 1450, 1380, 1360, 1288, 1198, 1163, 1100, 1080, 1023, 951, 924, 846, 825, 795, 733, 695 cm^{-1} **HRMS** (ESI) m/z [M+H]$^+$ ($C_{39}H_{39}N_2O_2$), calcd.: 567.3012; found: 567.3007.

(*R*)-1,1,1',1'-tetramethyl-7,7'-diphenyl-2,2',4,4'-tetrahydro-1H,1'H-3,3'-spirobi[cyclopenta [b]indole]

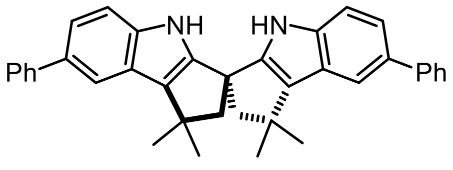

4i

Light yellow solid, m. p. 165.0-166.7 °C. 45.7 mg, 90% yield. **[α]$_D^{25}$** = -90.3 (*c* = 1.0, CH_2Cl_2). **SFC**: ee = 95% (analytical SFC, Trefoil AMY-1 column, IPA : CO_2 = 30 : 70, flow rate 0.6 mL/min, 2000psi, PDA wavelength: 280 nm): major isomer: t_R = 3.15 min; minor isomer: t_R = 7.41 min. **^{1}H NMR** (400 MHz, $CDCl_3$) δ 7.79 (s, 2H), 7.69 (d, *J* = 7.3 Hz, 4H), 7.62 (s, 2H), 7.49 (t, *J* = 7.6 Hz, 4H), 7.39 (ddd, *J* = 18.3, 9.6, 5.1 Hz, 6H), 2.85 (d, *J* = 13.1 Hz, 2H), 2.69 (d, *J* = 13.1 Hz, 2H), 1.66 (s, 6H), 1.58 (s, 6H). **^{13}C NMR** (101 MHz, $CDCl_3$) δ 145.02, 142.65, 140.57, 133.46, 128.64, 128.05, 127.44, 126.37, 123.93, 121.11, 117.10, 112.00, 62.14, 49.37, 39.31, 30.38. **IR** (neat) 3403, 3034, 2954, 2924, 2863, 1716, 1622, 1600, 1478, 1456, 1387, 1361, 1292, 1265, 1166, 1127, 1076, 1045, 980, 895, 875, 820, 763, 749, 696 cm^{-1} **HRMS** (ESI) m/z [M+H]$^+$ ($C_{37}H_{35}N_2$), calcd.: 507.2800; found:507.2793.

(*R*)-7,7'-di-*tert*-butyl-1,1,1',1'-tetramethyl-2,2',4,4'-tetrahydro-1H,1'H-3,3'-spirobi [cyclopenta[b]indole]

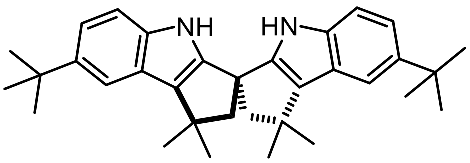

4j

Light yellow solid, m. p. 152.0-153.1 °C. 40.1 mg, 86% yield. **[α]$_D^{25}$** = -103.2 (*c* = 0.5, CH_2Cl_2). **SFC**: ee = 94% (analytical SFC, Trefoil AMY-1 column, IPA : CO_2 = 10 : 90, flow rate 0.6 mL/min, 2000psi, PDA wavelength: 280 nm): minor isomer: t_R = 3.30 min; major isomer: t_R = 3.91 min. **^{1}H NMR** (400 MHz, CD_2Cl_2) δ 7.57 (s, 4H), 7.25 – 7.13 (m, 4H), 2.81 (d, *J* = 13.1 Hz, 2H), 2.66 (d, *J* = 13.1 Hz, 2H), 1.64 (s, 6H), 1.55 (s, 6H), 1.42 (s, 18H). **^{13}C NMR** (101 MHz, CD_2Cl_2) δ 144.76, 142.62, 139.39, 127.55, 123.49, 119.34, 114.24, 111.26, 62.24, 49.42, 39.23, 34.52, 31.83, 30.23, 30.22. **IR** (neat) 3398, 2952, 2864, 1704, 1627, 1592, 1459, 1380, 1361, 1343, 1293, 1265, 1182, 1105, 871, 833, 801, 732 cm^{-1} **HRMS** (ESI) m/z [M-H]$^-$ ($C_{33}H_{41}N_2$), calcd.: 465.3270; found: 465.3273.

(*R*)-1,1,1',1',7,7'-hexamethyl-2,2',4,4'-tetrahydro-1H,1'H-3,3'-spirobi[cyclopenta[b]indole]

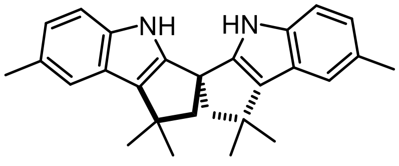

4k

Light yellow solid, m. p. 139.7-141.2 °C. 36.1 mg, 94% yield. **[α]D[25]** = -51.7 (*c* = 1.0, CH_2Cl_2). **SFC**: ee = 95% (analytical SFC, Trefoil AMY-1 column, IPA : CO_2 = 20 : 80, flow rate 0.6 mL/min, 2000psi, PDA wavelength: 280 nm): minor isomer: t_R = 1.99 min; major isomer: t_R = 2.44 min. **^{1}H NMR** (400 MHz, CD_2Cl_2) δ 7.55 (s, 2H), 7.37 (s, 2H), 7.11 (d, *J* = 8.3 Hz, 2H), 6.92 (d, *J* = 8.2 Hz, 2H), 2.78 (d, *J* = 13.1 Hz, 2H), 2.63 (dd, *J* = 13.1, 1.2 Hz, 2H), 2.45 (s, 6H), 1.60 (d, *J* = 1.7 Hz, 6H), 1.51 (s, 6H). **^{13}C NMR** (101 MHz, CD_2Cl_2) δ 144.72, 139.57, 128.74, 127.17, 123.81, 122.52, 118.24, 111.43, 62.14, 49.40, 39.16, 30.16, 30.13, 26.32, 21.26. **IR** (neat) 3410, 2957, 2924, 2864, 1699, 1626, 1492, 1455, 1361, 1294, 1200, 1151, 1128, 1075, 1041, 953, 889, 864, 814, 795, 711 cm^{-1} **HRMS** (ESI) m/z [M+H]$^+$ ($C_{27}H_{31}N_2$), calcd.: 383.2487; found: 383.2483.

(*R*)-dimethyl 1,1,1',1'-tetramethyl-2,2',4,4'-tetrahydro-1H,1'H-3,3'-spirobi[cyclopenta[b]indole]-7,7'-dicarboxylate

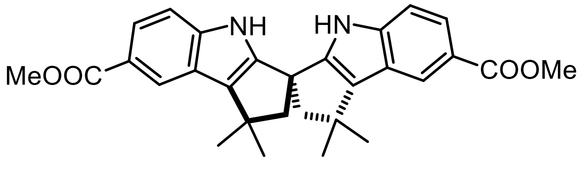

4l

Yellow solid, m. p. >300 °C. 40.0 mg, 85% yield. **[α]D[25]** = -76.1 (*c* = 1.0, CH_2Cl_2). **SFC**: ee = 95% (analytical SFC, Trefoil AMY-1 column, IPA : CO_2 = 20 : 80, flow rate 0.6 mL/min, 2000psi, PDA wavelength: 280 nm): major isomer: t_R = 2.14 min; minor isomer: t_R = 2.64 min. **^{1}H NMR** (400 MHz, Acetone-D_6) δ 10.29 (s, 2H), 8.27 (s, 2H), 7.78 (dd, *J* = 8.6, 0.8 Hz, 2H), 7.38 (d, *J* = 8.6 Hz, 2H), 3.88 (s, 6H), 2.88 (d, *J* = 13.0 Hz, 2H), 2.68 (d, *J* = 13.0 Hz, 2H), 1.60 (s, 6H), 1.53 (s, 6H). **^{13}C NMR** (101 MHz, Acetone-D_6) δ167.59, 146.81, 144.49, 128.09, 123.10, 122.00, 121.21, 120.64, 111.79, 62.15, 51.12, 49.49, 39.23, 30.16. **IR** (neat) 3263, 2951, 2922, 2858, 1714, 1703, 1681, 1618, 1472, 1453, 1436, 1360, 1350, 1295, 1269, 1249, 1230, 1190, 1173, 1119, 1096, 1047, 997, 973, 899, 819, 769, 751, 739, 715, 669 cm^{-1} **HRMS** (ESI) m/z [M+H]$^+$ ($C_{29}H_{31}N_2O_4$), calcd.: 471.2284; found: 471.2278.

(*R*)-6,6'-difluoro-1,1,1',1'-tetramethyl-2,2',4,4'-tetrahydro-1H,1'H-3,3'-spirobi[cyclopenta [b]indole]

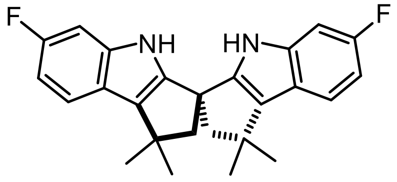
4m

Light yellow solid, m. p. 235.6-237.1 °C. 32.0 mg, 82% yield. **[α]$_D$^{25}** = -27.8 (*c* = 0.50, CH_2Cl_2). **SFC**: ee = 95% (analytical SFC, Trefoil CEL-1 column, IPA : CO_2 = 20 : 80, flow rate 0.6 mL/min, 2000psi, PDA wavelength: 280 nm): major isomer: t_R = 1.38 min; minor isomer: t_R = 2.48 min. **^{1}H NMR** (400 MHz, CD_2Cl_2) δ 7.73 (s, 2H), 7.46 (dd, *J* = 8.6, 5.5 Hz, 2H), 6.96 (dd, *J* = 10.0, 2.3 Hz, 2H), 6.84 (ddd, *J* = 9.8, 8.7, 2.3 Hz, 2H), 2.83 – 2.71 (m, 2H), 2.63 (t, *J* = 7.7 Hz, 2H), 1.57 (s, 6H), 1.49 (s, 6H). **^{13}C NMR** (101 MHz, CD_2Cl_2) δ 160.54, 158.19, 144.56, 144.52, 141.24, 141.12, 127.73, 120.29, 119.00, 118.90, 107.94, 107.70, 98.51, 98.25, 61.95, 49.50, 39.23, 30.11, 30.09. **^{19}F NMR** (282 MHz, CD_2Cl_2) δ -122.42, -122.44, -122.45, -122.47, -122.49, -122.51. **IR** (neat) 3394, 3056, 2954, 2919, 2860, 1571, 1490, 1453, 1440, 1377, 1357, 1341, 1223, 1183, 1130, 1105, 1005, 951, 904, 837, 807, 799, 749, 705 cm^{-1} **HRMS** (ESI) m/z [M+H]$^+$ ($C_{25}H_{25}F_2N_2$), calcd.: 391.1986; found: 391.1982.

(*R*)-6,6'-dichloro-1,1,1',1'-tetramethyl-2,2',4,4'-tetrahydro-1H,1'H-3,3'-spirobi[cyclopenta [b]indole]

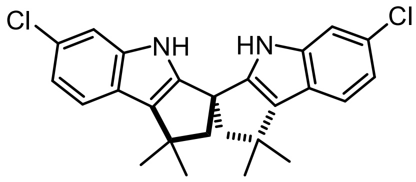
4n

Light yellow solid, m. p. 252.1-254.2 °C. 34.0 mg, 80% yield. **[α]$_D$^{25}** = 27.3 (*c* = 1.0, CH_2Cl_2). **SFC**: ee = 91% (analytical SFC, Trefoil AMY-1 column, MeOH : CO_2 = 30 : 70, flow rate 0.6 mL/min, 2000psi, PDA wavelength: 280 nm): major isomer: t_R = 1.37 min; minor isomer: t_R = 2.92 min. **^{1}H NMR** (400 MHz, CD_2Cl_2) δ 7.76 (s, 2H), 7.46 (d, *J* = 8.4 Hz, 2H), 7.26 (d, *J* = 1.8 Hz, 2H), 7.05 (dd, *J* = 8.4, 1.9 Hz, 2H), 2.77 (d, *J* = 13.2 Hz, 2H), 2.62 (d, *J* = 13.2 Hz, 2H), 1.56 (s, 6H), 1.47 (s, 6H). **^{13}C NMR** (101 MHz, CD_2Cl_2) δ 144.98, 141.57, 127.91, 126.82, 122.16, 120.10, 119.22, 111.77, 61.92, 49.43, 39.21, 30.07, 30.02. **IR** (neat) 3415, 3064, 2950, 2930, 2861,1617,1569, 1453, 1427, 1358,

1333, 1299, 1258, 1246, 1226, 1178, 1151, 1109, 1088, 1061, 1040, 1014, 902, 872, 844, 795, 776, 729, 718, 692 cm^{-1} **HRMS** (ESI) m/z [M+H]$^+$ ($C_{25}H_{25}Cl_2N_2$), calcd.:423.1395; found:423.1392.

(*R*)-6,6'-dibromo-1,1,1',1'-tetramethyl-2,2',4,4'-tetrahydro-1H,1'H-3,3'-spirobi[cyclopenta [b]indole]

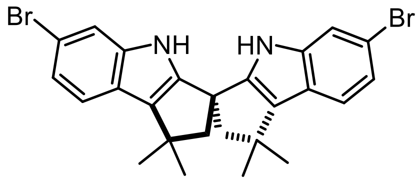

4o

Yellow solid, m. p. 148.7-150.2 °C. 36.0 mg, 79% yield. **[α]$_D^{25}$** = 25.6 (*c* = 0.5, CH_2Cl_2). **SFC**: ee = 92% (analytical SFC, Trefoil AMY-1 column, IPA : CO_2 = 20 : 80, flow rate 0.6 mL/min, 2000psi, PDA wavelength: 280 nm): major isomer: t_R = 4.15 min; minor isomer: t_R = 10.98 min. **^{1}H NMR** (400 MHz, CD_2Cl_2) δ 7.73 (s, 2H), 7.41 (d, *J* = 8.6 Hz, 4H), 7.17 (dd, *J* = 8.4, 1.4 Hz, 2H), 2.76 (d, *J* = 13.2 Hz, 2H), 2.61 (d, *J* = 13.2 Hz, 2H), 1.55 (s, 6H), 1.46 (s, 6H). **^{13}C NMR** (101 MHz, CD_2Cl_2) δ 144.21, 140.74, 128.31, 123.09, 121.89, 121.20, 119.75, 116.12, 62.16, 49.49, 39.35, 30.21, 30.04, 16.61. **IR** (neat) 3439, 3049, 2953, 2925, 2861, 1705, 1609, 1550, 1452, 1424, 1360, 1294, 1214, 1176, 1044, 1008, 946, 907, 848, 801, 729 cm^{-1} **HRMS** (ESI) m/z [M+H]$^+$ ($C_{25}H_{25}Br_2N_2$), calcd.: 511.0384; found: 511.0377.

(*R*)-6,6'-diiodo-1,1,1',1'-tetramethyl-2,2',4,4'-tetrahydro-1H,1'H-3,3'-spirobi[cyclopenta [b]indole]

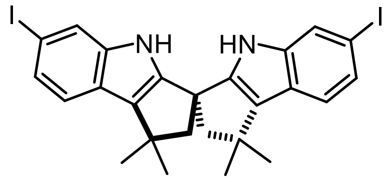

4p

Yellow solid, m. p. 230.0-231.5 °C. 40.2 mg, 66% yield. **[α]$_D^{25}$** = 37.3 (*c* = 0.5, CH_2Cl_2). **SFC**: ee = 78% (analytical SFC, Trefoil AMY-1 column, IPA : CO_2 = 20 : 80, flow rate 0.6 mL/min, 2000psi, PDA wavelength: 280 nm): major isomer: t_R = 7.08 min; minor isomer: t_R = 15.40 min. **^{1}H NMR** (400 MHz, Acetone-D_6) δ 10.02 (s, 2H), 7.70 (d, *J* = 1.2 Hz, 2H), 7.35 (dt, *J* = 8.3, 4.9 Hz, 4H), 2.82 (s, 2H), 2.64 (d, *J* = 13.0 Hz, 2H), 1.55 (s, 6H), 1.48 (s, 6H). **^{13}C NMR** (101 MHz, Acetone-D_6) δ145.85, 143.06, 127.51, 126.89, 122.84, 120.81, 119.93, 83.28, 62.05, 49.12, 38.97, 29.95. **IR** (neat) 3421, 2954, 2921, 2859, 1707, 1609, 1546, 1451, 1421, 1378, 1356, 1326, 1294, 1248, 1217, 1177, 1040, 848, 804, 751, 729, 703 cm^{-1}. **HRMS** (ESI) m/z [M-H]$^-$ ($C_{25}H_{23}I_2N_2$), calcd.: 604.9951; found: 604.9957.

(*R*)-6,6'-dimethoxy-1,1,1',1'-tetramethyl-2,2',4,4'-tetrahydro-1H,1'H-3,3'-spirobi [cyclo penta[b]indole]

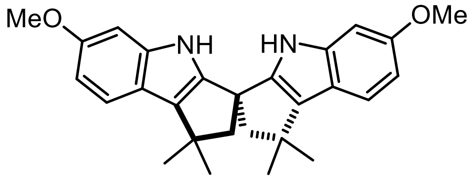

4q

Yellow solid, m. p. 117.5-118.8 °C. 37.0 mg, 70% yield. **[α]$_D$^{25}** = 11.4 (*c* = 0.5, CH_2Cl_2). **SFC**: ee = 85% (analytical SFC, Trefoil AMY-1 column, IPA : CO_2 = 15 : 85, flow rate 0.6 mL/min, 2000psi, PDA wavelength: 280 nm): major isomer: t_R = 5.62 min; minor isomer: t_R = 6.37 min. **^{1}H NMR** (400 MHz, CD_2Cl_2) δ 7.61 (s, 2H), 7.39 (d, *J* = 8.6 Hz, 2H), 6.77 (d, *J* = 2.1 Hz, 2H), 6.71 (dd, *J* = 8.6, 2.2 Hz, 2H), 3.77 (s, 6H), 2.74 (d, *J* = 13.1 Hz, 2H), 2.58 (d, *J* = 13.1 Hz, 2H), 1.54 (s, 6H), 1.46 (s, 6H). **^{13}C NMR** (101 MHz, CD_2Cl_2) δ 155.91, 143.16, 142.08, 127.47, 118.82, 118.02, 108.87, 95.99, 62.07, 55.66, 49.52, 39.15, 30.18. **IR** (neat) 3410, 2950, 2862, 1695, 1623, 1601, 1566, 1499, 1455, 1379, 1360, 1338, 1262, 1235, 1195, 1154, 1112, 1030, 948,801,782, 691 cm^{-1} **HRMS** (ESI) m/z [M+H]$^+$ ($C_{27}H_{31}N_2O_2$), calcd.: 415.2386; found: 415.2379.

(*R*)-1,1,1',1',6,6'-hexamethyl-2,2',4,4'-tetrahydro-1H,1'H-3,3'-spirobi[cyclopenta[b]indole]

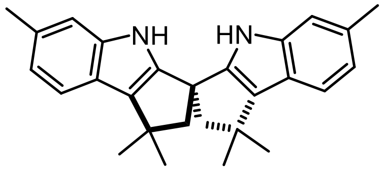

4r

Light yellow solid, m. p. 147.4-148.8 °C. 33.5 mg, 88% yield. **[α]$_D$^{25}** = 24.0 (*c* = 0.5, CH_2Cl_2). **SFC**: ee = 98% (analytical SFC, Trefoil AMY-1 column, IPA : CO_2 = 20 : 80, flow rate 0.6 mL/min, 2000psi, PDA wavelength: 280 nm): major isomer: t_R = 2.22 min; minor isomer: t_R = 3.63 min. **^{1}H NMR** (400 MHz, CD_2Cl_2) δ 7.54 (s, 2H), 7.44 (d, *J* = 8.0 Hz, 2H), 7.18 – 6.98 (m, 2H), 6.92 (d, *J* = 8.0 Hz, 2H), 2.77 (d, *J* = 13.1 Hz, 2H), 2.62 (dd, *J* = 13.1, 0.7 Hz, 2H), 2.42 (s, 6H), 1.58 (s, 6H), 1.49 (s, 6H). **^{13}C NMR** (101 MHz, CD_2Cl_2) δ 143.82, 141.78, 130.94, 127.46, 121.43, 121.14, 118.01, 111.87, 62.11, 49.47, 39.18, 30.18, 21.46. **IR** (neat) 3406, 2952, 2923, 2861, 1698, 1624, 1452, 1361, 1307, 1293, 1233, 1181, 1127, 1080, 1043, 1007, 952, 854, 800, 757, 734, 710 cm^{-1} **HRMS** (ESI) m/z [M+H]$^+$ ($C_{27}H_{31}N_2$), calcd.: 383.2487; found: 383.2482.

(*R*)-6,6'-di-tert-butyl-1,1,1',1'-tetramethyl-2,2',4,4'-tetrahydro-1H,1'H-3,3'-spirobi [cyclopenta[b]indole]

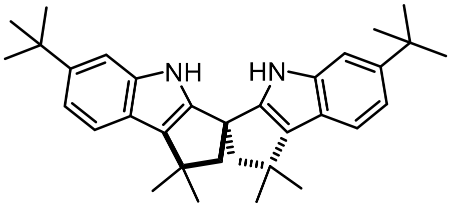

4s

Light yellow solid, m. p.210.0-211.9 °C. 41.4 mg, 89% yield. $[\alpha]_D^{25}$ = 79.2 (*c* = 1.0, CH_2Cl_2). **SFC**: ee = 81% (analytical SFC, Trefoil CEL-1 column, MeOH : CO_2 = 10 : 90, flow rate 0.6 mL/min, 2000psi, PDA wavelength: 280 nm): major isomer: t_R = 3.31 min; minor isomer: t_R = 6.32 min. **^{1}H NMR** (400 MHz, CD_2Cl_2) δ 7.58 (s, 2H), 7.48 (d, *J* = 8.3 Hz, 2H), 7.28 (s, 2H), 7.18 (dd, *J* = 8.4, 1.4 Hz, 2H), 2.78 (d, *J* = 13.1 Hz, 2H), 2.62 (d, *J* = 13.1 Hz, 2H), 1.58 (s, 6H), 1.50 (s, 6H), 1.36 (s, 18H). **^{13}C NMR** (101 MHz, CD_2Cl_2) δ 144.74, 144.24, 141.61, 127.36, 121.30, 117.78, 117.69, 108.32, 62.20, 49.55, 39.18, 34.66, 31.65, 30.24, 30.21. **IR** (neat) 3410, 2955, 2864, 1704, 1626, 1492, 1454, 1361, 11294, 1202, 1179, 1151, 1130, 1077, 1044, 943, 883, 864, 809, 757, 733, 698, 653 cm^{-1} **HRMS** (ESI) m/z [M-H]$^-$ ($C_{33}H_{41}N_2$), calcd.: 465.3270; found: 465.3278.

(*R*)-6,6',7,7'-tetrafluoro-1,1,1',1'-tetramethyl-2,2',4,4'-tetrahydro-1H,1'H-3,3'-spirobi [cyclopenta[b]indole]

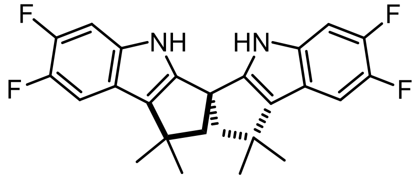

4t

White solid, m. p. >300°C. 40.2 mg, 94% yield. $[\alpha]_D^{25}$ = -28.7 (*c* = 0.5, CH_2Cl_2). **SFC**: ee = 96% (analytical SFC, Trefoil AMY-1 column, IPA : CO_2 = 20 : 80, flow rate 0.6 mL/min, 2000psi, PDA wavelength: 280 nm): major isomer: t_R = 1.00 min; minor isomer: t_R = 1.85 min. **^{1}H NMR** (400 MHz, Acetone-D_6) δ 9.93 (s, 2H), 7.28 (dd, *J* = 11.3, 8.0 Hz, 2H), 7.10 (dd, *J* = 11.4, 7.0 Hz, 2H), 2.70 (d, *J* = 13.0 Hz, 2H), 2.50 (d, *J* = 13.0 Hz, 2H), 1.42 (s, 6H), 1.35 (s, 6H). **^{13}C NMR** (101 MHz, Acetone-D_6) δ148.12, 147.97, 147.04, 147.01, 146.86, 145.77, 145.61, 144.67, 144.53, 136.82, 136.72, 127.08, 127.04, 118.91, 118.84, 104.72, 104.53, 100.05, 99.84, 62.17, 49.42, 38.99, 29.88. **^{19}F NMR** (282 MHz, Acetone) δ 29.55, 29.52, 29.51, 29.48, 29.48, 29.45, 29.44, 29.41, 27.18, 27.15, 27.14, 27.11, 27.10, 27.08, 27.06, 27.04. **IR** (neat) 3410, 2952, 2932, 2862, 1700, 1634, 1590, 1458, 1379, 1358, 1340, 1288, 1247, 1227, 1158, 1100, 1048, 1000, 948, 834 cm^{-1} **HRMS** (ESI) m/z [M+H]$^+$ ($C_{25}H_{23}F_4N_2$), calcd.: 427.1797; found: 427.1793.

(*R*)-7,7'-dibromo-1,1,1',1',6,6'-hexamethyl-2,2',4,4'-tetrahydro-1H,1'H-3,3'-spirobi [cyclopenta[b]indole]

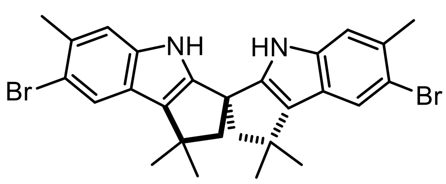

4u

Yellow solid, m. p. 168.5-170.6 °C. 37.2 mg, 69% yield. **[α]$_D$^{25}** = -46.5 (c = 1.0, CH_2Cl_2). **SFC**: ee = 73% (analytical SFC, Trefoil AMY-1 column, MeOH : CO_2 = 30 : 70, flow rate 0.6 mL/min, 2000psi, PDA wavelength: 280 nm): major isomer: t_R = 3.29 min; minor isomer: t_R = 4.16 min. **^{1}H NMR** (400 MHz, CD_2Cl_2) δ 7.71 (s, 2H), 7.64 (s, 2H), 7.15 (s, 2H), 2.74 (d, J = 13.1 Hz, 2H), 2.59 (d, J = 13.1 Hz, 2H), 2.43 (s, 6H), 1.53 (s, 6H), 1.45 (s, 6H). **^{13}C NMR** (101 MHz, CD_2Cl_2) δ 144.96, 140.75, 129.91, 127.15, 123.29, 121.51, 115.85, 113.31, 61.90, 49.37, 39.13, 30.04, 30.02, 23.24. **IR** (neat) 3416, 2951, 2925, 2861, 1702, 1595, 1450, 1418, 1378, 1361, 1292, 1269, 1173, 1153, 1124, 1093, 1014, 958, 880, 859, 669 cm^{-1} **HRMS** (ESI) m/z [M+H]$^+$ ($C_{27}H_{29}Br_2N_2$), calcd.: 539.0697; found: 539.0691.

(*R*)-7,7'-dichloro-1,1,1',1',6,6'-hexamethyl-2,2',4,4'-tetrahydro-1H,1'H-3,3'-spirobi [cyclopenta[b]indole]

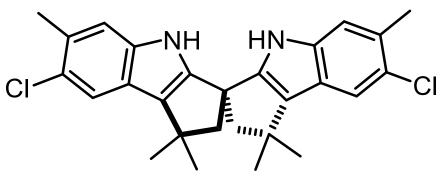

4v

Light yellow solid, m. p. 144.4-145.6 °C. 39.9 mg, 88% yield. **[α]$_D$^{25}** = -65.5 (c = 1.0, CH_2Cl_2). **SFC**: ee = 99% (analytical SFC, Trefoil AMY-1 column, IPA : CO_2 = 20 : 80, flow rate 0.6 mL/min, 2000psi, PDA wavelength: 280 nm): major isomer: t_R = 2.16 min; minor isomer: t_R = 6.25 min. **^{1}H NMR** (400 MHz, CD_2Cl_2) δ 7.63 (s, 2H), 7.52 (s, 2H), 7.13 (s, 2H), 2.75 (d, J = 13.1 Hz, 2H), 2.60 (d, J = 13.1 Hz, 2H), 2.41 (s, 6H), 1.54 (s, 6H), 1.46 (s, 6H). **^{13}C NMR** (101 MHz, CD_2Cl_2) δ 145.05, 140.22, 128.52, 127.30, 125.90, 122.73, 118.19, 113.36, 61.92, 49.40, 39.12, 30.04, 30.01, 20.45. **IR** (neat) 3411, 2954, 2928, 2862, 1703, 1598, 1452, 1379, 1362, 1328, 1294, 1278,1175, 1125, 1095, 1006, 952, 881, 860, 736, 687 cm^{-1} **HRMS** (ESI) m/z [M+H]$^+$ ($C_{27}H_{29}Cl_2N_2$), calcd.: 451.1708; found: 451.1704.

(*R*)-6,6'-difluoro-1,1,1',1',7,7'-hexamethyl-2,2',4,4'-tetrahydro-1H,1'H-3,3'-spirobi [cyclopenta[b]indole]

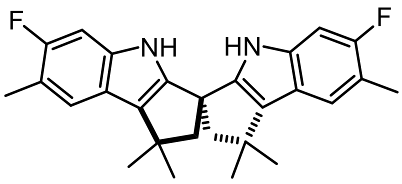

4w

Light yellow solid, m. p. 139.9-140.8 °C. 38.1 mg, 91% yield. $[\alpha]_D^{25}$ = -77.4 (*c* = 0.5, CH_2Cl_2). **SFC**: ee = 97% (analytical SFC, Trefoil AMY-1 column, IPA : CO_2 = 20 : 80, flow rate 0.6 mL/min, 2000psi, PDA wavelength: 280 nm): major isomer: t_R = 1.38 min; minor isomer: t_R = 2.39 min. **^{1}H NMR** (400 MHz, CD_2Cl_2) δ 7.60 (s, 2H), 7.30 (d, *J* = 7.6 Hz, 2H), 6.92 (d, *J* = 10.7 Hz, 2H), 2.74 (d, *J* = 13.1 Hz, 2H), 2.59 (d, *J* = 13.1 Hz, 2H), 2.34 (d, *J* = 1.7 Hz, 6H), 1.55 (s, 6H), 1.47 (s, 6H). **^{13}C NMR** (101 MHz, CD_2Cl_2) δ 159.24, 156.89, 144.42, 144.39, 139.67, 139.56, 127.25, 120.13, 119.69, 119.63, 117.02, 116.83, 98.14, 97.87, 62.01, 49.45, 39.15, 30.10, 14.80, 14.76. **^{19}F NMR** (282 MHz, CD_2Cl_2) δ -125.50, -125.51, -125.52, -125.53, -125.54, -125.55, -125.57, -125.57. **IR** (neat) 3392, 2956, 2930, 2864, 1703, 1632, 1594, 1461, 1381, 1362, 1343, 1299, 1252, 1230, 1150, 1107, 1050, 1001,952, 885, 862, 834 cm^{-1} **HRMS** (ESI) m/z $[M+H]^+$ ($C_{27}H_{29}F_2N_2$), calcd.: 419.2299; found: 419.2294.

(*R*)-6,6'-dibromo-1,1,1',1',7,7'-hexamethyl-2,2',4,4'-tetrahydro-1H,1'H-3,3'-spirobi[cyclopenta[b]indole]

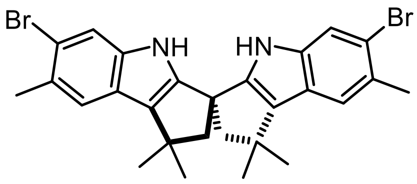

4x

Light yellow solid, m. p. 190.9-192.2 °C. 42.0 mg, 78% yield. $[\alpha]_D^{25}$ = 26.8 (*c* = 0.8, CH_2Cl_2). **SFC**: ee = 93% (analytical SFC, Trefoil AMY-1 column, IPA : CO_2 = 20 : 80, flow rate 0.6 mL/min, 2000psi, PDA wavelength: 280 nm): major isomer: t_R = 3.52 min; minor isomer: t_R = 7.64 min. **^{1}H NMR** (400 MHz, Acetone-D_6) δ 9.89 (s, 2H), 7.52 (d, *J* = 10.9 Hz, 4H), 2.82 (d, *J* = 13.0 Hz, 2H), 2.62 (d, *J* = 13.0 Hz, 2H), 2.48 (s, 6H), 1.55 (s, 6H), 1.48 (s, 6H). **^{13}C NMR** (101 MHz, Acetone-D_6) δ 146.20, 141.10, 126.91, 126.31, 123.30, 119.32, 116.38, 115.17, 62.04, 49.27, 38.88, 29.94, 22.37. **IR** (neat) 3443, 2953, 2924, 2886, 1700, 1654, 1618, 1542, 1360, 1292, 1226, 1154, 1077, 992, 854, 789, 727 cm^{-1} **HRMS** (ESI) m/z $[M-H]^-$ ($C_{27}H_{27}Br_2N_2$), calcd.: 537.0541; found: 537.0548.

(*R*)-8,8'-difluoro-1,1,1',1'-tetramethyl-2,2',4,4'-tetrahydro-1H,1'H-3,3'-spirobi[cyclopenta[b]indole]

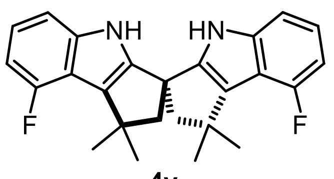

4y

Light yellow solid, m. p. 200.0-201.9 °C. 33.1 mg, 82% yield. **$[\alpha]_D^{25}$** = -17.5 (*c* = 0.5, CH_2Cl_2). **SFC**: ee = 93% (analytical SFC, Trefoil AMY-1 column, IPA : CO_2 = 20 : 80, flow rate 0.6 mL/min, 2000psi, PDA wavelength: 280 nm): major isomer: t_R = 1.76 min; minor isomer: t_R = 2.39 min. **^{1}H NMR** (400 MHz, CD_2Cl_2) δ 7.81 (s, 2H), 7.02 (ddd, *J* = 13.2, 9.1, 6.6 Hz, 4H), 6.76 (ddd, *J* = 10.4, 7.5, 1.0 Hz, 2H), 2.80 (d, *J* = 13.2 Hz, 2H), 2.65 (d, *J* = 13.2 Hz, 2H), 1.56 (s, 6H), 1.48 (s, 6H). **^{13}C NMR** (101 MHz, CD_2Cl_2) δ 157.07, 154.64, 144.17, 143.92, 143.79, 126.22, 126.20, 121.91, 121.83, 112.69, 112.45, 107.92, 107.89, 104.85, 104.66, 61.85, 49.50, 39.04, 30.65, 30.62. **^{19}F NMR** (282 MHz, CD_2Cl_2) δ -119.06, -119.09, -119.10. **IR** (neat) 3465, 2956, 2927, 2869, 1707, 1684, 1594, 1460, 1380, 1360, 1316, 1288, 1271, 1249, 1222, 1192, 1160, 1113, 1100, 1039, 936, 906, 846, 831, 794, 777, 736, 701 cm^{-1} **HRMS** (ESI) m/z $[M-H]^-$ ($C_{25}H_{23}F_2N_2$), calcd.: 389.1829; found: 389.1830.

(*R*)-5,5'-difluoro-1,1,1',1'-tetramethyl-2,2',4,4'-tetrahydro-1H,1'H-3,3'-spirobi[cyclopenta[b]indole]

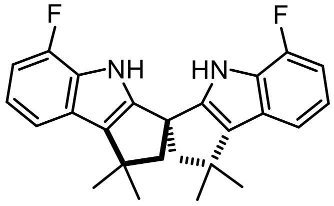

4z

Light yellow solid, m. p. 225.3-227.3 °C. 10.0 mg, 25% yield. **$[\alpha]_D^{25}$** = 12.2 (*c* = 0.5, CH_2Cl_2). **SFC**: ee = 32% (analytical SFC, Trefoil AMY-1 column, IPA : CO_2 = 10 : 90, flow rate 0.6 mL/min, 2000psi, PDA wavelength: 280 nm): major isomer: t_R = 2.58 min; minor isomer: t_R = 3.68 min. **^{1}H NMR** (400 MHz, CD_2Cl_2) δ 7.90 (s, 2H), 7.40 – 7.26 (m, 2H), 7.01 (td, *J* = 7.9, 4.9 Hz, 2H), 6.85 (ddd, *J* = 11.4, 7.9, 0.7 Hz, 2H), 2.83 (d, *J* = 13.2 Hz, 2H), 2.67 (d, *J* = 13.2 Hz, 2H), 1.59 (s, 6H), 1.50 (s, 6H). **^{13}C NMR** (101 MHz, CD_2Cl_2) δ 150.97, 148.55, 145.01, 128.99, 128.86, 126.99, 126.94, 119.89, 119.82, 114.43, 114.40, 106.38, 106.22, 62.18, 49.21, 39.17, 30.07, 29.98. **^{19}F NMR** (282 MHz, CD_2Cl_2) δ -135.33, -135.34, -135.37, -135.38. **IR** (neat) 3458, 3044, 2953, 2927, 2861, 1637, 1570, 1499, 1456, 1359, 1303, 1251, 1233, 1180, 1157, 1110, 1023, 959,934, 863, 815, 779, 732 cm^{-1} **HRMS** (ESI) m/z $[M+H]^+$ ($C_{25}H_{25}F_2N_2$), calcd.: 391.1986; found: 391.1980.

(*R*)-1,1,1',1',5,5'-hexamethyl-2,2',4,4'-tetrahydro-1H,1'H-3,3'-spirobi[cyclopenta[b]indole]

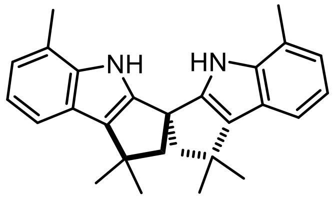

4aa

Light yellow solid, m. p. >300°C. 30.1 mg, 79% yield. **[α]$_D^{25}$** = -0.4 (*c* = 0.5, CH_2Cl_2). **SFC**: ee = 24% (analytical SFC, Trefoil AMY-1 column, IPA : CO_2 = 30 : 70, flow rate 0.6 mL/min, 2000psi, PDA wavelength: 280 nm): minor isomer: t_R = 1.23 min; major isomer: t_R = 1.41 min. **^{1}H NMR** (400 MHz, CD_2Cl_2) δ 7.68 (s, 2H), 7.43 (d, *J* = 7.8 Hz, 2H), 7.02 (t, *J* = 7.5 Hz, 2H), 6.93 (d, *J* = 7.2 Hz, 2H), 2.85 (d, *J* = 13.0 Hz, 2H), 2.68 (d, *J* = 13.1 Hz, 2H), 2.40 (s, 6H), 1.62 (s, 6H), 1.52 (s, 6H). **^{13}C NMR** (101 MHz, CD_2Cl_2) δ 144.21, 140.74, 128.31, 123.09, 121.89, 121.20, 119.75, 116.12, 62.16, 49.49, 39.35, 30.21, 30.04, 16.61. **IR** (neat) 3468, 3049, 2949, 2918, 2859, 1698, 1615, 1588, 1452, 1407, 1379, 1358, 1299, 1248, 1177, 1132, 1108, 1062, 950, 853, 824, 780, 746 cm^{-1} **HRMS** (ESI) m/z [M+H]$^+$ ($C_{27}H_{31}N_2$), calcd.: 383.2487; found: 383.2484.

(*R*)-5,5'-bis(benzyloxy)-1,1,1',1'-tetramethyl-2,2',4,4'-tetrahydro-1H,1'H-3,3'-spirobi[cyclopenta[b]indole]

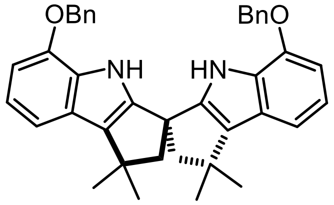

4bb

Yellow solid, m. p. 174.5-175.8 °C. 25.5 mg, 45% yield. **[α]$_D^{25}$** = -13.5 (*c* = 0.55, CH_2Cl_2). **SFC**: ee = 13% (analytical SFC, Trefoil AMY-1 column, IPA : CO_2 = 30 : 70, flow rate 0.6 mL/min, 2000psi, PDA wavelength: 280 nm): minor isomer: t_R = 2.09 min; major isomer: t_R = 7.62 min. **^{1}H NMR** (400 MHz, CD_2Cl_2) δ 7.94 (s, 2H), 7.45 (d, *J* = 7.2 Hz, 4H), 7.36 (dd, *J* = 11.4, 4.4 Hz, 4H), 7.31 (d, *J* = 7.1 Hz, 2H), 7.18 (d, *J* = 7.9 Hz, 2H), 6.98 (t, *J* = 7.9 Hz, 2H), 6.69 (d, *J* = 7.8 Hz, 2H), 5.17 (s, 4H), 2.79 (d, *J* = 13.0 Hz, 2H), 2.63 (d, *J* = 13.0 Hz, 2H), 1.56 (s, 6H), 1.48 (s, 6H). **^{13}C NMR** (101 MHz, CD_2Cl_2) δ 145.56, 144.08, 137.28, 131.30, 128.54, 128.40, 128.04, 127.81, 124.79, 119.77, 111.64, 102.94, 70.19, 62.20, 49.23, 39.16, 30.19, 29.98. **IR** (neat) 3391, 3067, 2949,2931,2861, 1625, 1569, 1496,1452, 1361, 1302, 1259, 1246, 1229, 1179, 1111, 1088, 1039, 1027, 1014, 922, 844, 776, 719, 730, 692 cm^{-1} **HRMS** (ESI) m/z [M+H]$^+$ ($C_{39}H_{39}N_2O_2$), calcd.: 567.3012; found: 567.3008.

10 Crystal Data

10.1 Crystal Data and Experimental of 4d

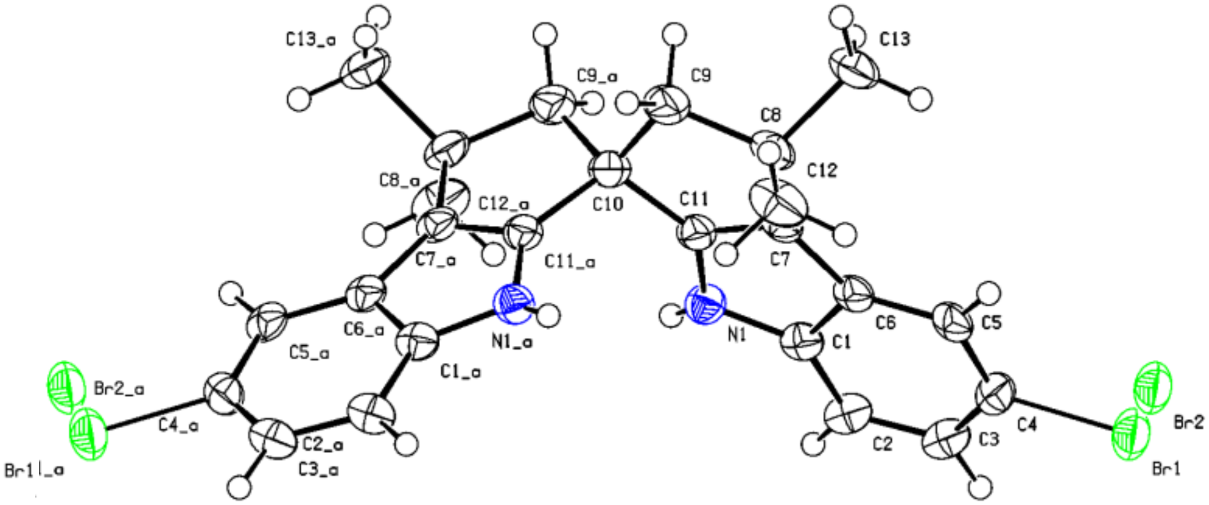

Experimental. Single colourless prism-shaped crystals of **4d** recrystallised from a mixture of DCM and hexanes by slow evaporation. A suitable crystal with dimensions 0.19 × 0.18 × 0.13 mm^3 was selected and mounted on a MITIGEN holder in oil on a Bruker APEX II area detector diffractometer.[24] The crystal was kept at a steady T = 100(2) K during data collection. The structure was solved with the **ShelXT 2018/2** [25] solution program using Intrinsic Phasing methods and by using **Olex2 1.5** [26] as the graphical interface. The model was refined with **XL** [24] using full matrix least squares minimisation on $\boldsymbol{F^2}$.

Crystal Data. $C_{25}H_{24}N_2Br_2$, M_r = 512.28, tetragonal, $P4_12_12$ (No. 92), a = 7.84020(10) Å, b = 7.84020(10) Å, c = 36.1939(12) Å, $\alpha = \beta = \gamma = 90^\circ$, V = 2224.79(9) Å^3, T = 100(2) K, Z = 4, Z' = 0.5, $\mu(\text{CuK}_\alpha)$ = 4.712, 29321 reflections measured, 2022 unique (R_{int} = 0.0385) which were used in all calculations. The final wR_2 was 0.0825 (all data) and R_1 was 0.0319 (I≥2 σ(I)).

10.2 Crystal Data and Experimental of 11b

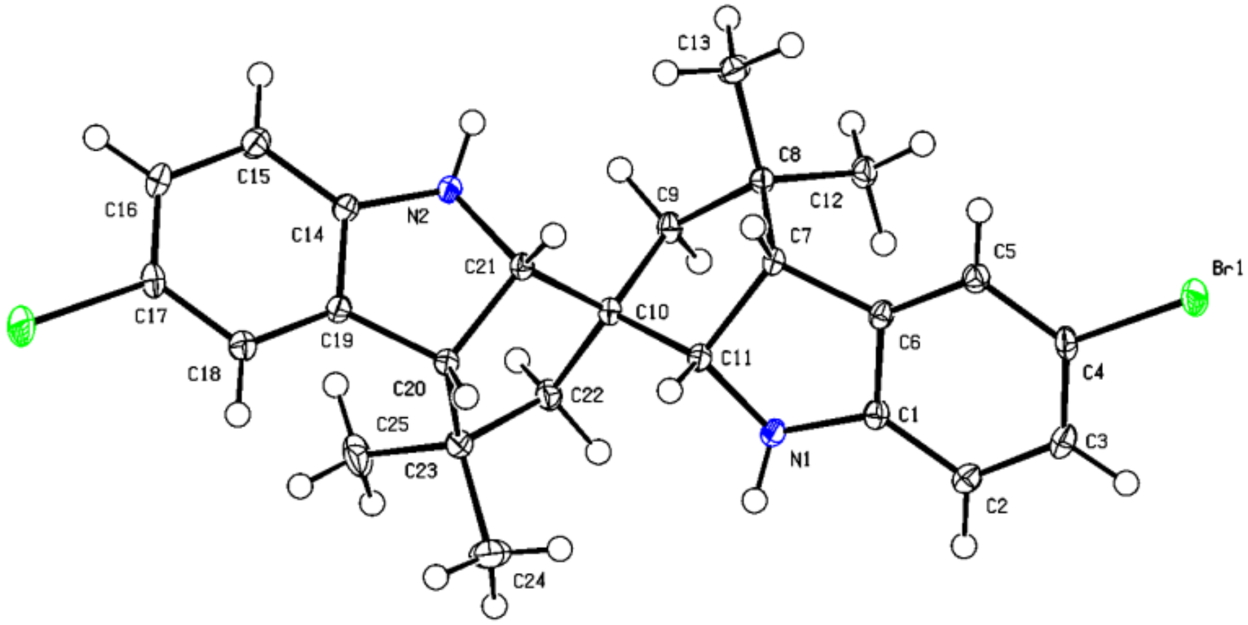

Experimental. Single colourless prism-shaped crystals of **11b** recrystallised from a mixture of Et_2O and hexanes by slow evaporation. A suitable crystal with dimensions 0.24 × 0.18 × 0.08 mm^3 was selected and mounted on a mylar loop in oil on a Bruker APEX II area detector diffractometer. [24] The crystal was kept at a steady T = 100(2) K during data collection. The structure was solved with the **ShelXT 2018/2** [25] solution program using Intrinsic Phasing methods and by using **Olex2 1.5** [26] as the graphical interface. The model was refined with **XL** [24] using full matrix least squares minimisation on $\boldsymbol{F^2}$.

Crystal Data. $C_{25}H_{28}Br_2N_2$, M_r = 516.31, monoclinic, $P2_1$ (No. 4), a = 10.544(5) Å, b = 10.605(7) Å, c = 10.591(6) Å, β = 110.480(16)°, $\alpha = \gamma$ = 90°, V = 1109.5(11) Å^3, T = 100(2) K, Z = 2, Z' = 1, μ (MoK_α) = 3.667, 29124 reflections measured, 6738 unique (R_{int} = 0.0203) which were used in all calculations. The final wR_2 was 0.0424 (all data) and R_1 was 0.0179 (I≥2 σ(I)).

10.3 Crystal Data and Experimental of Pd complex 13

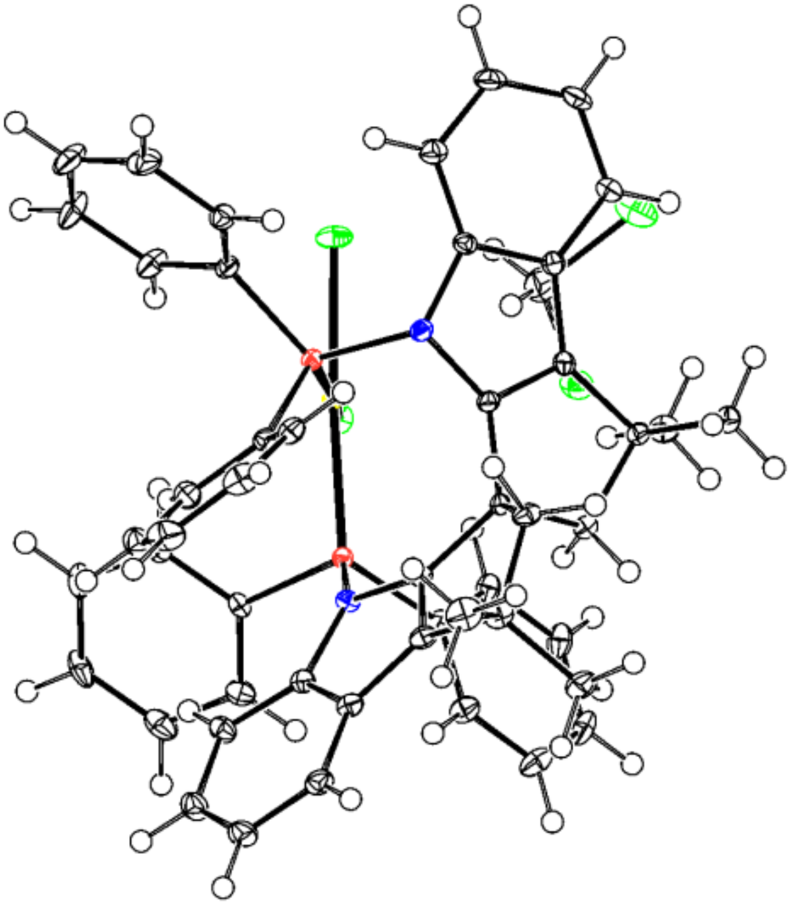

Experimental. Single orange needle-shaped crystals of **13** recrystallised from a mixture of DCM and hexanes by slow evaporation. A suitable crystal with dimensions 0.35 × 0.08 × 0.05 mm^3 was selected and mounted on a mylar loop in oil on a Bruker APEX II area detector diffractometer. [24] The crystal was kept at a steady T = 100(2) K during data collection. The structure was solved with the **ShelXT 2018/2** [25] solution program using Intrinsic Phasing methods and by using **Olex2 1.5** [26] as the graphical interface. The model was refined with **XL** [24] using full matrix least squares minimisation on $\boldsymbol{F}^2$.

Crystal Data. $C_{51}H_{48}N_2P_2Cl_6Pd$, M_r = 1069.95, orthorhombic, $P2_12_12_1$ (No. 19), a = 10.5687(13) Å, b = 12.8239(16) Å, c = 35.318(4) Å, $\alpha = \beta = \gamma =$ 90°, V = 4786.7(10) Å^3, T = 100(2) K, Z = 4, Z' = 1, μ(MoK$_\alpha$) = 0.828, 122941 reflections measured, 14584 unique (R_{int} = 0.0468) which were used in all calculations. The final wR_2 was 0.0609 (all data) and R_1 was 0.0283 (I≥2 σ(I)).

Compound	4d	11b	13
CCDC deposit NO.	CCDC-2408120	CCDC-2408122	CCDC-2408121
Formula	$C_{25}H_{24}N_2Br_2$	$C_{25}H_{28}Br_2N_2$	$C_{51}H_{48}N_2P_2Cl_6Pd$
$D_{calc.}$/ g cm^{-3}	1.529	1.545	1.485
μ/mm^{-1}	4.712	3.667	0.828
Formula Weight	512.28	516.31	1069.95
Colour	colourless	colourless	orange
Shape	prism-shaped	prism-shaped	needle-shaped
Size/mm^3	0.19×0.18×0.13	0.24×0.18×0.08	0.35×0.08×0.05
T/K	100(2)	100(2)	100(2)
Crystal System	tetragonal	monoclinic	orthorhombic
Flack Parameter	-0.015(9)	-0.0126(19)	-0.014(5)
Hooft Parameter	-0.015(9)	-0.0072(16)	-0.022(5)
Space Group	$P4_12_12$	$P2_1$	$P2_12_12_1$
a/Å	7.84020(10)	10.544(5)	10.5687(13)
b/Å	7.84020(10)	10.605(7)	12.8239(16)
c/Å	36.1939(12)	10.591(6)	35.318(4)
α/°	90	90	90
β/°	90	110.480(16)	90
γ/°	90	90	90
V/Å^3	2224.79(9)	1109.5(11)	4786.7(10)
Z	4	2	4
Z'	0.5	1	1
Wavelength/Å	1.54178	0.71073	0.71073
Radiation type	CuK$_\alpha$	MoK$_\alpha$	MoK$_\alpha$

$\Theta_{min}/°$	4.887	2.053	1.689
$\Theta_{max}/°$	67.966	30.556	30.510
Measured Refl's.	29321	29124	122941
Indep't Refl's	2022	6738	14584
Refl's I≥2 σ(I)	1987	6541	13766
R_{int}	0.0385	0.0203	0.0468
Parameters	142	274	591
Restraints	0	1	81
Largest Peak	0.506	0.424	0.563
Deepest Hole	-0.263	-0.378	-0.999
GooF	1.189	1.047	1.095
wR_2 (all data)	0.0825	0.0424	0.0609
wR_2	0.0822	0.0420	0.0598
R_1 (all data)	0.0325	0.0191	0.0318
R_1	0.0319	0.0179	0.0283

11 NMR spectra

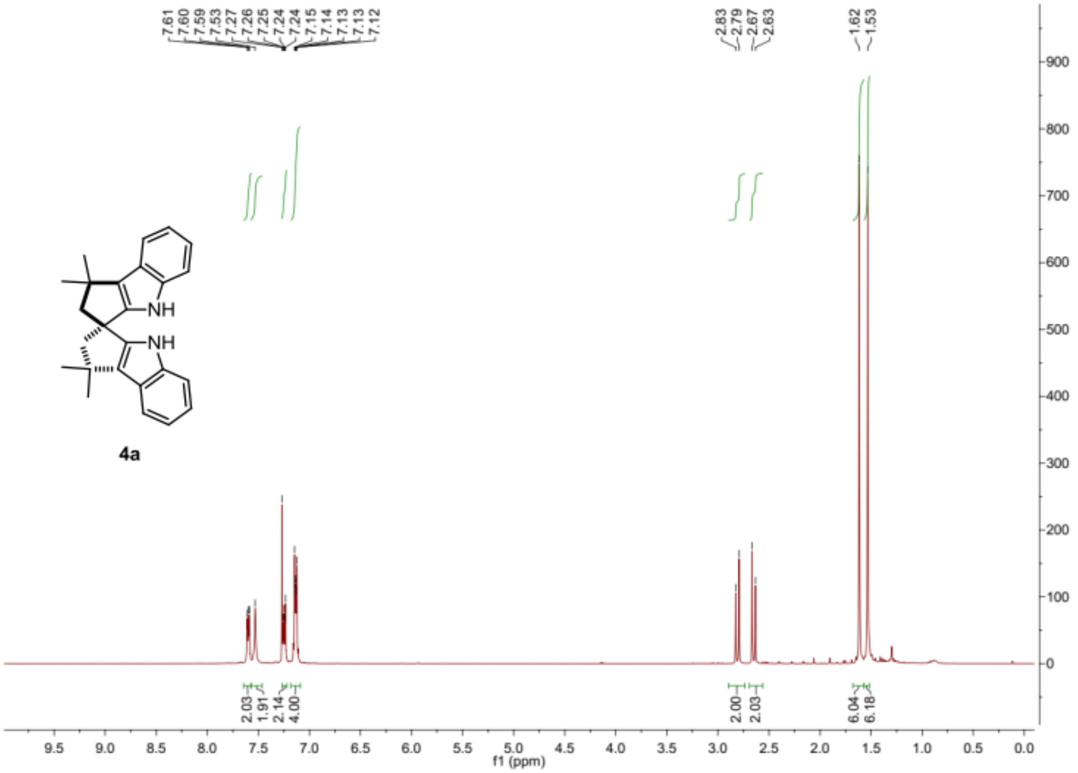

^{1}H NMR (400 MHz, $CDCl_3$, rt)

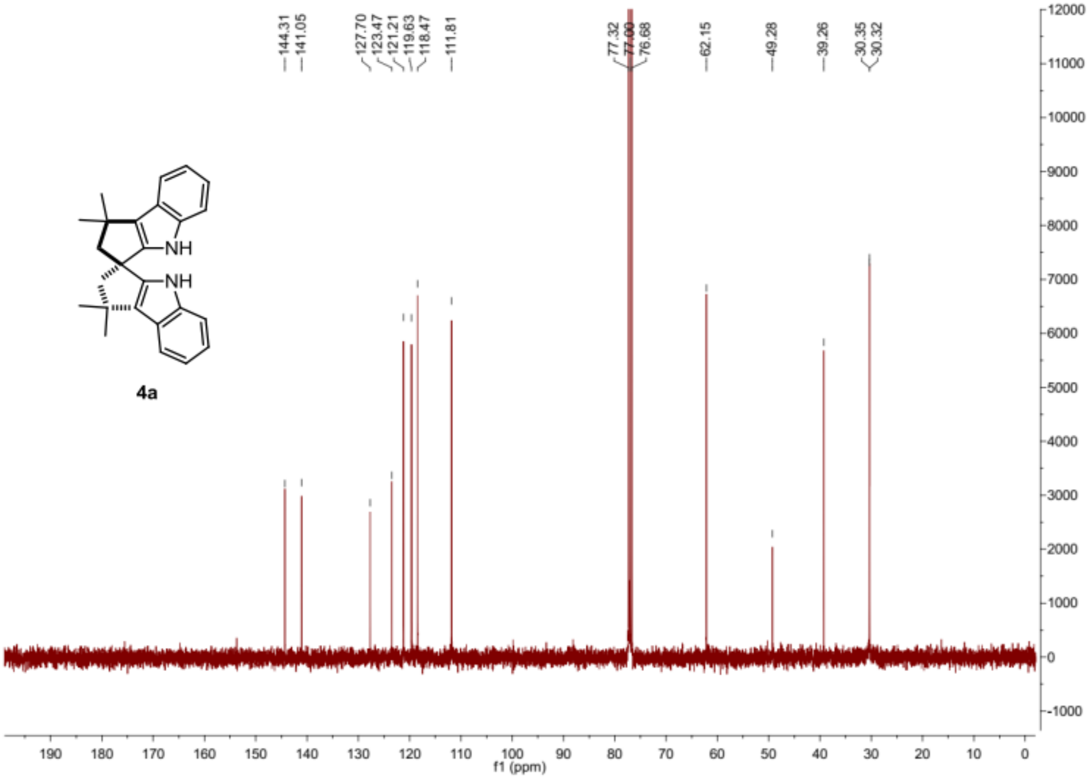

^{13}C NMR (101 MHz, $CDCl_3$, rt)

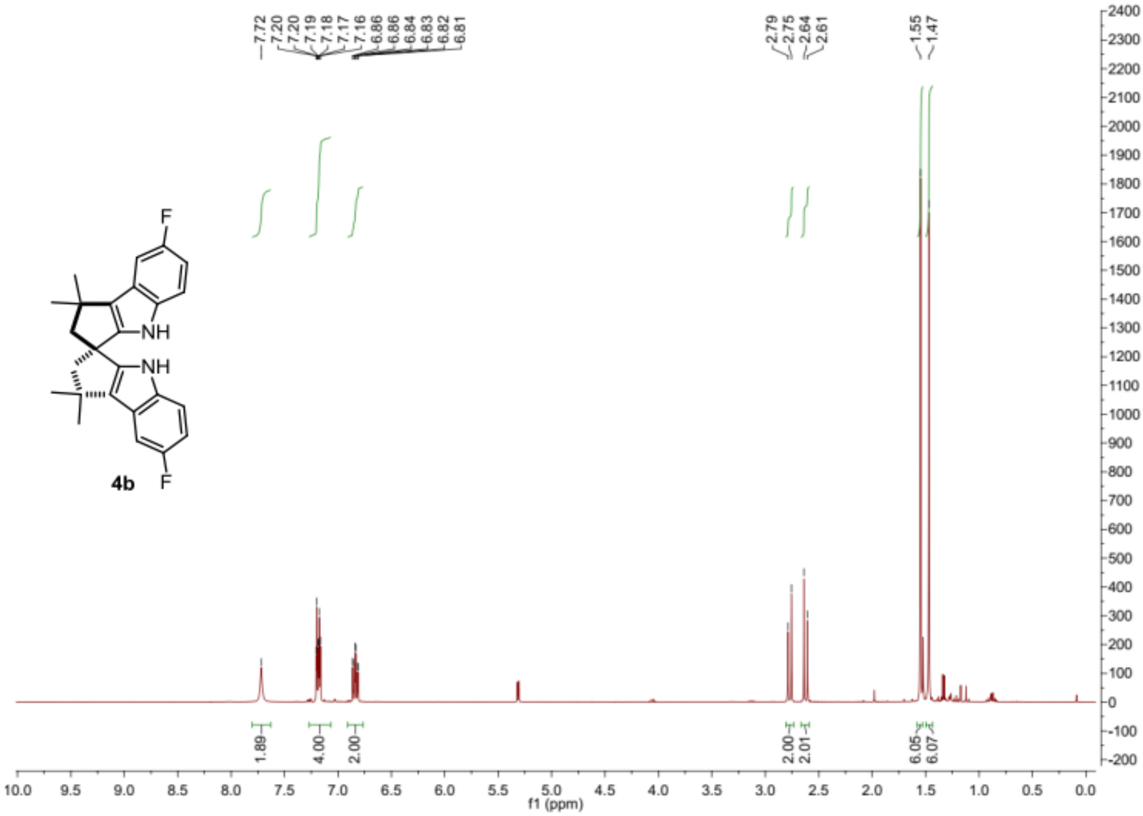

^1H NMR (400 MHz, CD_2Cl_2, rt)

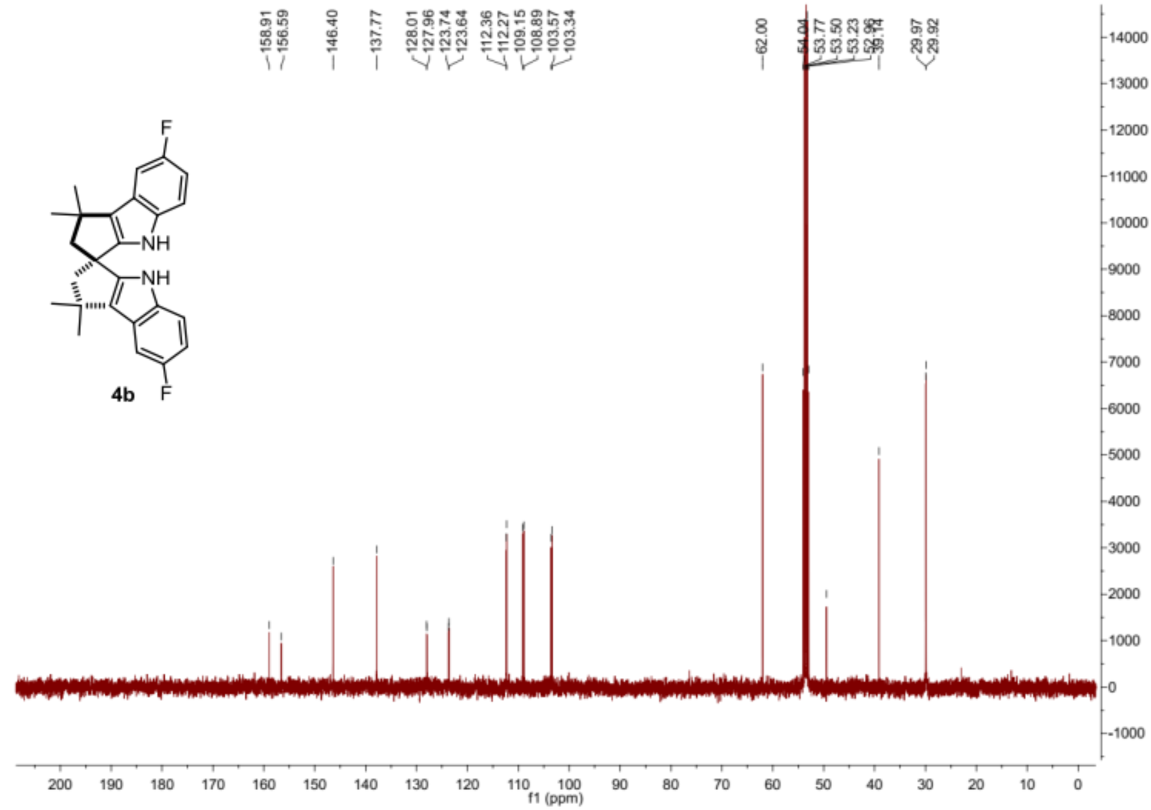

^{13}C NMR (101 MHz, CD_2Cl_2, rt)

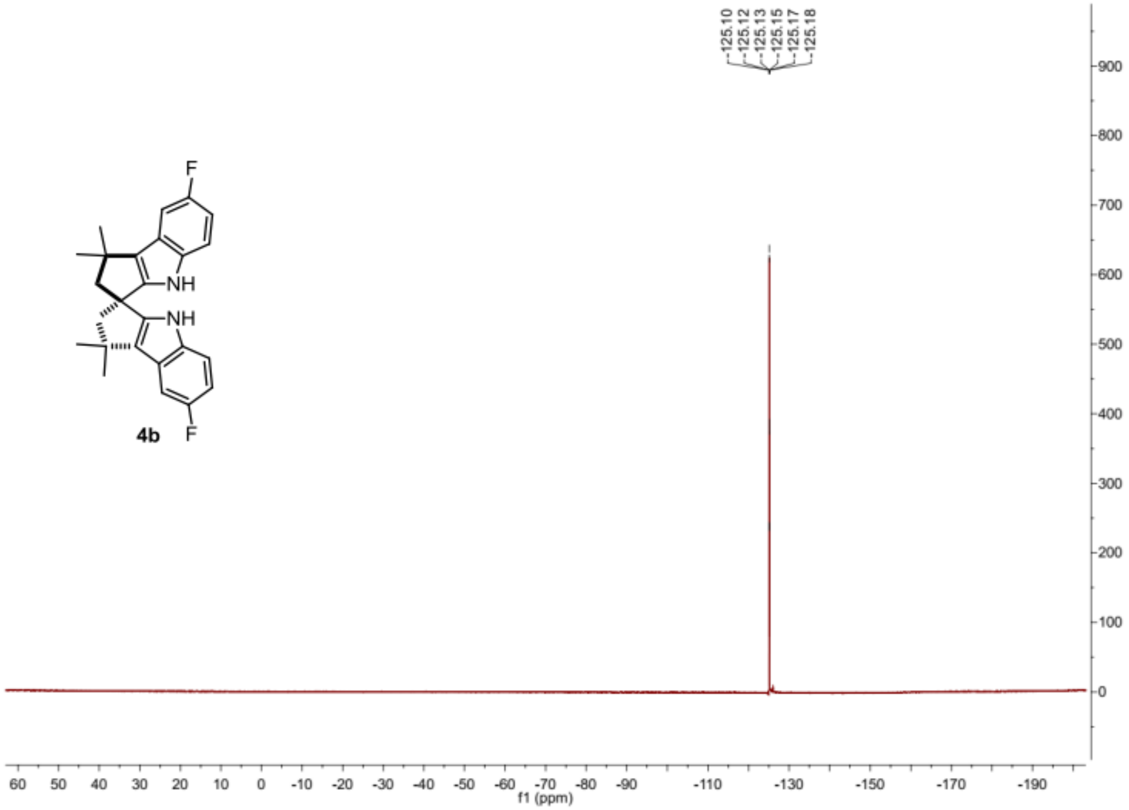

^{19}F NMR (282 MHz, CD_2Cl_2, rt)

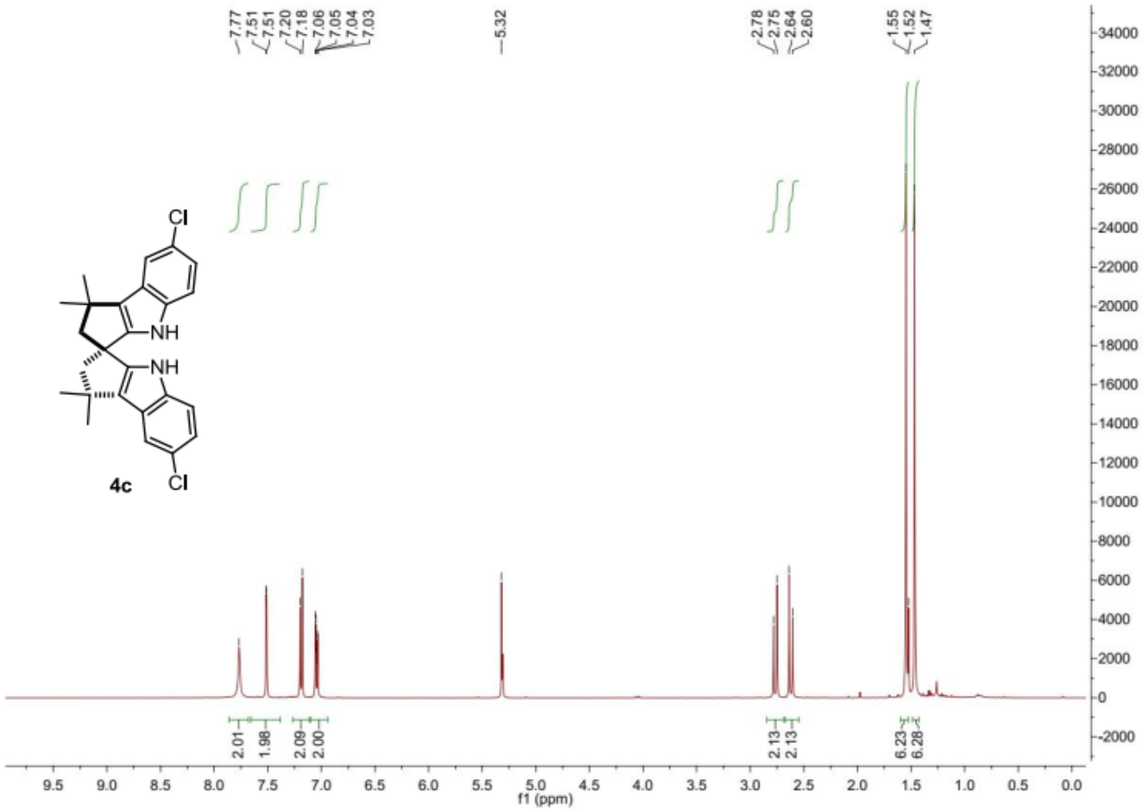

^{1}H NMR (400 MHz, CD_2Cl_2, rt)

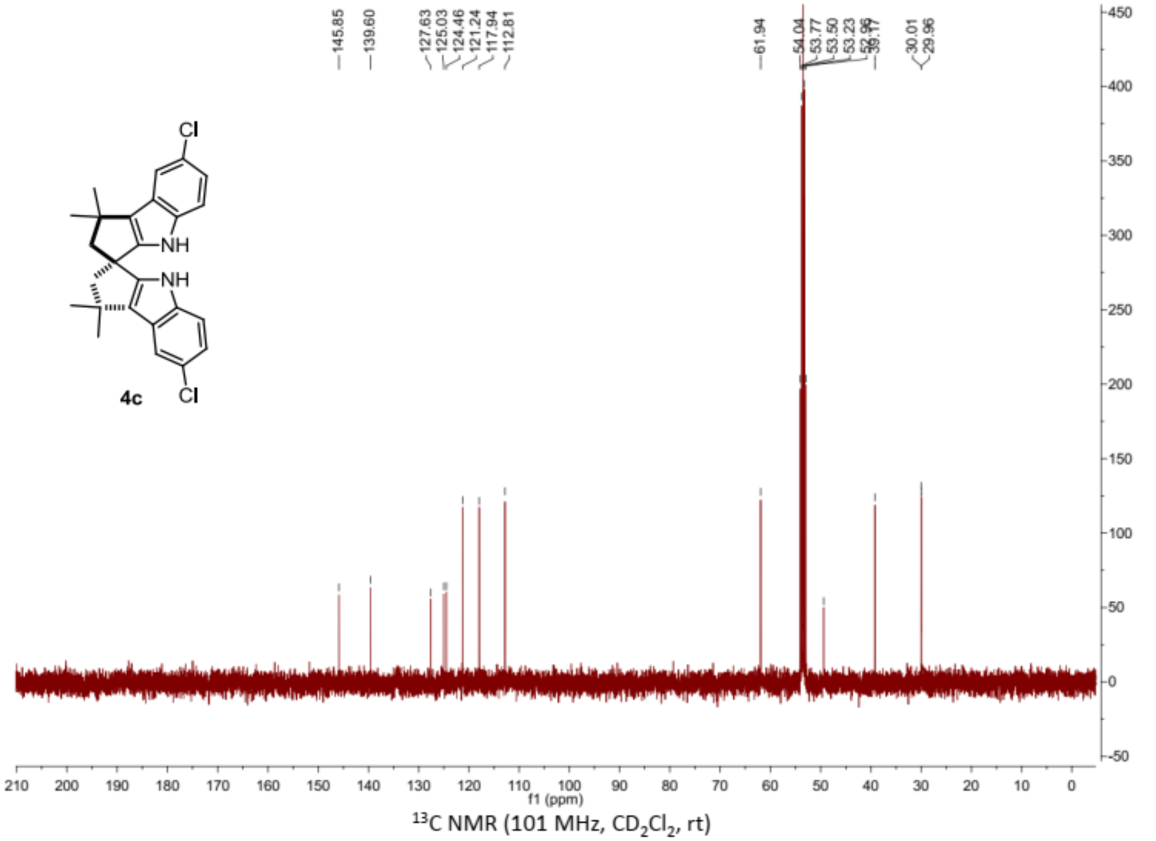

^{13}C NMR (101 MHz, CD_2Cl_2, rt)

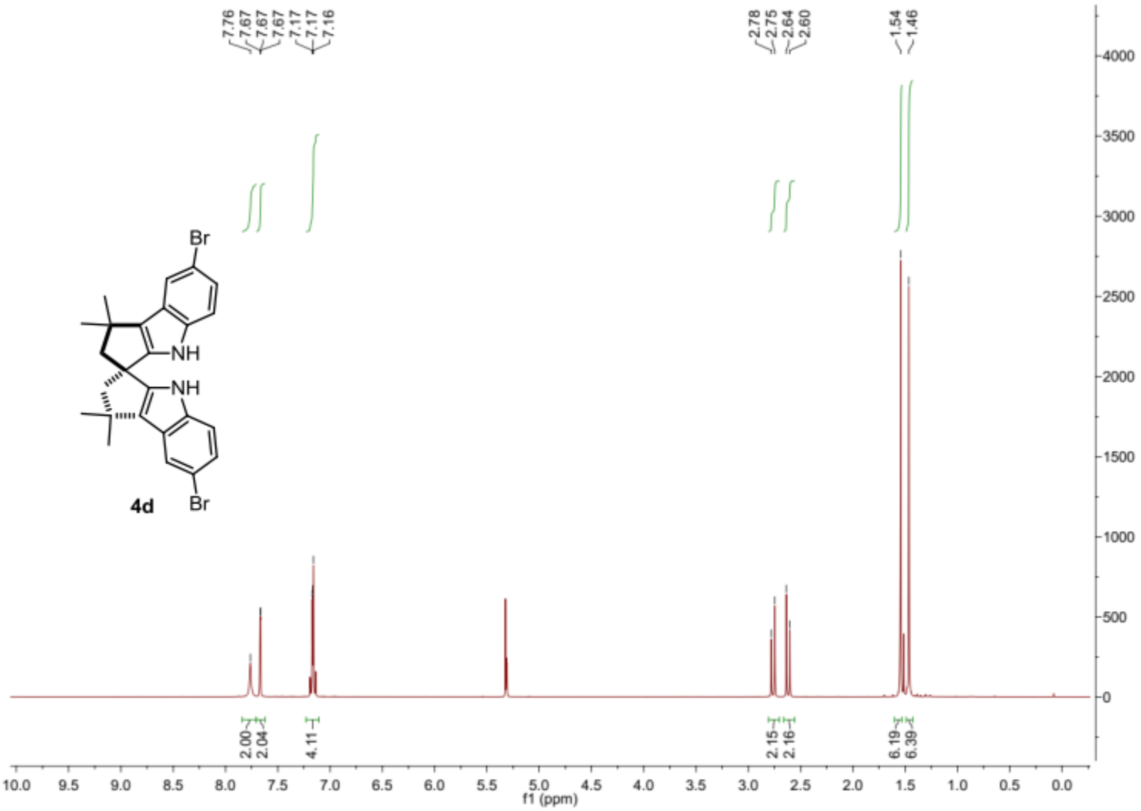

^{1}H NMR (400 MHz, CD_2Cl_2, rt)

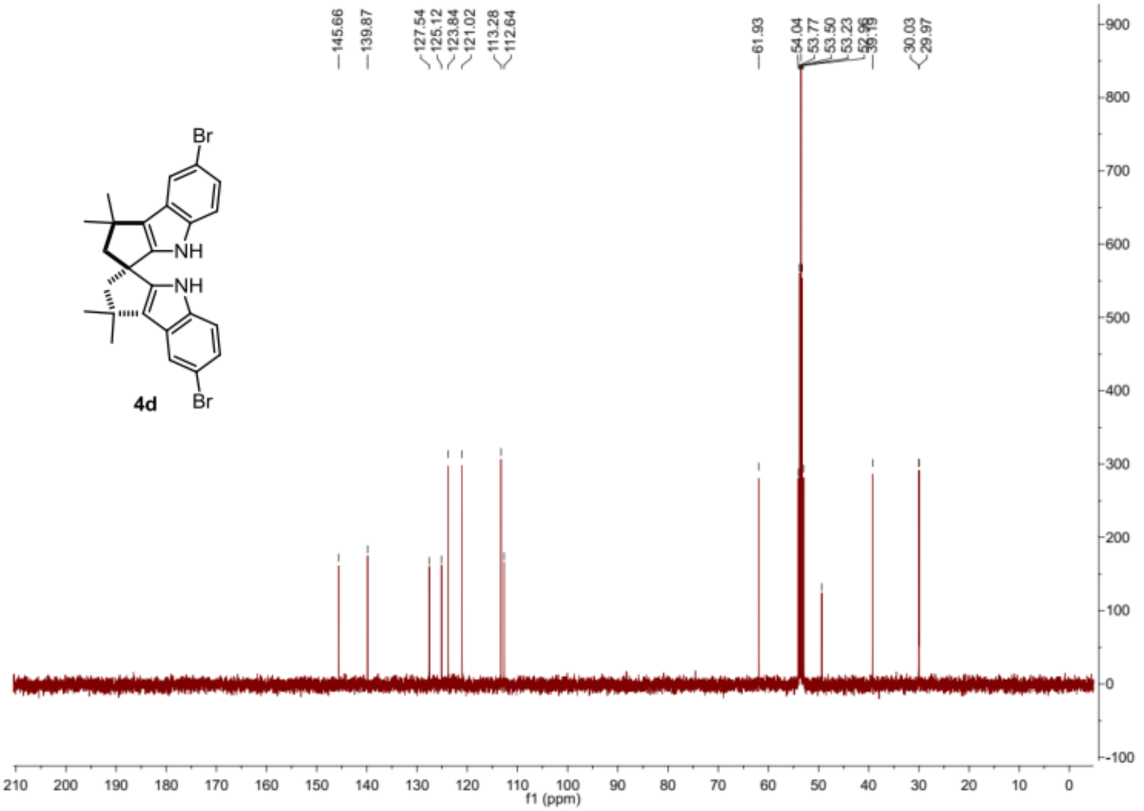

^{13}C NMR (101 MHz, CD_2Cl_2, rt)

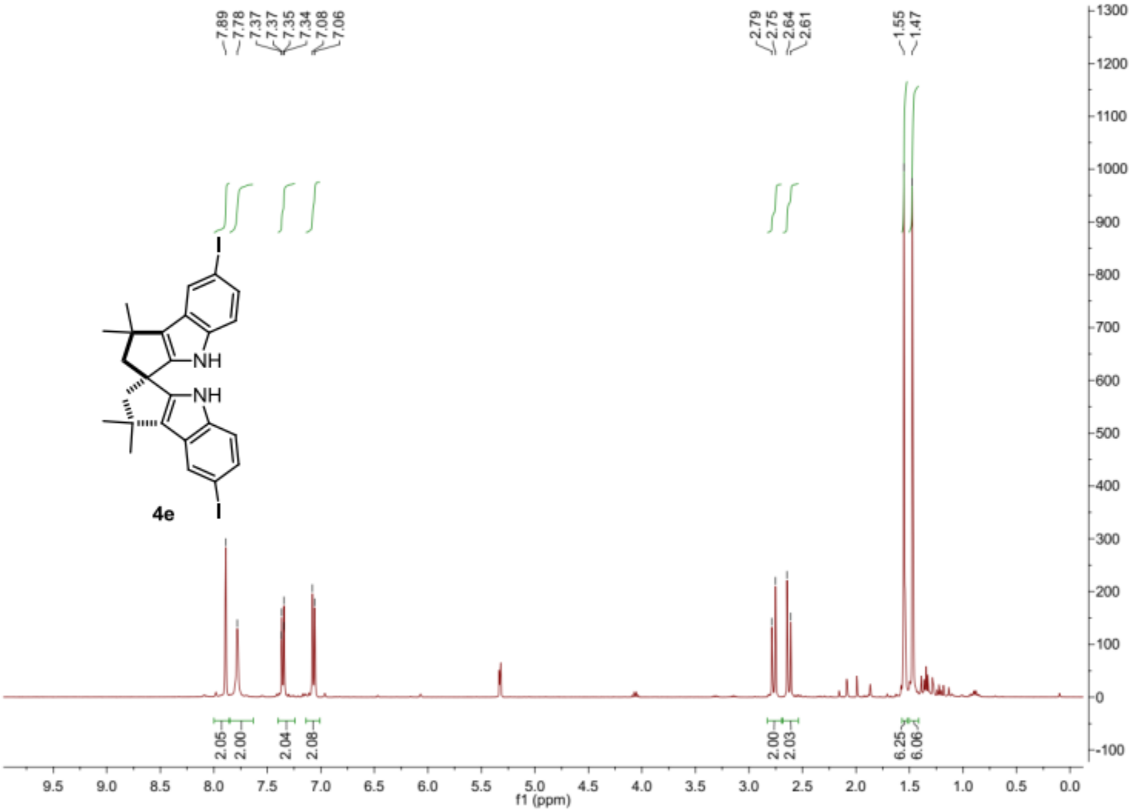

^{1}H NMR (400 MHz, CD_2Cl_2, rt)

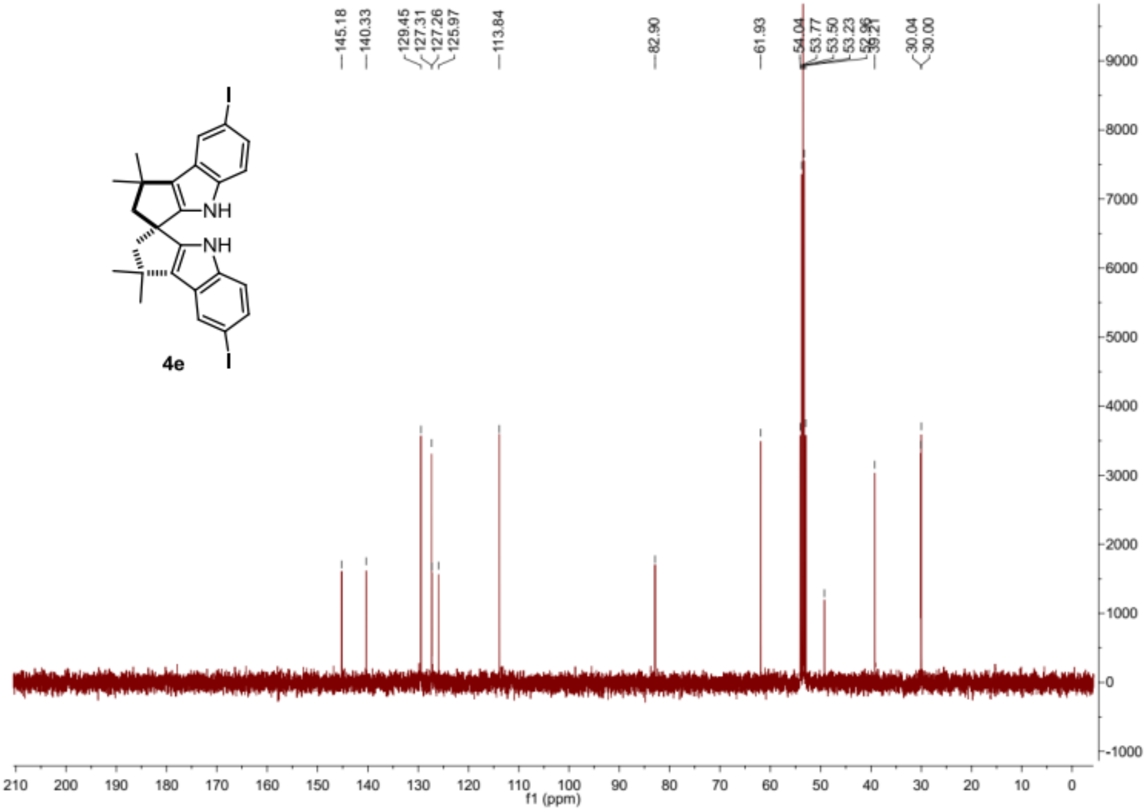

^{13}C NMR (101 MHz, CD_2Cl_2, rt)

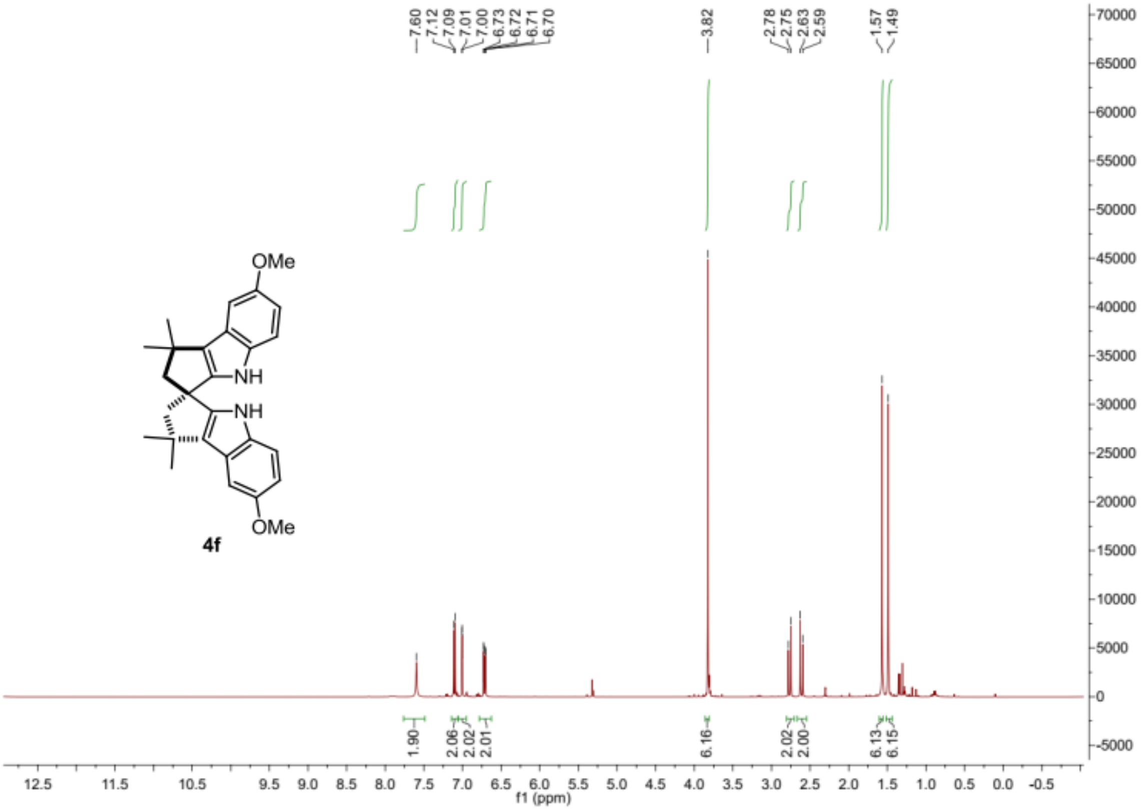

^{1}H NMR (400 MHz, CD_2Cl_2, rt)

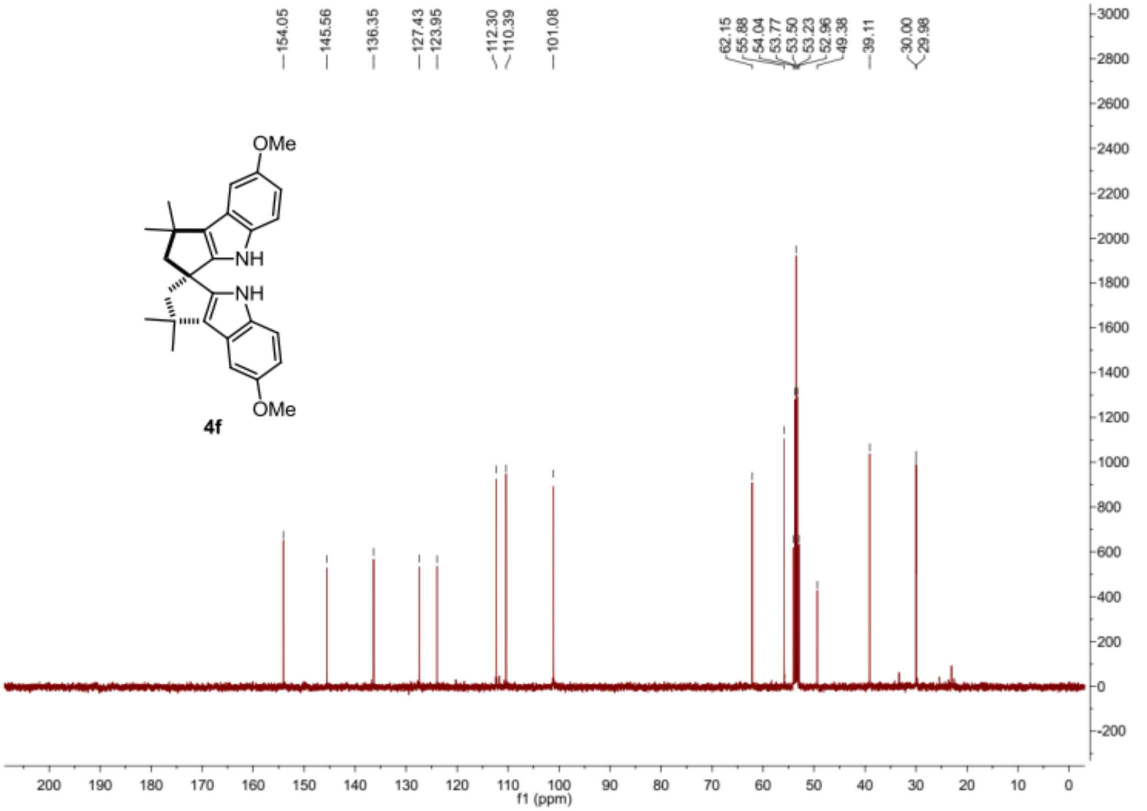

^{13}C NMR (101 MHz, CD_2Cl_2, rt)

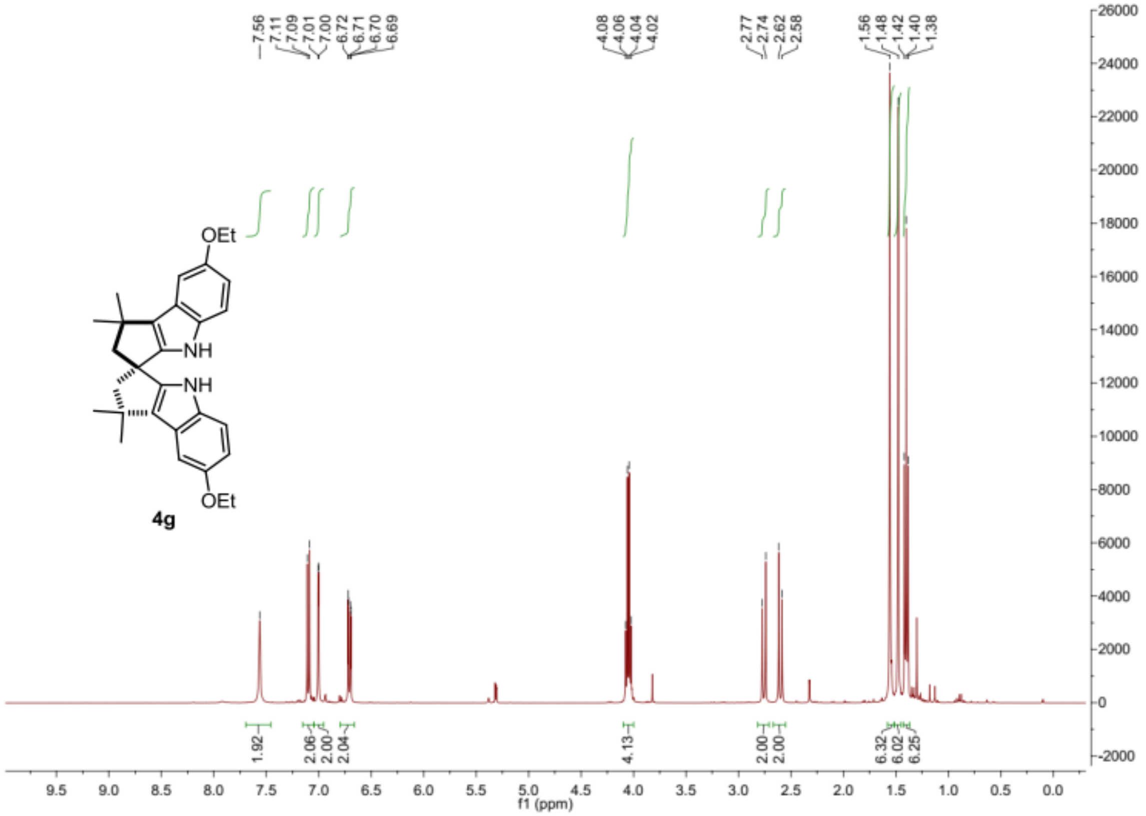

^1H NMR (400 MHz, CD_2Cl_2, rt)

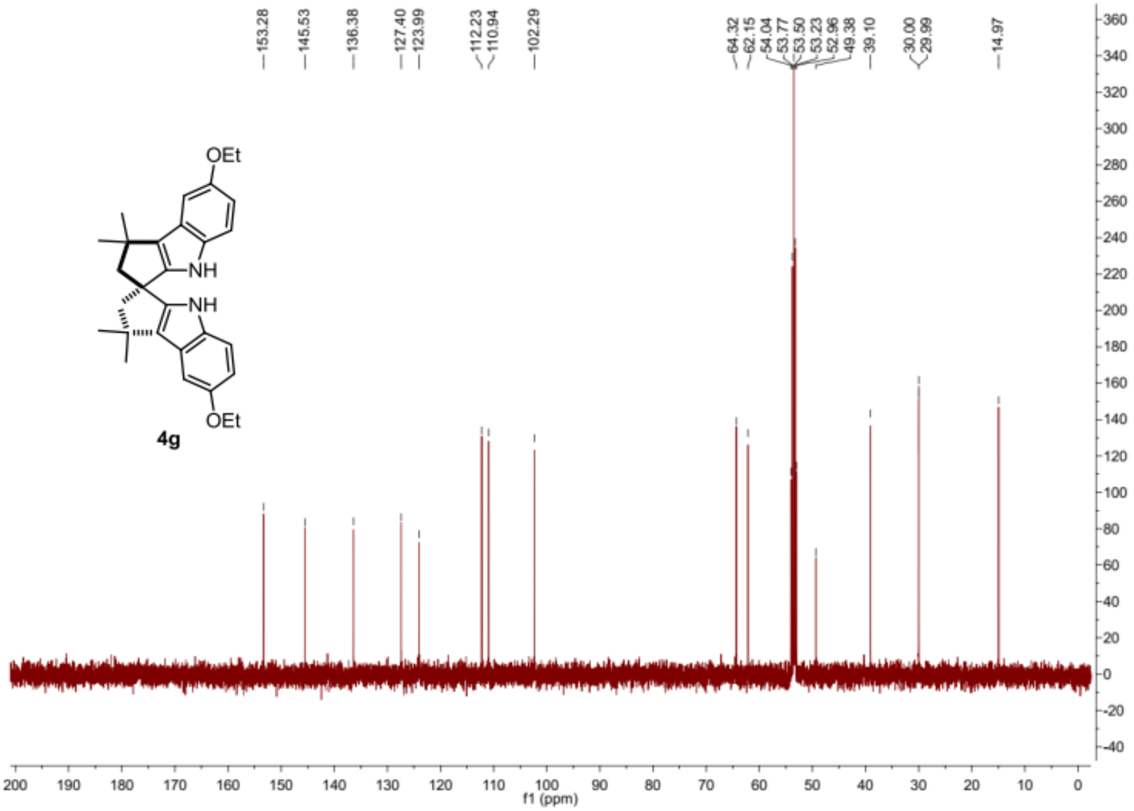

^{13}C NMR (101 MHz, CD_2Cl_2, rt)

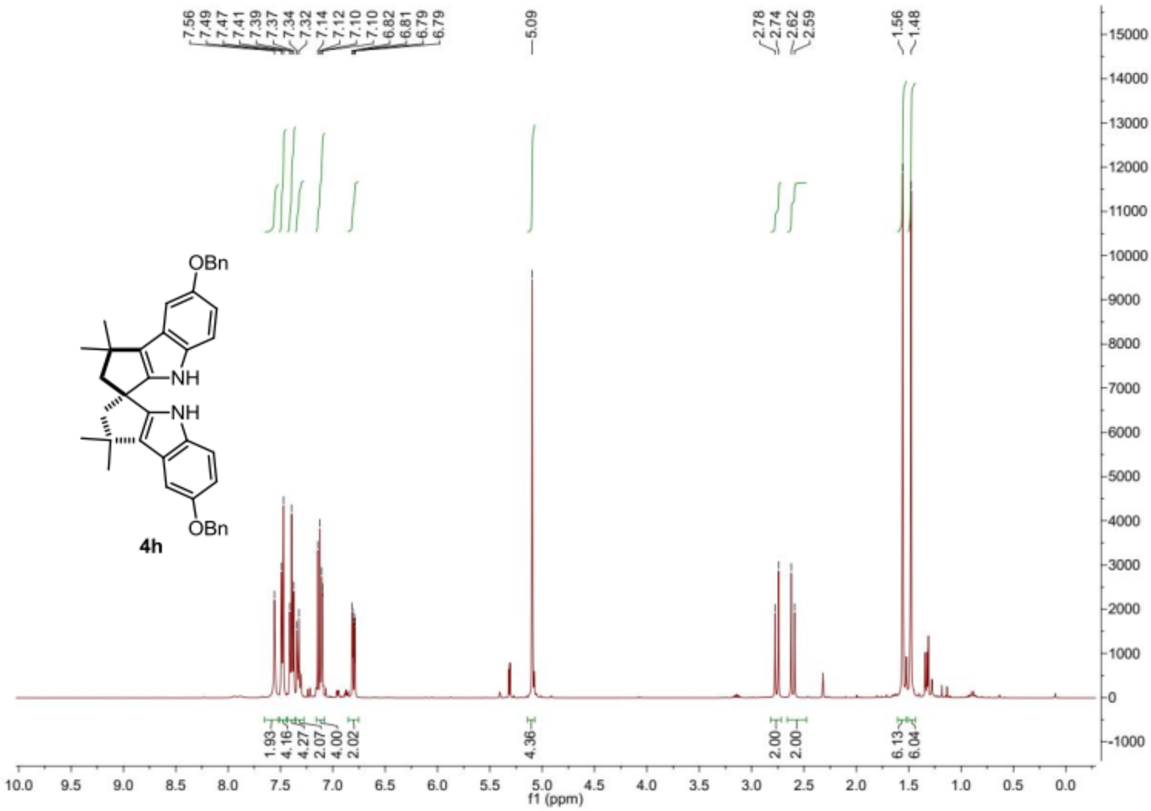

^1H NMR (400 MHz, CD_2Cl_2, rt)

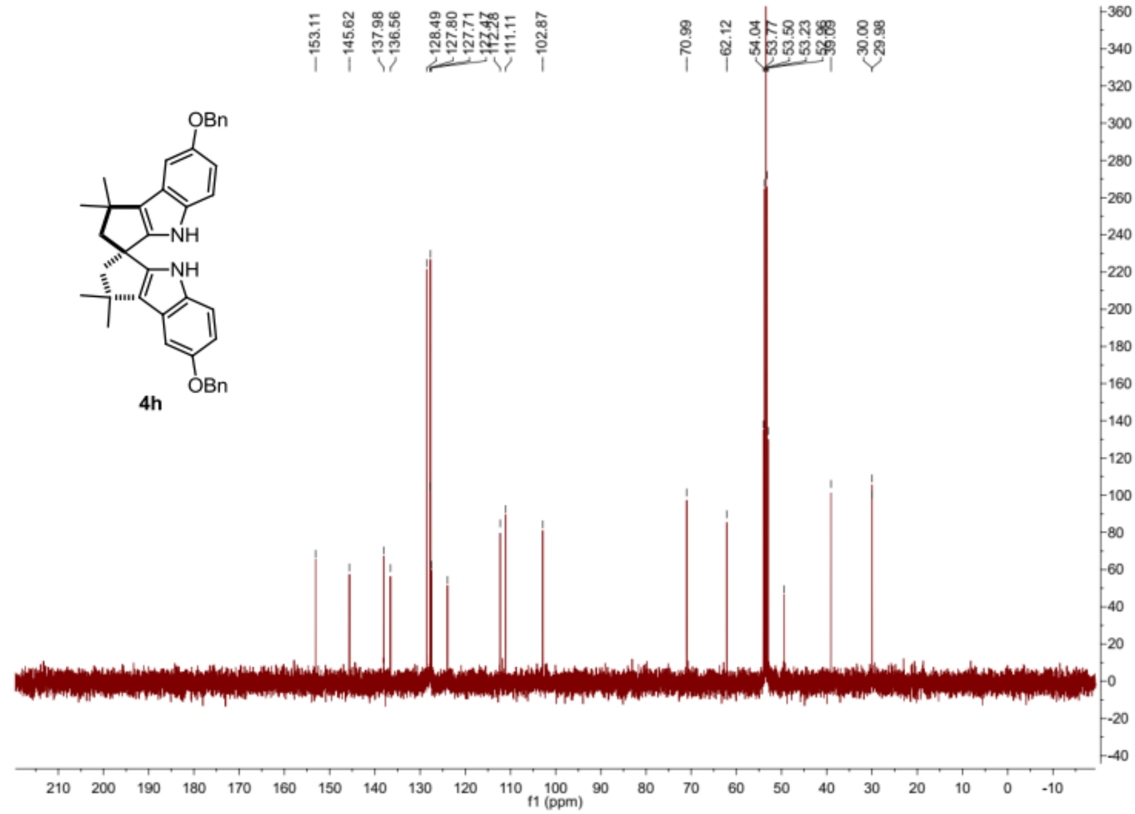

^{13}C NMR (101 MHz, CD_2Cl_2, rt)

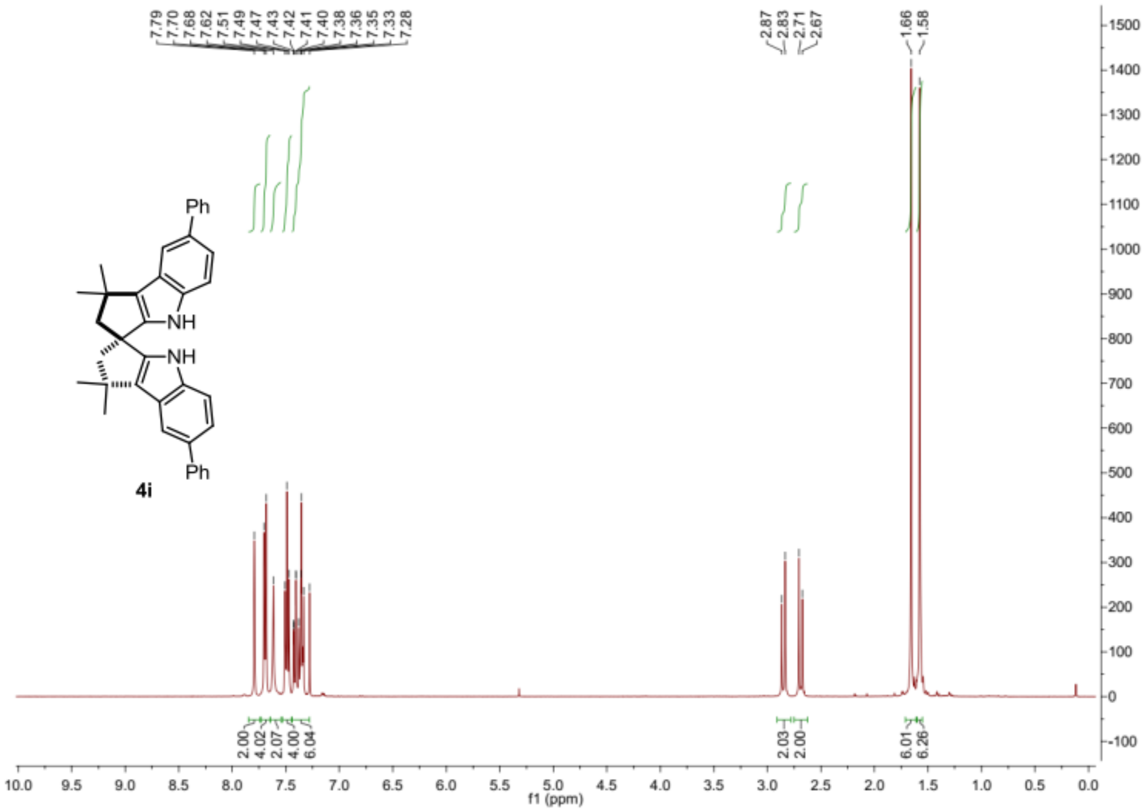

^{1}H NMR (400 MHz, $CDCl_3$, rt)

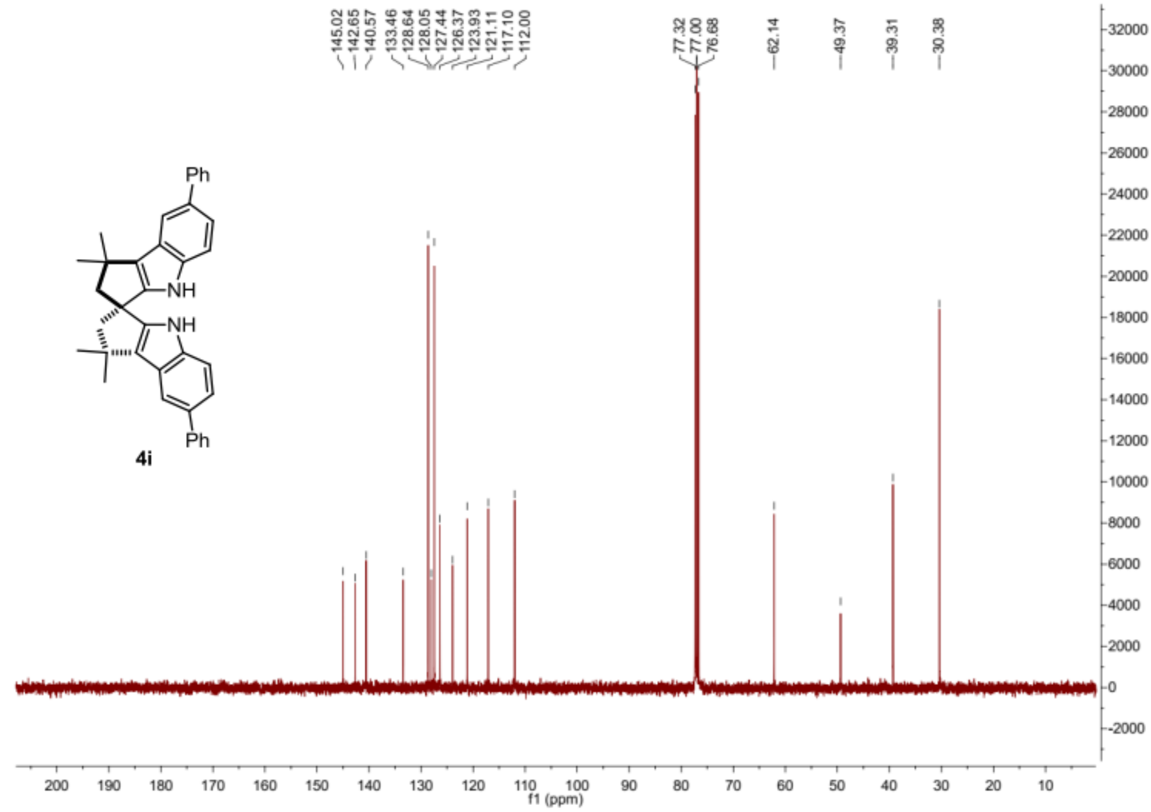

^{13}C NMR (101 MHz, $CDCl_3$, rt)

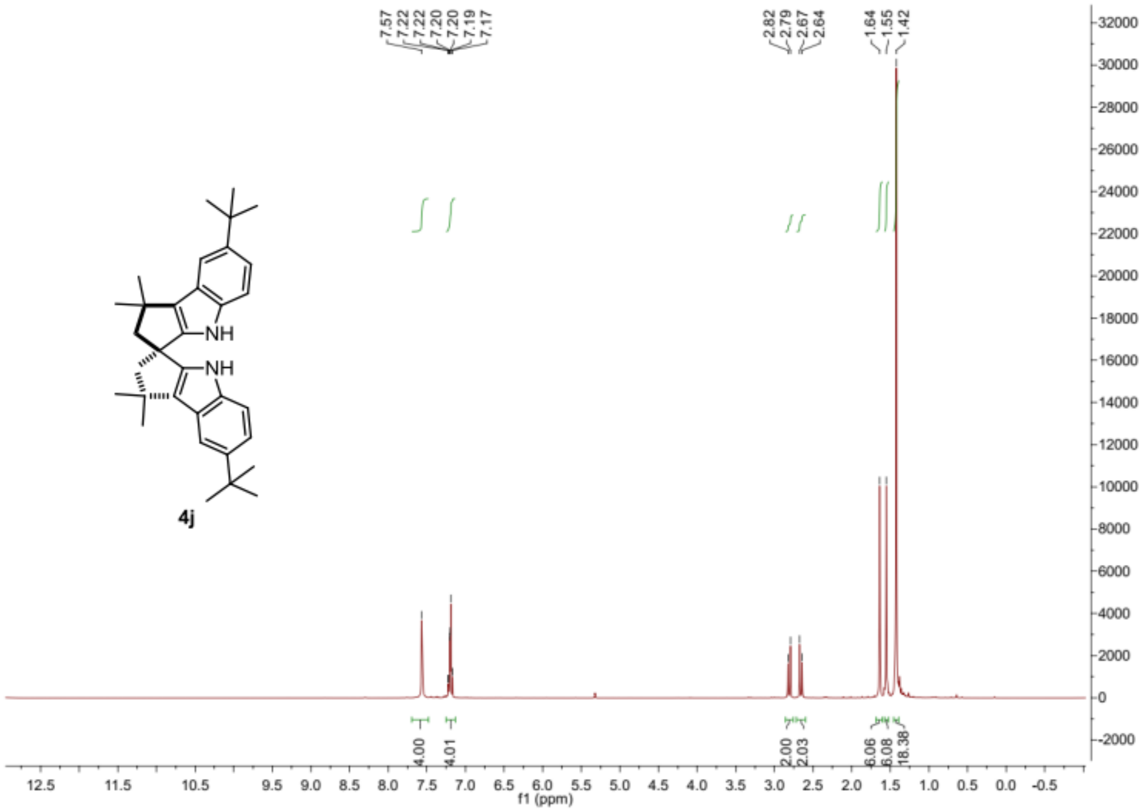

^{1}H NMR (400 MHz, CD_2Cl_2, rt)

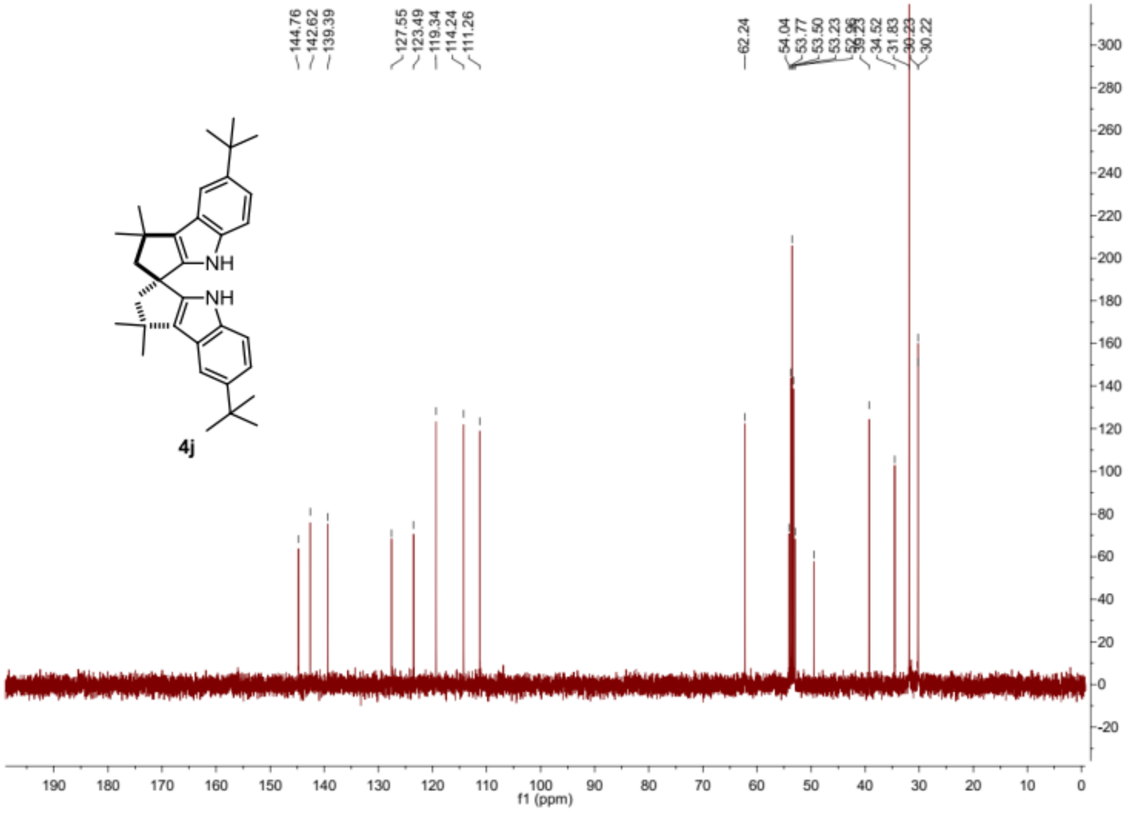

^{13}C NMR (101 MHz, CD_2Cl_2, rt)

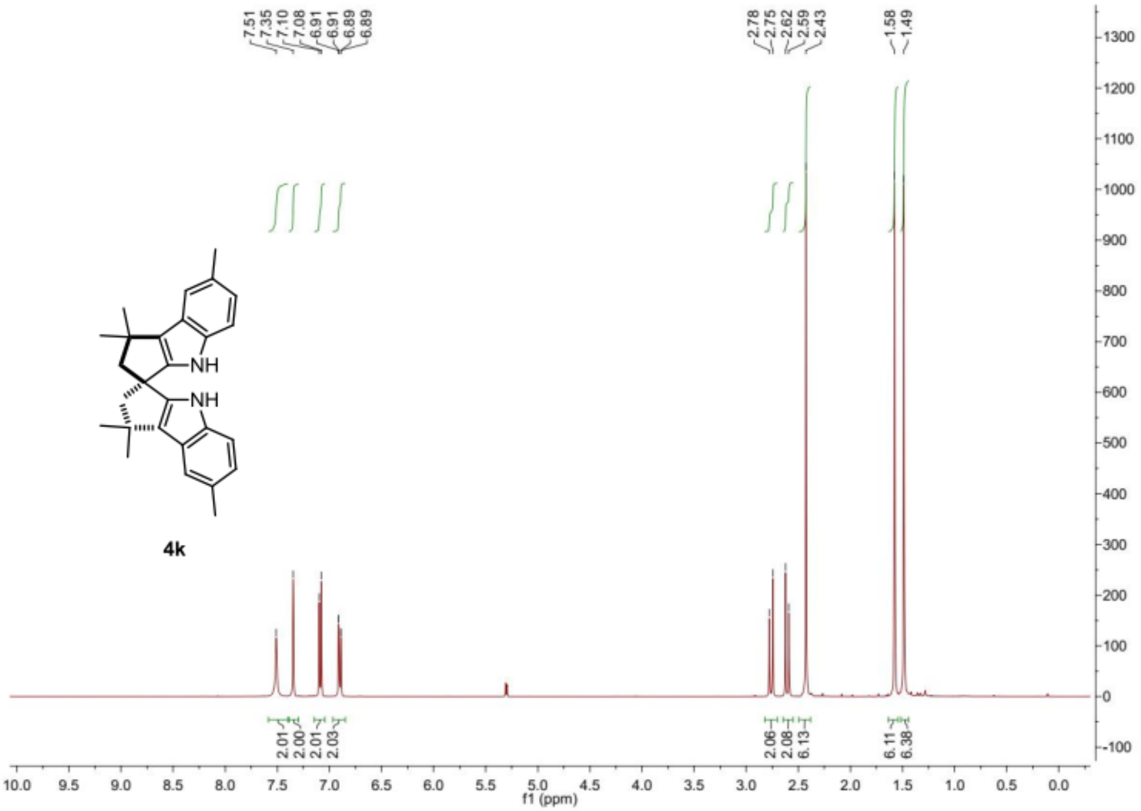

^{1}H NMR (400 MHz, CD_2Cl_2, rt)

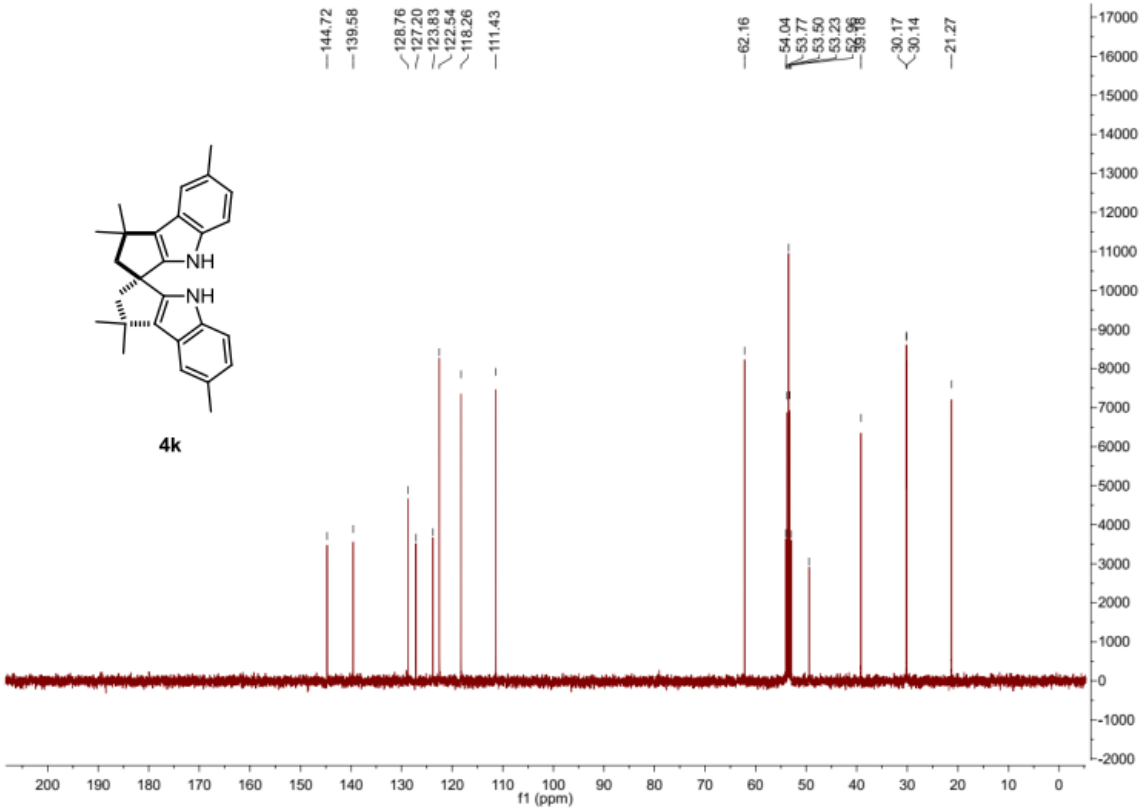

^{13}C NMR (101 MHz, CD_2Cl_2, rt)

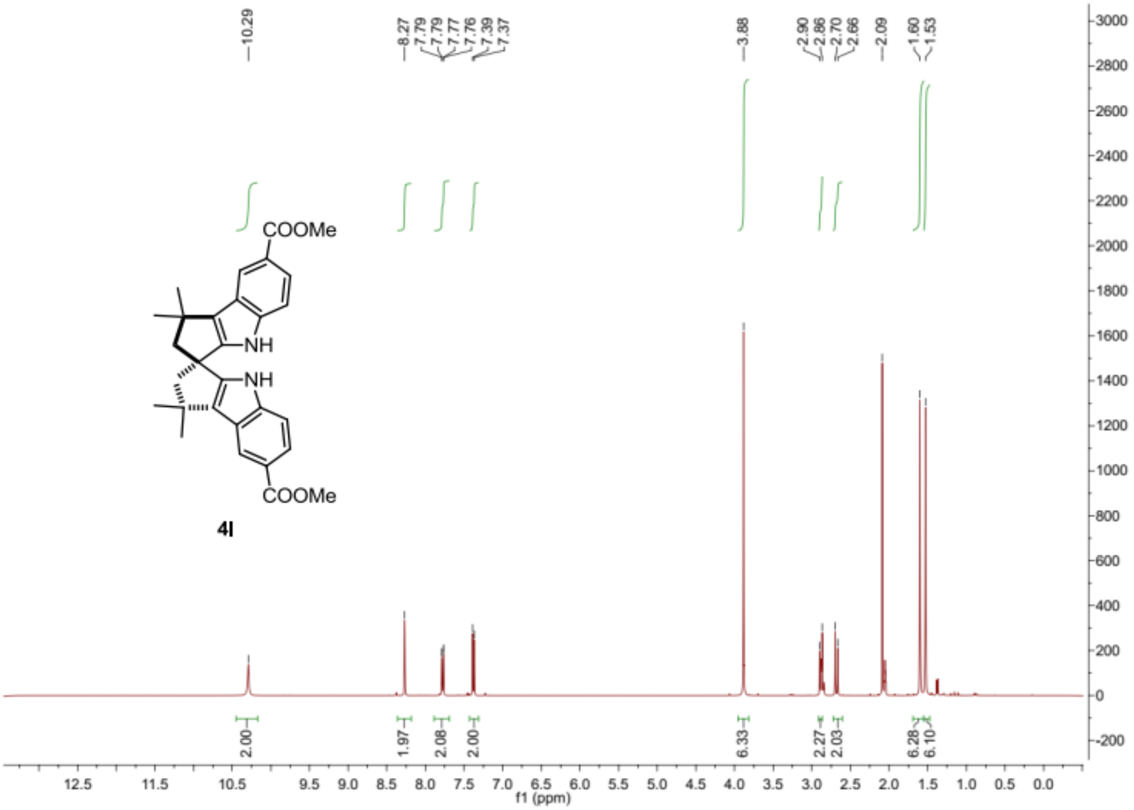

^{1}H NMR (400 MHz, Acetone-D_6, rt)

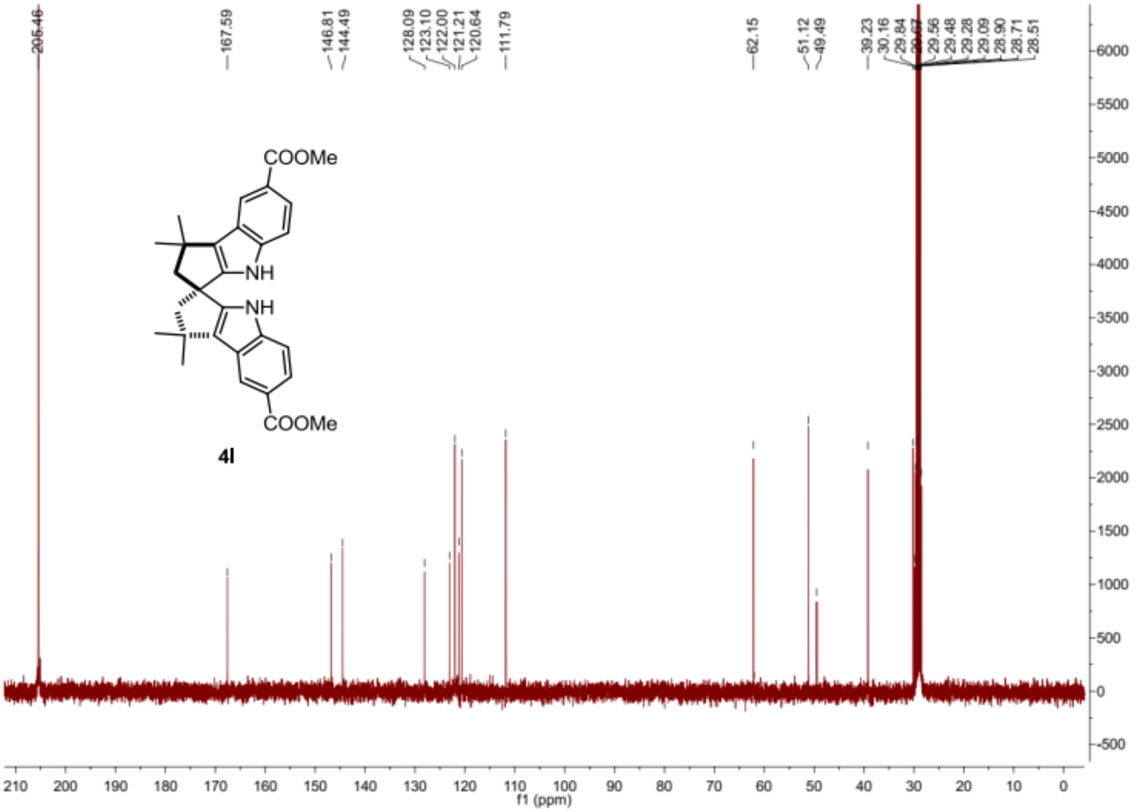

^{13}C NMR (101 MHz, Acetone-D_6, rt)

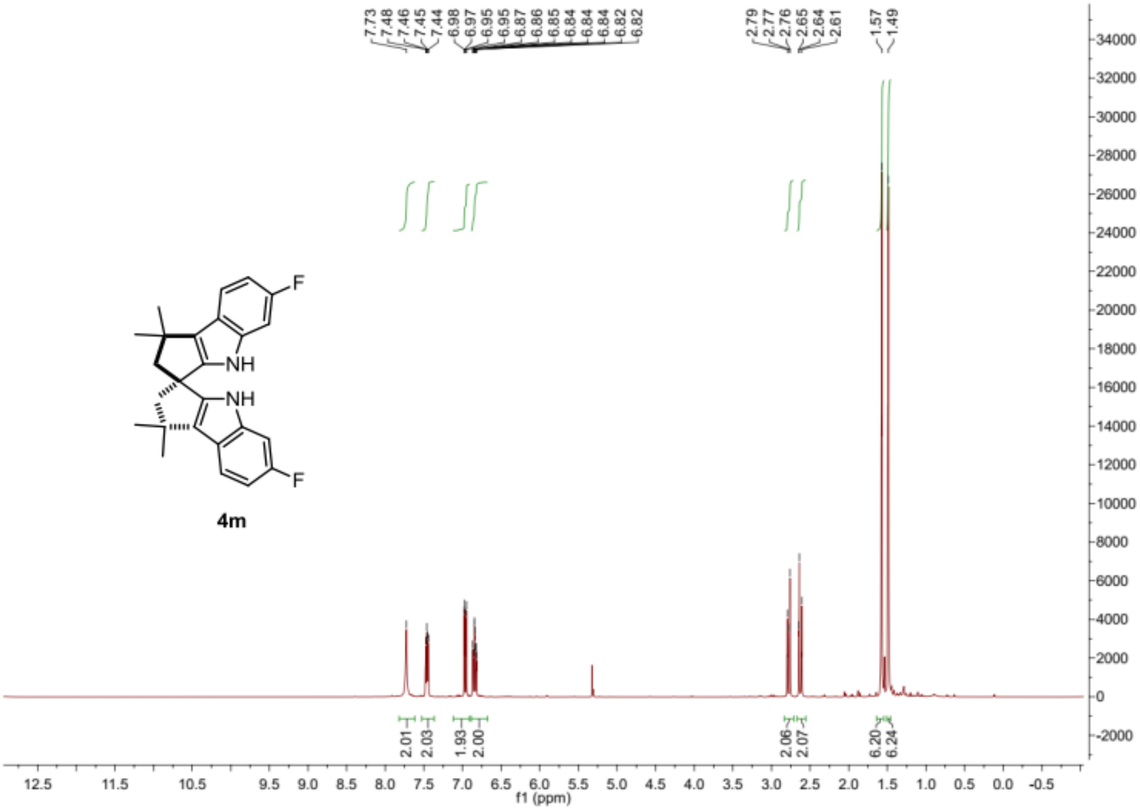

^{1}H NMR (400 MHz, CD_2Cl_2, rt)

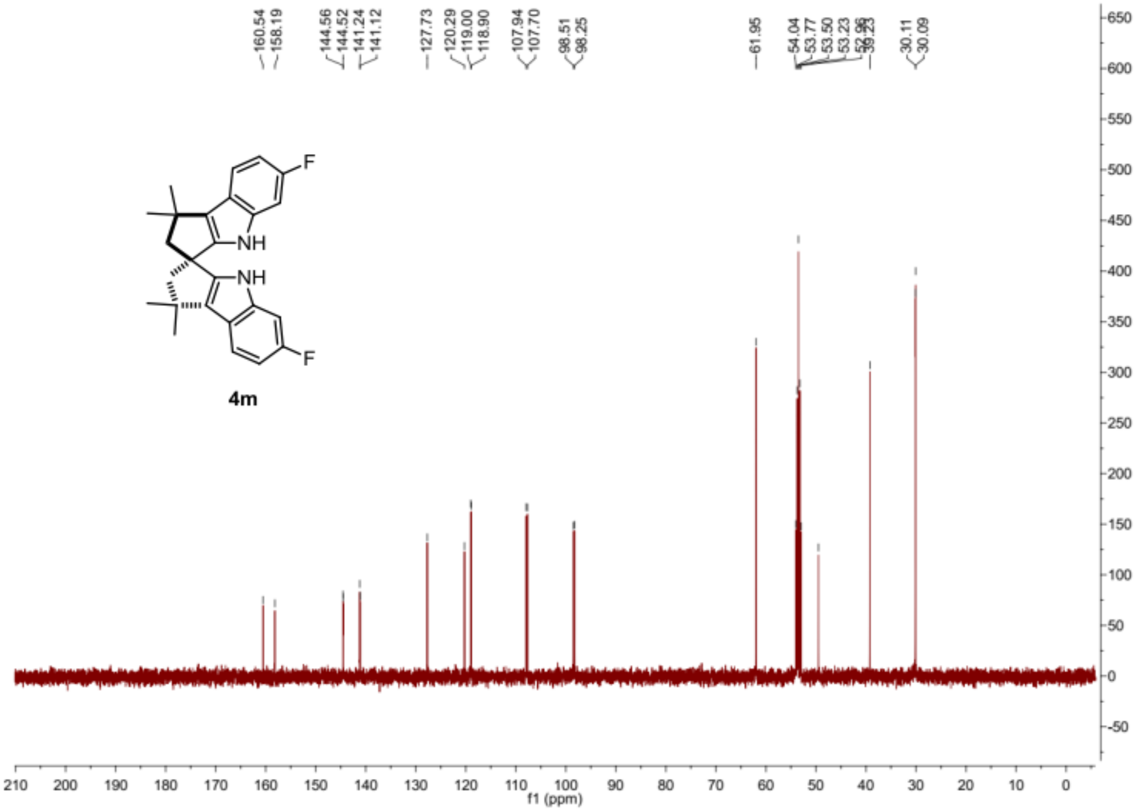

^{13}C NMR (101 MHz, CD_2Cl_2, rt)

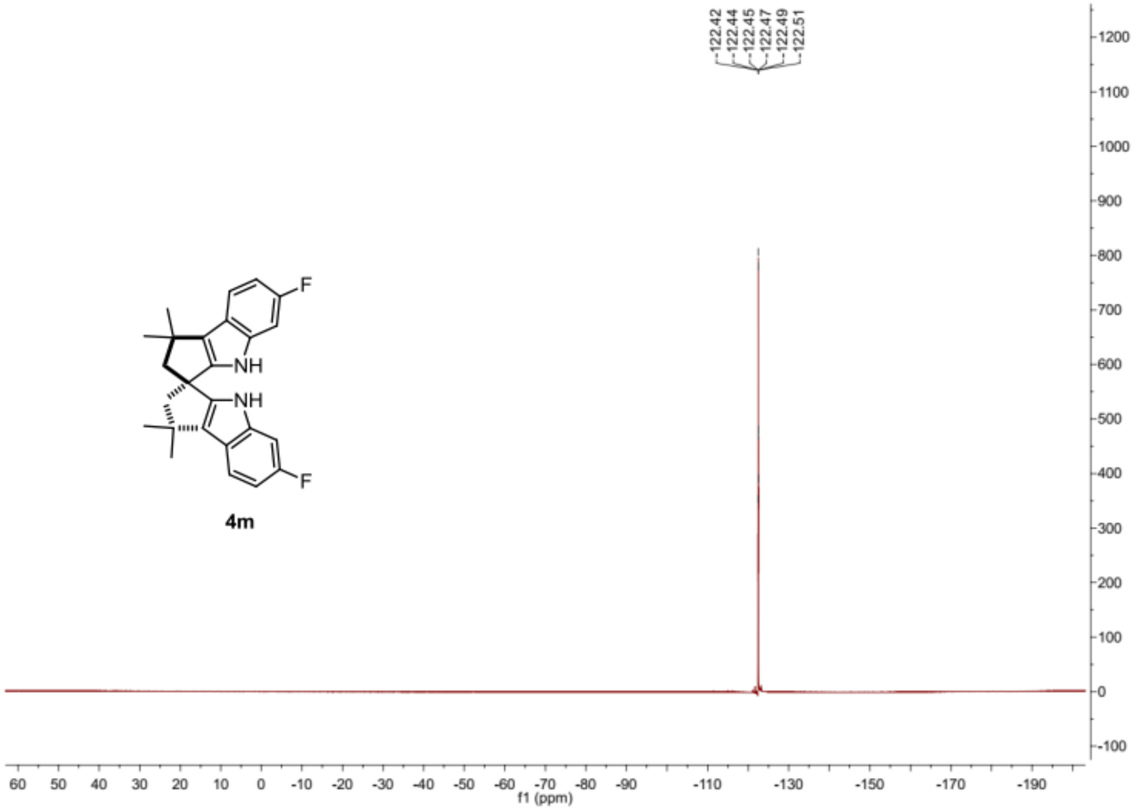

^{19}F NMR (282 MHz, CD_2Cl_2, rt)

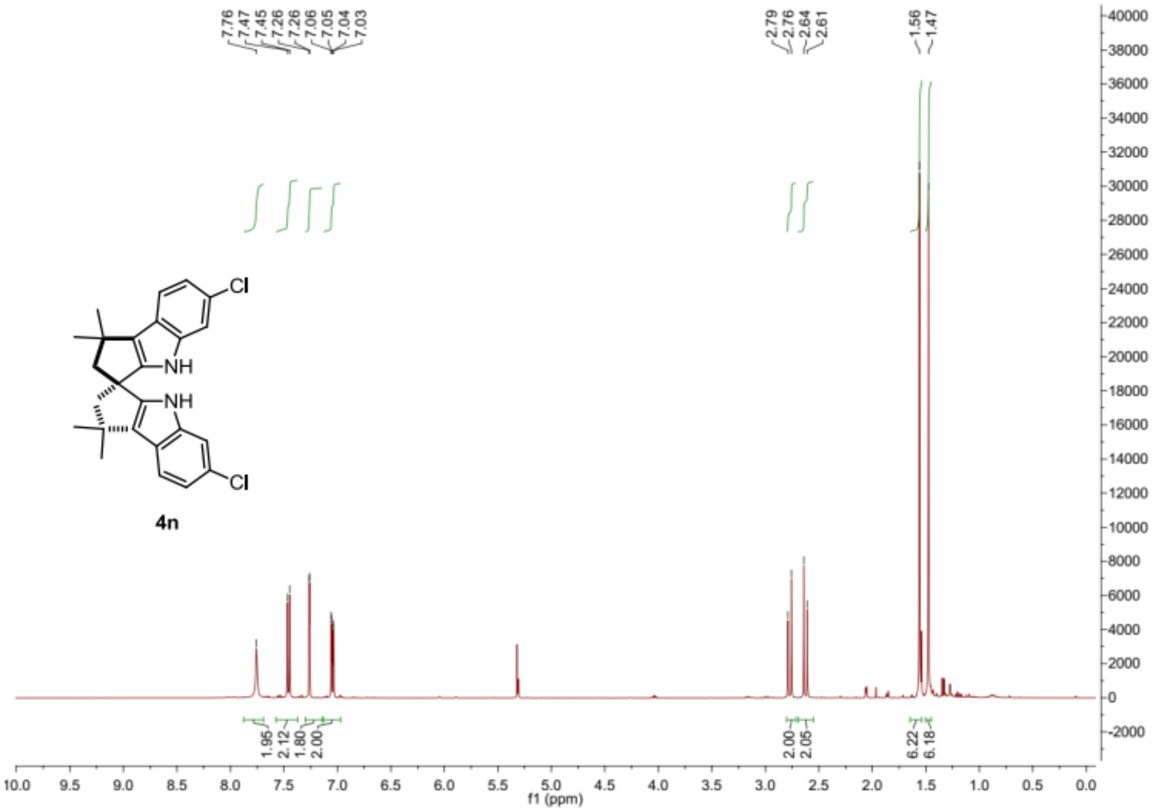

^1H NMR (400 MHz, CD_2Cl_2, rt)

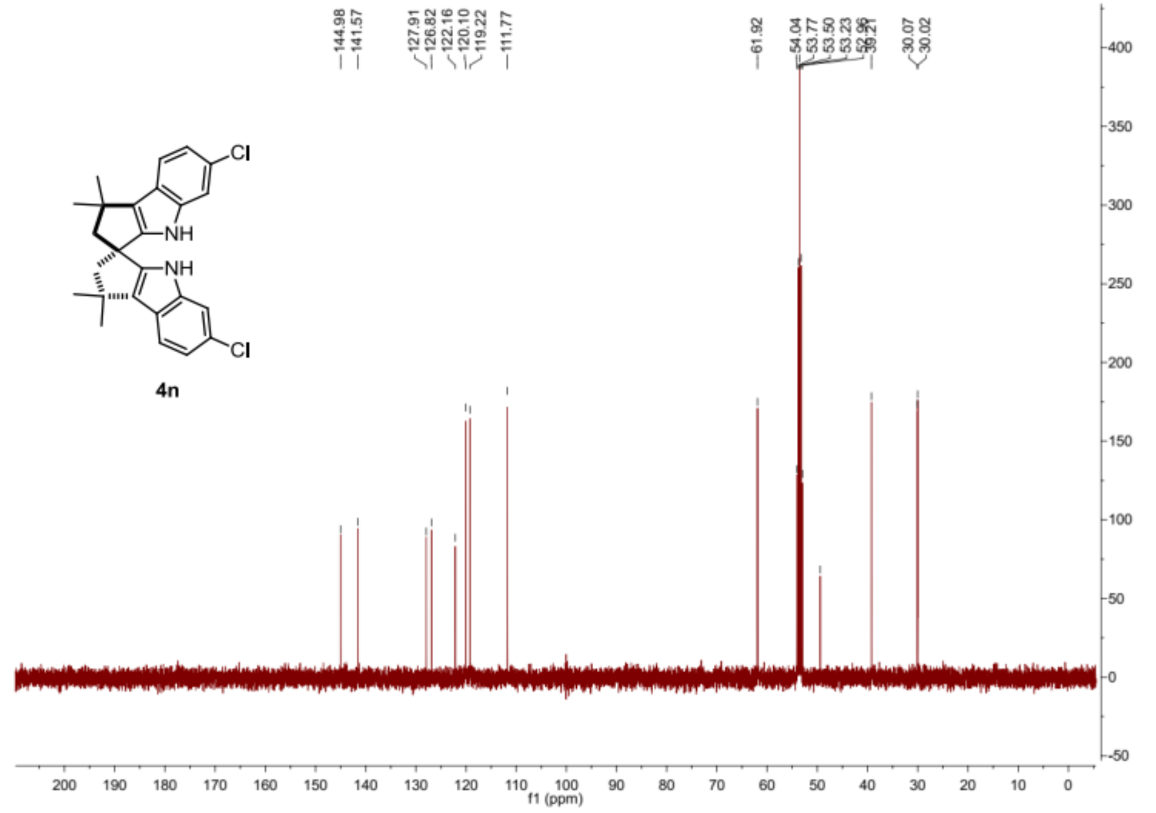

^{13}C NMR (101 MHz, CD_2Cl_2, rt)

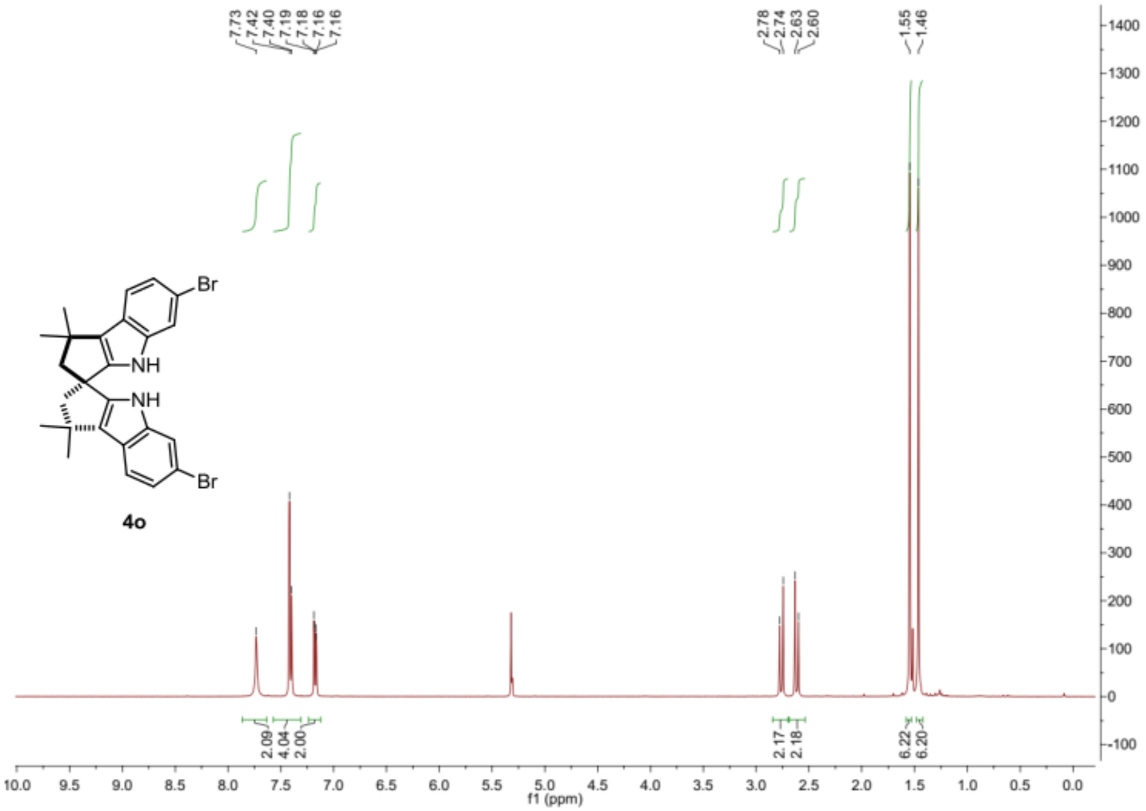

^{1}H NMR (400 MHz, CD_2Cl_2, rt)

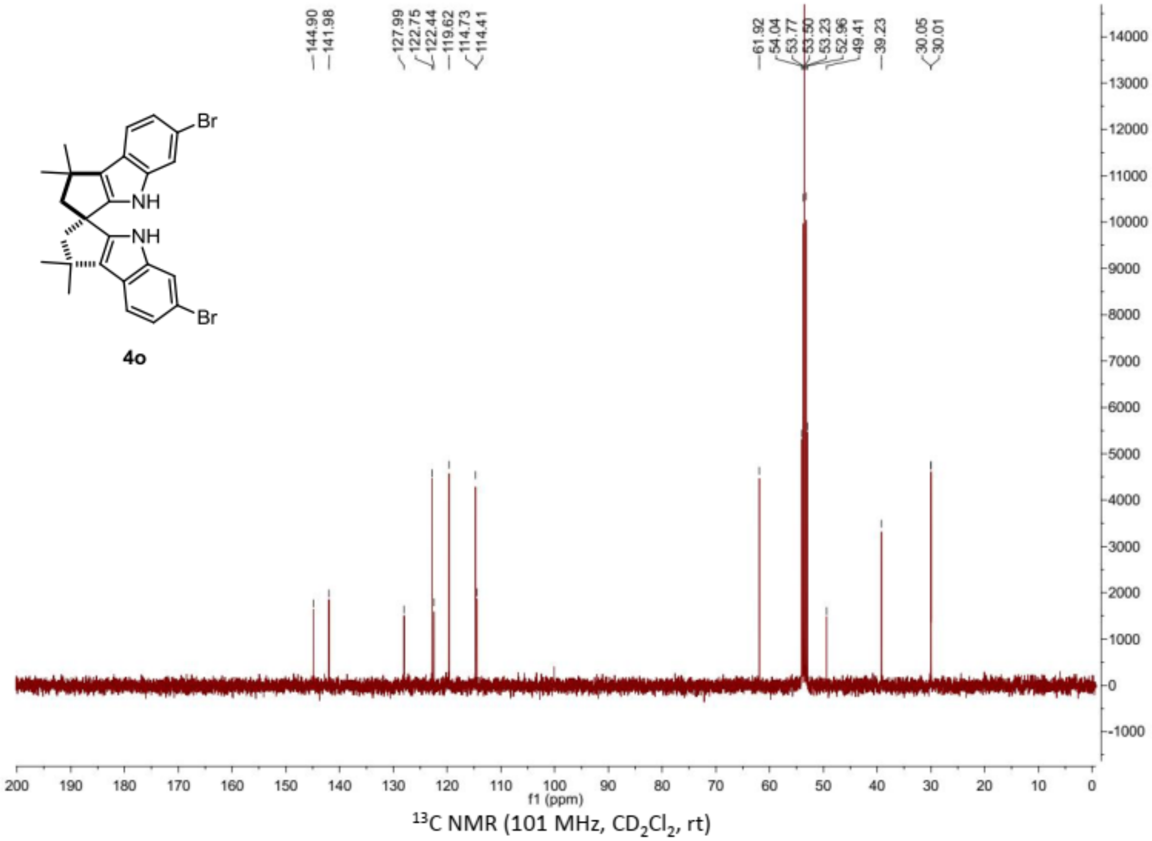

^{13}C NMR (101 MHz, CD_2Cl_2, rt)

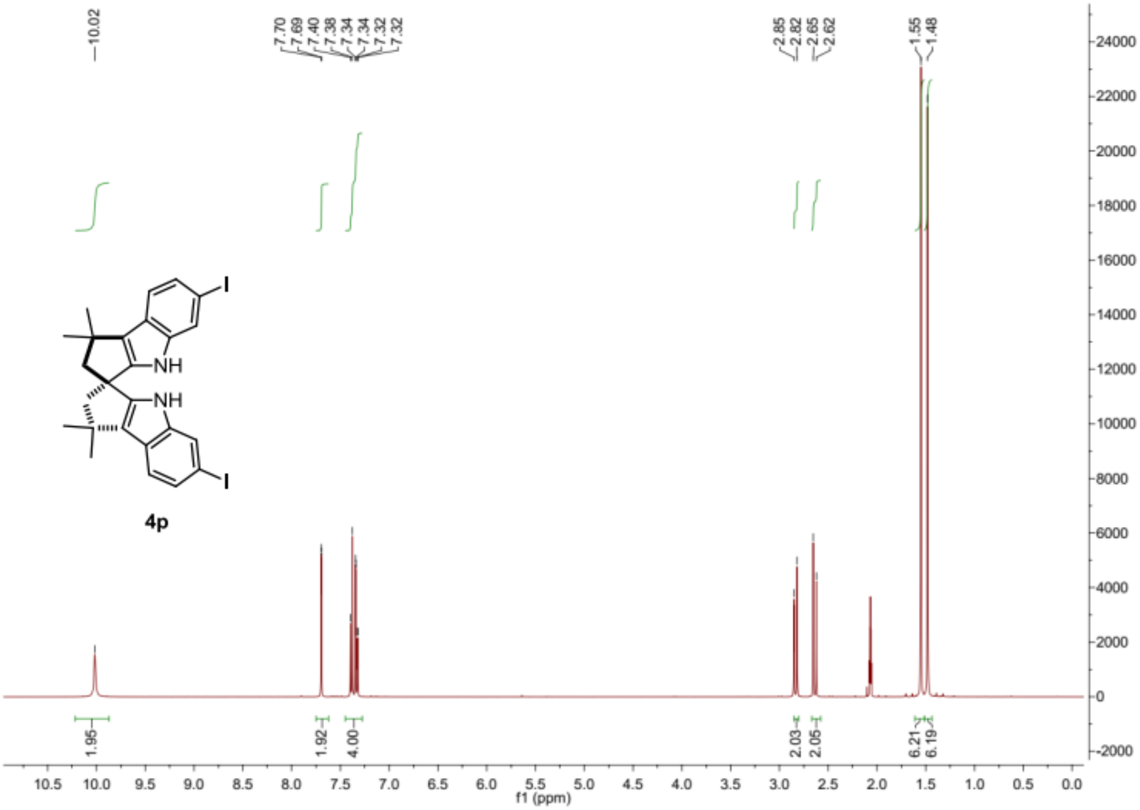

^{1}H NMR (400 MHz, Acetone-D_6, rt)

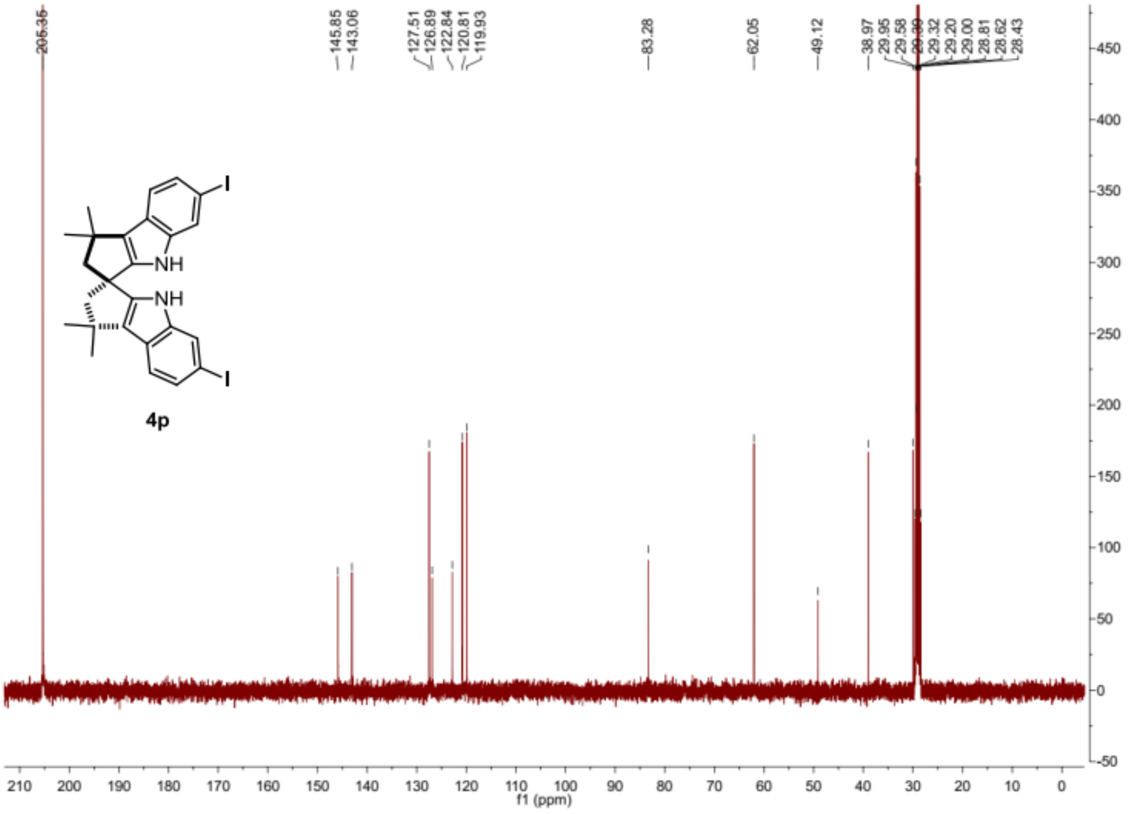

^{13}C NMR (101 MHz, Acetone-D_6, rt)

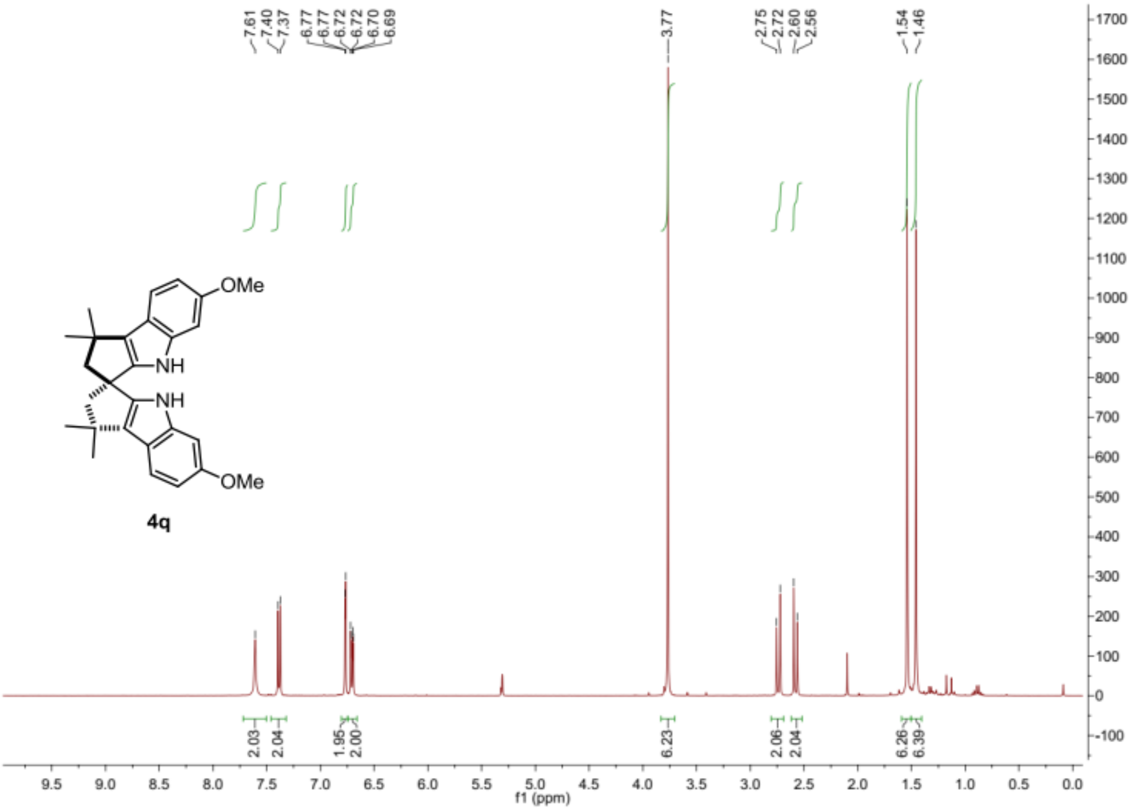

^{1}H NMR (400 MHz, CD_2Cl_2, rt)

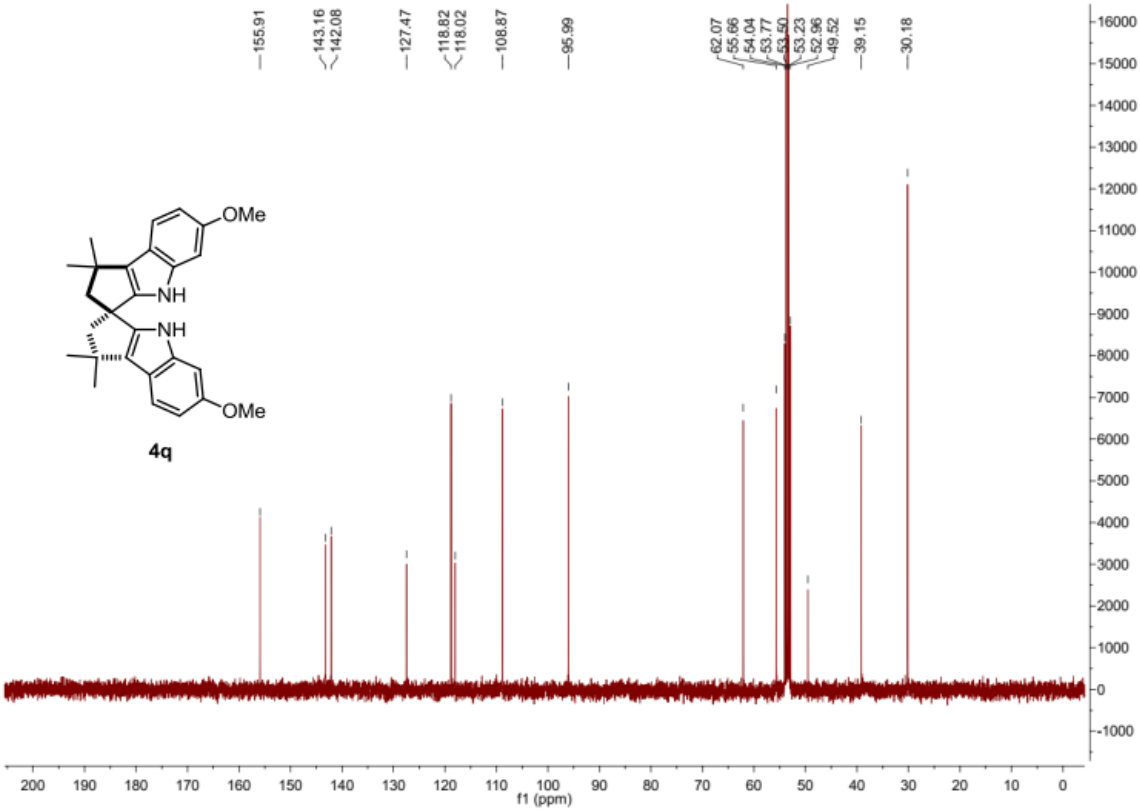

^{13}C NMR (101 MHz, CD_2Cl_2, rt)

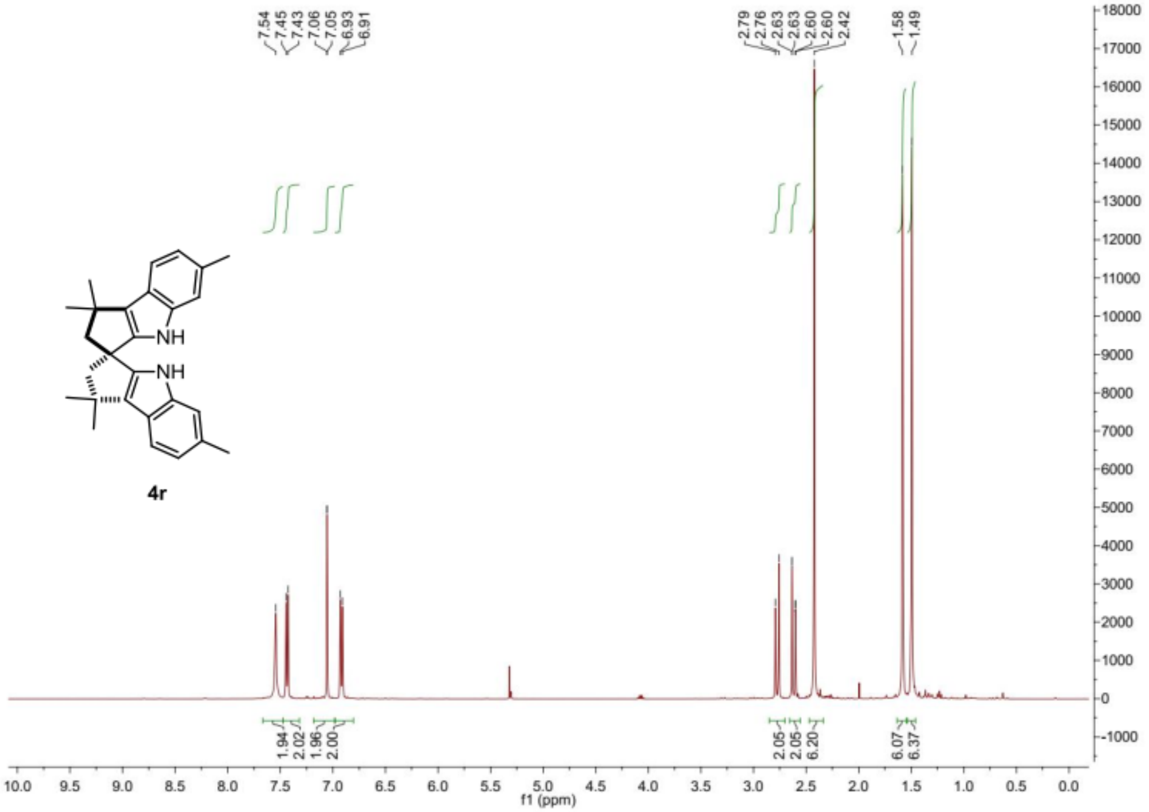

^{1}H NMR (400 MHz, CD_2Cl_2, rt)

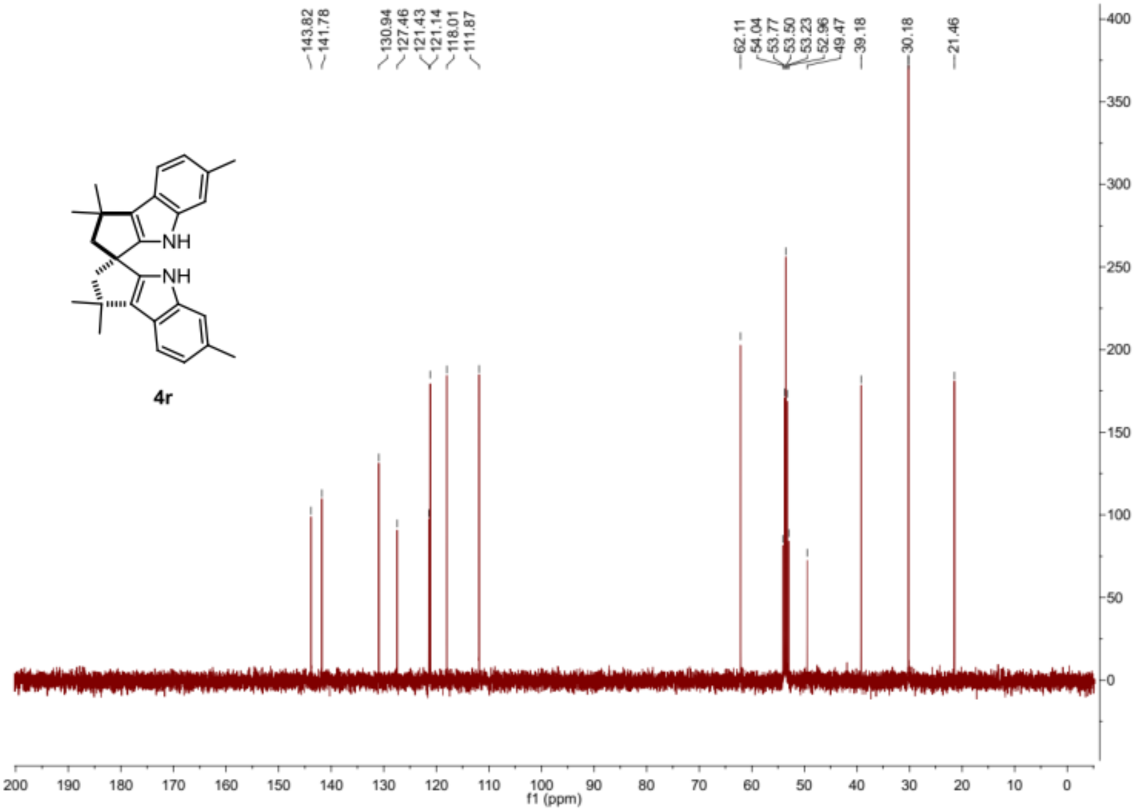

^{13}C NMR (101 MHz, CD_2Cl_2, rt)

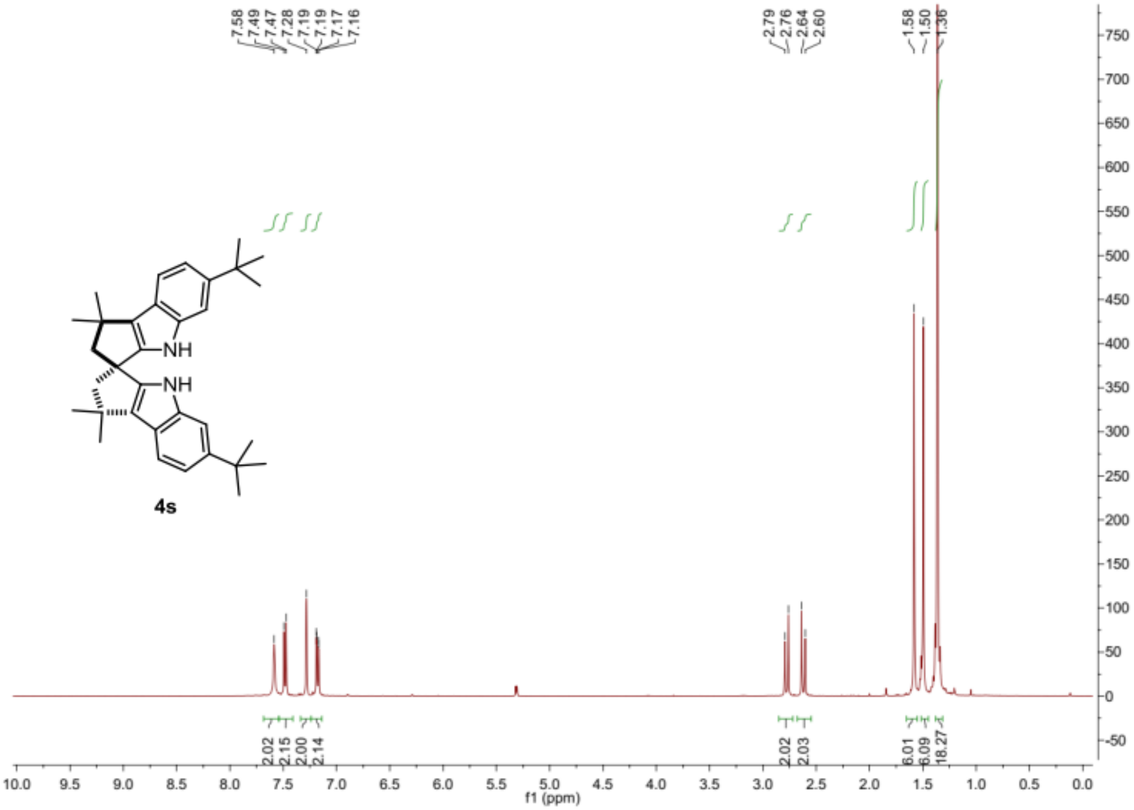

^{1}H NMR (400 MHz, CD_2Cl_2, rt)

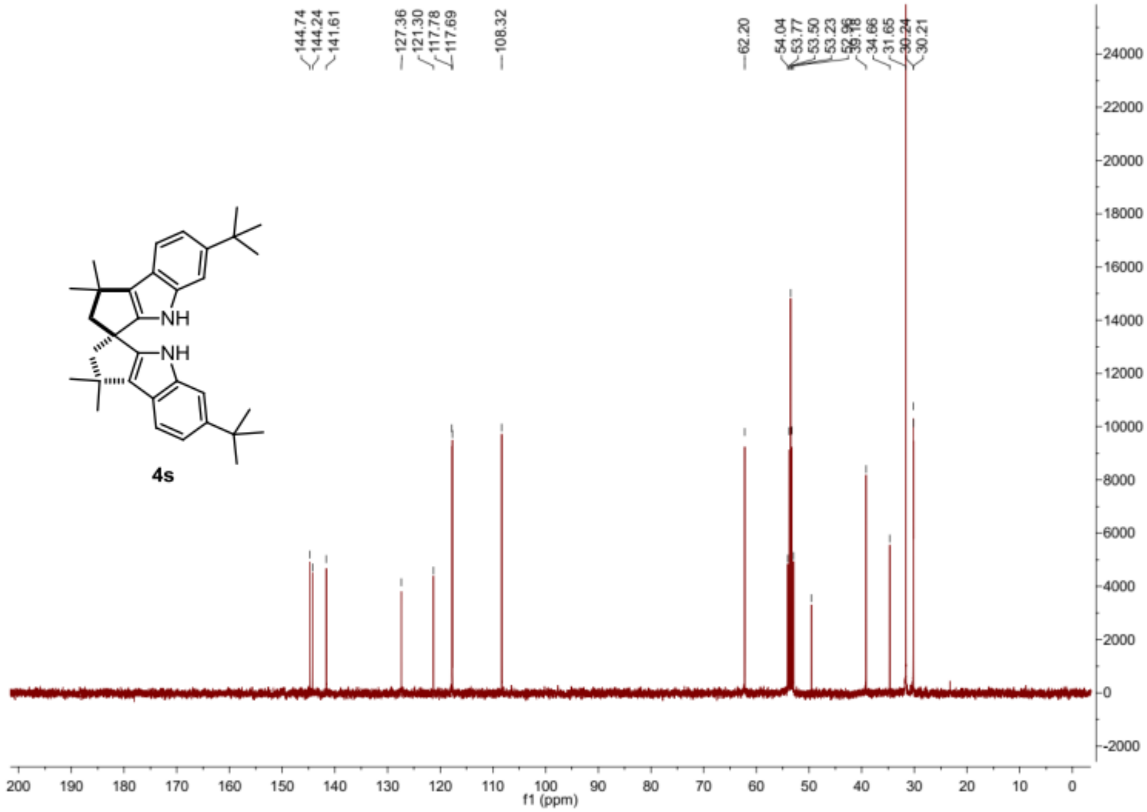

^{13}C NMR (101 MHz, CD_2Cl_2, rt)

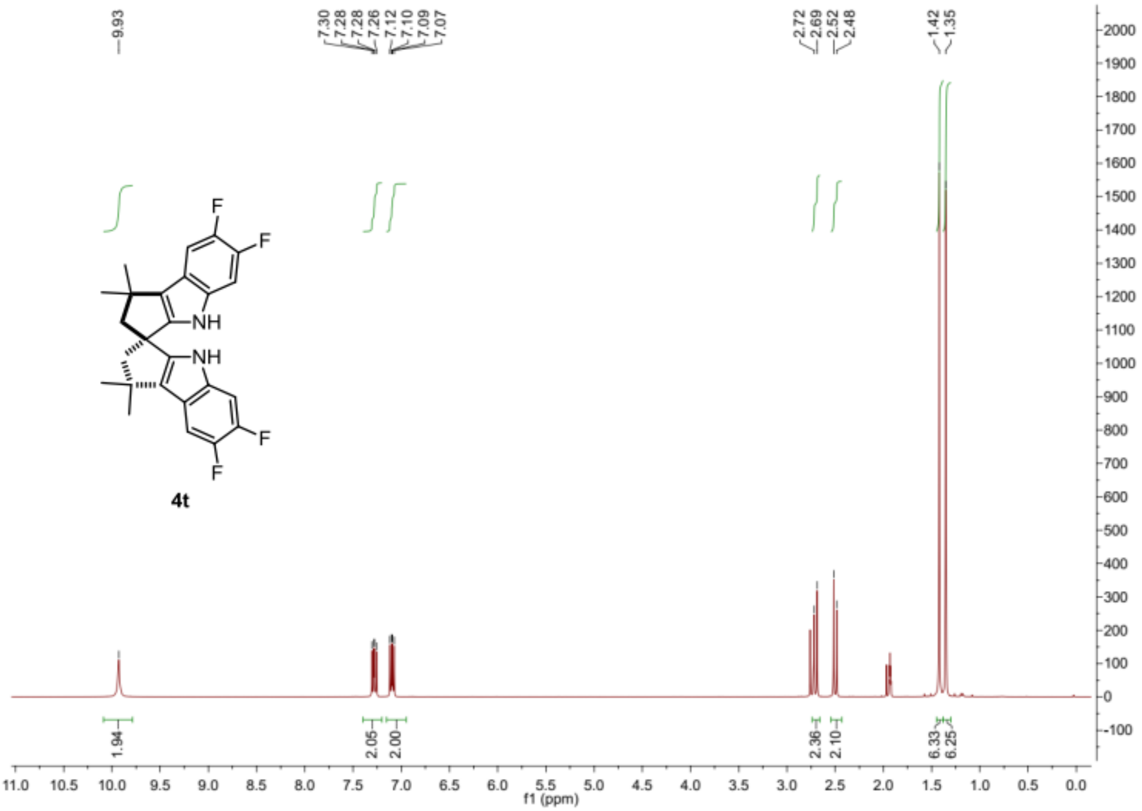

^1H NMR (400 MHz, Acetone-D_6, rt)

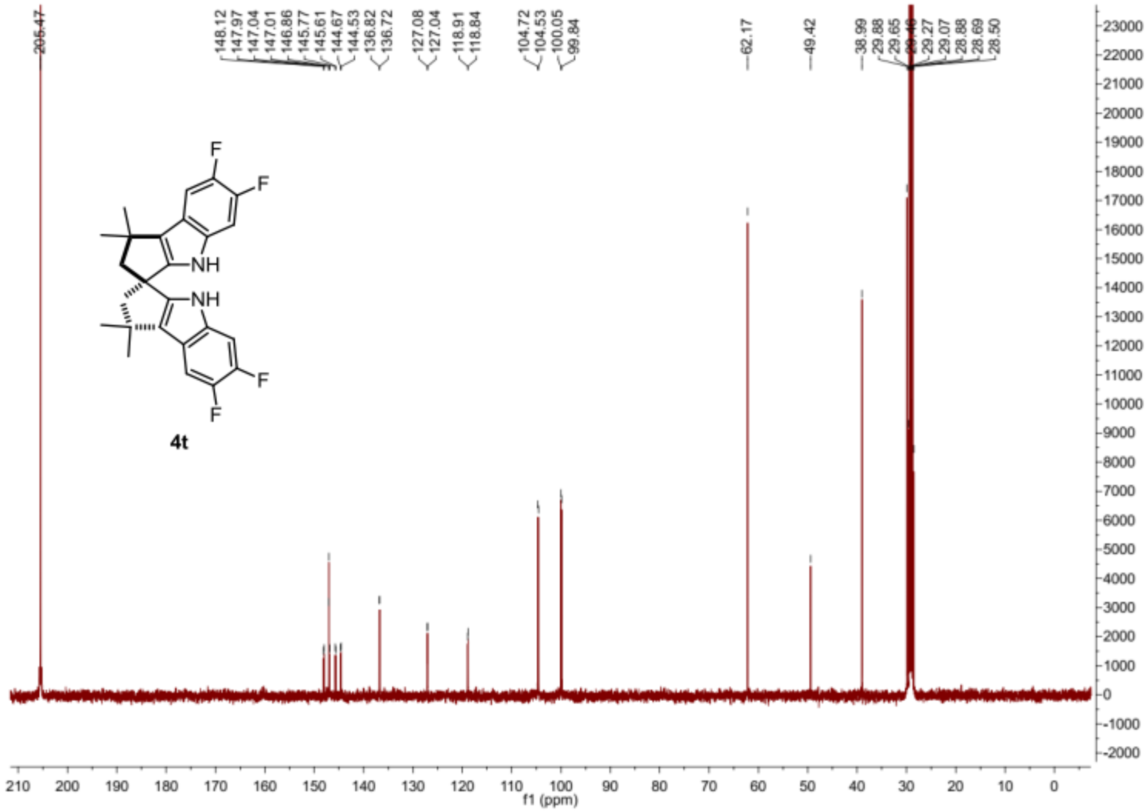

^{13}C NMR (101 MHz, Acetone-D_6, rt)

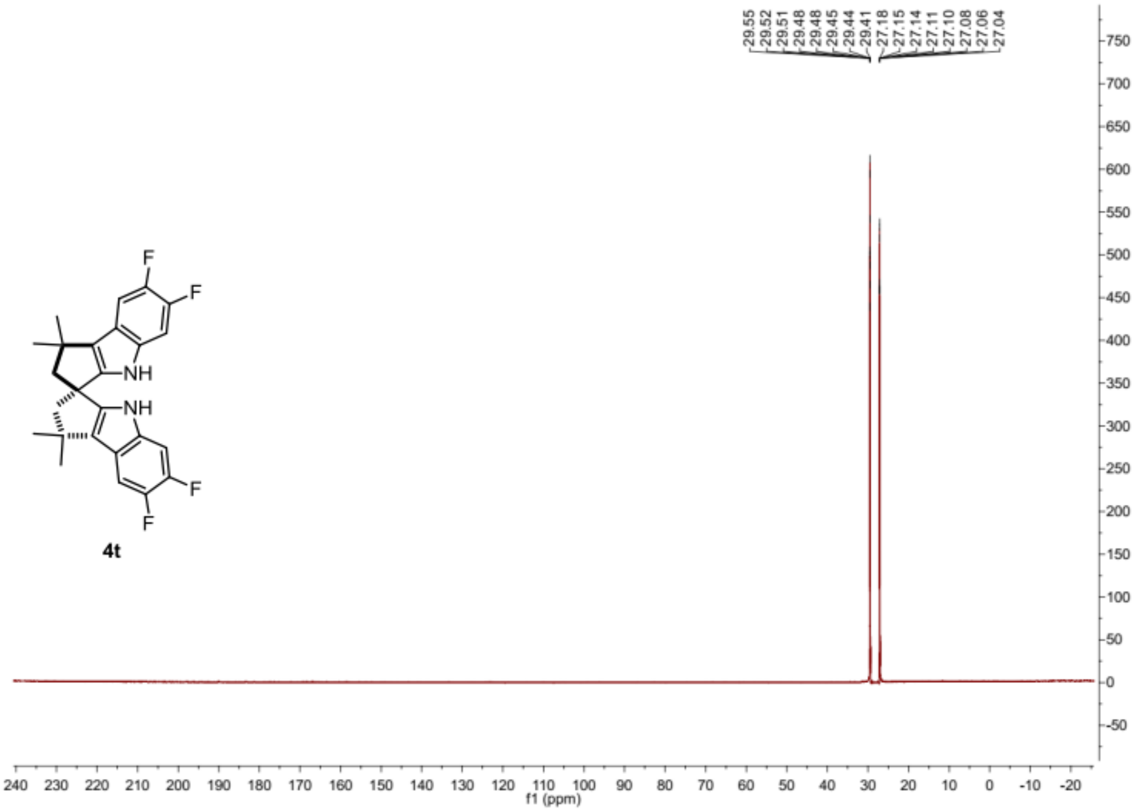

^{19}F NMR (282 MHz, Acetone-D_6, rt)

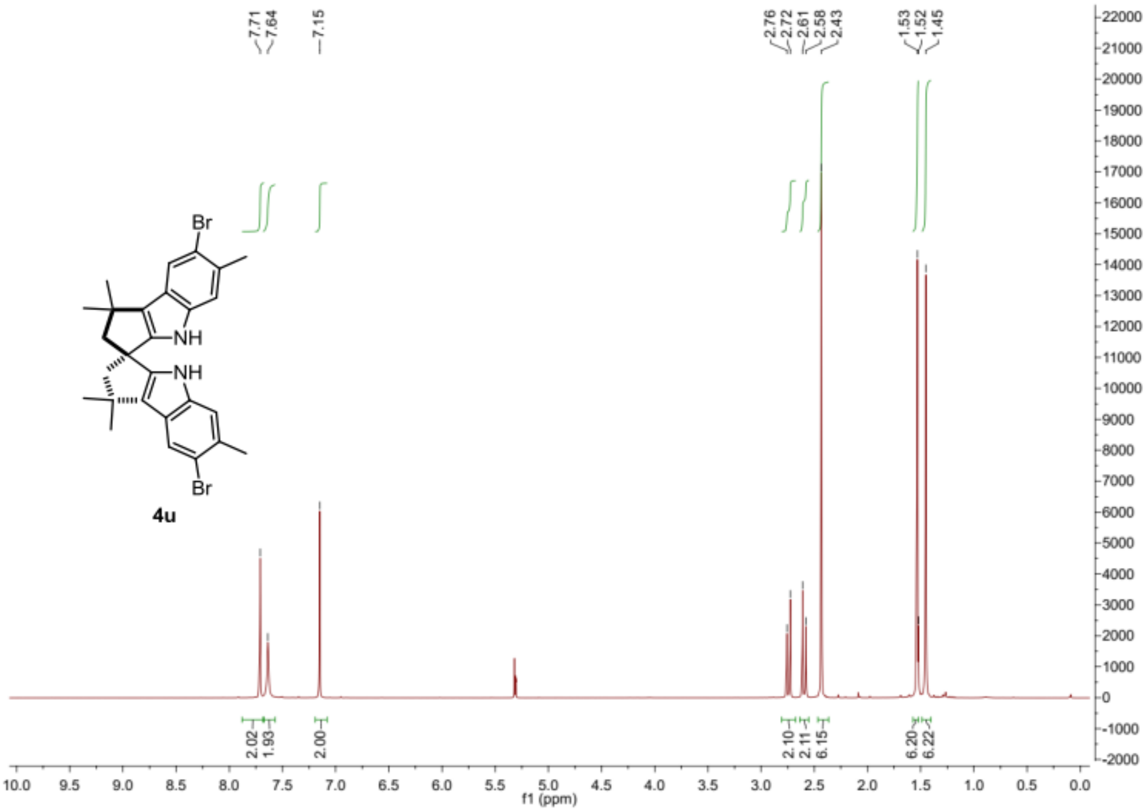

^{1}H NMR (400 MHz, CD_2Cl_2, rt)

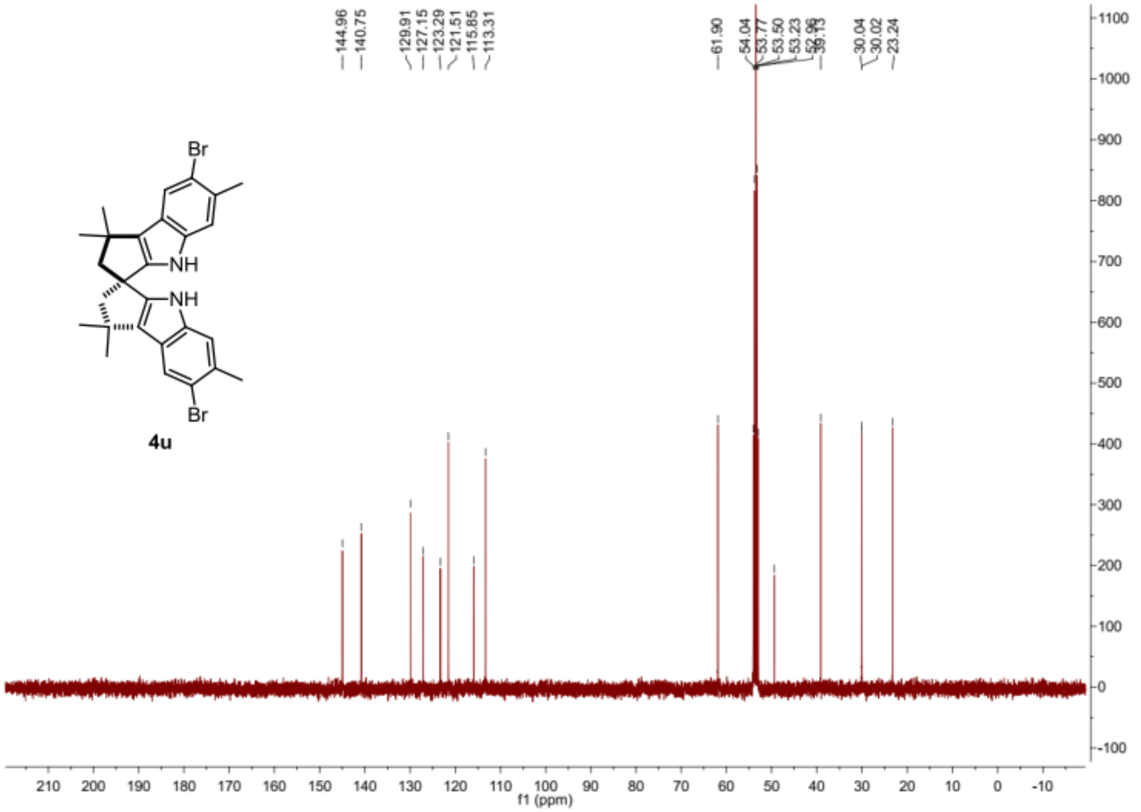

^{13}C NMR (101 MHz, CD_2Cl_2, rt)

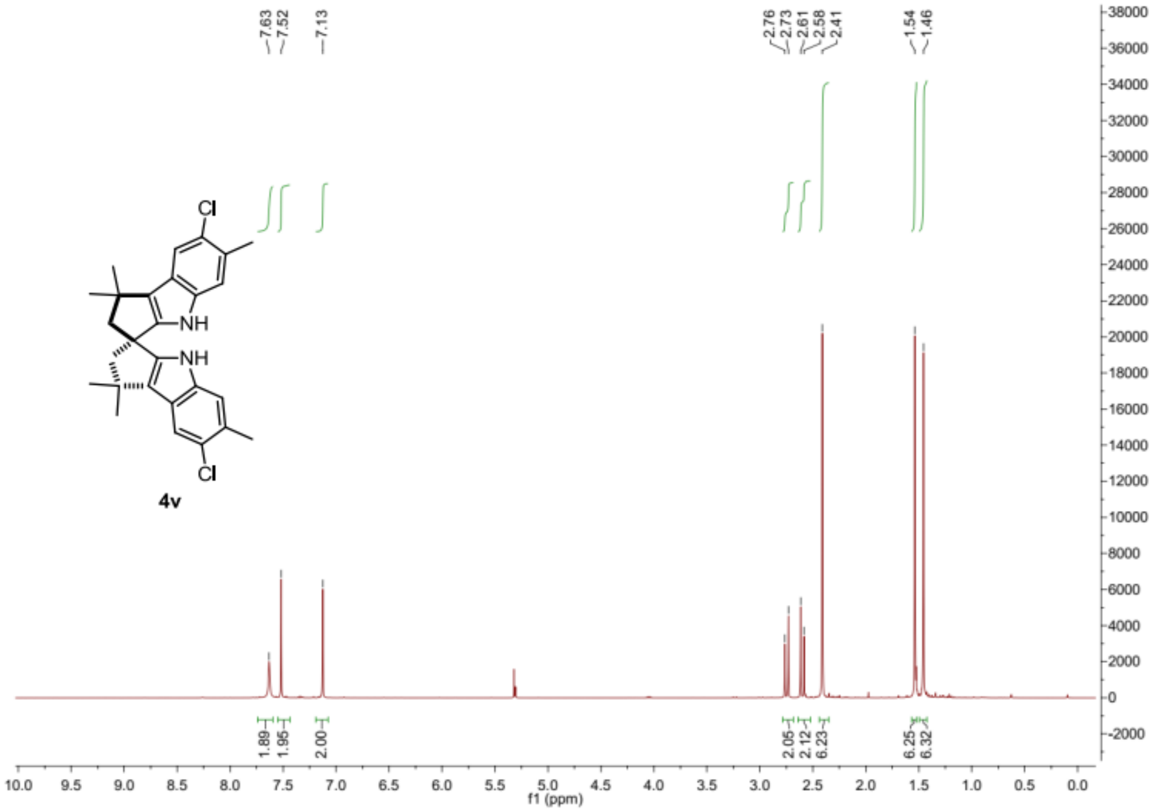

^{1}H NMR (400 MHz, CD_2Cl_2, rt)

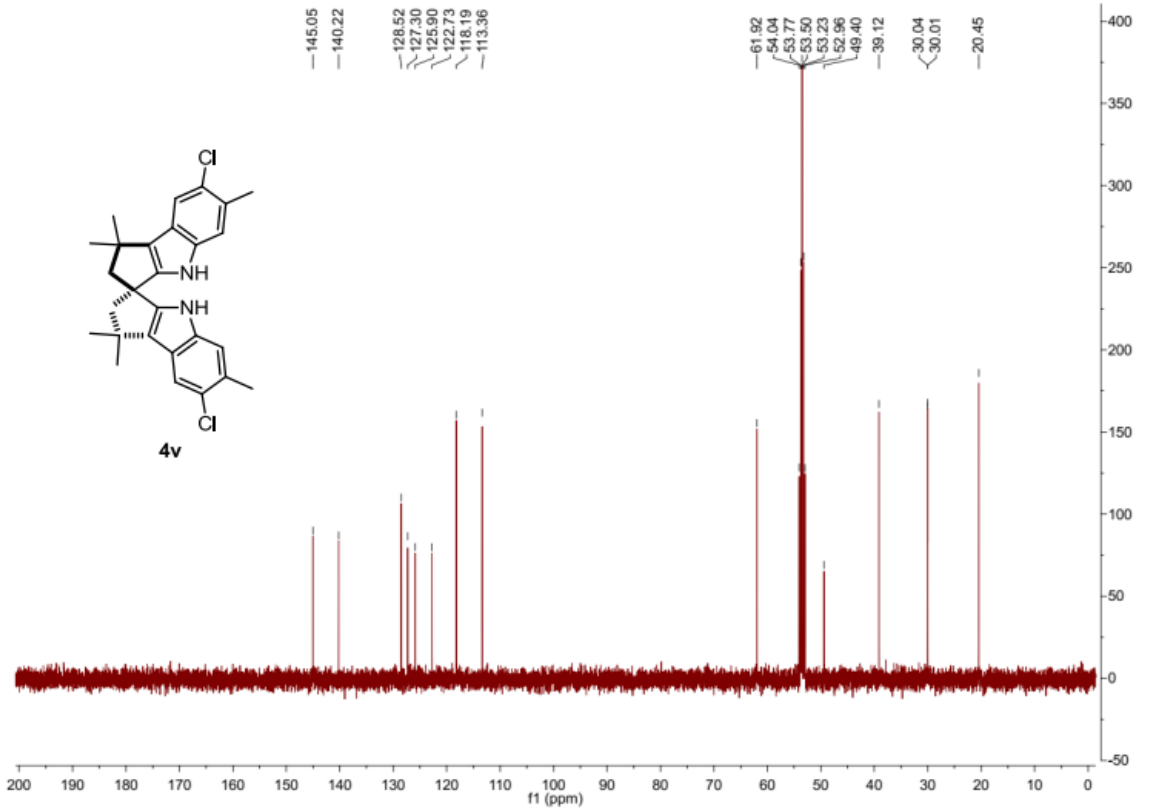

^{13}C NMR (101 MHz, CD_2Cl_2, rt)

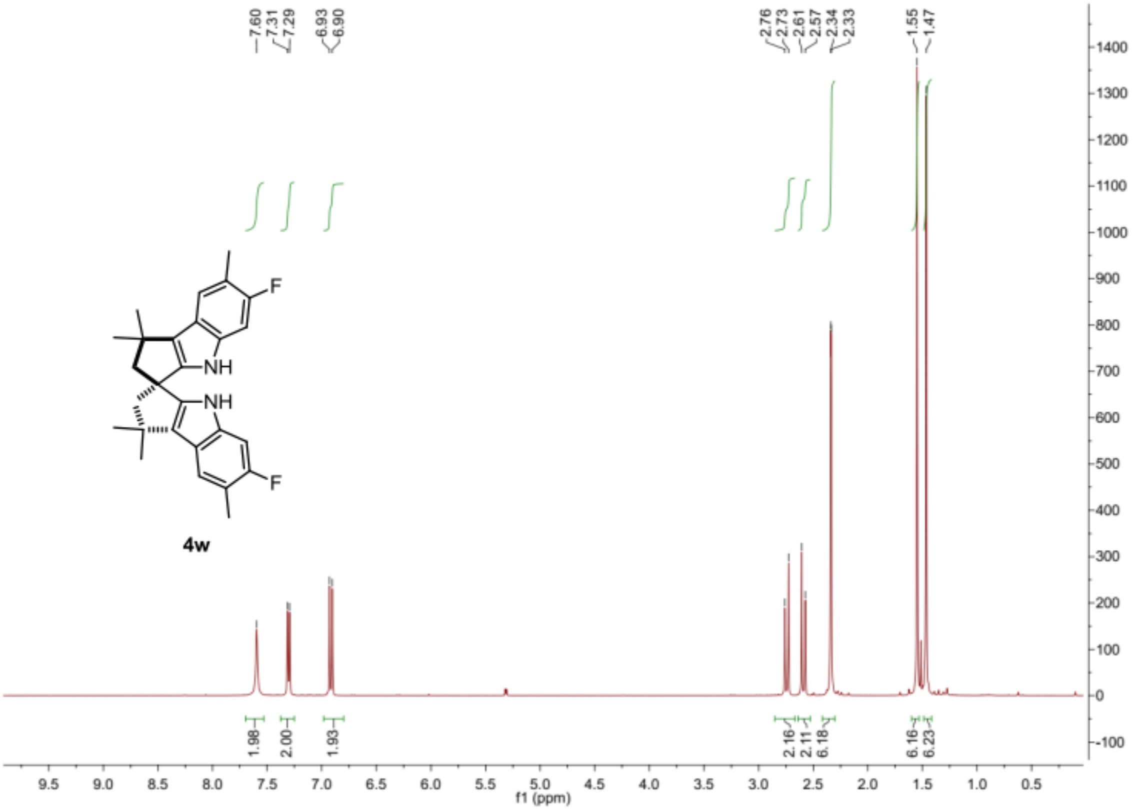

^{1}H NMR (400 MHz, CD_2Cl_2, rt)

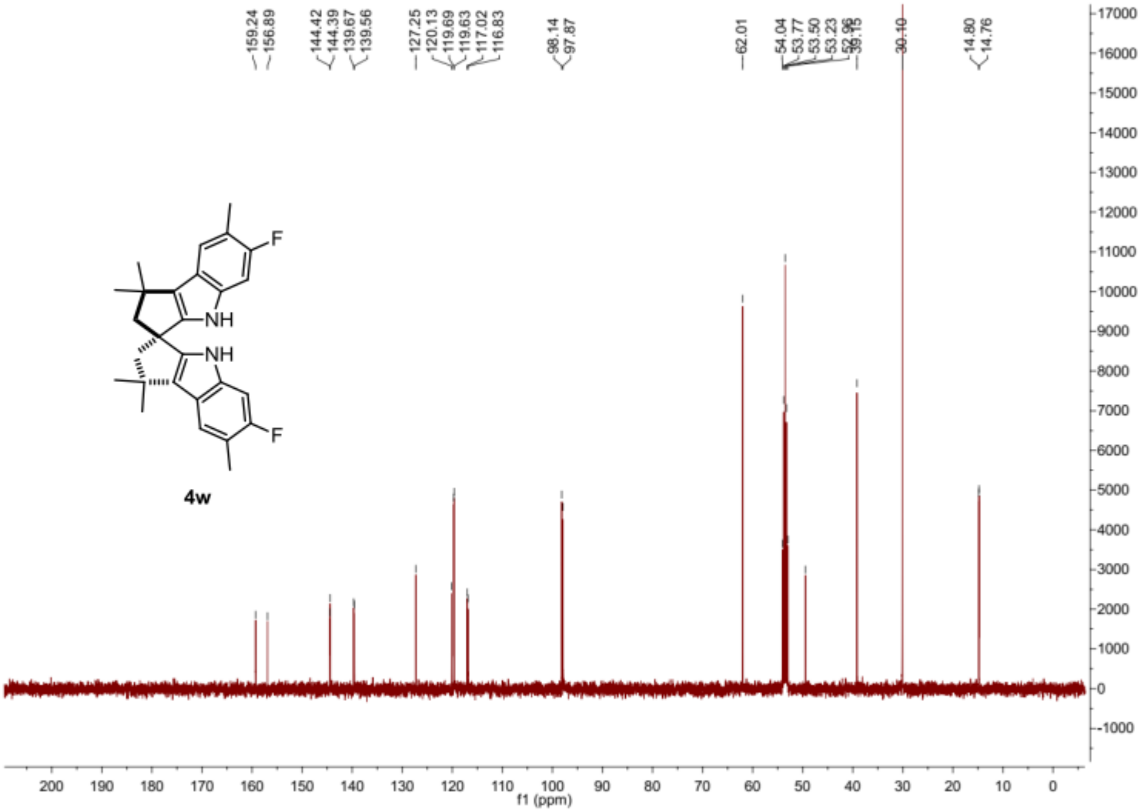

^{13}C NMR (101 MHz, CD_2Cl_2, rt)

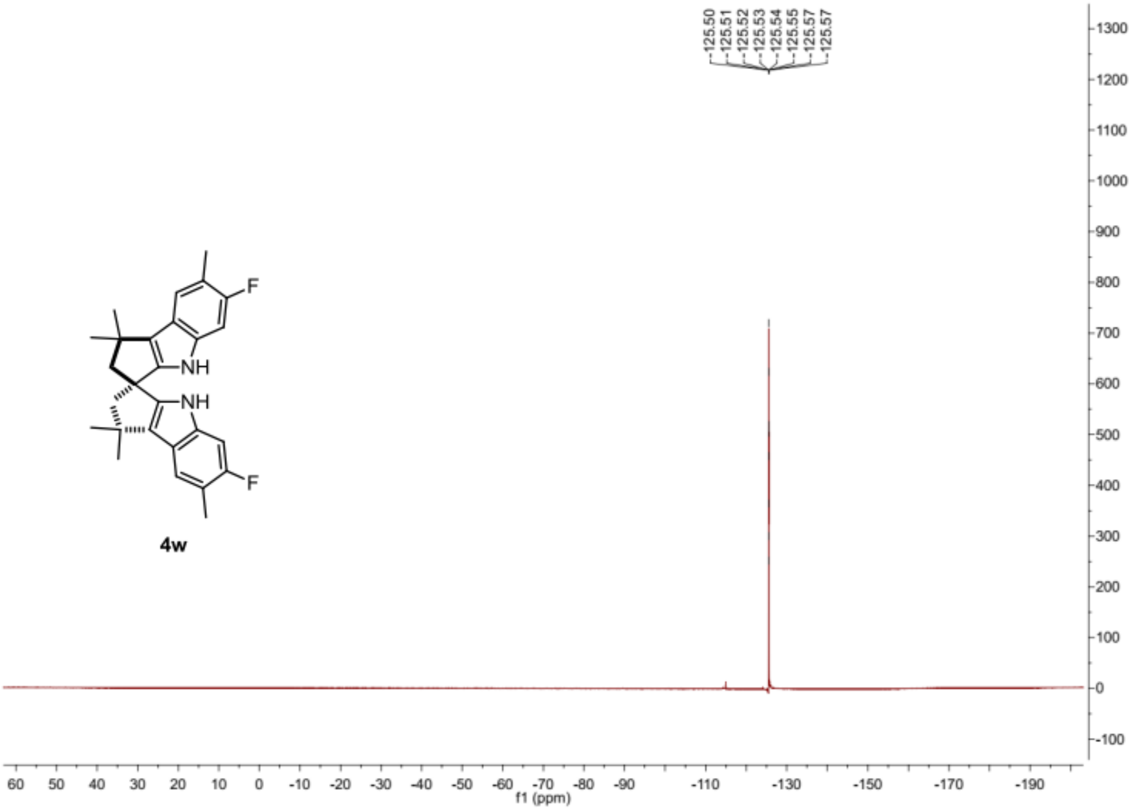

^{19}F NMR (282 MHz, CD_2Cl_2, rt)

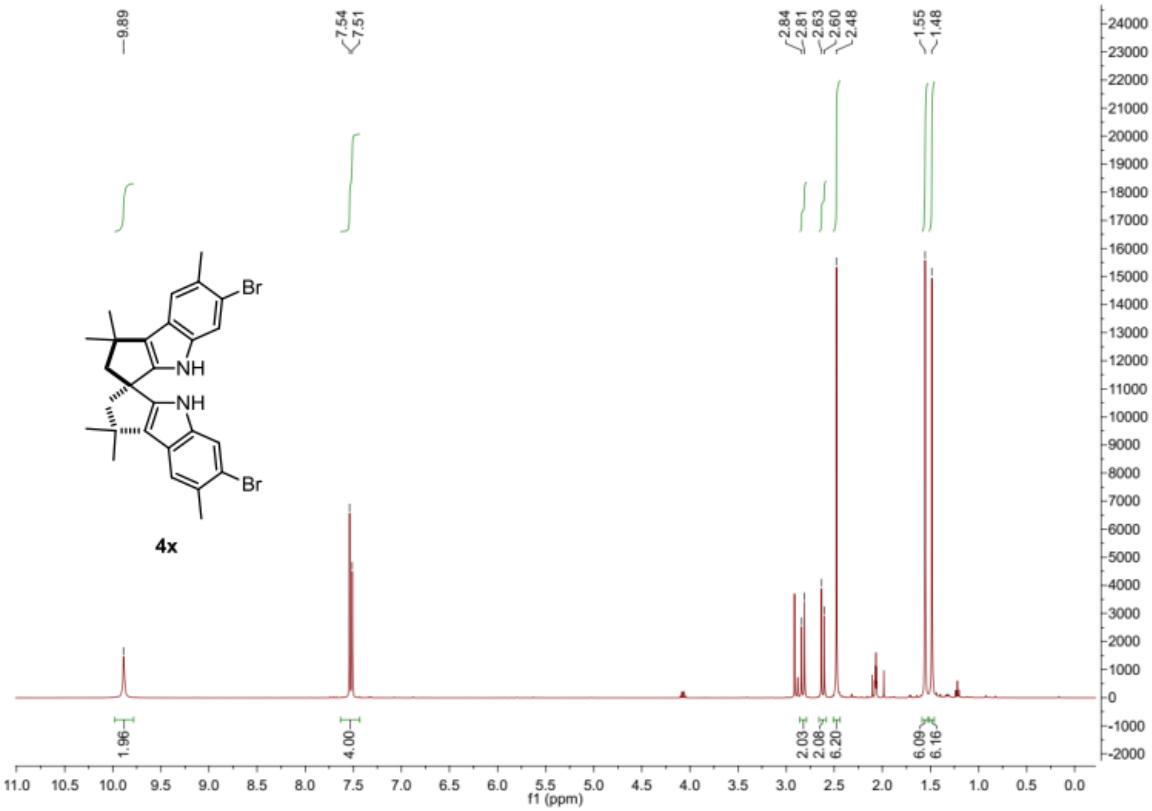

^1H NMR (400 MHz, Acetone-D_6, rt)

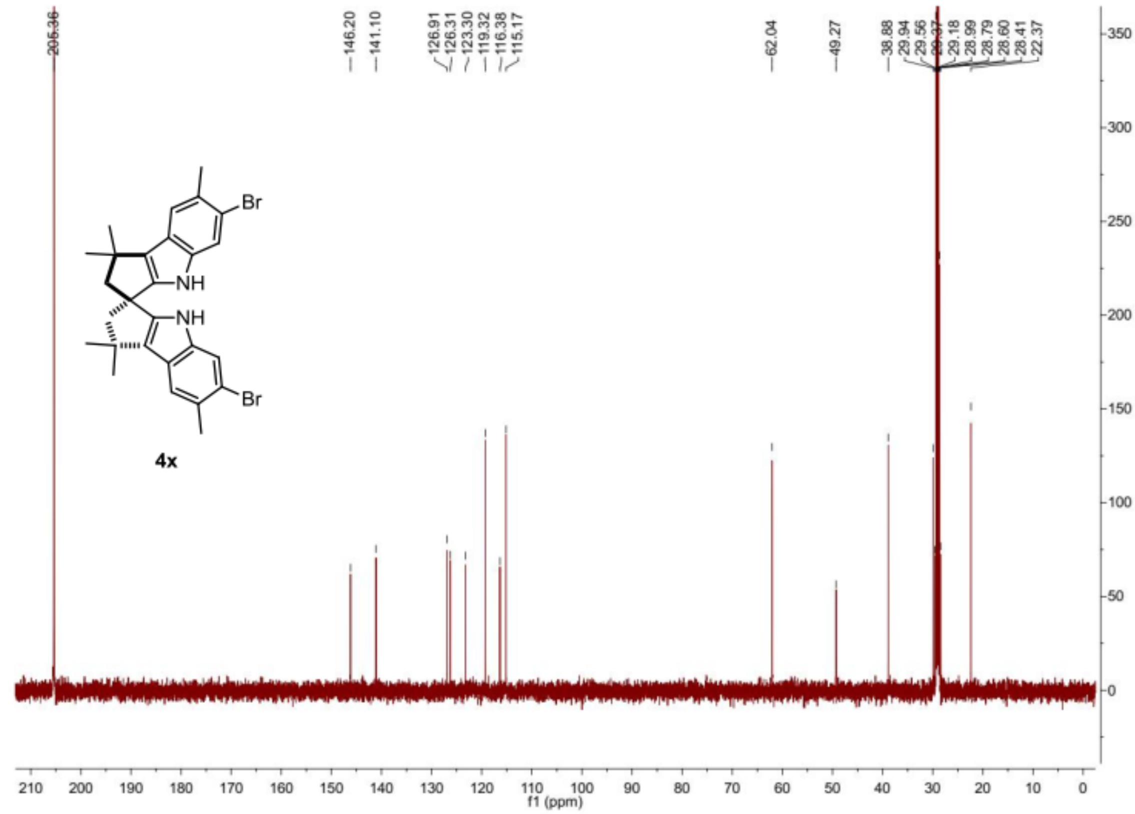

^{13}C NMR (101 MHz, Acetone-D_6, rt)

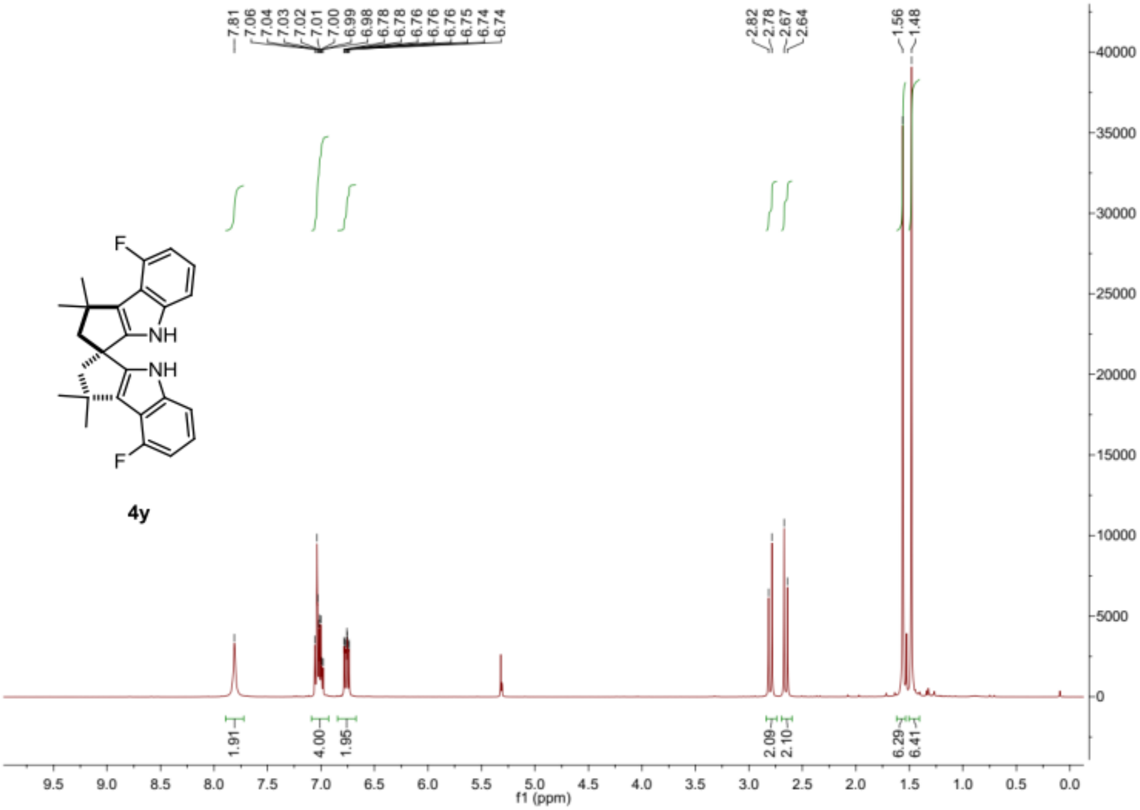

^{1}H NMR (400 MHz, CD_2Cl_2, rt)

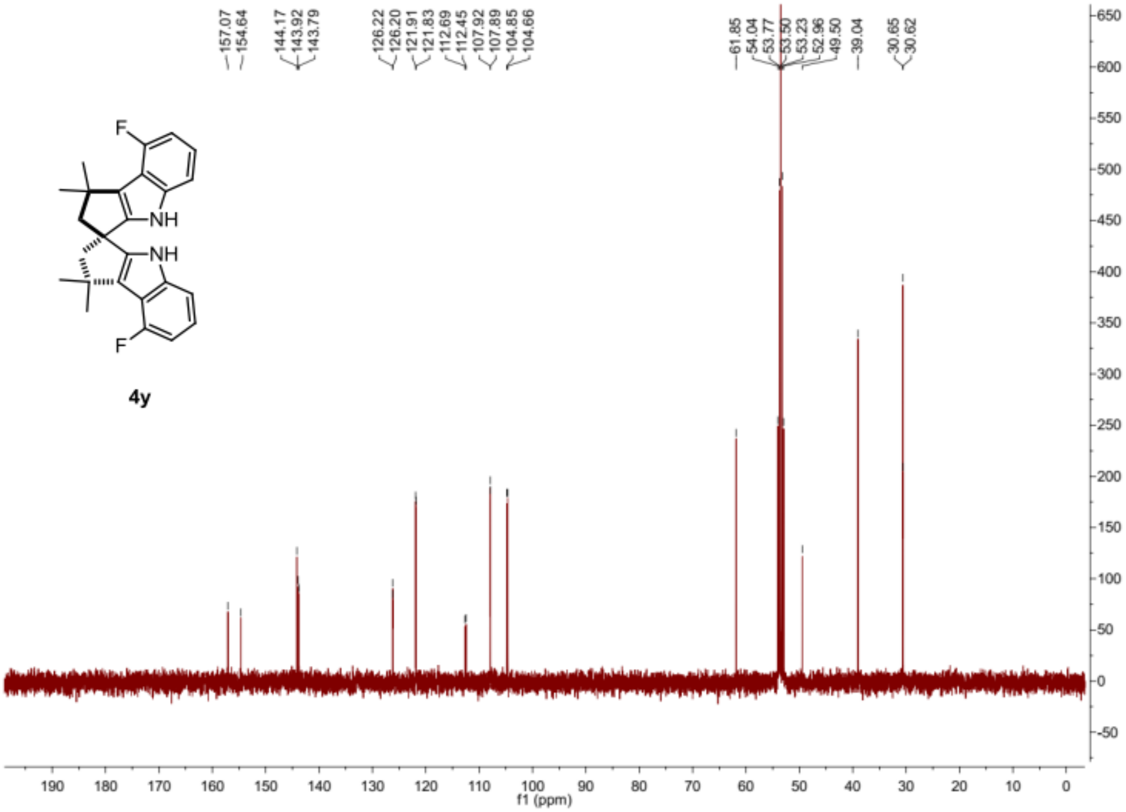

^{13}C NMR (101 MHz, CD_2Cl_2, rt)

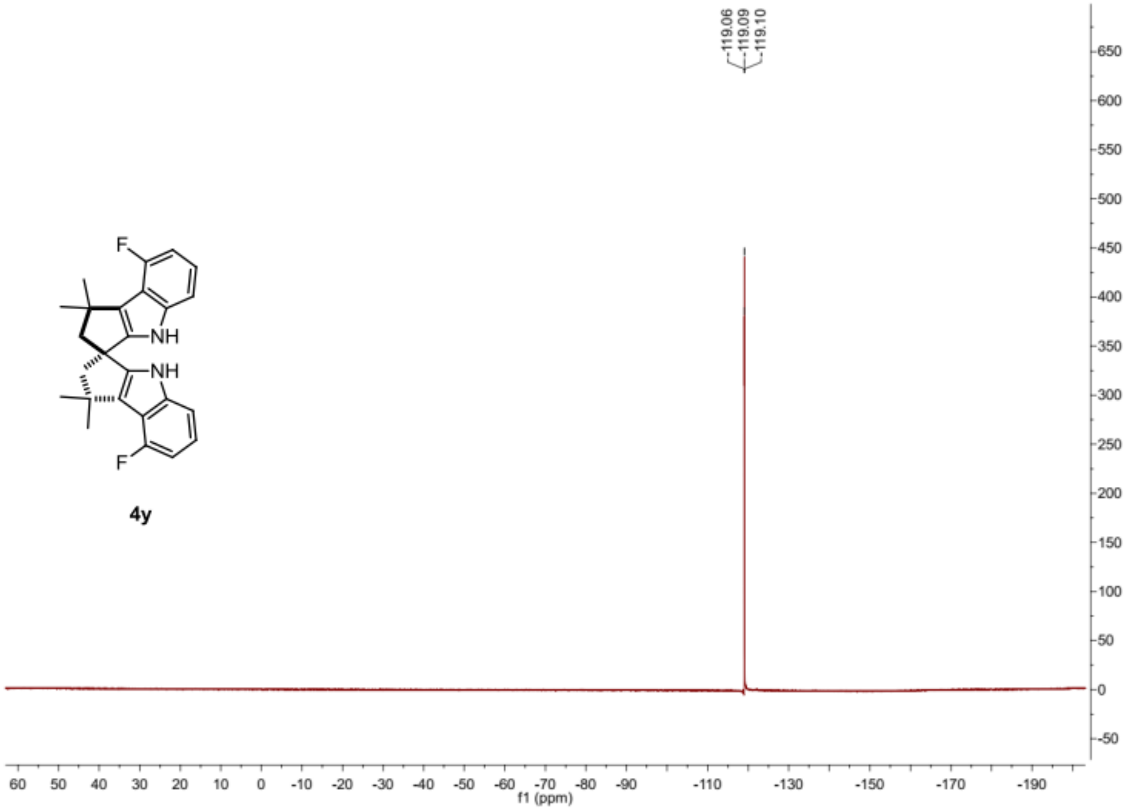

^{19}F NMR (282 MHz, CD_2Cl_2, rt)

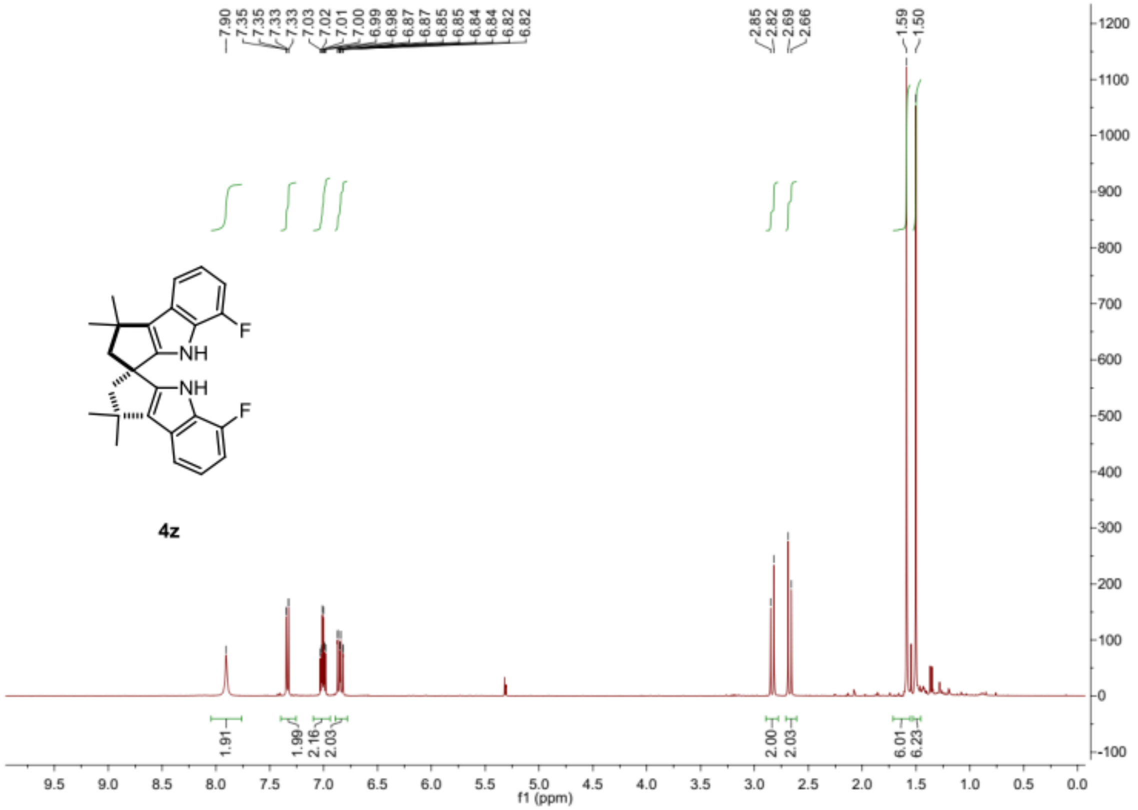

^1H NMR (400 MHz, CD_2Cl_2, rt)

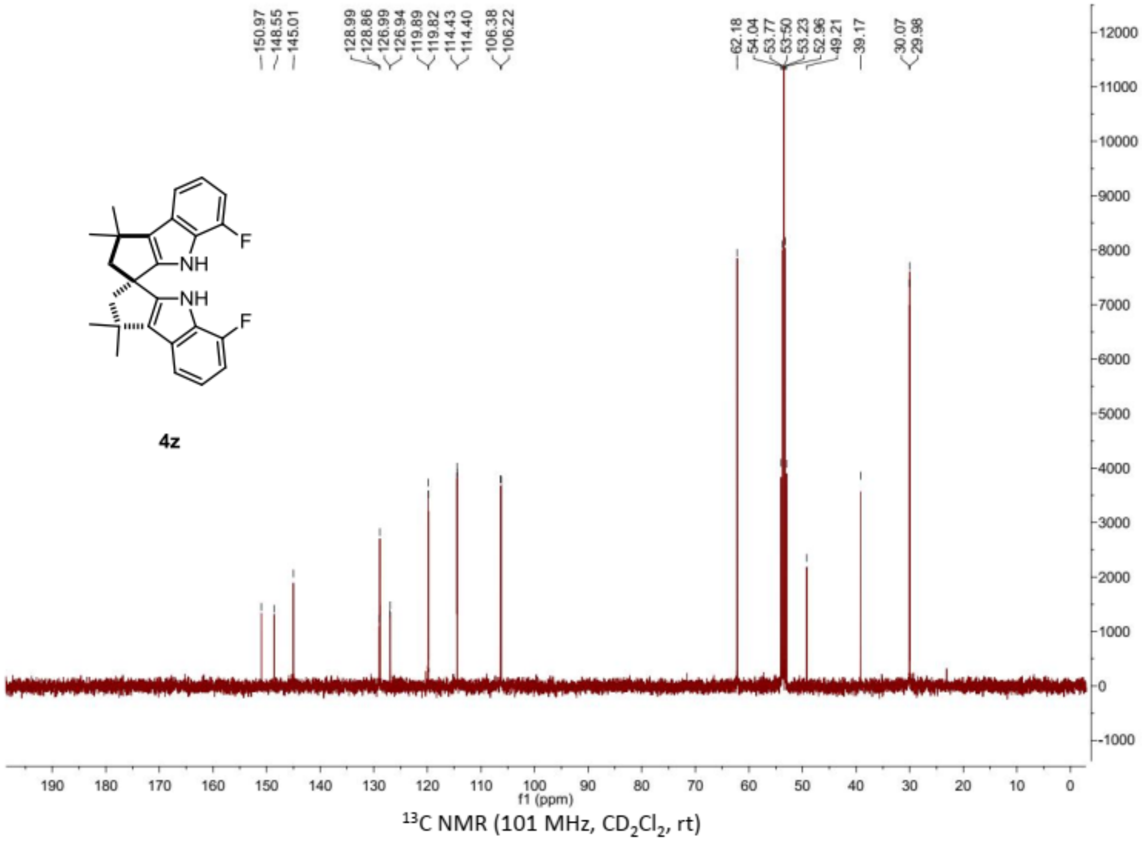

^{13}C NMR (101 MHz, CD_2Cl_2, rt)

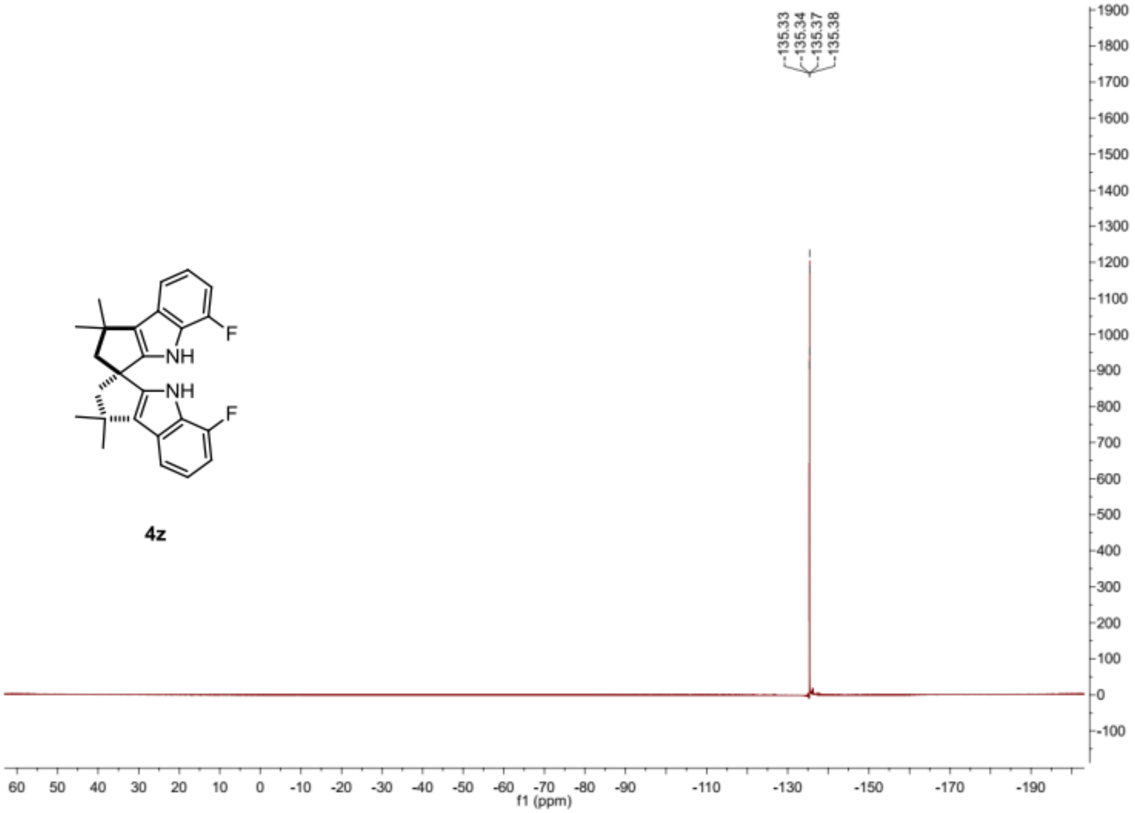

^{19}F NMR (282 MHz, CD_2Cl_2, rt)

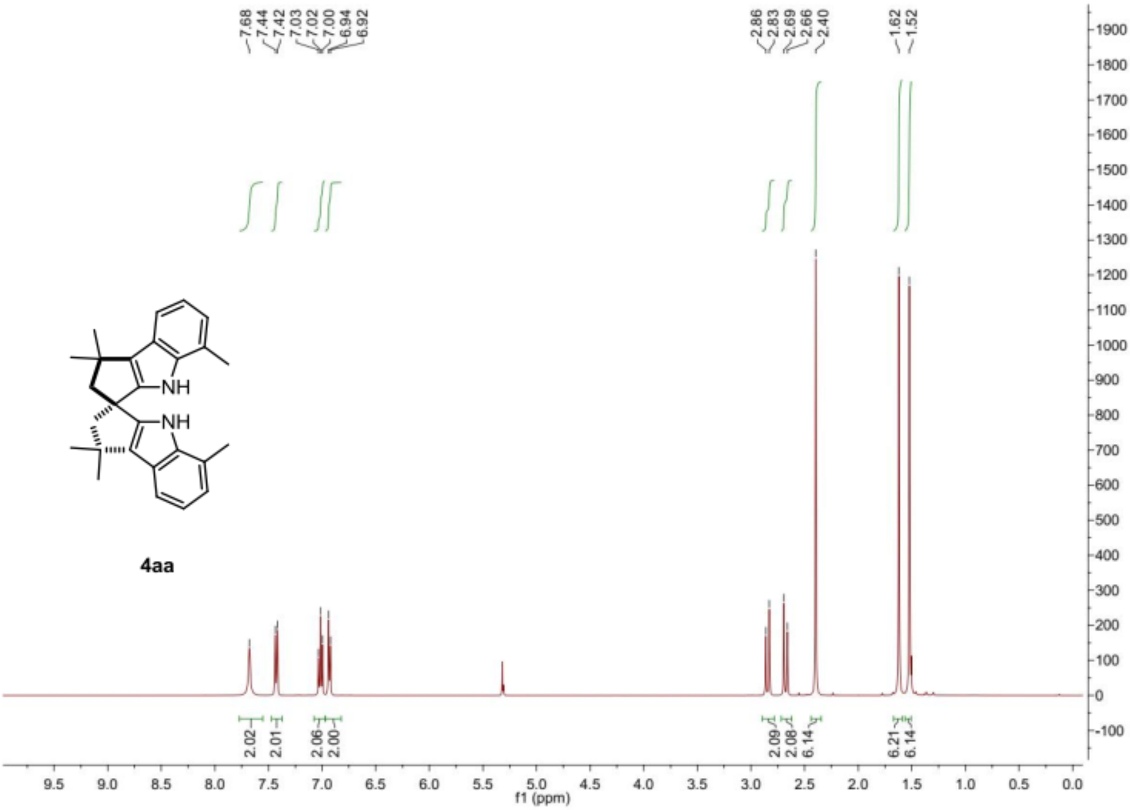

^{1}H NMR (400 MHz, CD_2Cl_2, rt)

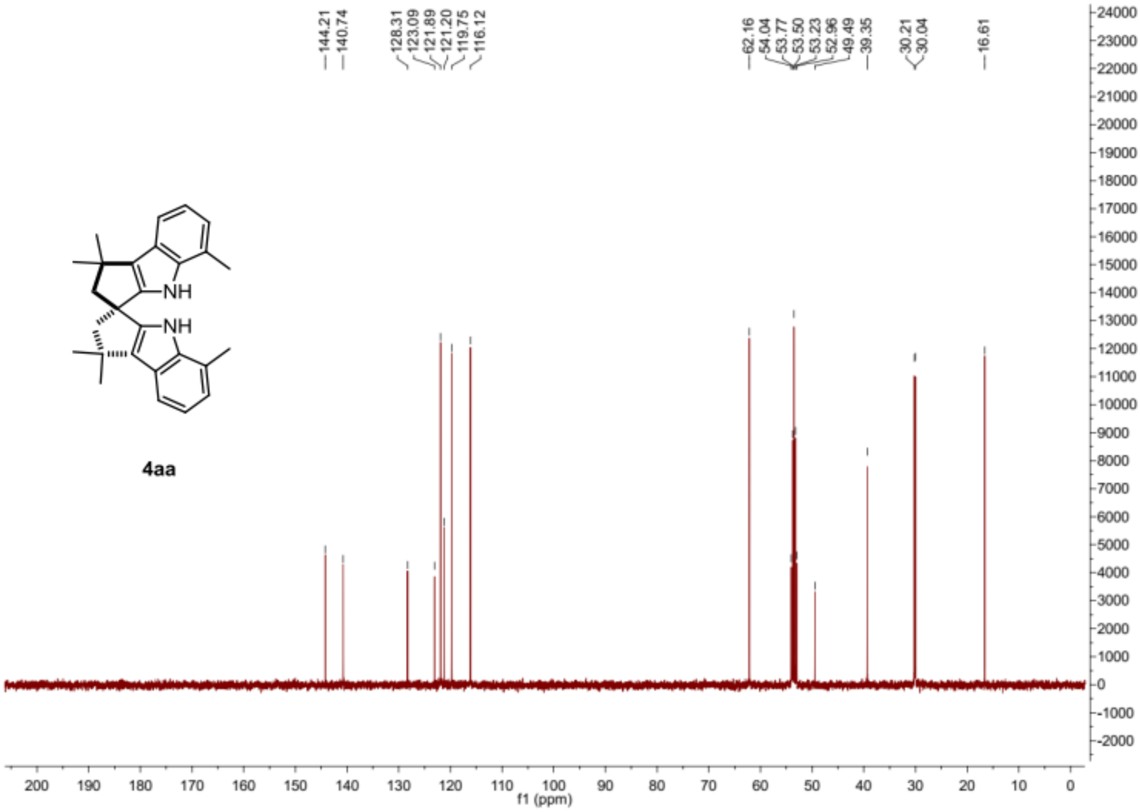

^{13}C NMR (101 MHz, CD_2Cl_2, rt)

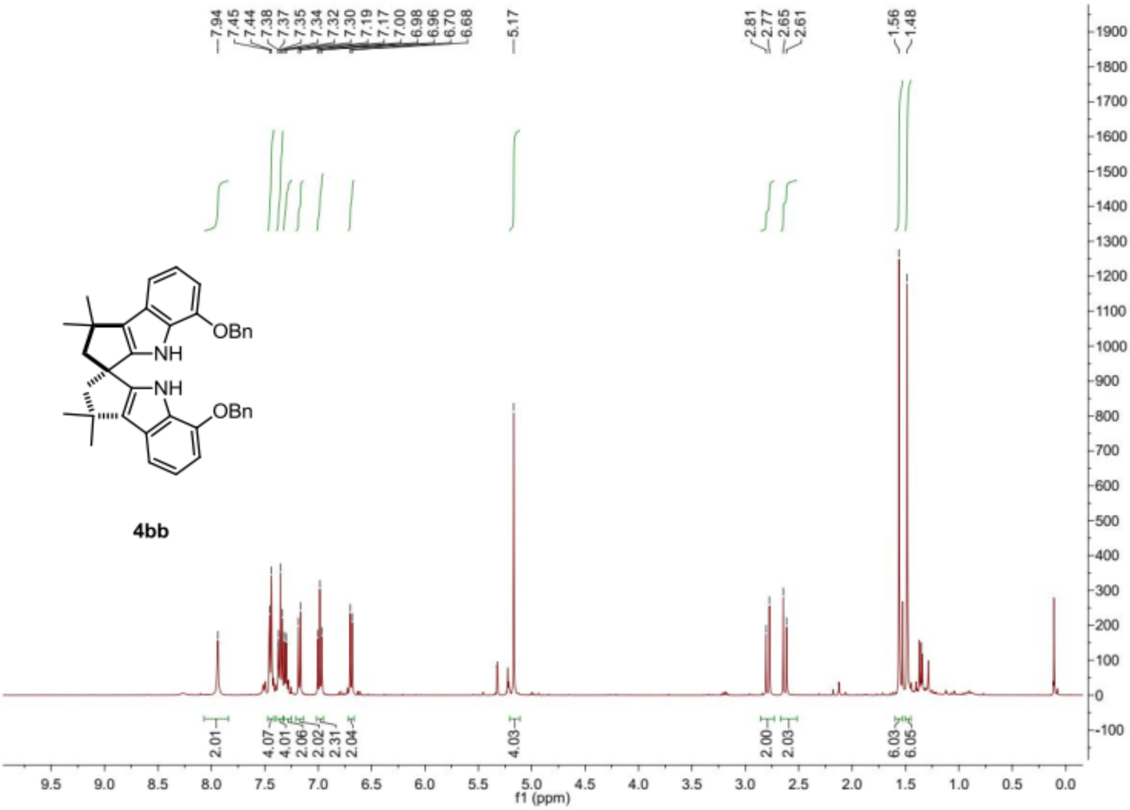

^{1}H NMR (400 MHz, CD_2Cl_2, rt)

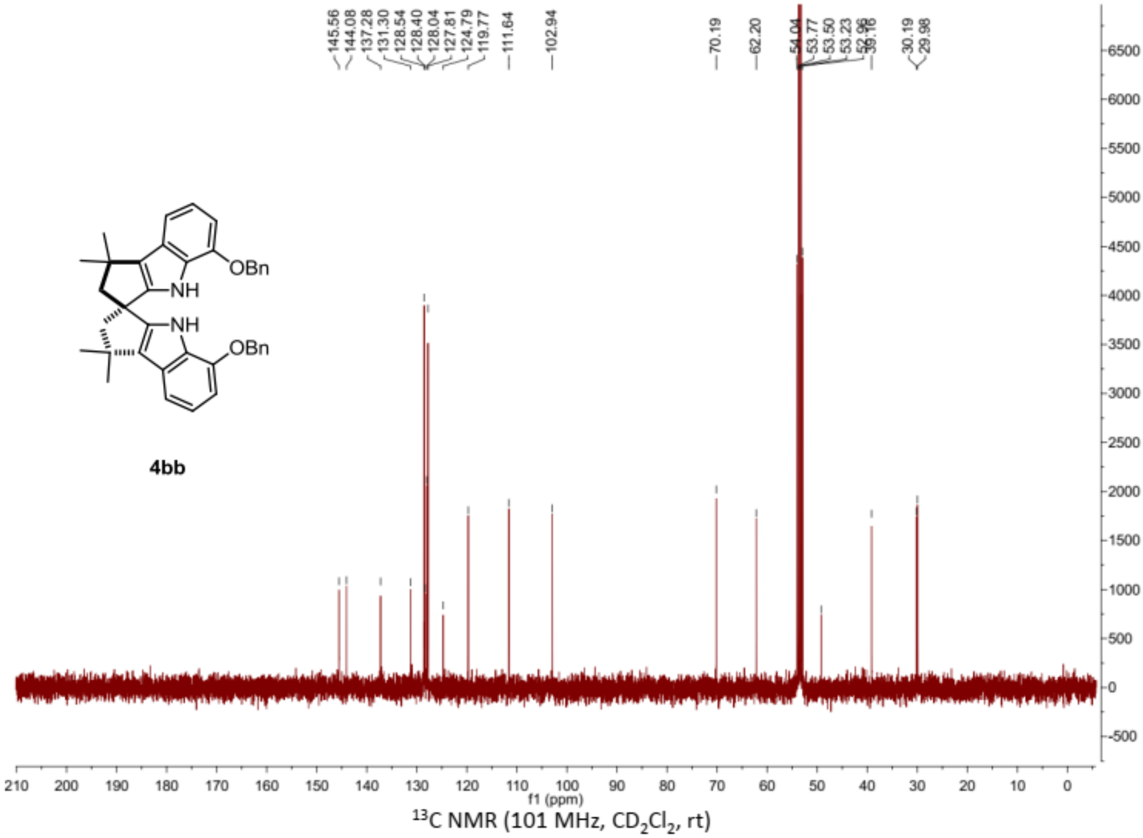

^{13}C NMR (101 MHz, CD_2Cl_2, rt)

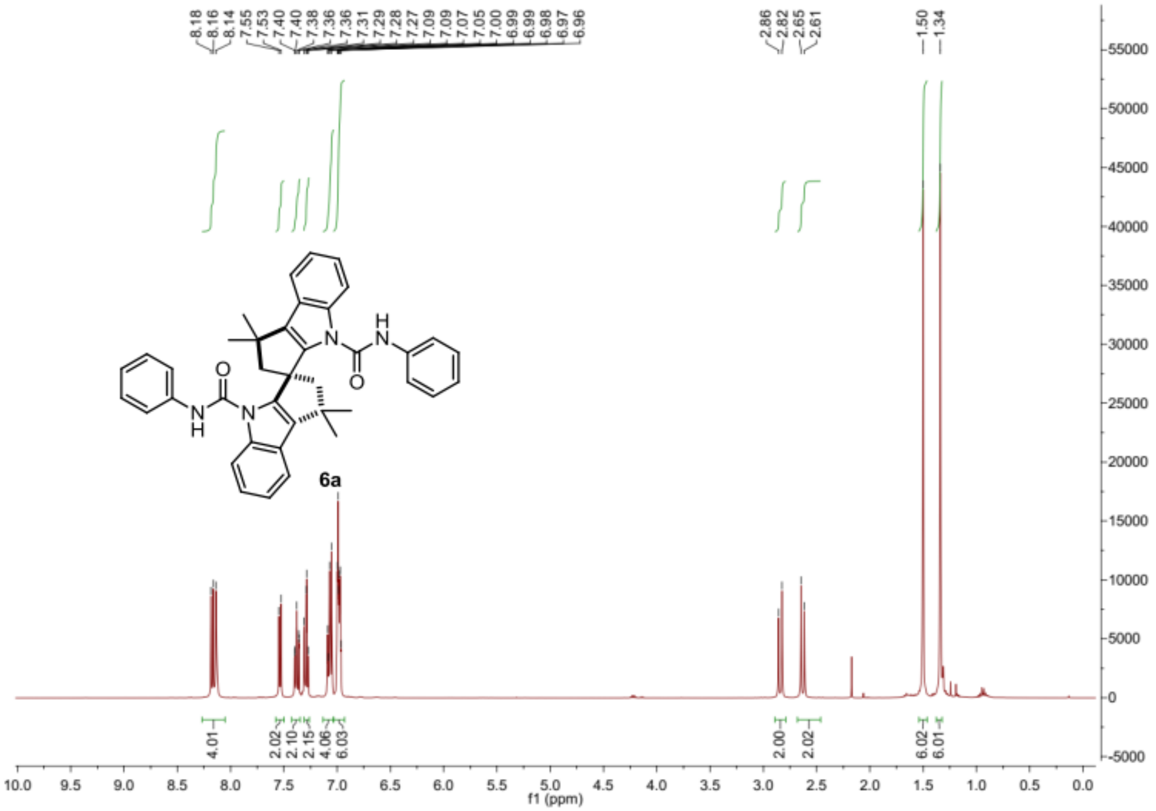

^{1}H NMR (400 MHz, $CDCl_3$, rt)

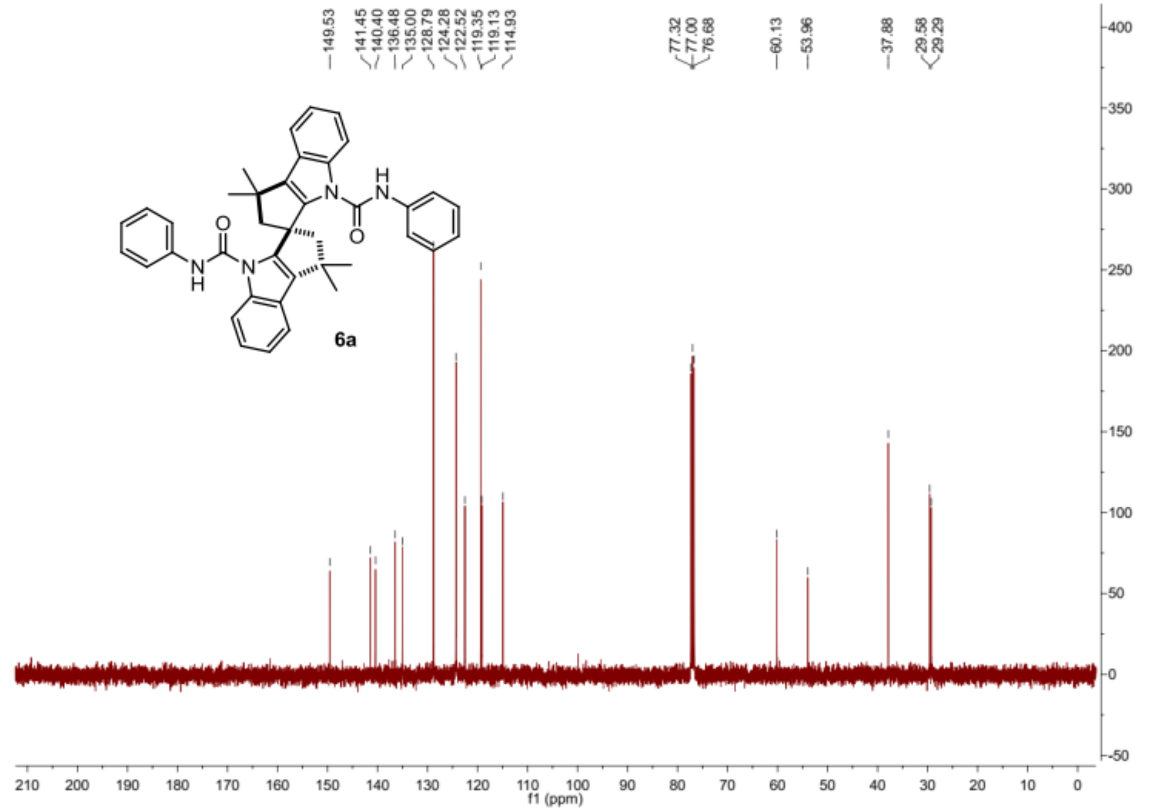

^{13}C NMR (101 MHz, $CDCl_3$, rt)

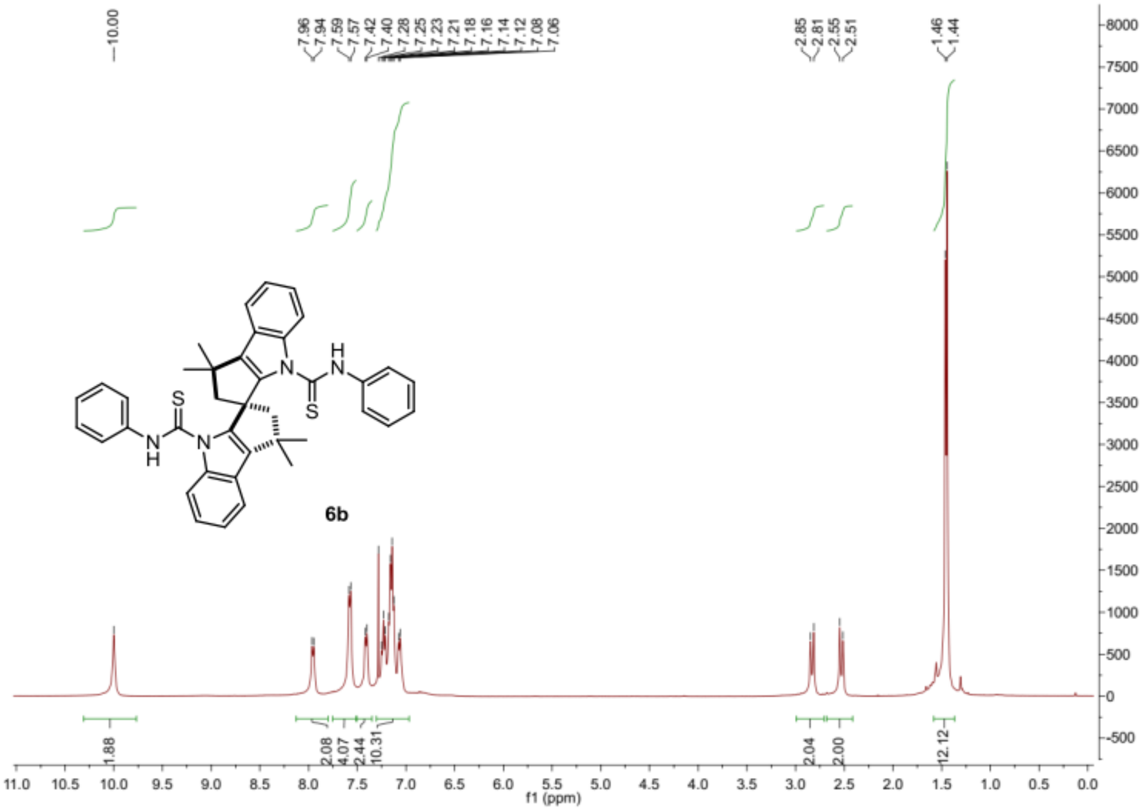

^{1}H NMR (400 MHz, CD_2Cl_2, rt)
^{1}H NMR (400 MHz, $CDCl_3$, rt)

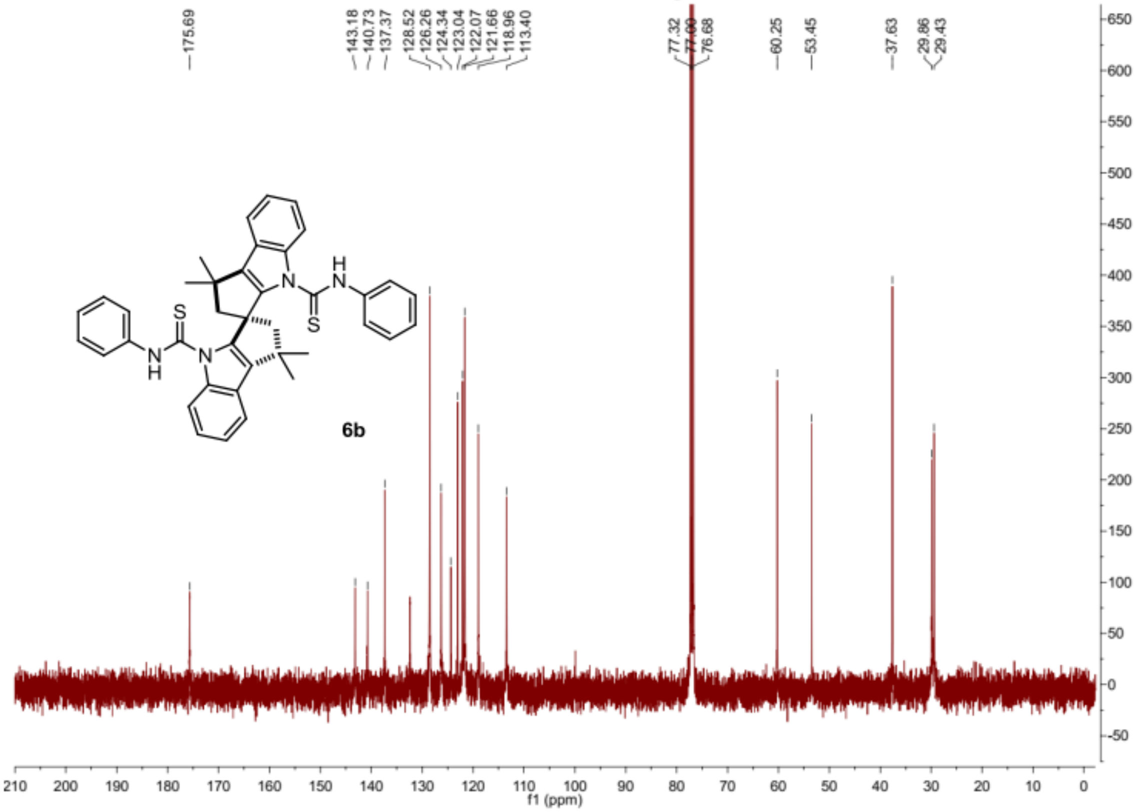

^{13}C NMR (101 MHz, $CDCl_3$, rt)

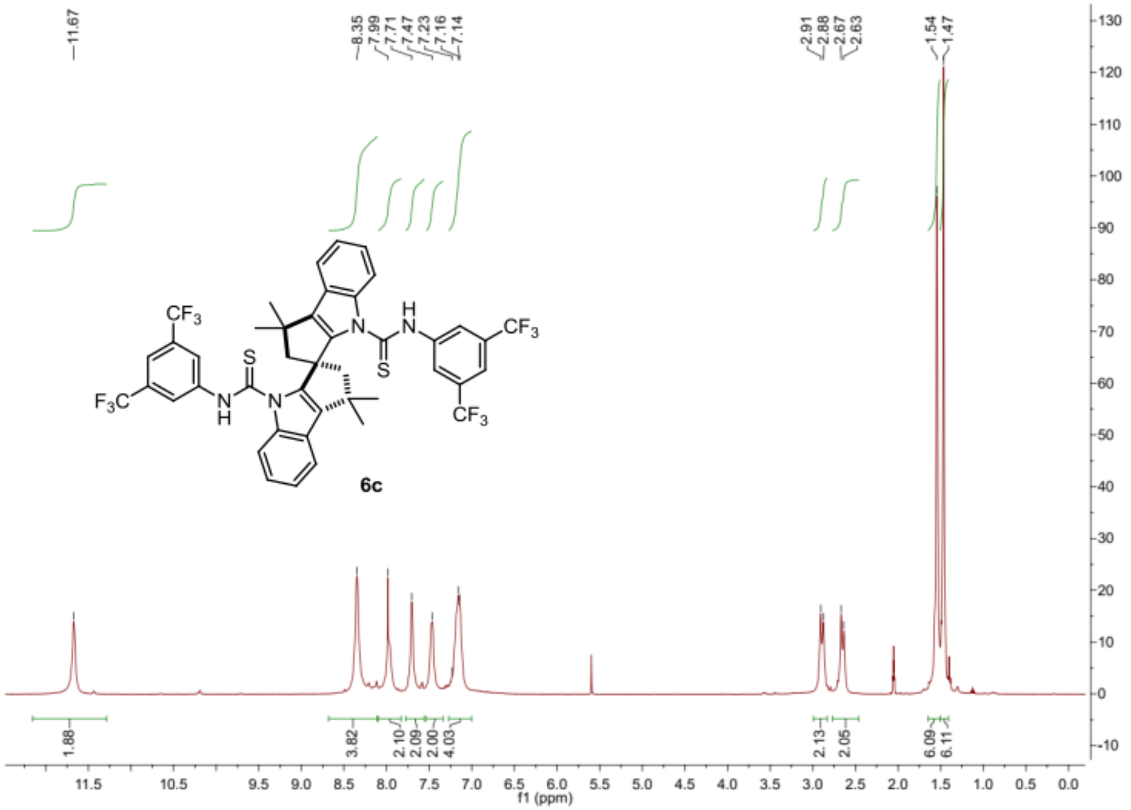

^{1}H NMR (400 MHz, Acetone-D_6, rt)

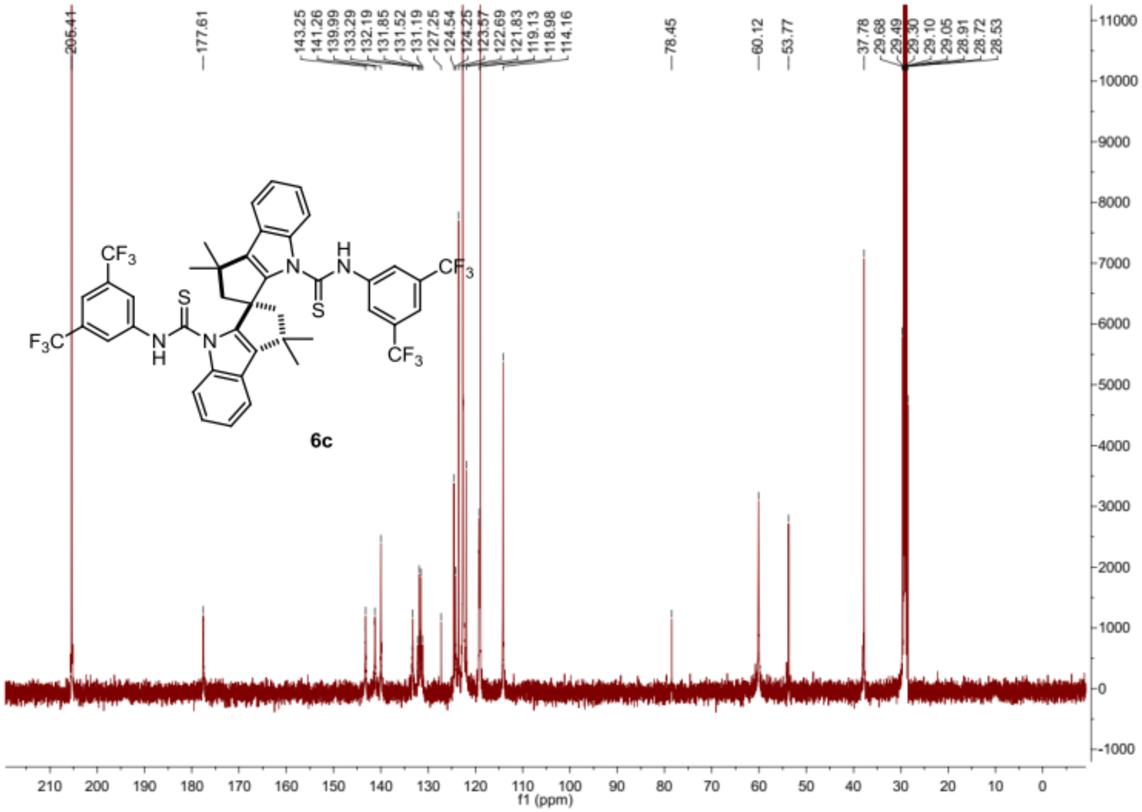

^{13}C NMR (101 MHz, Acetone-D_6, rt)

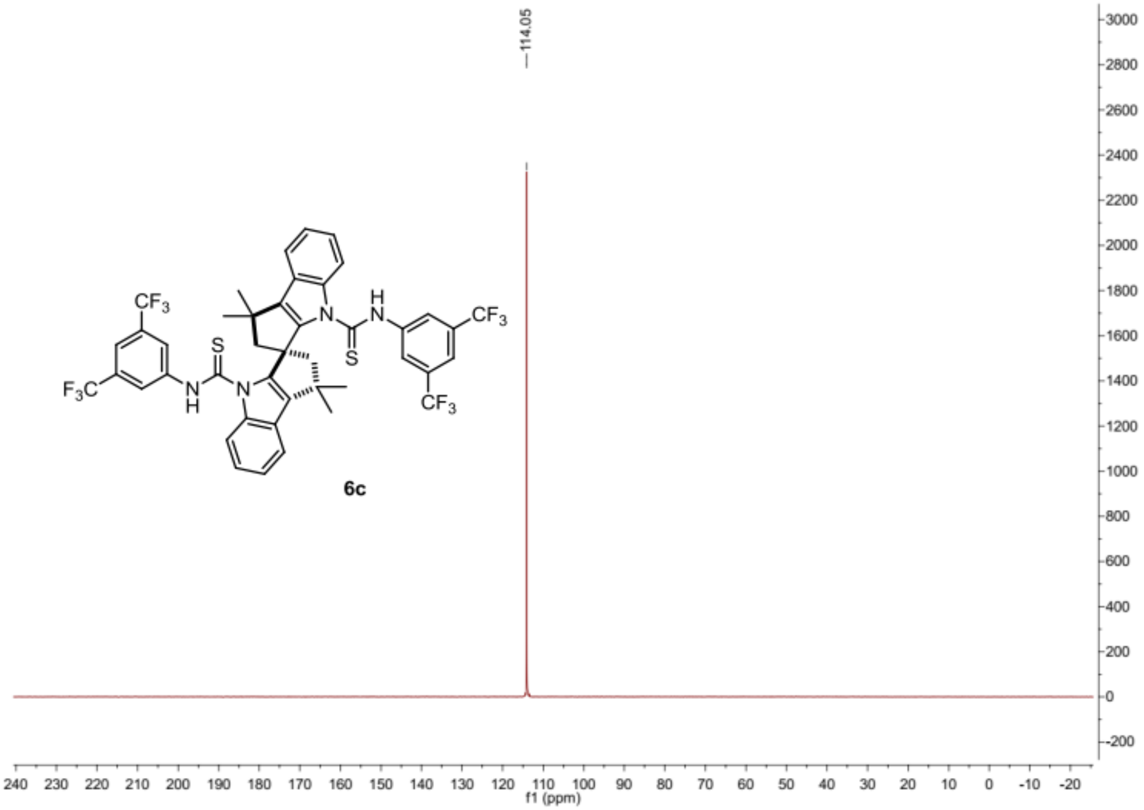

^{19}F NMR (282 MHz, Acetone-D$_6$, rt)

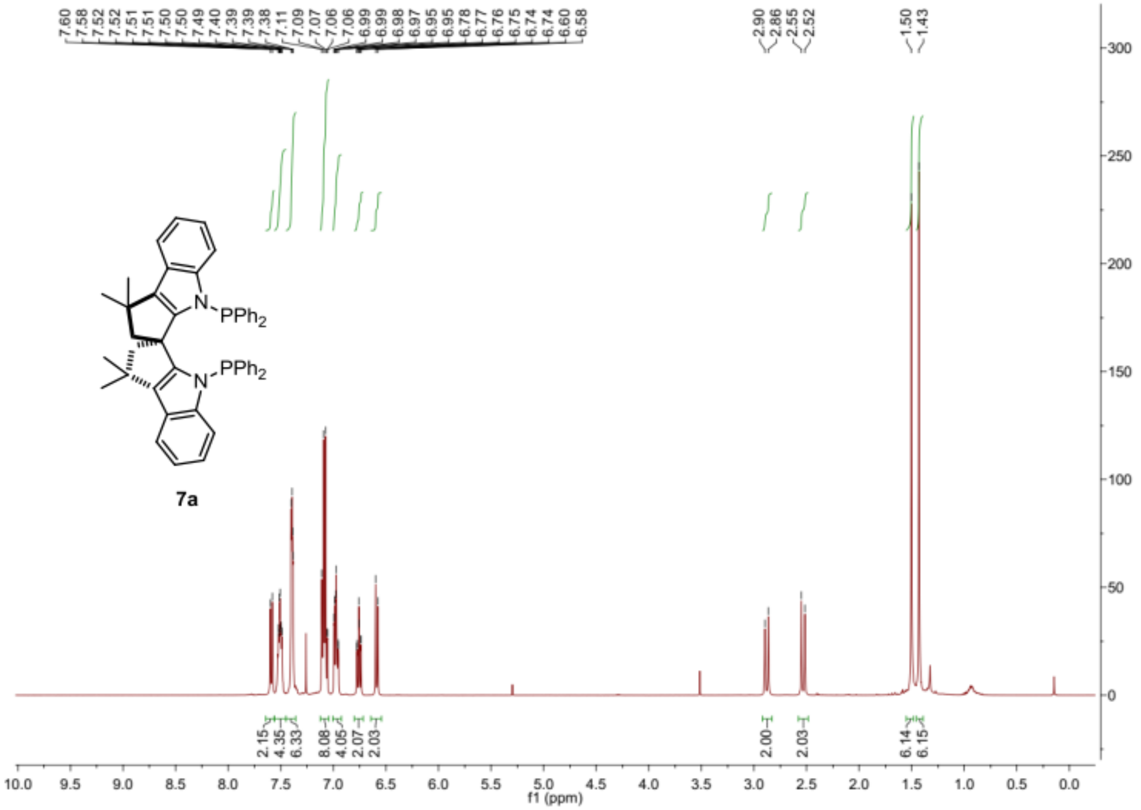

7a

¹H NMR (400 MHz, $CDCl_3$, rt)

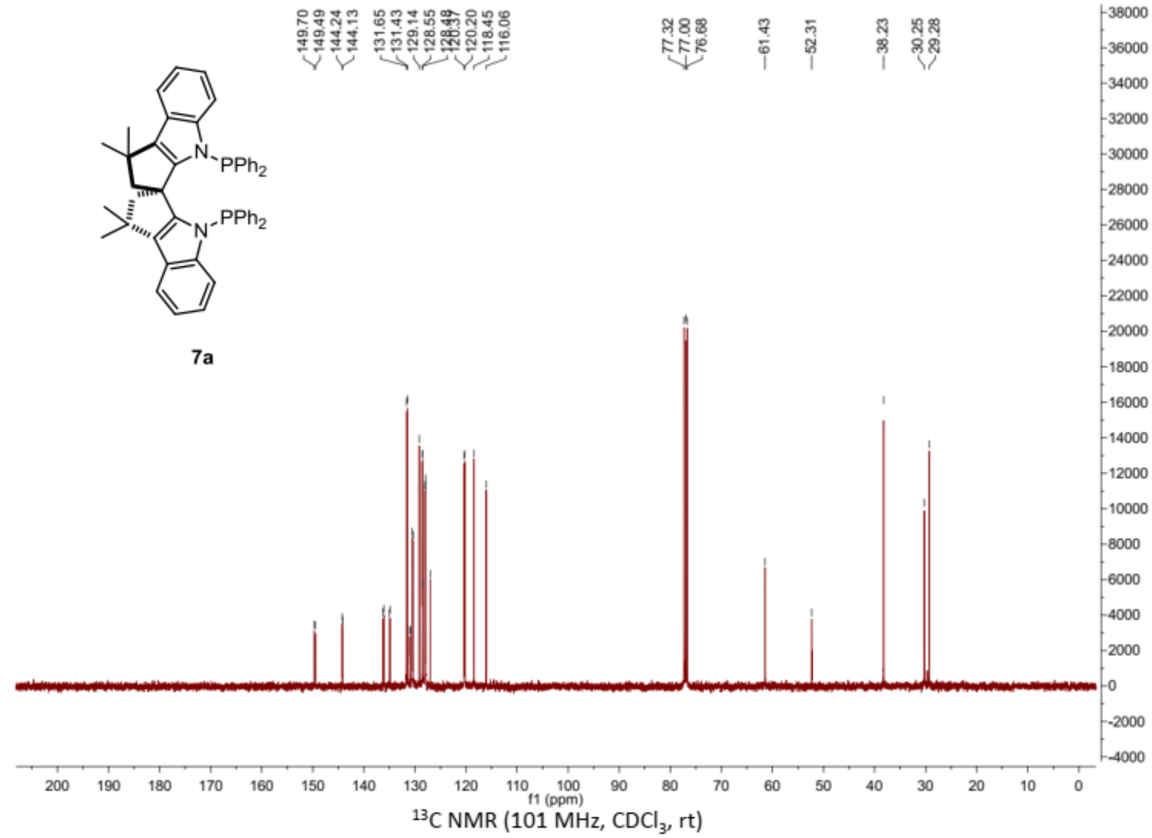

7a

¹³C NMR (101 MHz, $CDCl_3$, rt)

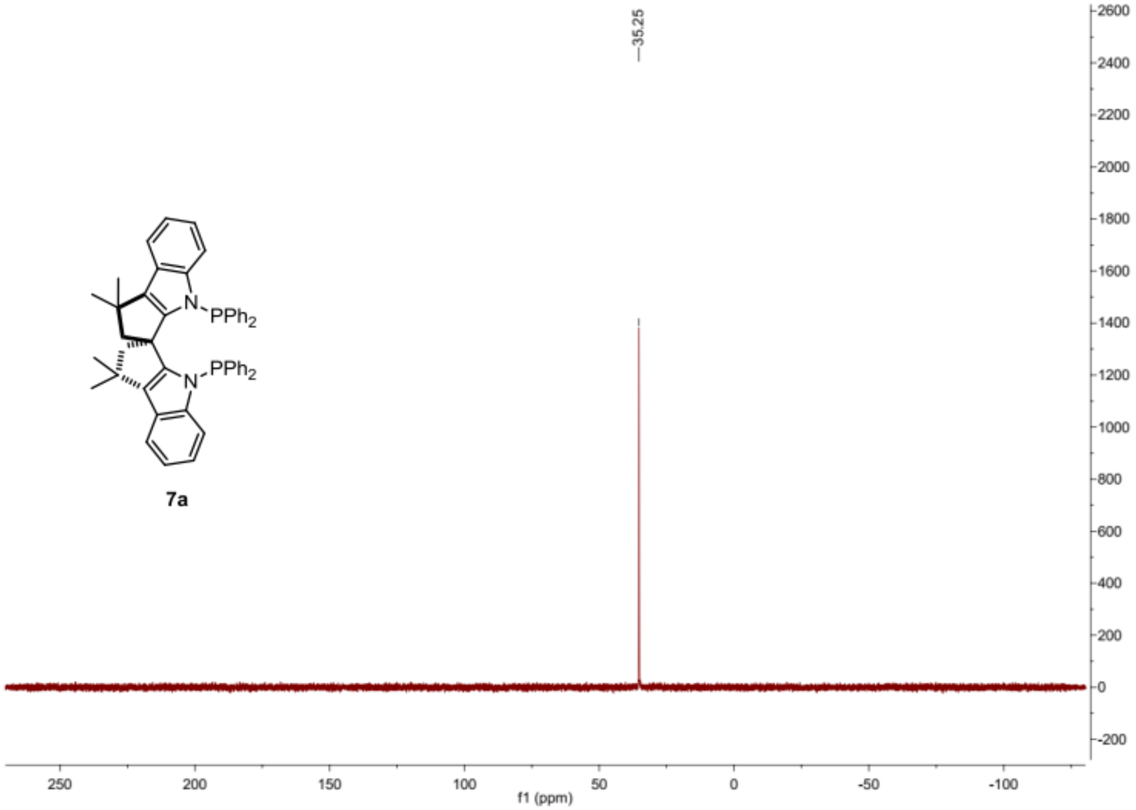

^{31}P NMR (121 MHz, $CDCl_3$, rt)

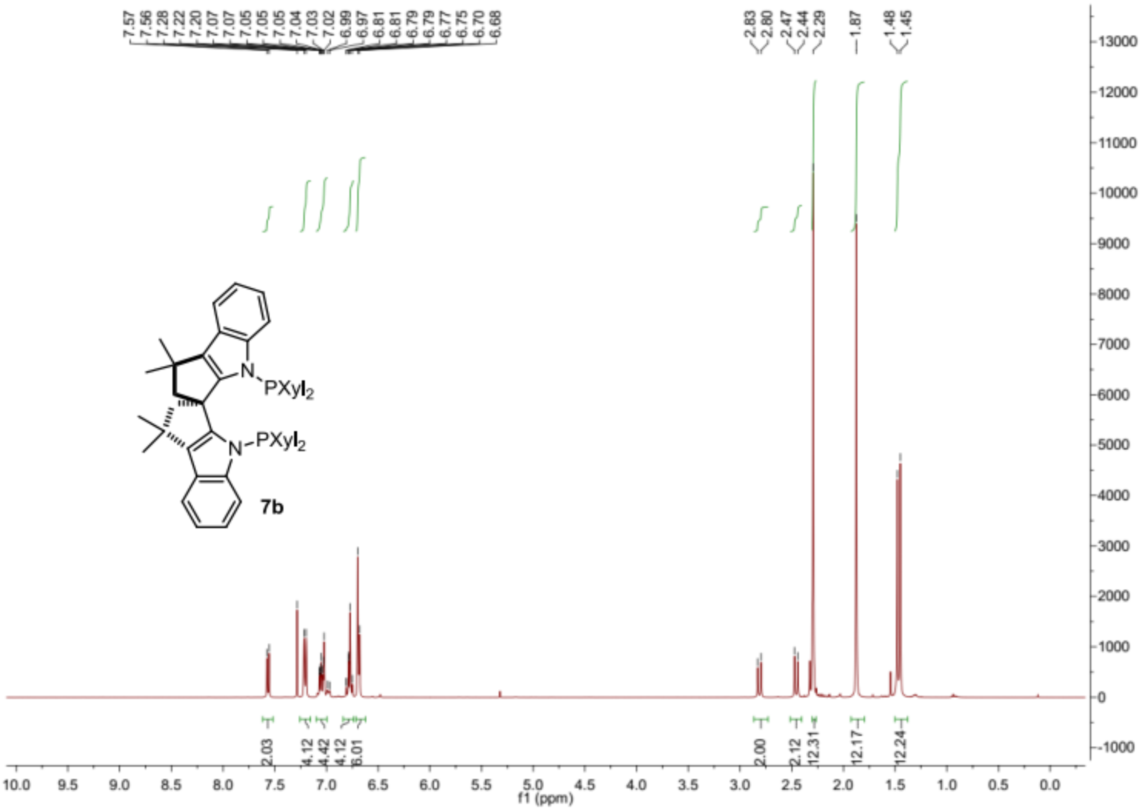

^{1}H NMR (400 MHz, $CDCl_3$, rt)

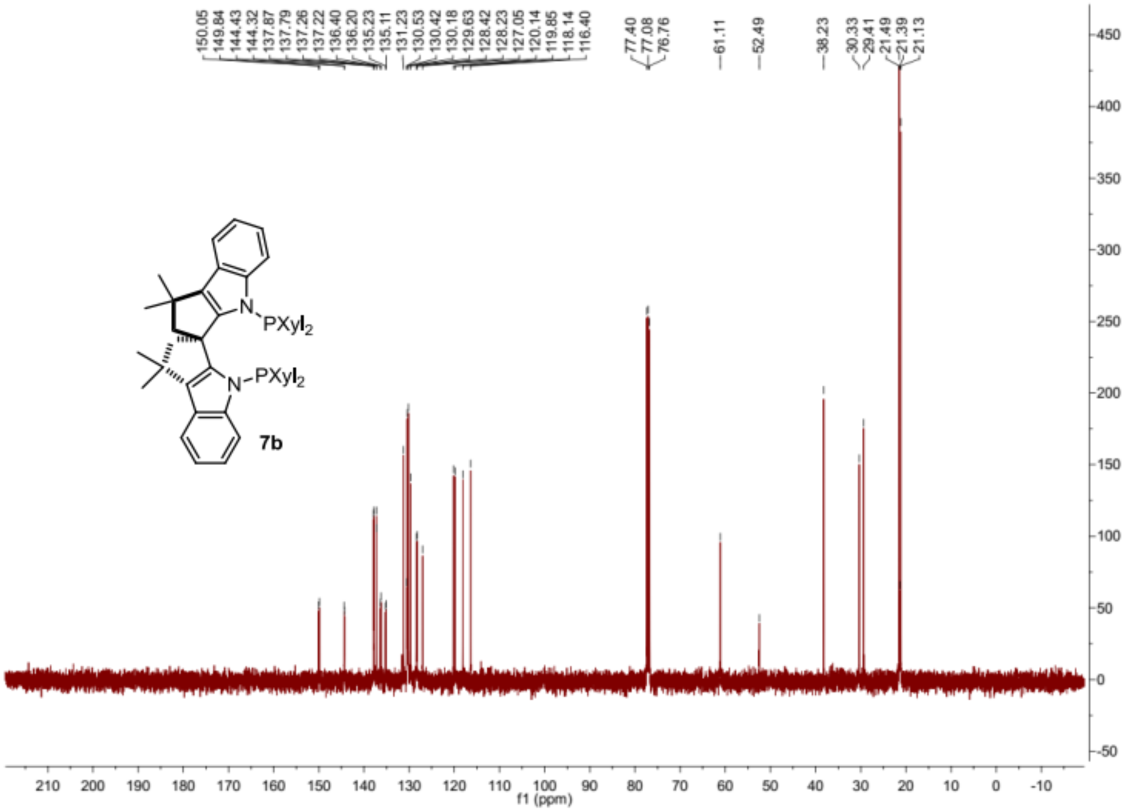

^{13}C NMR (101 MHz, $CDCl_3$, rt)

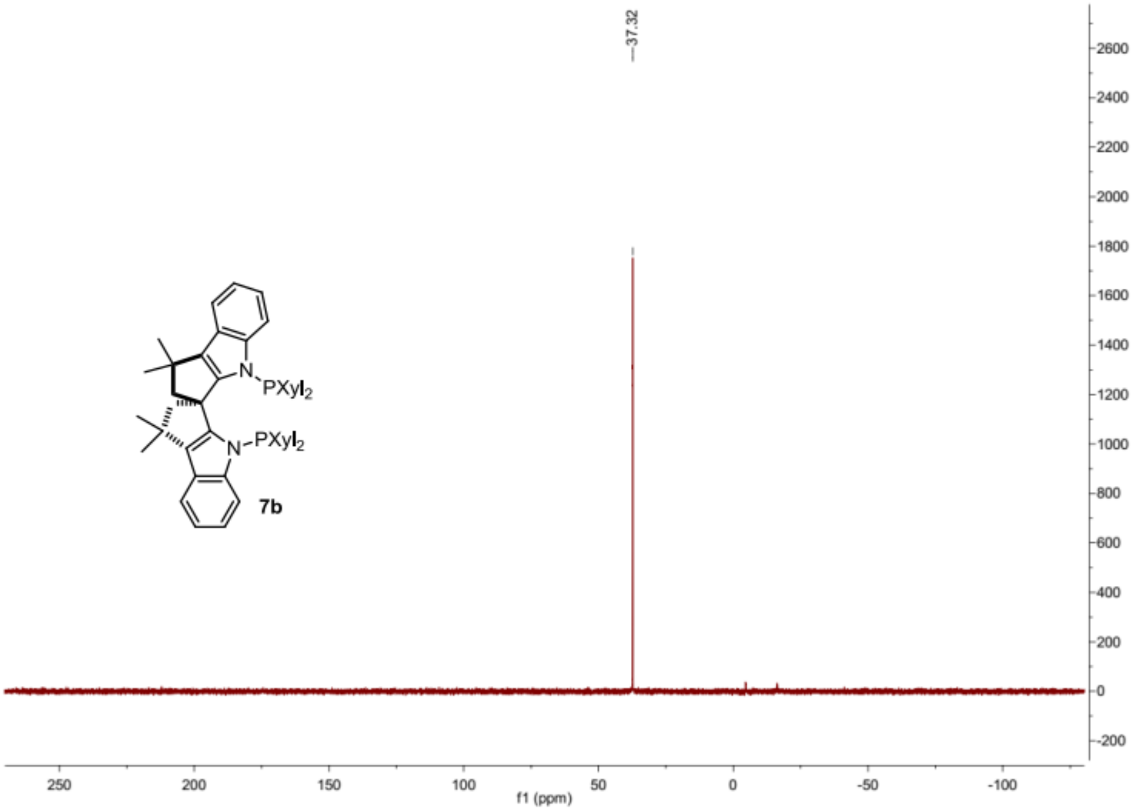

^{31}P NMR (121 MHz, $CDCl_3$, rt)

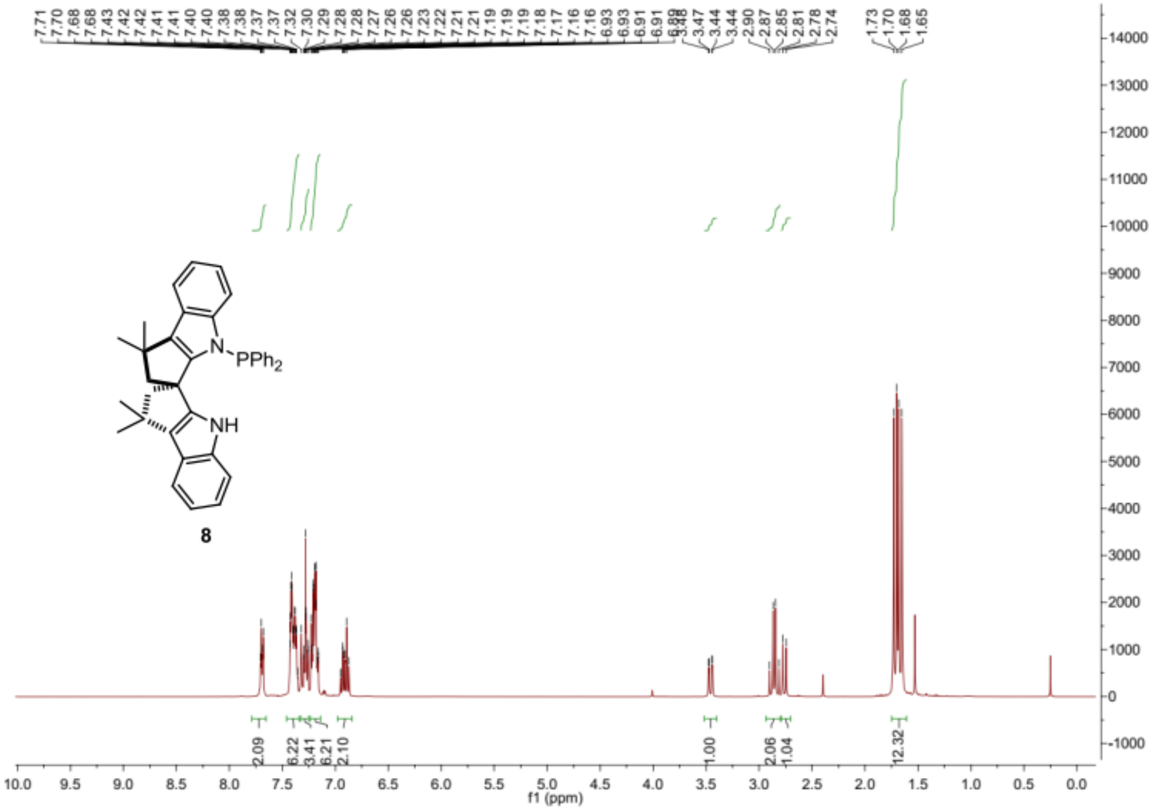

^{1}H NMR (400 MHz, $CDCl_3$, rt)

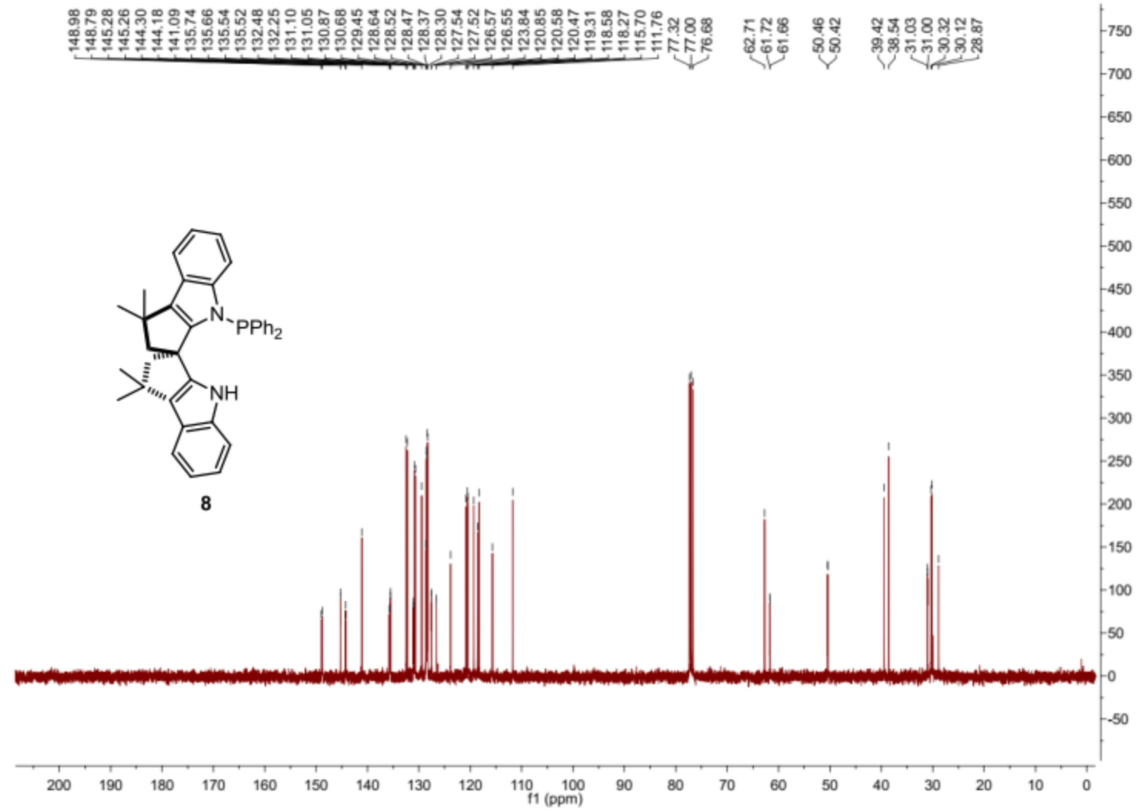

^{13}C NMR (101 MHz, $CDCl_3$, rt)

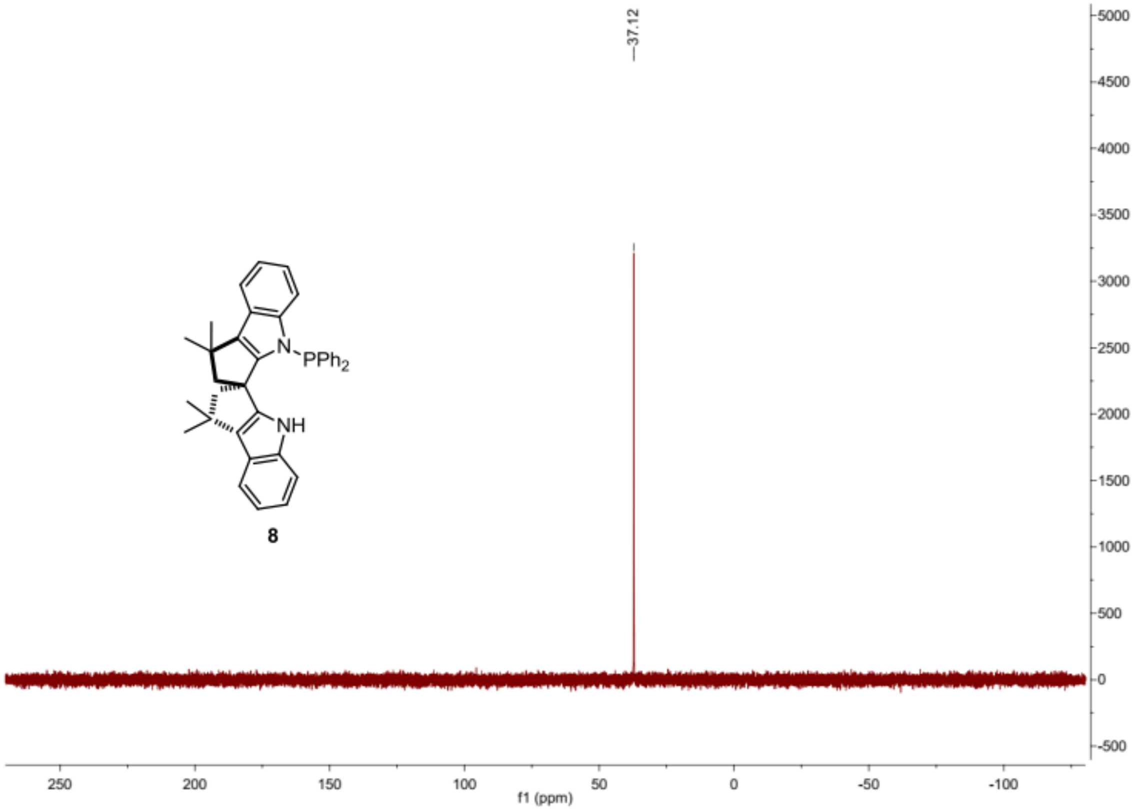

^{31}P NMR (121 MHz, $CDCl_3$, rt)

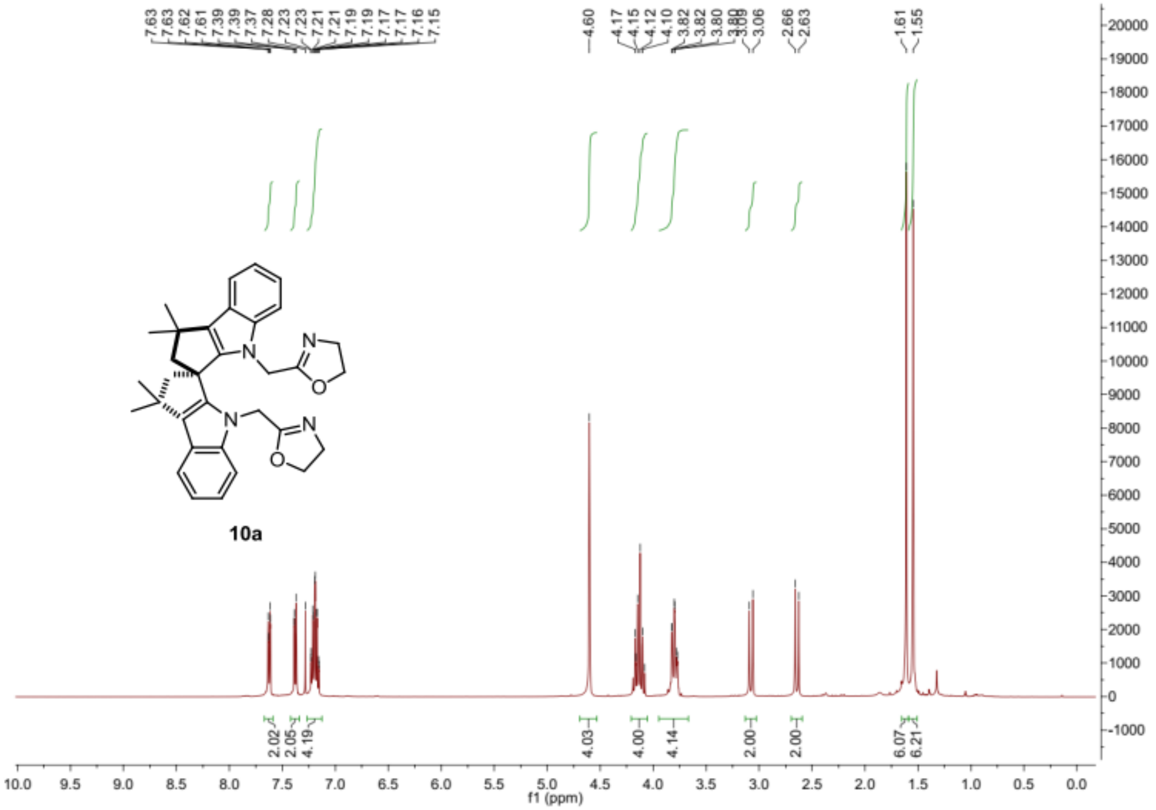

^{1}H NMR (400 MHz, $CDCl_3$, rt)

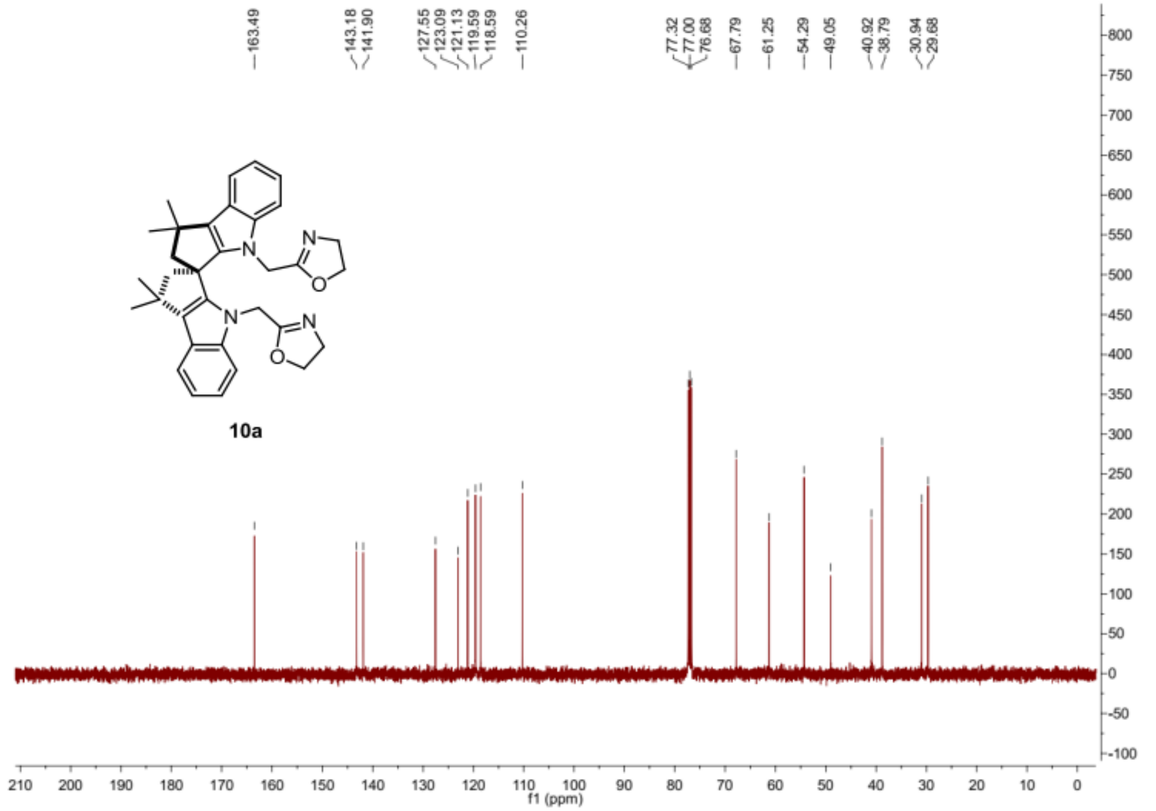

^{13}C NMR (101 MHz, $CDCl_3$, rt)

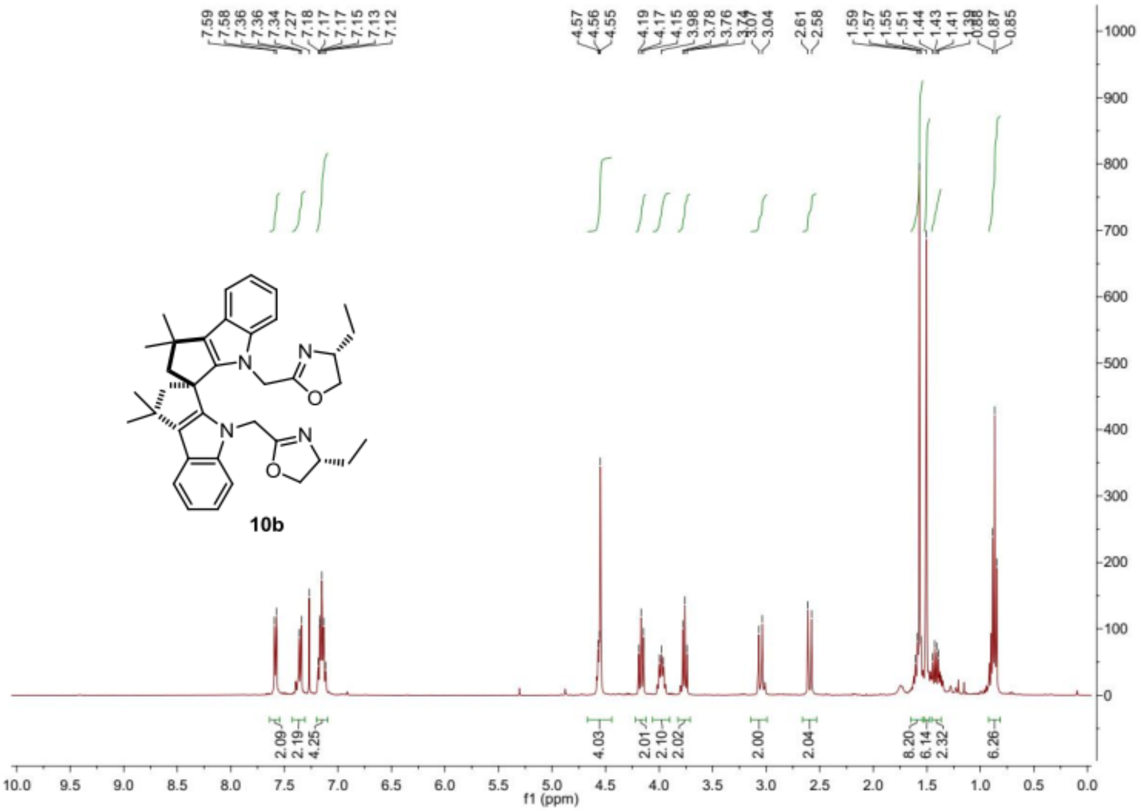

^{1}H NMR (400 MHz, $CDCl_3$, rt)

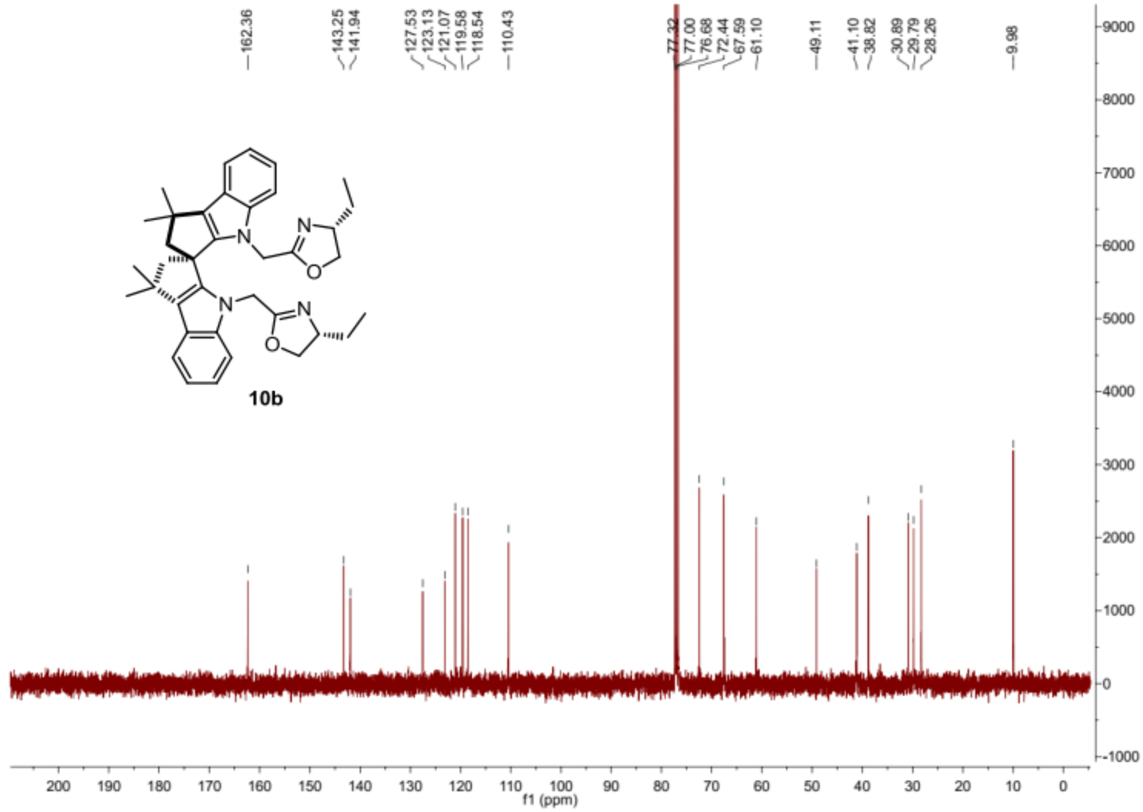

^{13}C NMR (101 MHz, $CDCl_3$, rt)

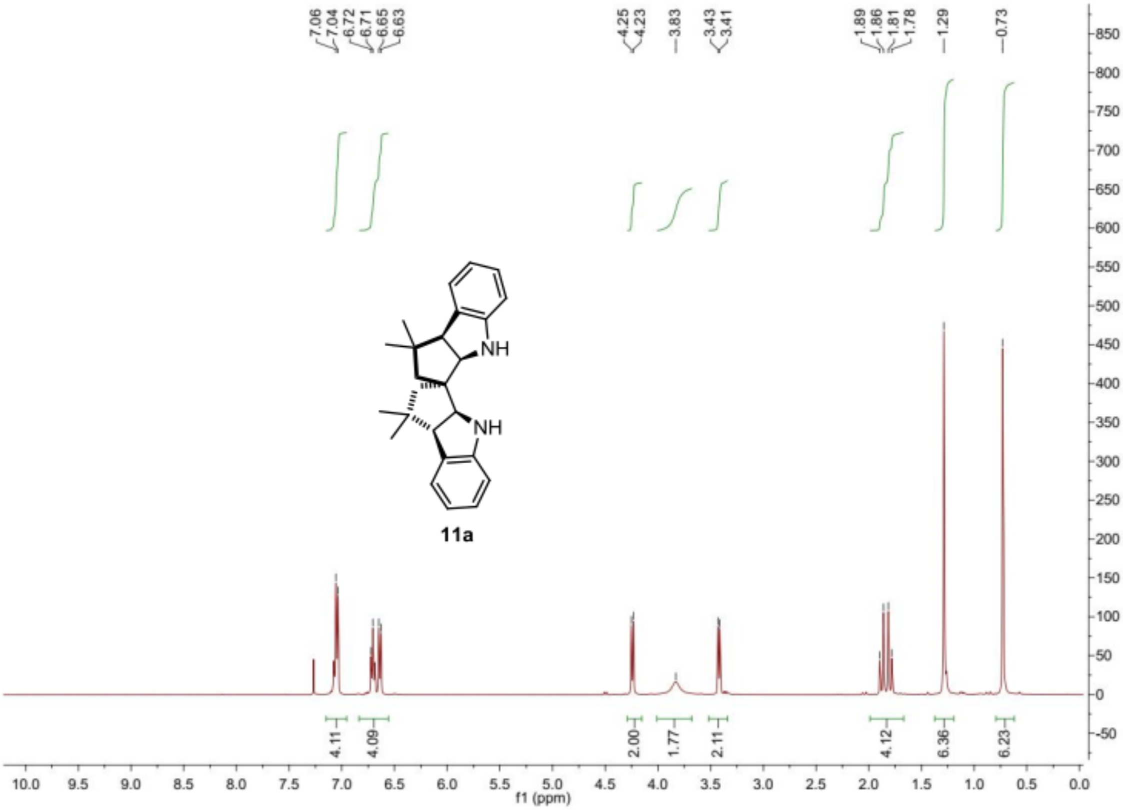

^{1}H NMR (400 MHz, $CDCl_3$, rt)

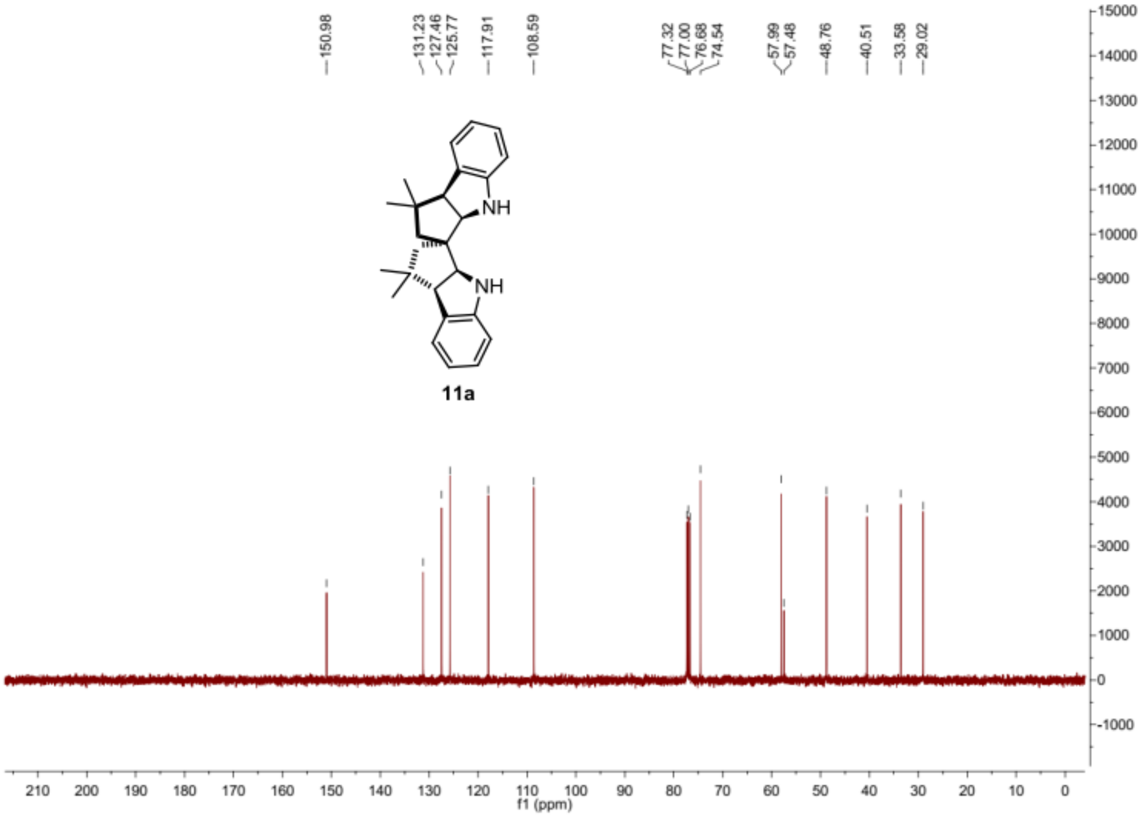

^{13}C NMR (101 MHz, $CDCl_3$, rt)

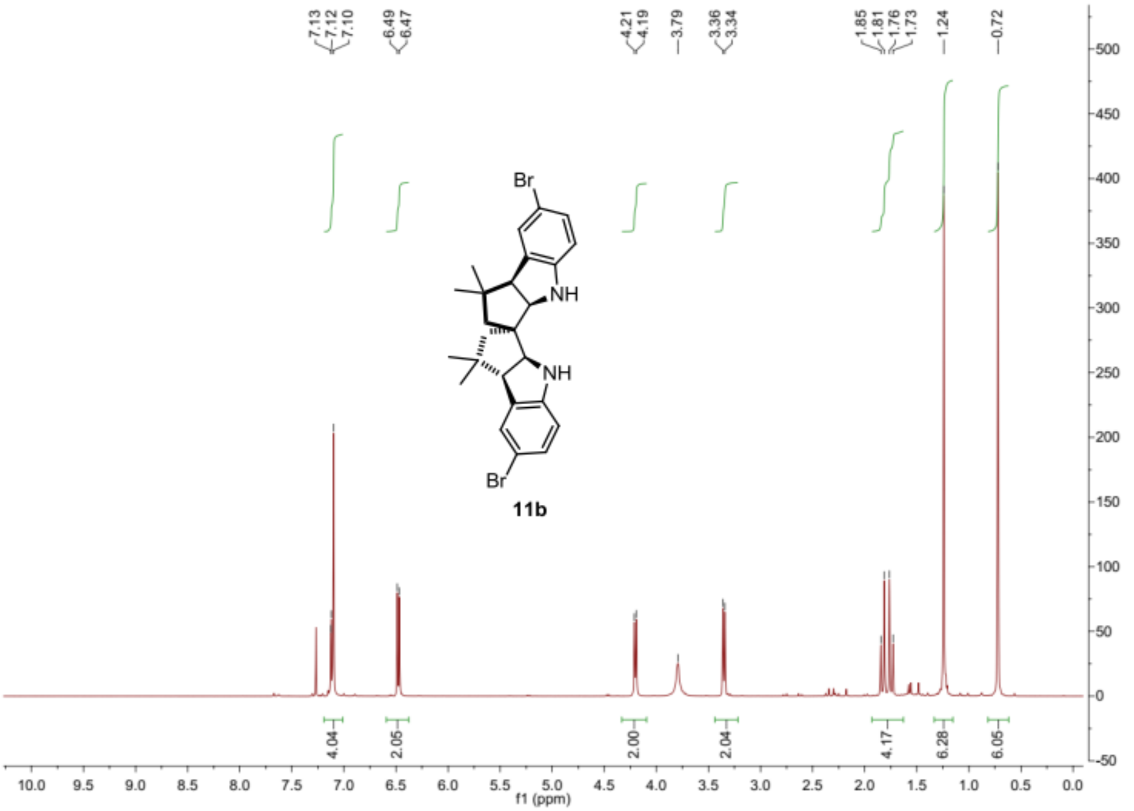

^{1}H NMR (400 MHz, $CDCl_3$, rt)

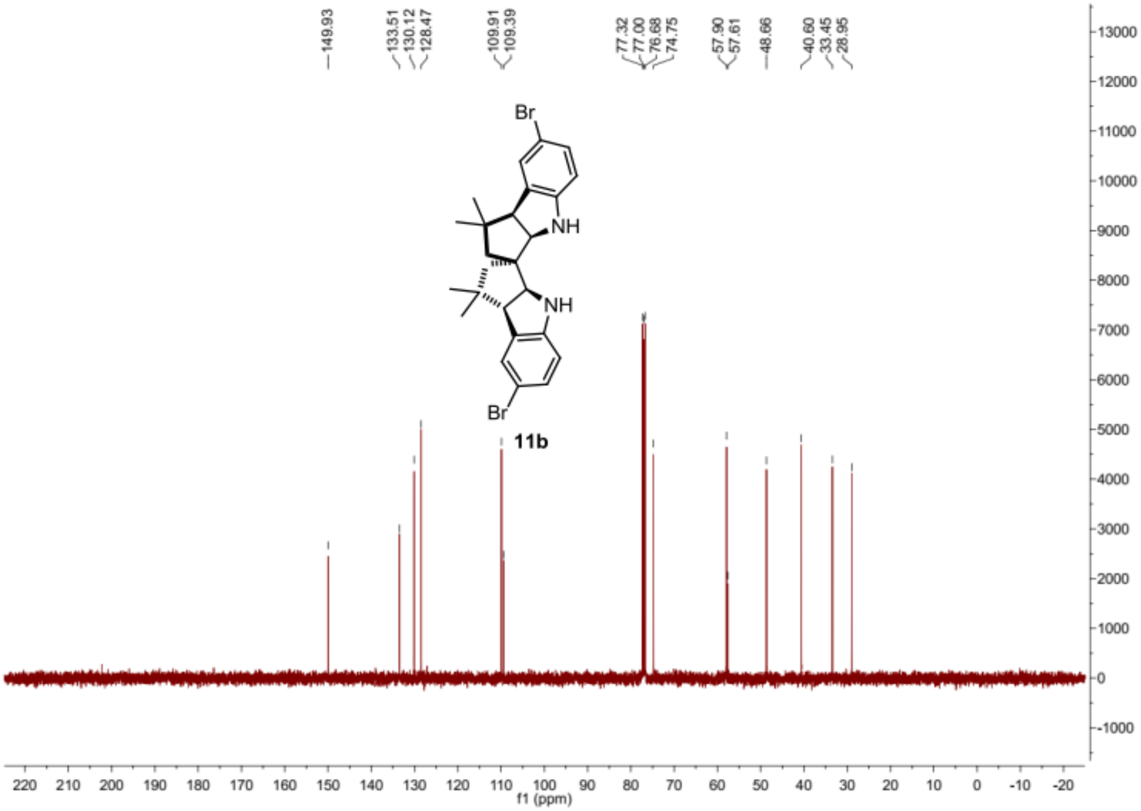

^{13}C NMR (101 MHz, $CDCl_3$, rt)

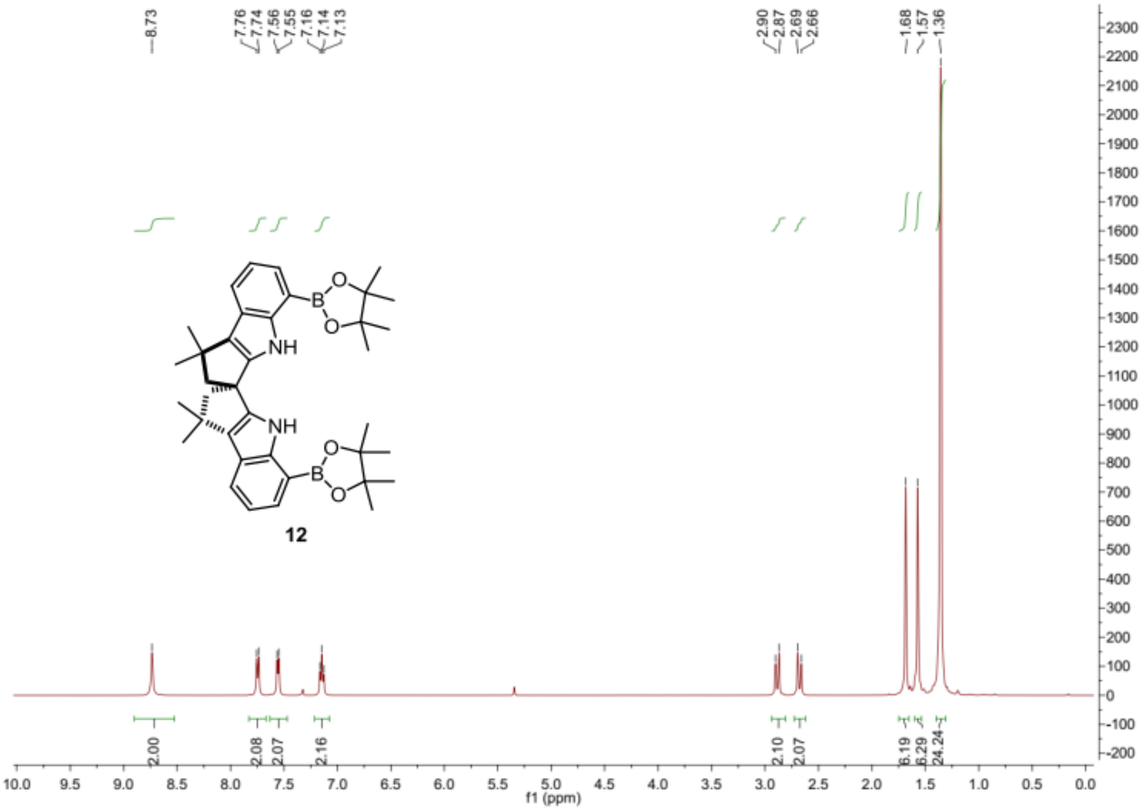

^1H NMR (400 MHz, CD_2Cl_2, rt)

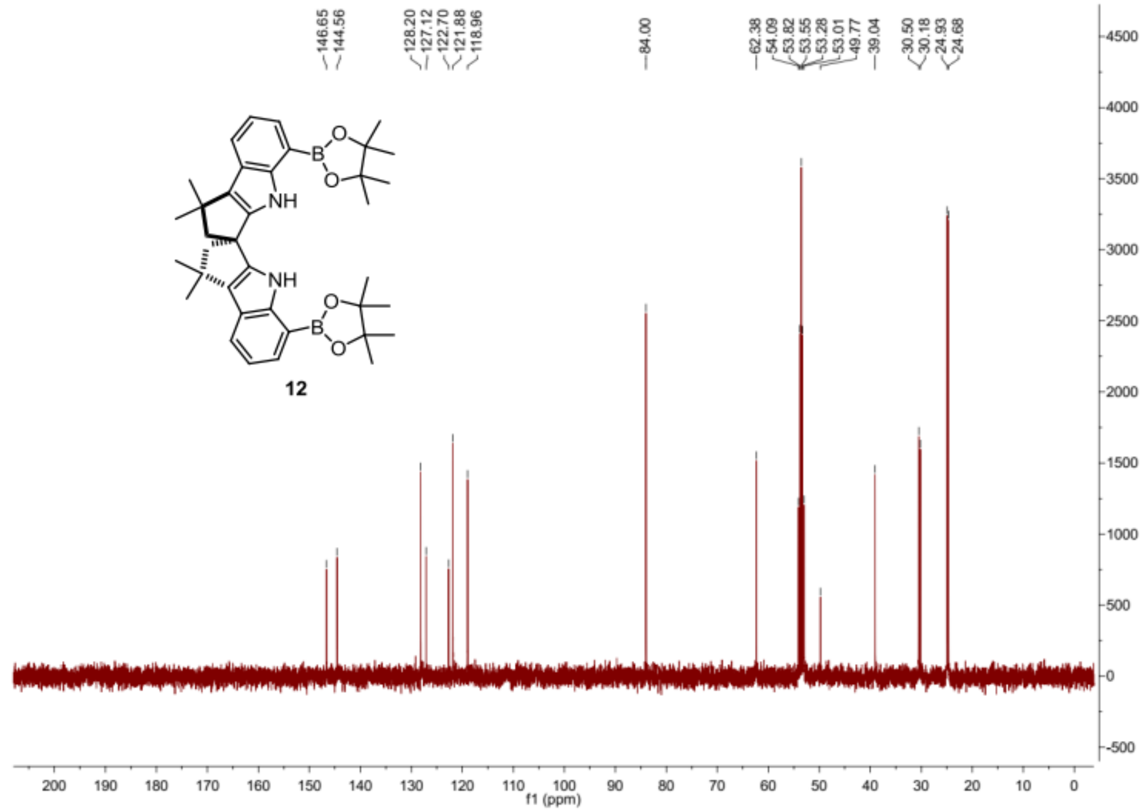

^{13}C NMR (101 MHz, CD_2Cl_2, rt)

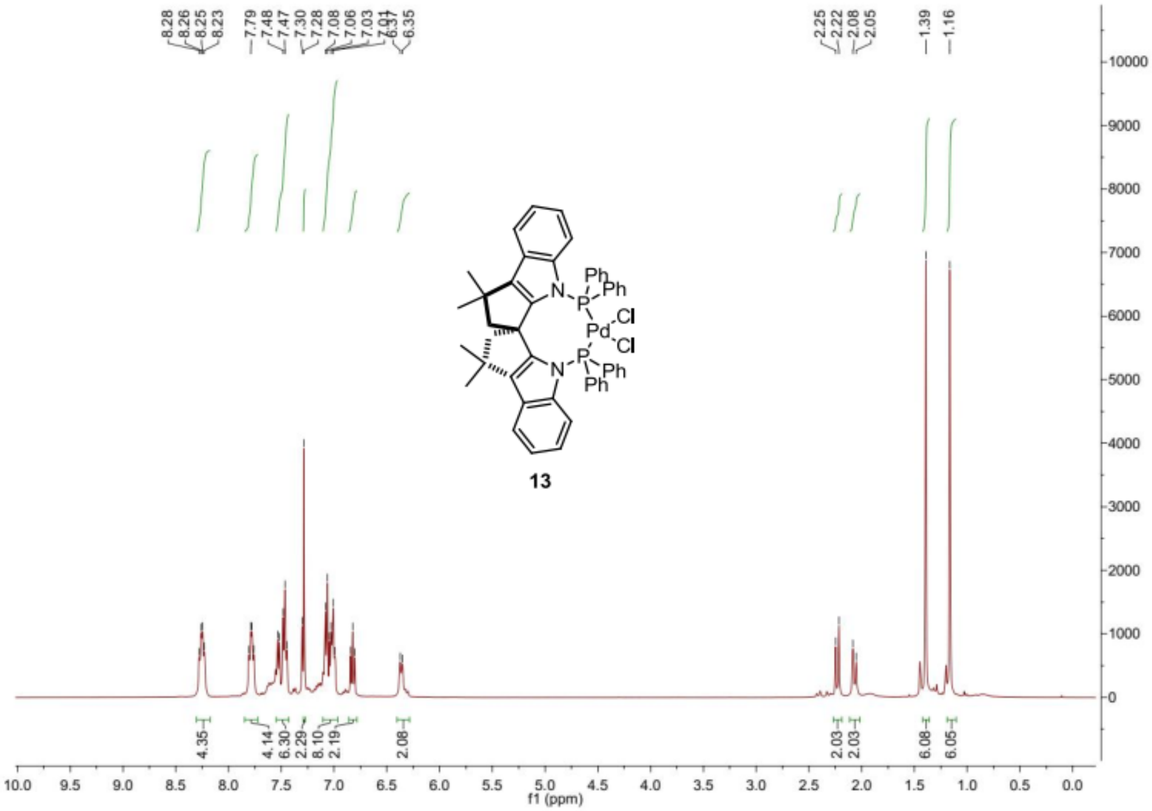

^{1}H NMR (400 MHz, $CDCl_3$, rt)

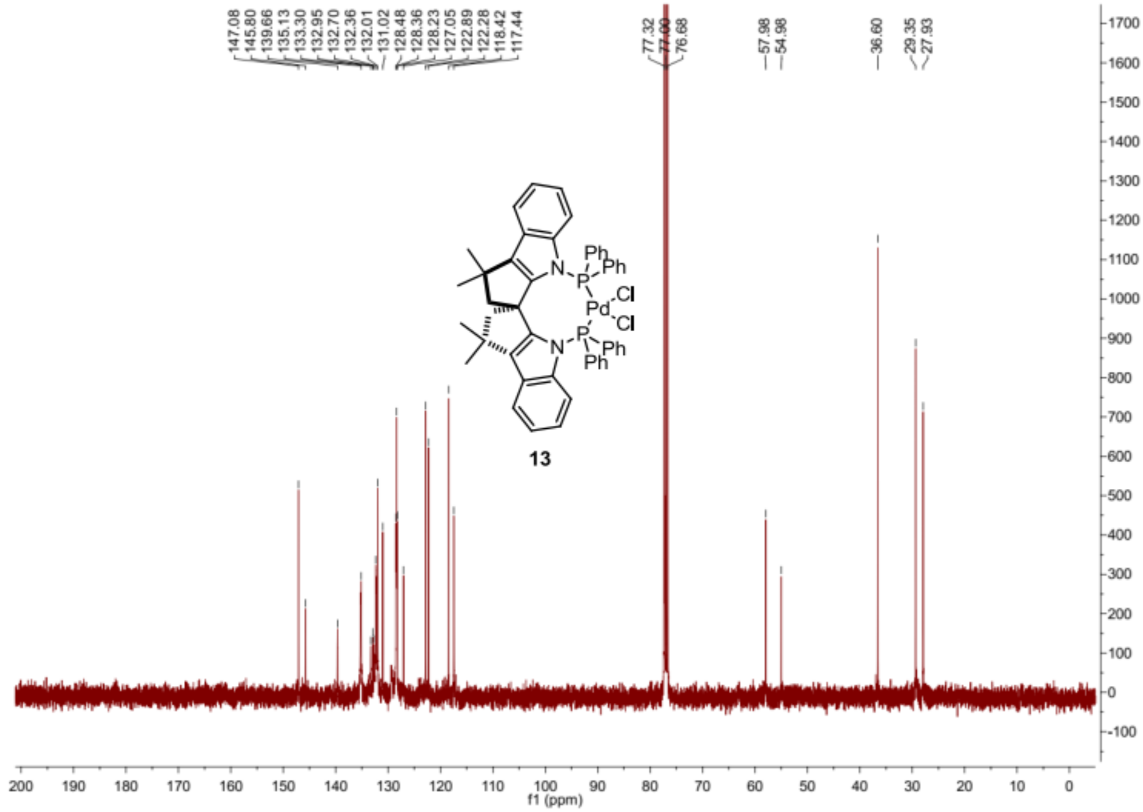

^{13}C NMR (101 MHz, $CDCl_3$, rt)

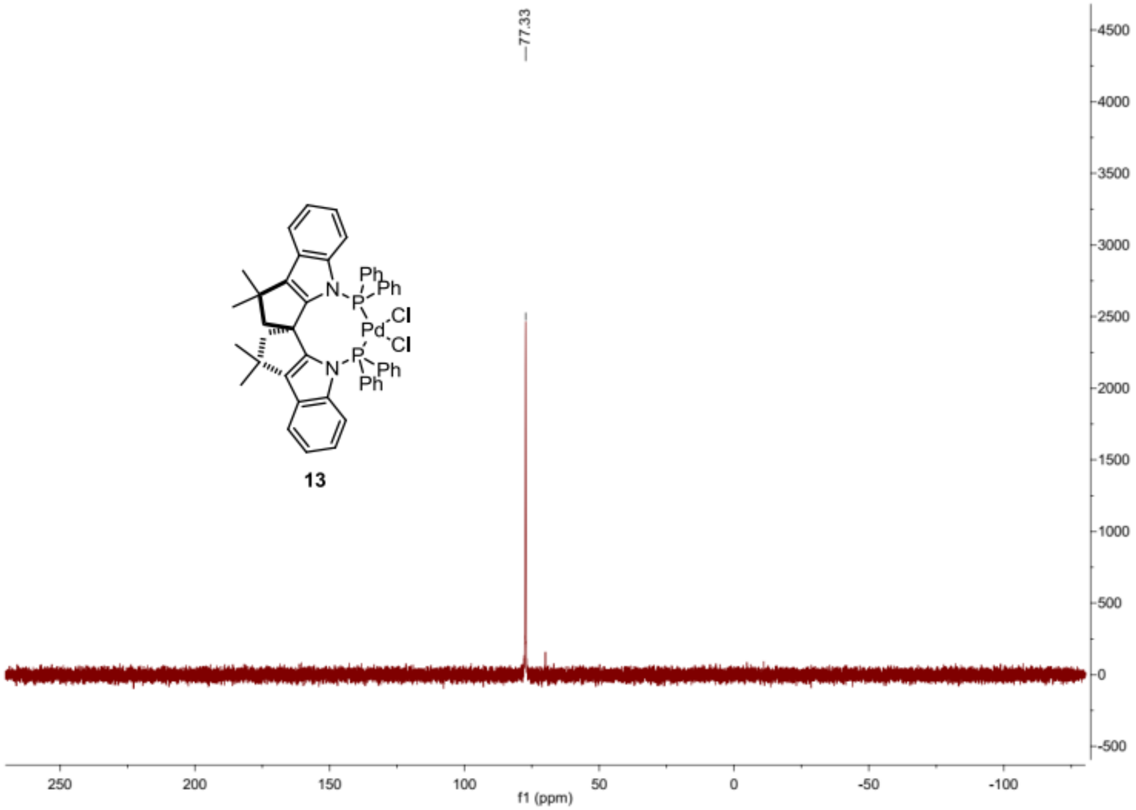

^{31}P NMR (121 MHz, $CDCl_3$, rt)

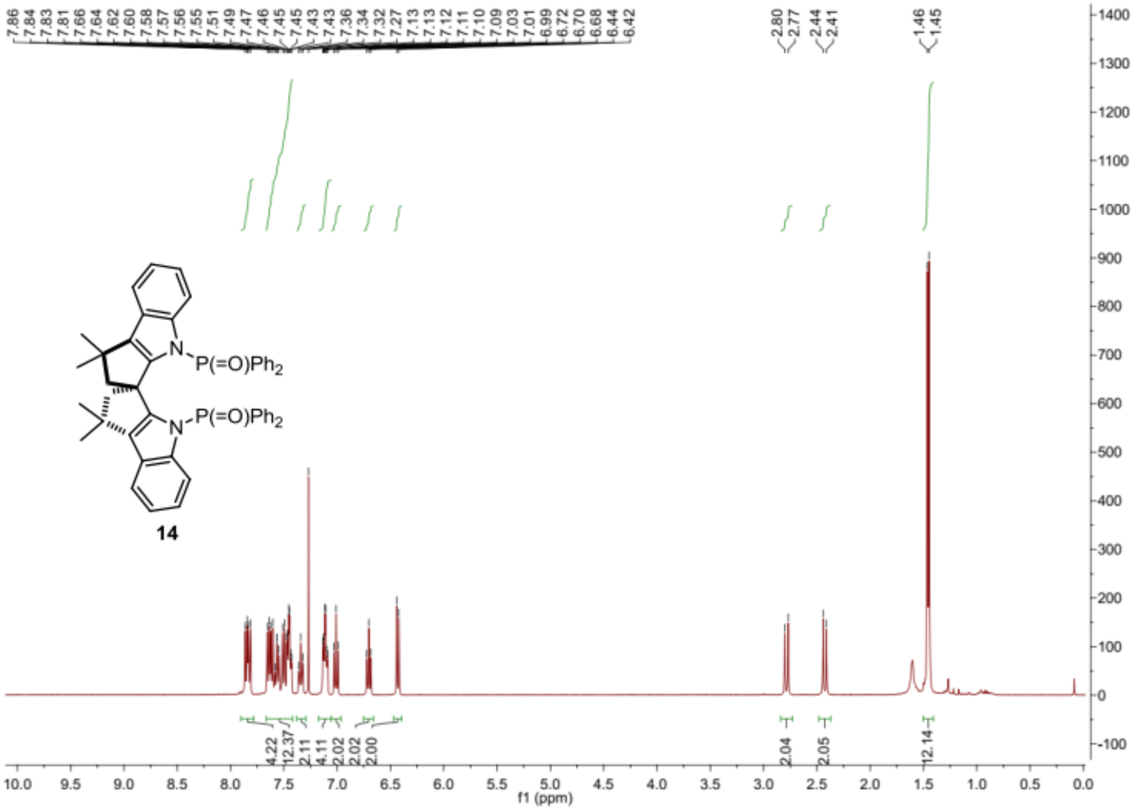

^{1}H NMR (400 MHz, CDCl$_3$, rt)

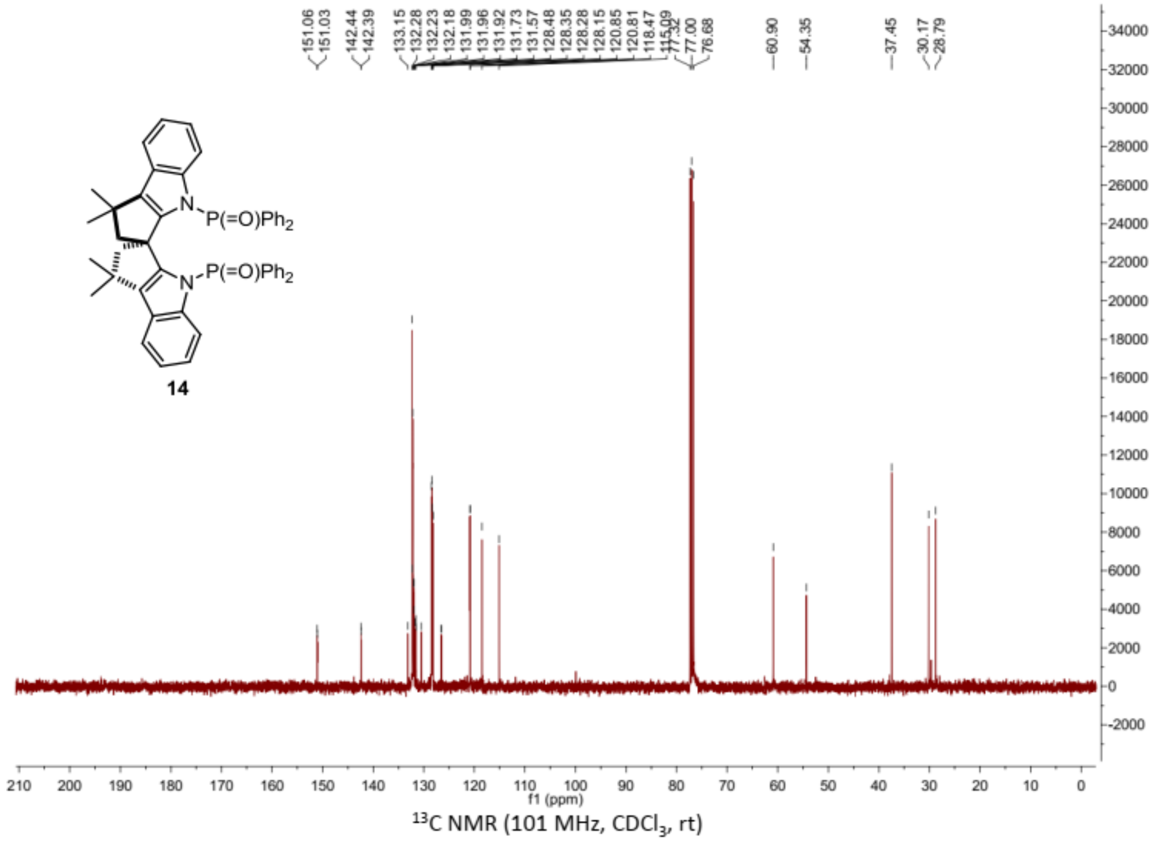

^{13}C NMR (101 MHz, CDCl$_3$, rt)

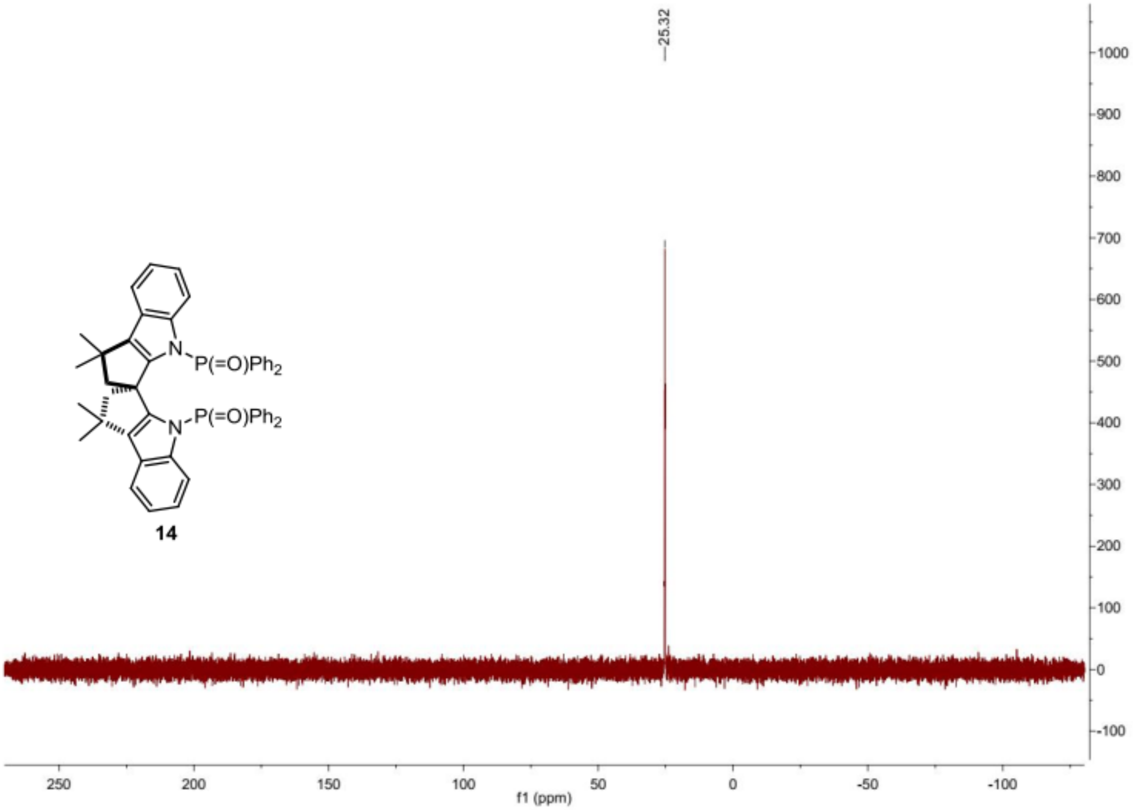

^{31}P NMR (121 MHz, $CDCl_3$, rt)

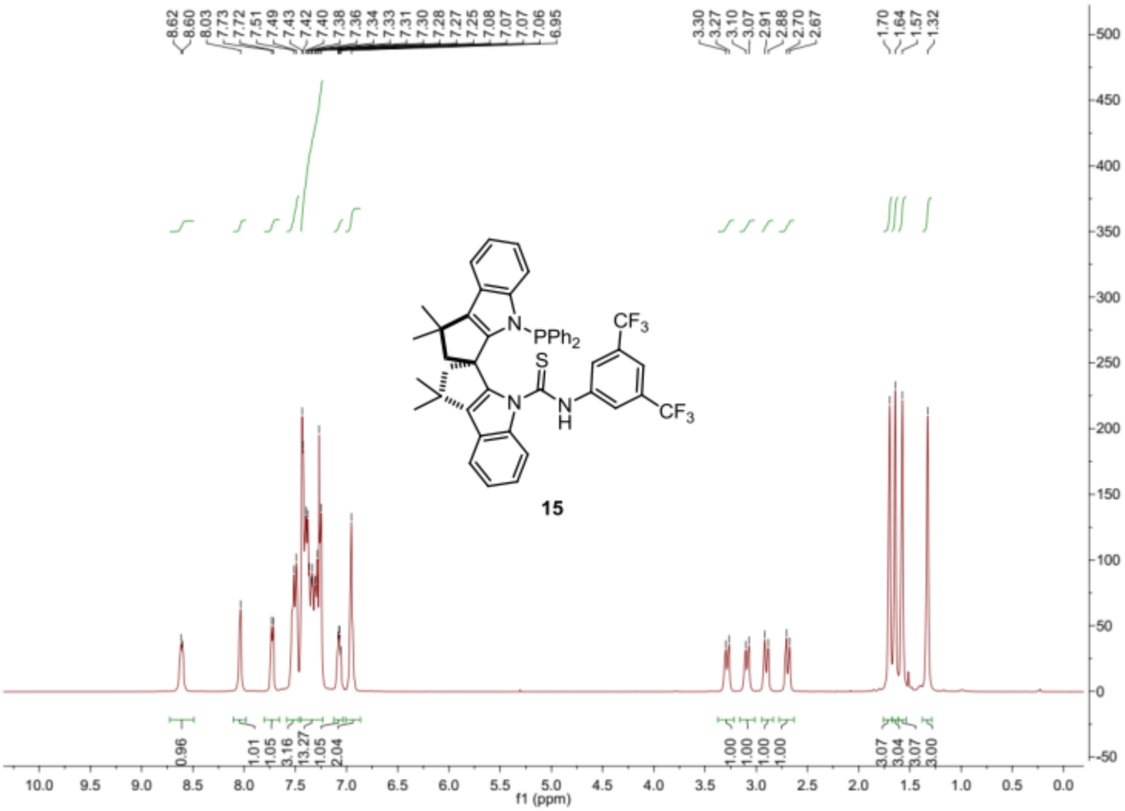

^1H NMR (400 MHz, $CDCl_3$, rt)

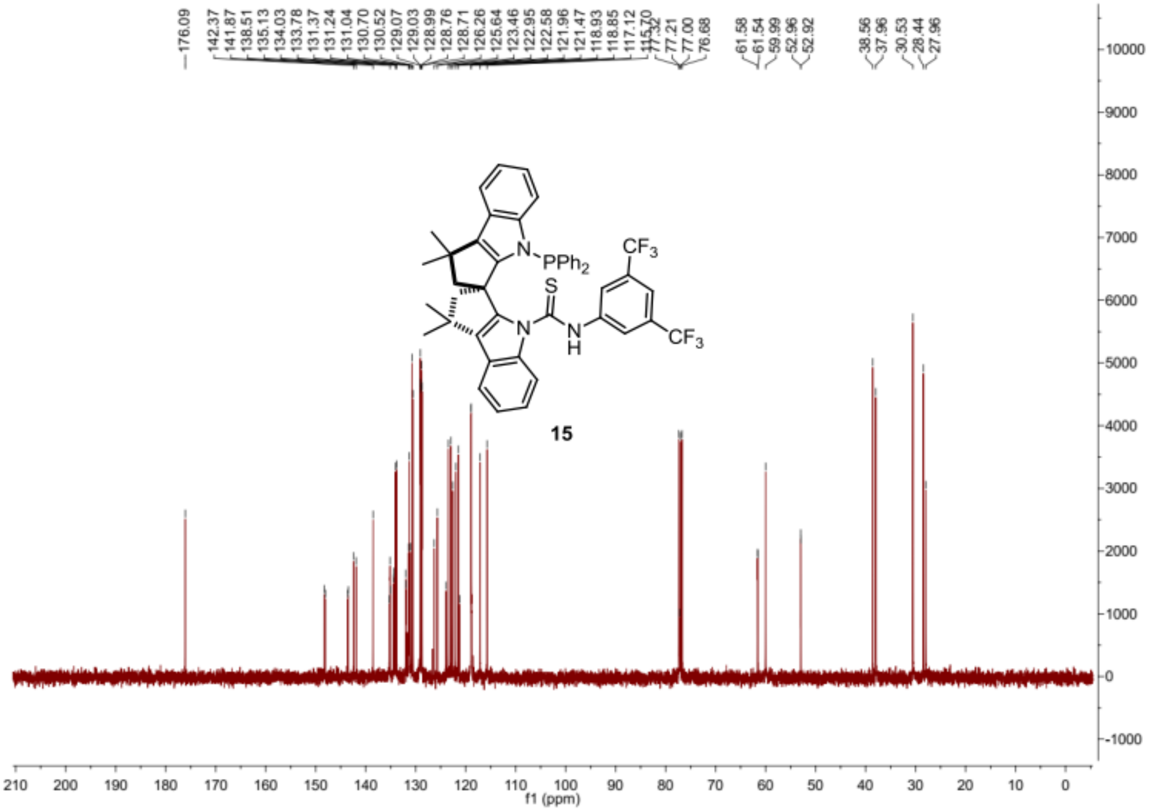

^{13}C NMR (101 MHz, $CDCl_3$, rt)

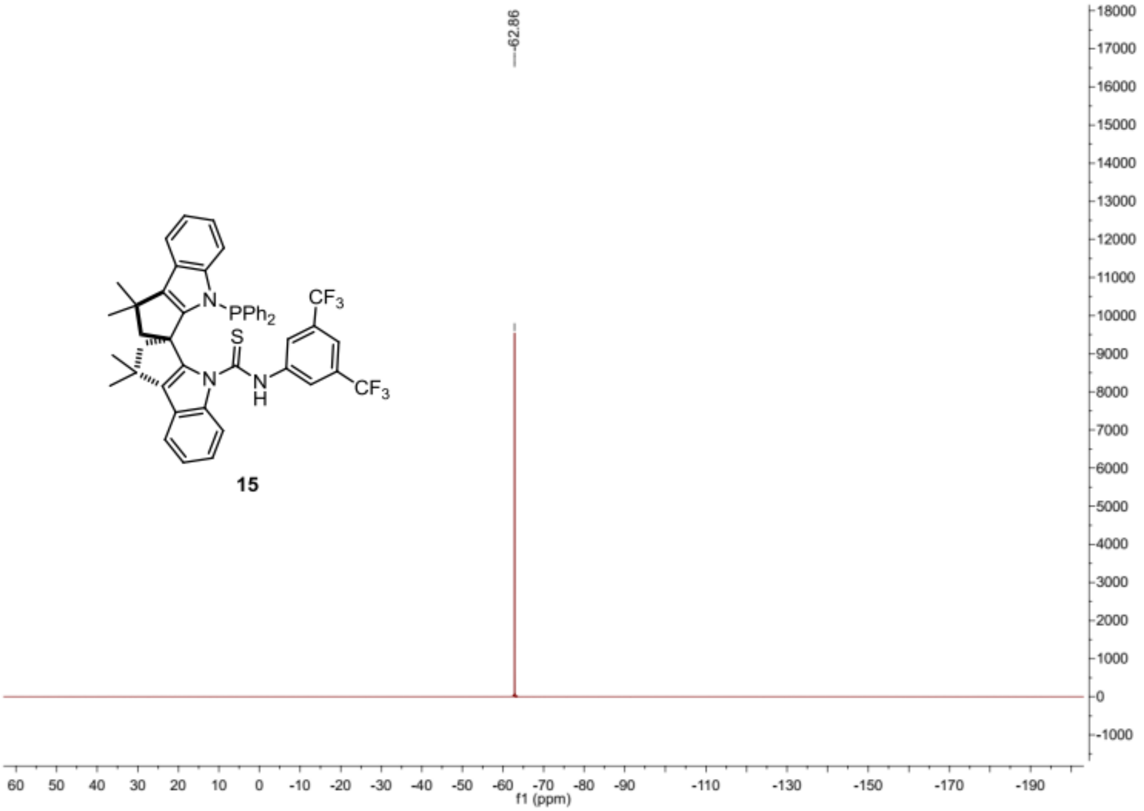

^{19}F NMR (282 MHz, CD_2Cl_2, rt)

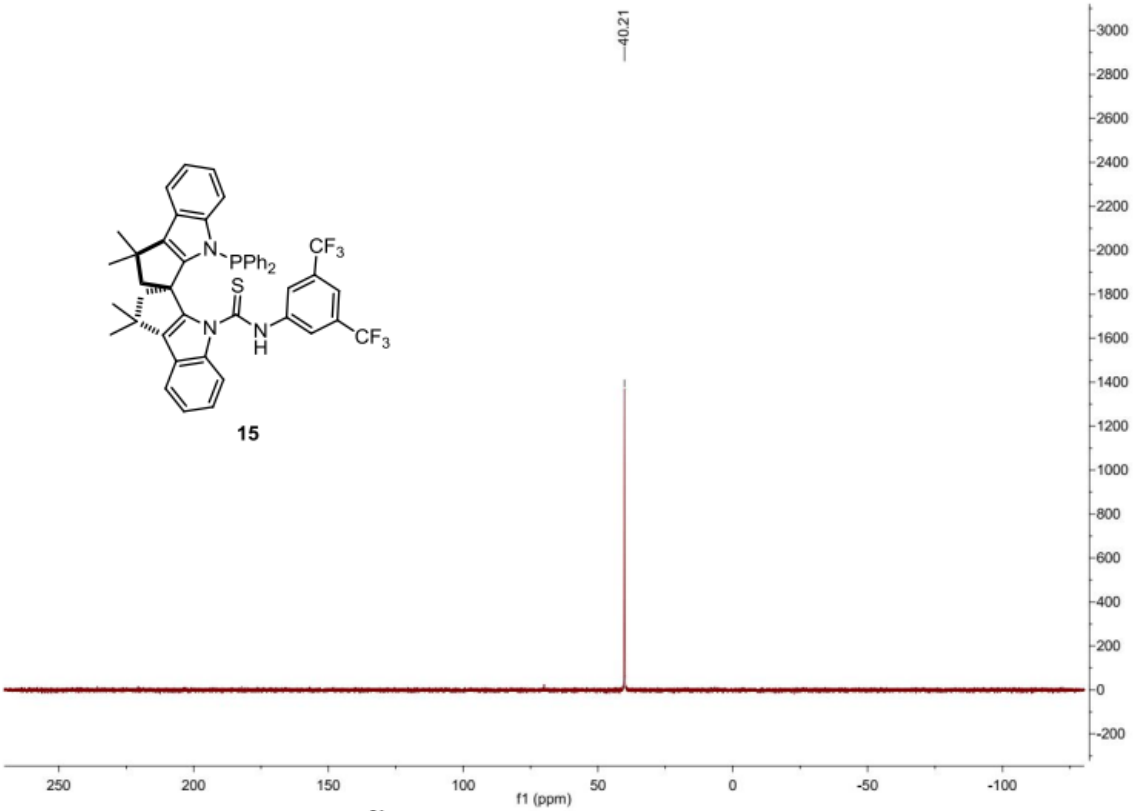

^{31}P NMR (121 MHz, $CDCl_3$, rt)

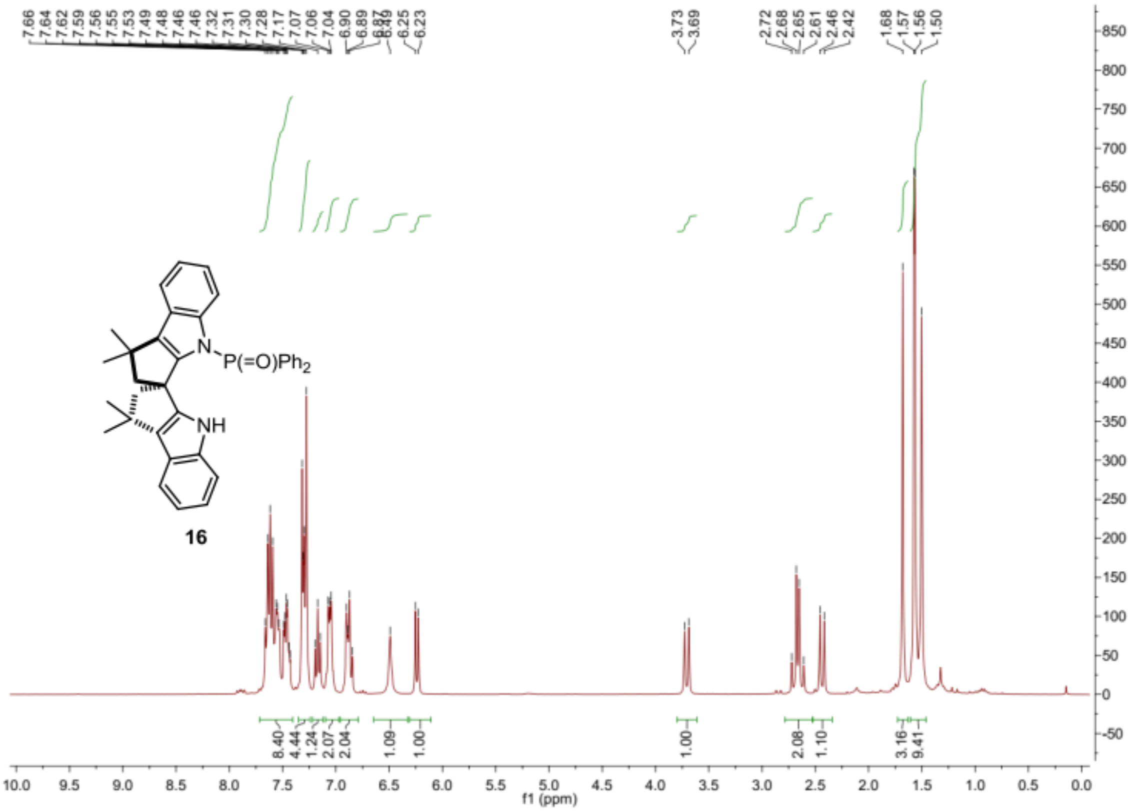

^1H NMR (400 MHz, $CDCl_3$, rt)

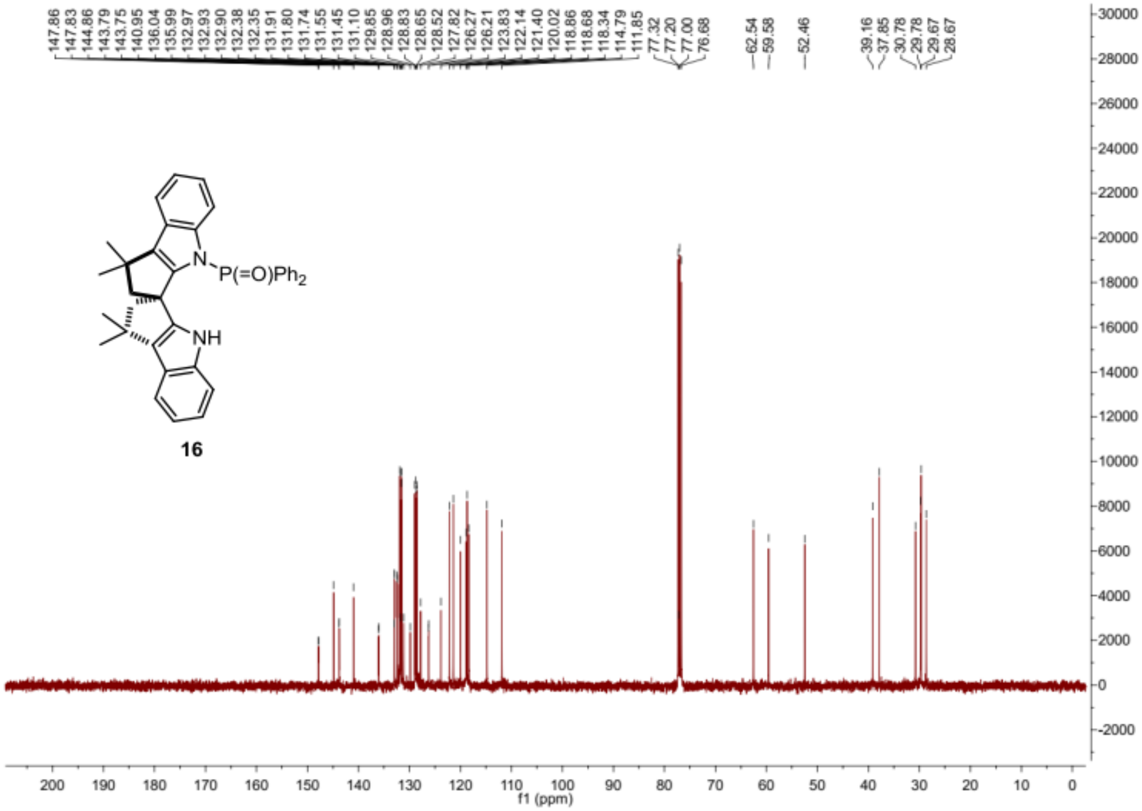

^{13}C NMR (101 MHz, $CDCl_3$, rt)

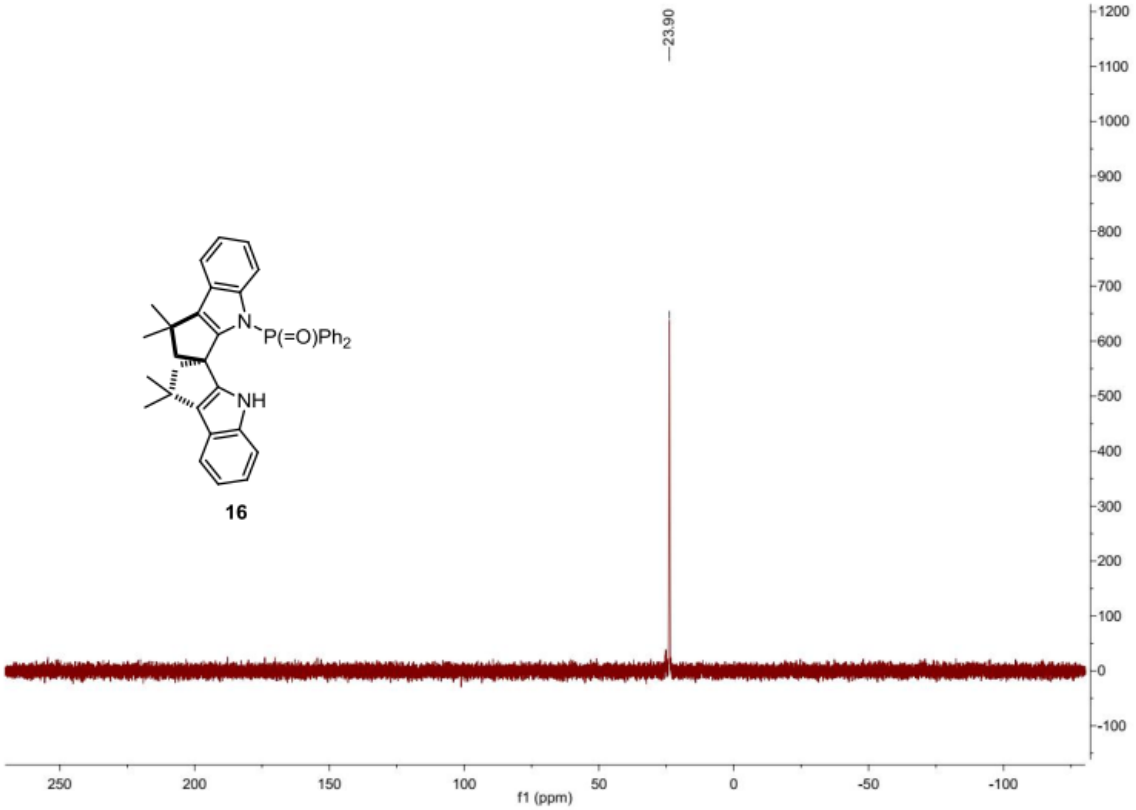

^{31}P NMR (121 MHz, $CDCl_3$, rt)

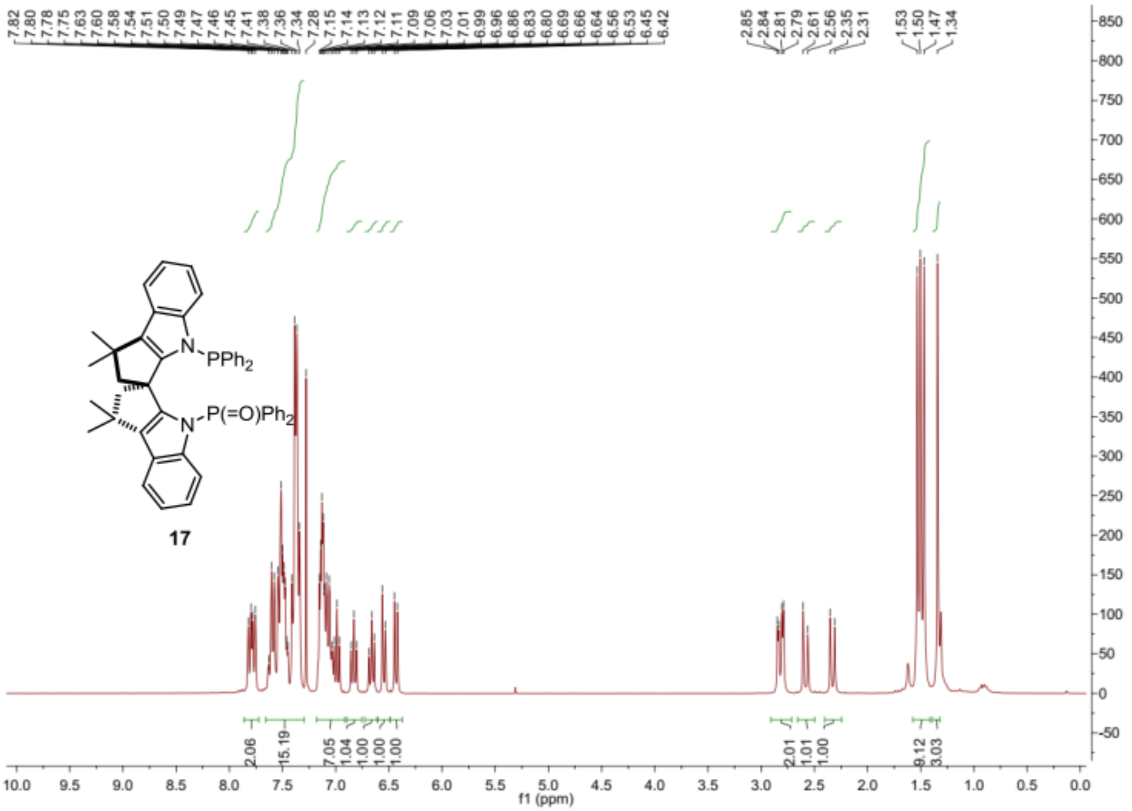

^1H NMR (400 MHz, $CDCl_3$, rt)

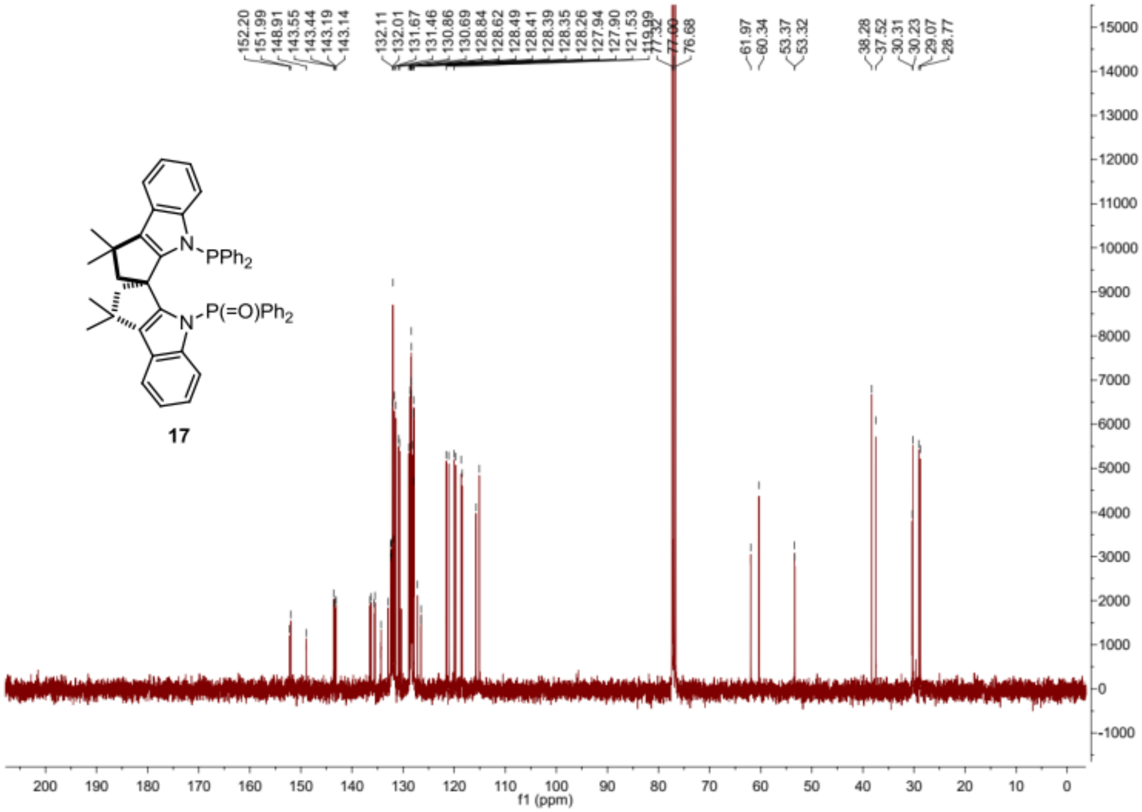

^{13}C NMR (101 MHz, $CDCl_3$, rt)

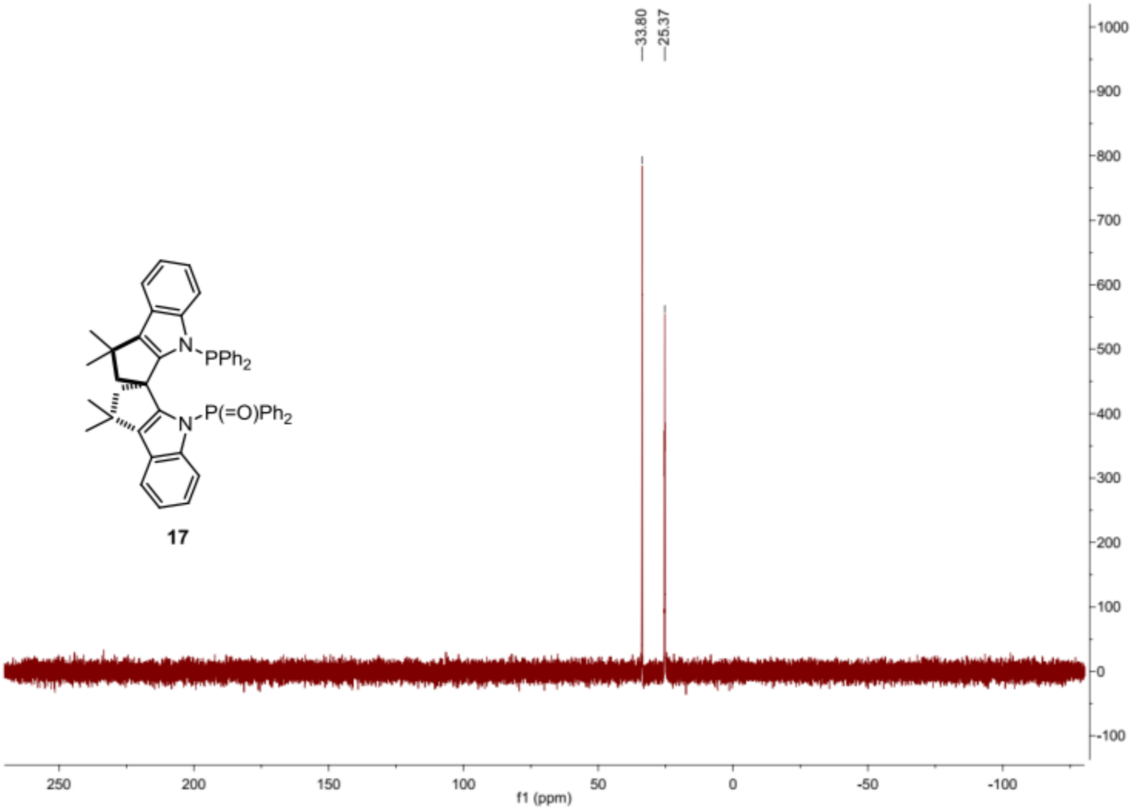

^{31}P NMR (121 MHz, $CDCl_3$, rt)

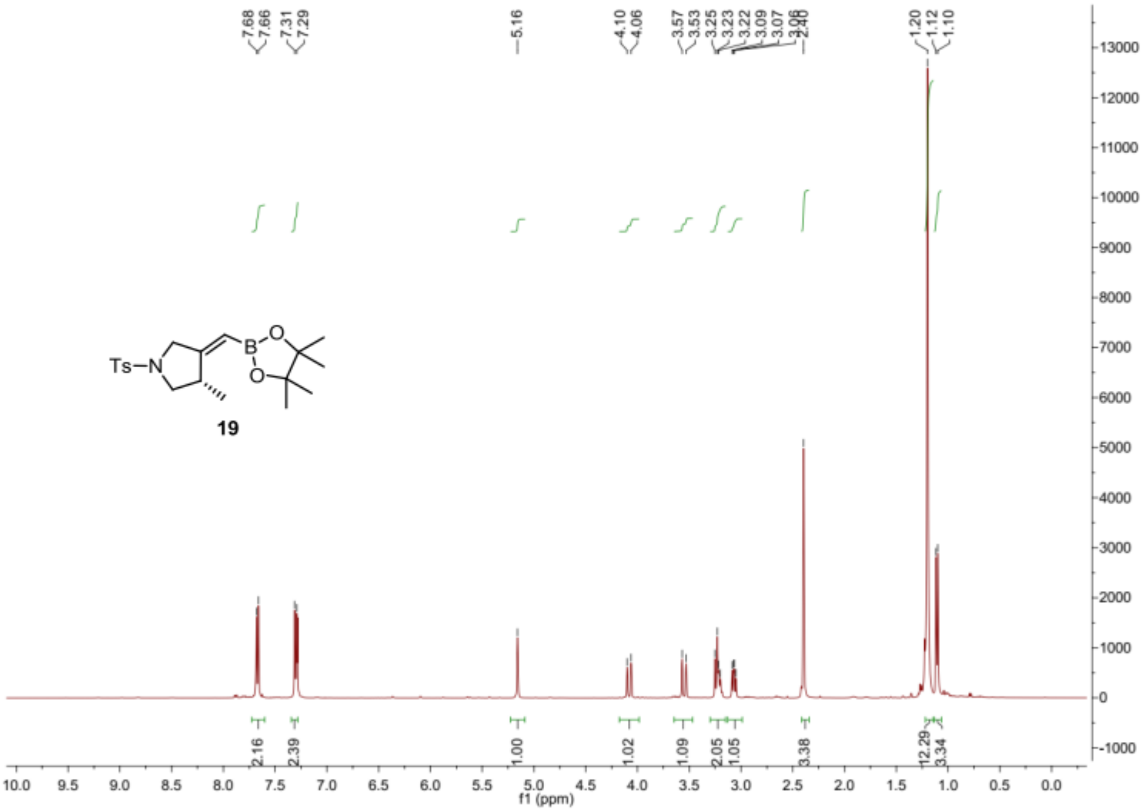

^{1}H NMR (400 MHz, $CDCl_3$, rt)

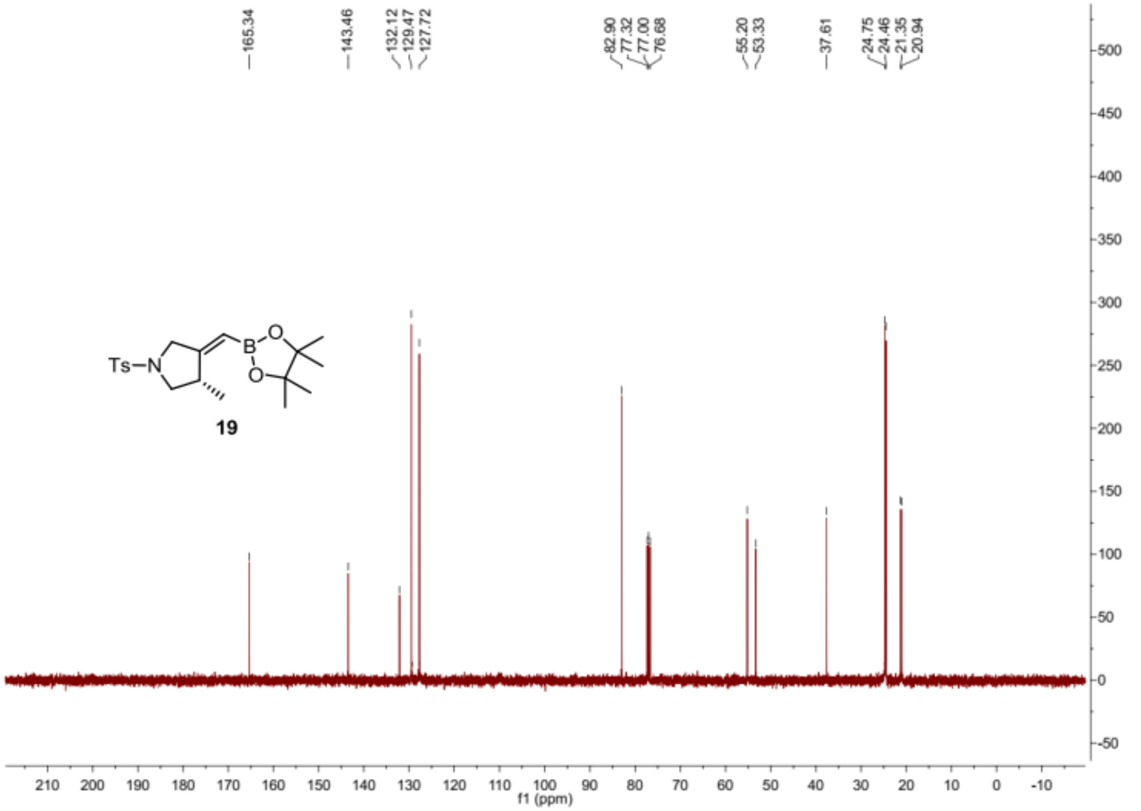

^{13}C NMR (101 MHz, $CDCl_3$, rt)

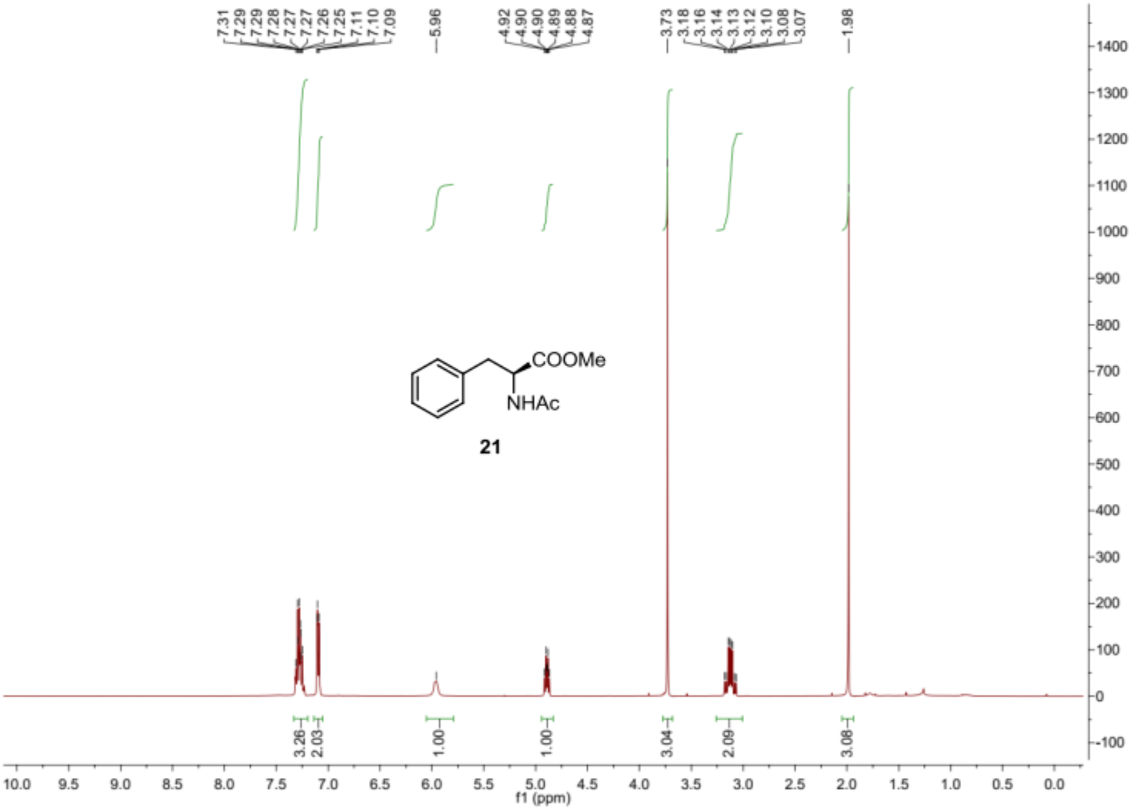

^{1}H NMR (400 MHz, $CDCl_3$, rt)

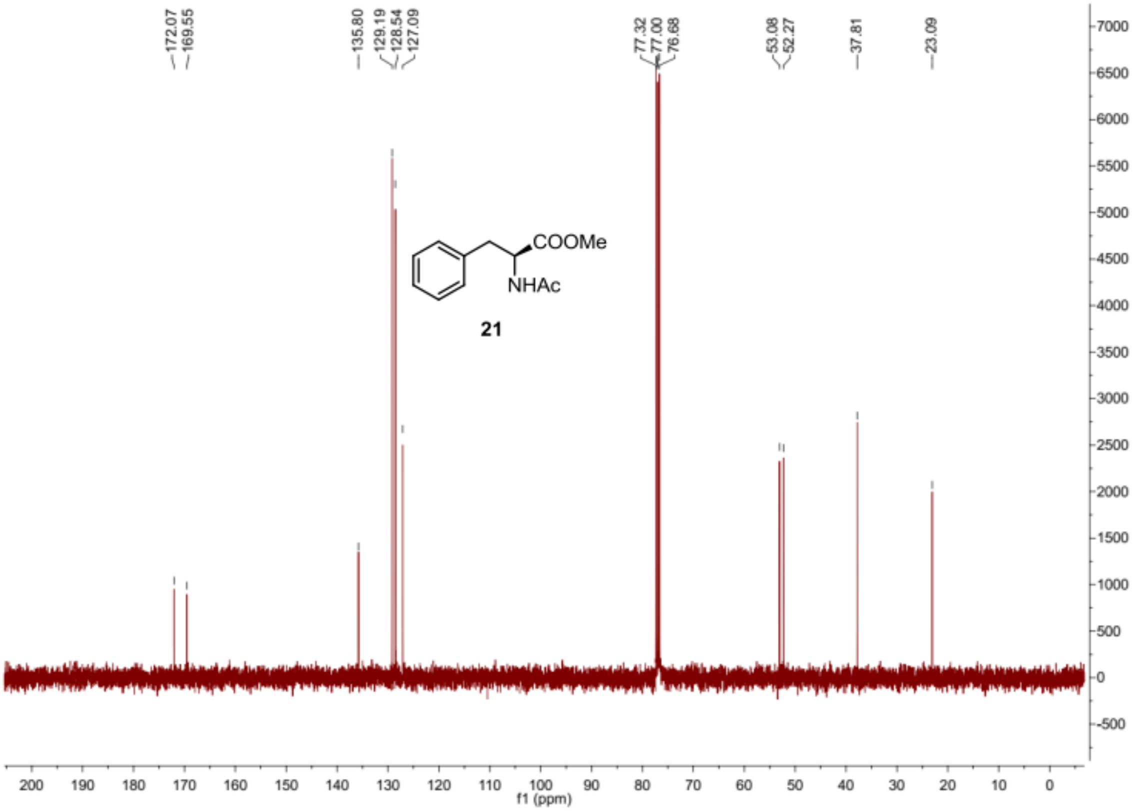

^{13}C NMR (101 MHz, $CDCl_3$, rt)

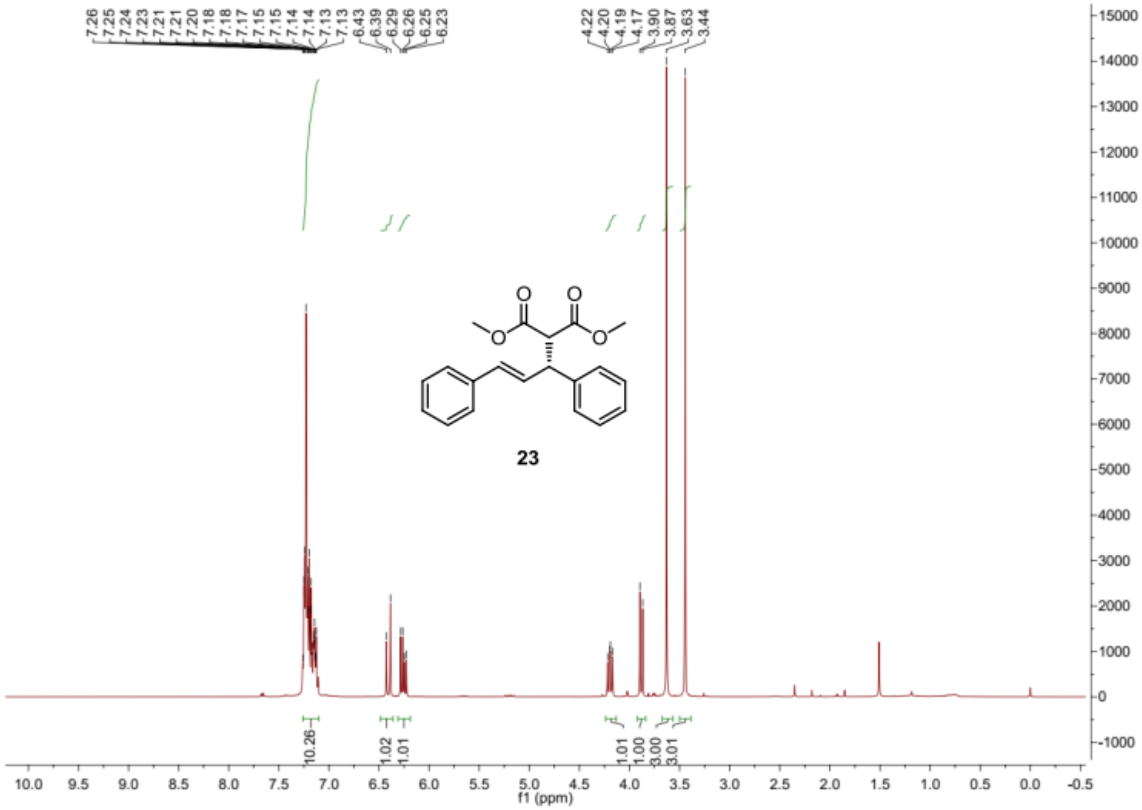

^1H NMR (400 MHz, $CDCl_3$, rt)

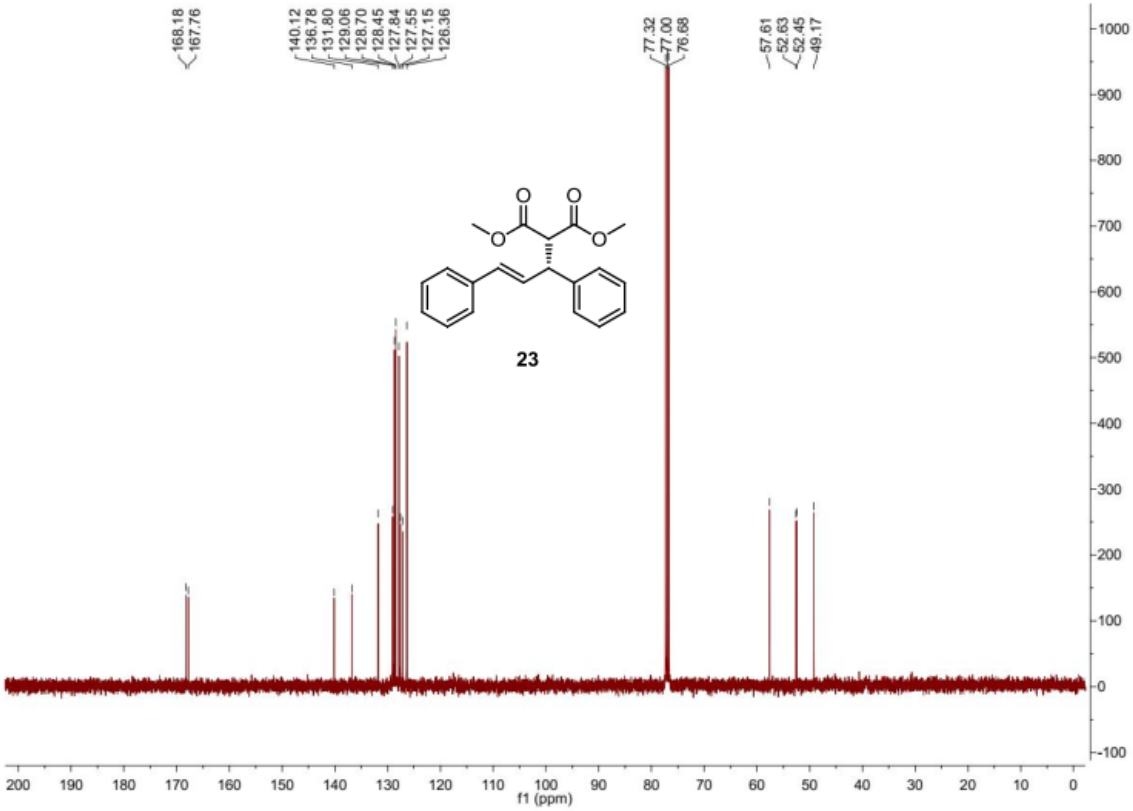

^{13}C NMR (101 MHz, $CDCl_3$, rt)

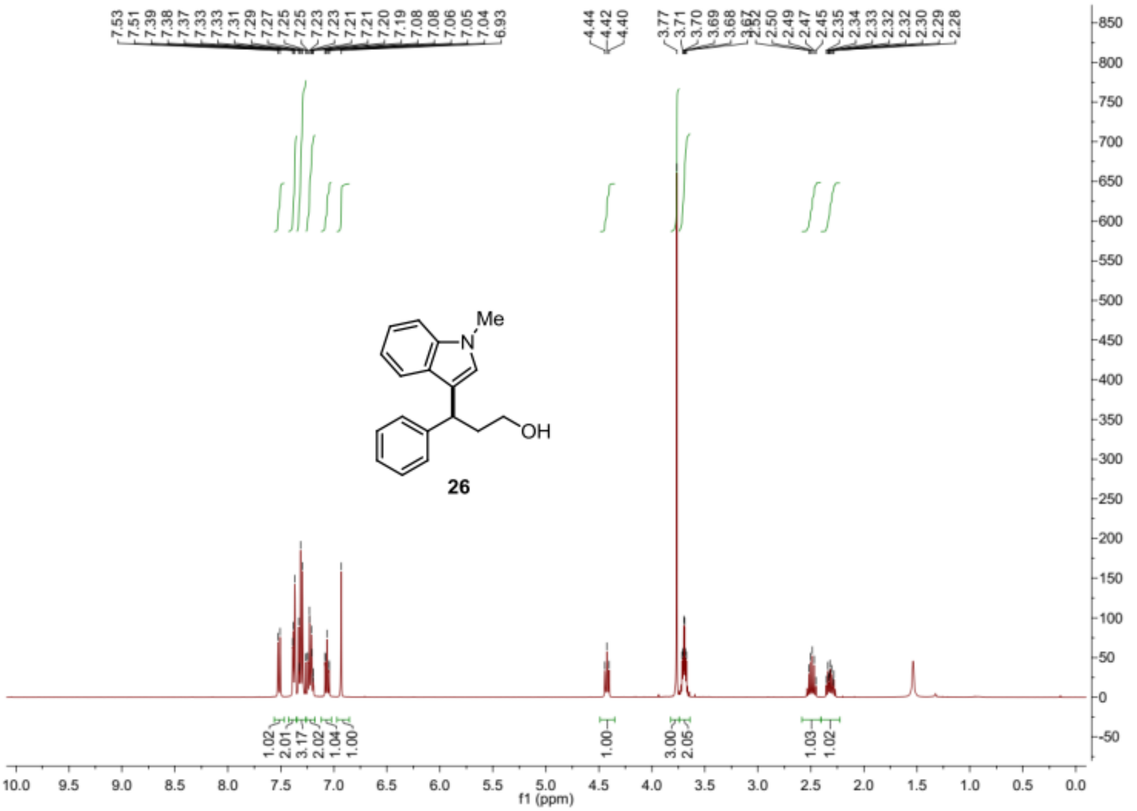

^{1}H NMR (400 MHz, $CDCl_3$, rt)

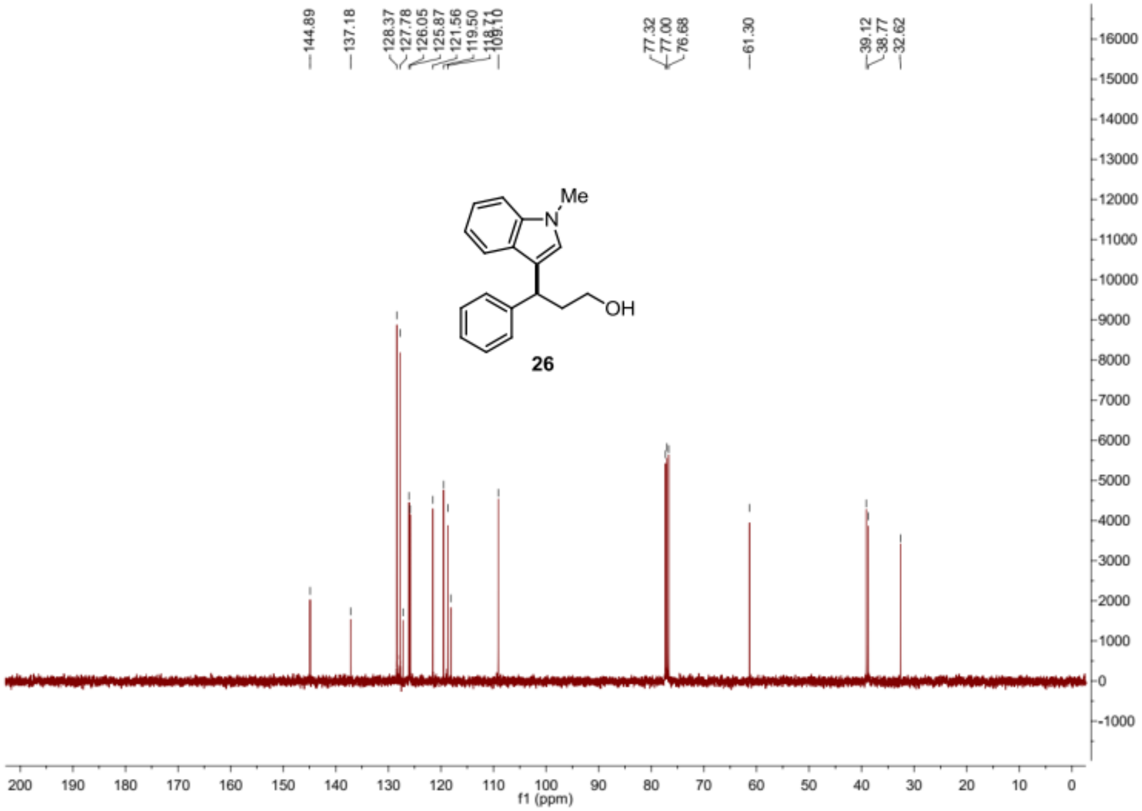

^{13}C NMR (101 MHz, $CDCl_3$, rt)

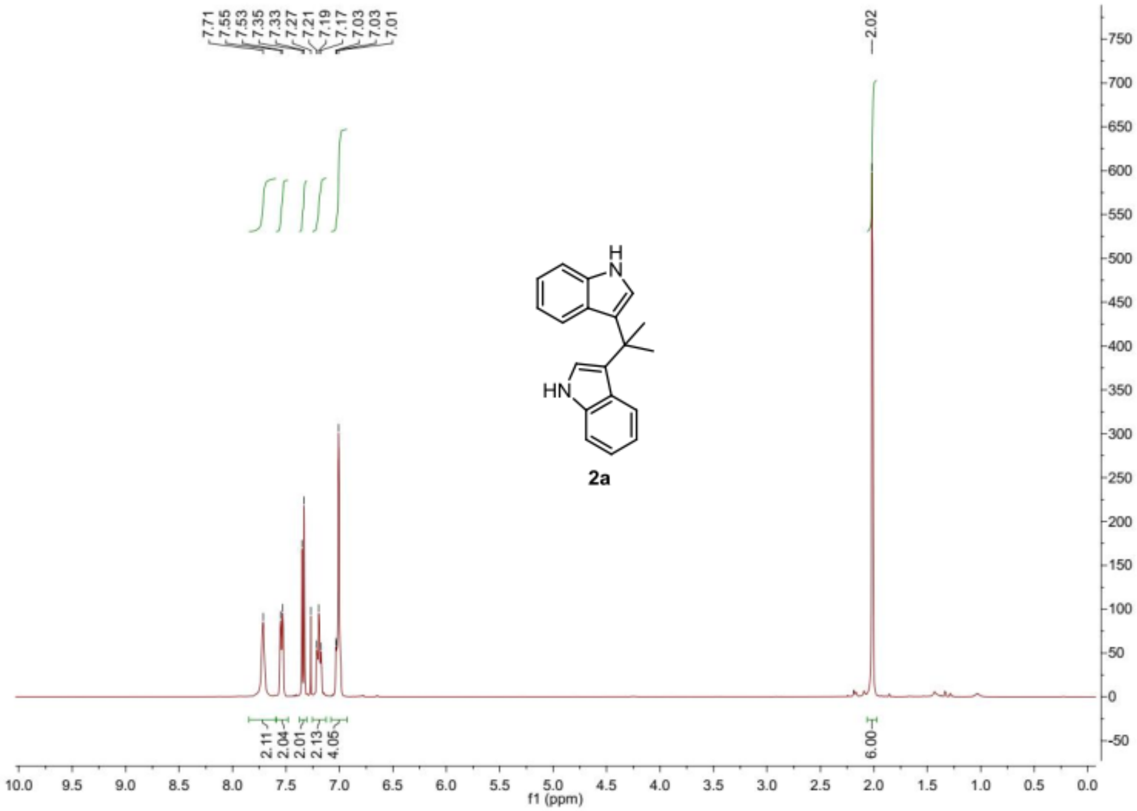

^{1}H NMR (400 MHz, $CDCl_3$, rt)

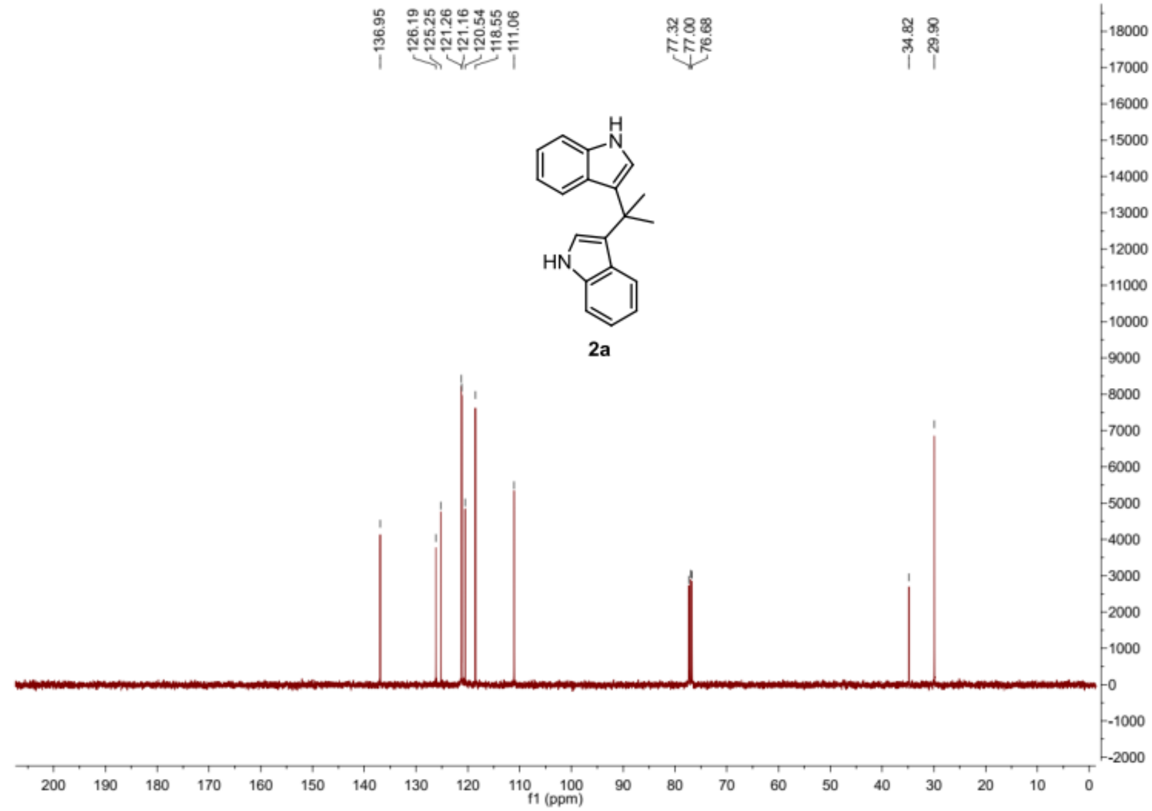

^{13}C NMR (101 MHz, $CDCl_3$, rt)

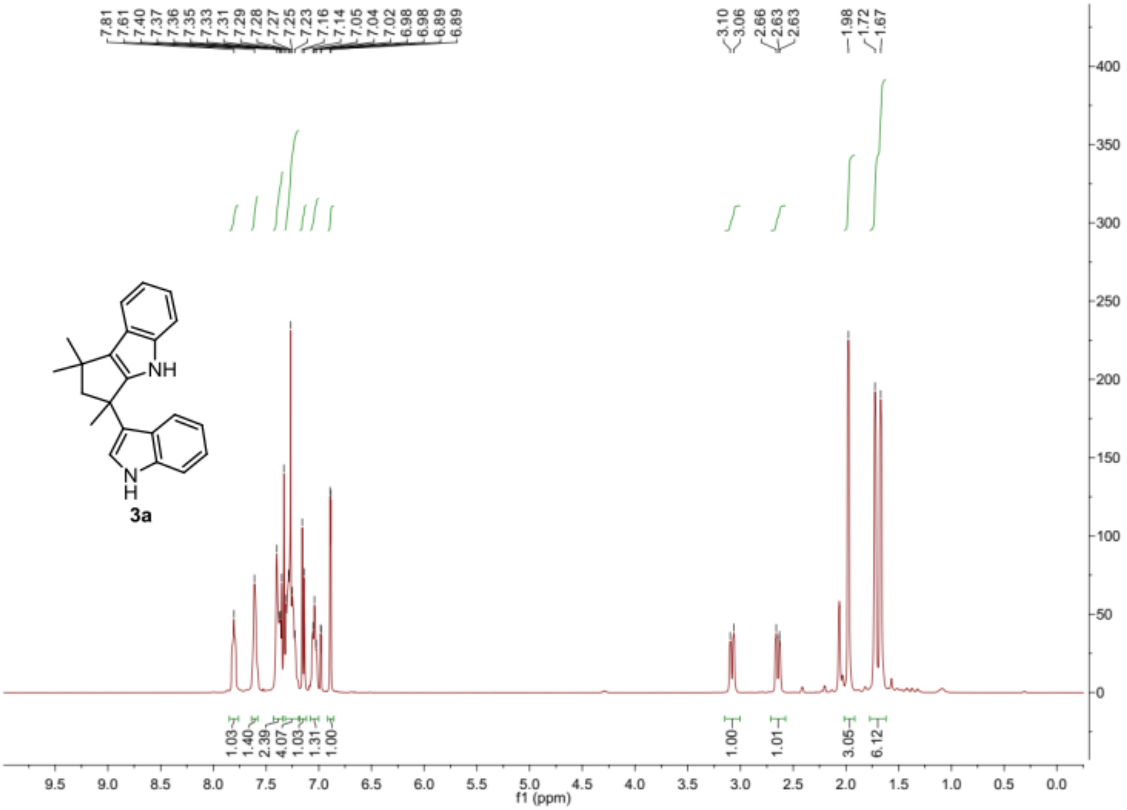

^1H NMR (400 MHz, $CDCl_3$, rt)

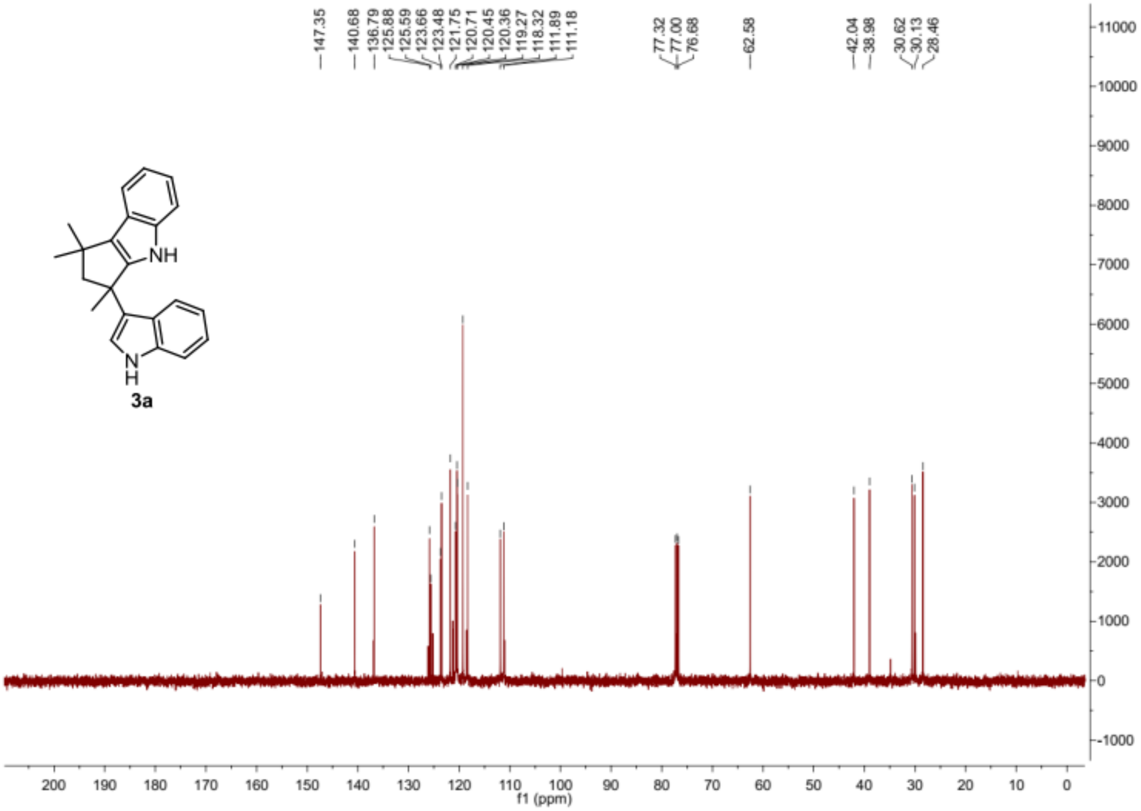

^{13}C NMR (101 MHz, $CDCl_3$, rt)

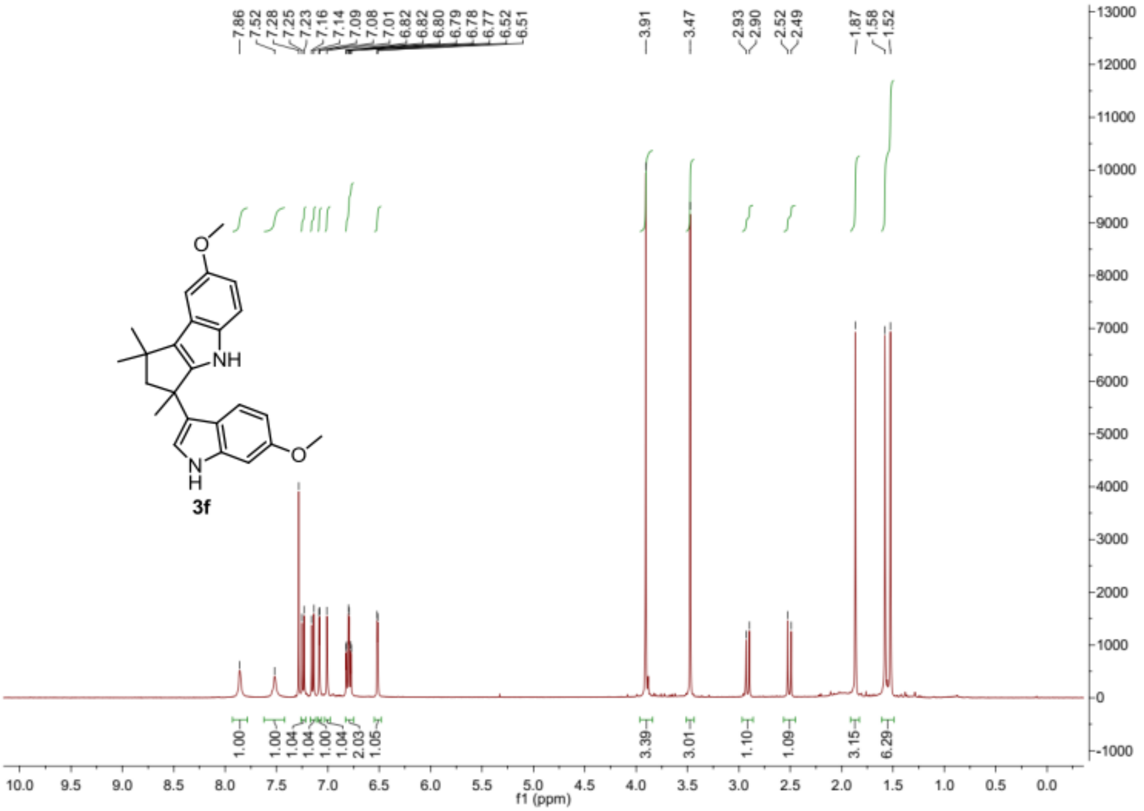

^1H NMR (400 MHz, $CDCl_3$, rt)

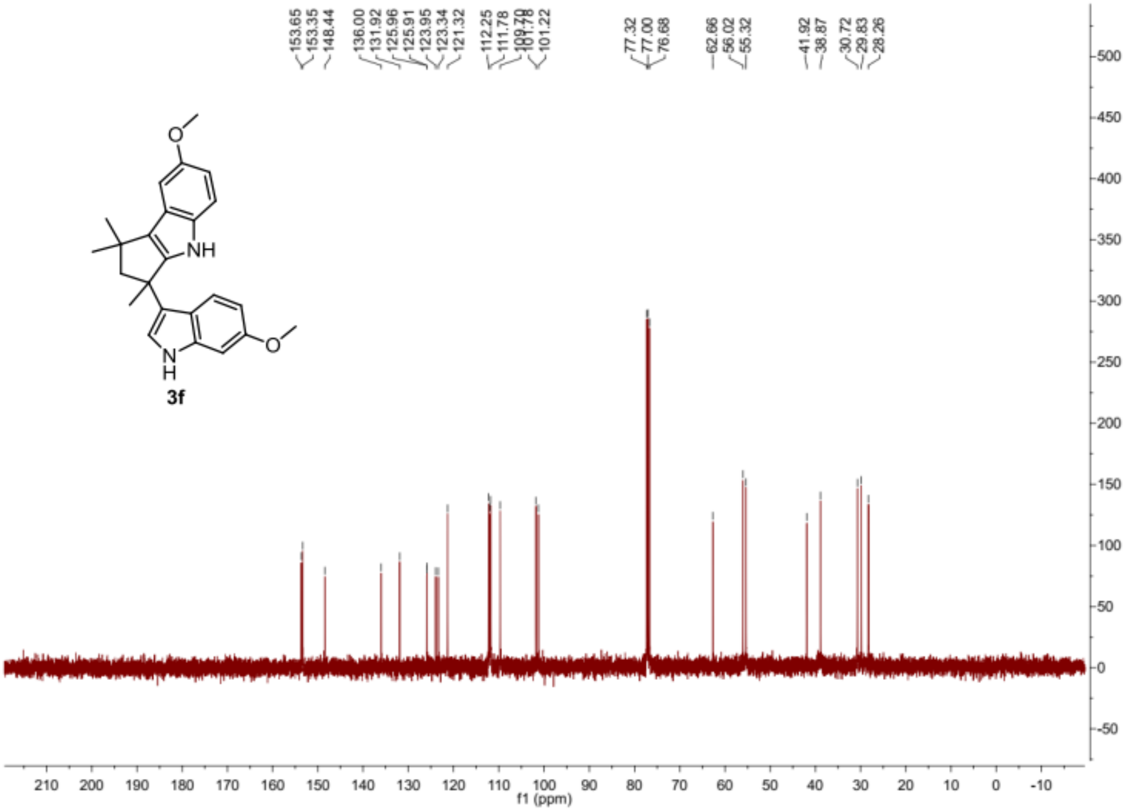

^{13}C NMR (101 MHz, $CDCl_3$, rt)

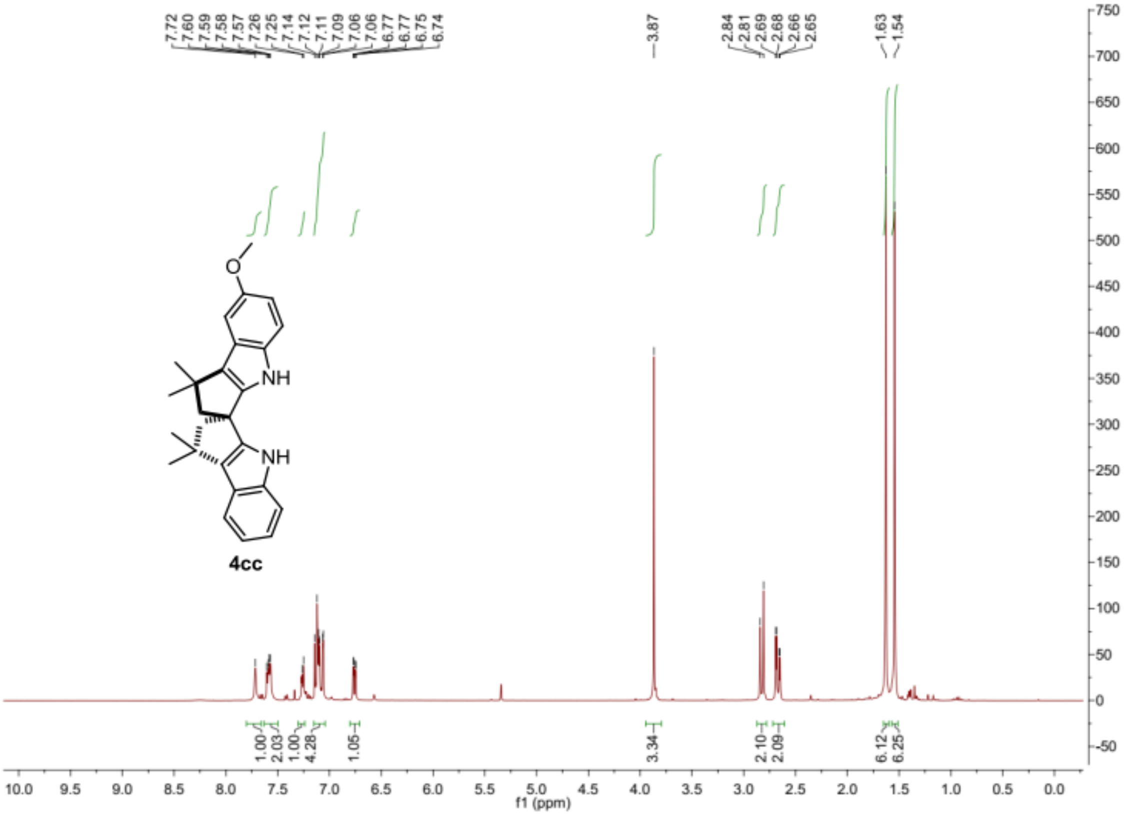

^{1}H NMR (400 MHz, CD_2Cl_2, rt)

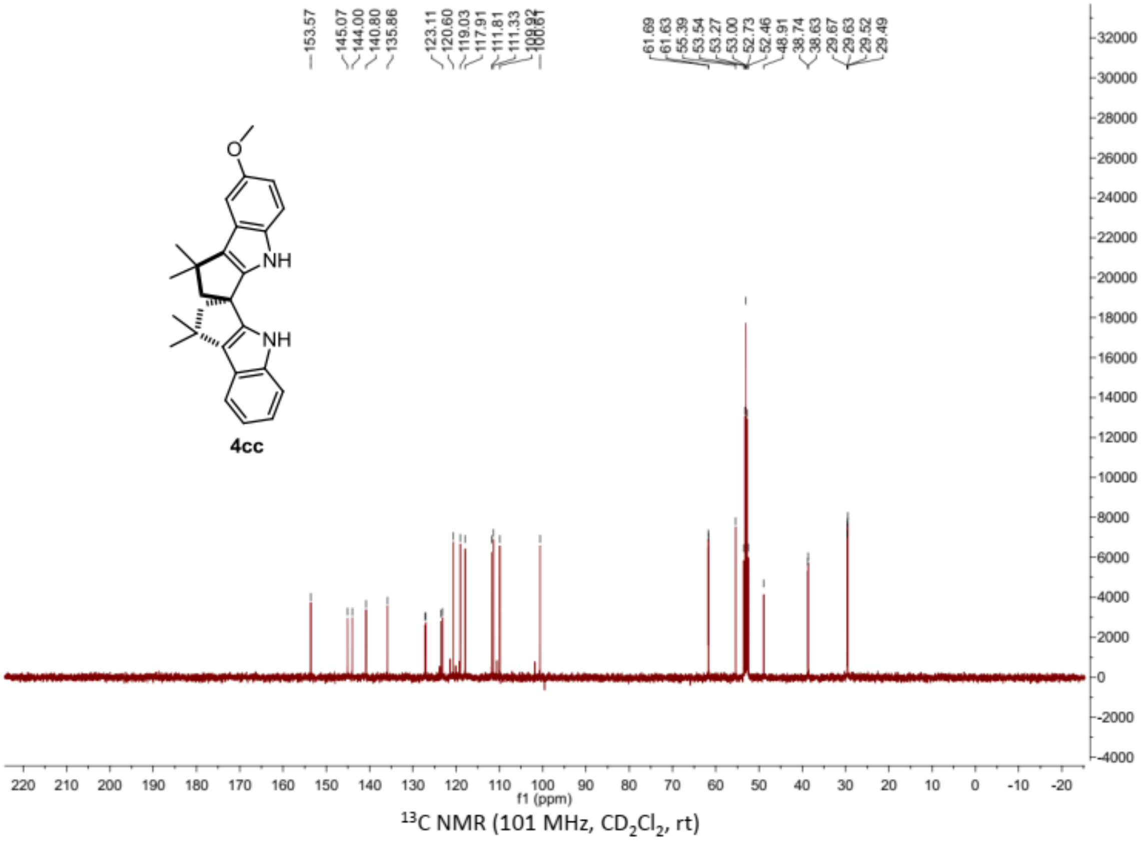

^{13}C NMR (101 MHz, CD_2Cl_2, rt)

12 SFC chromatograms

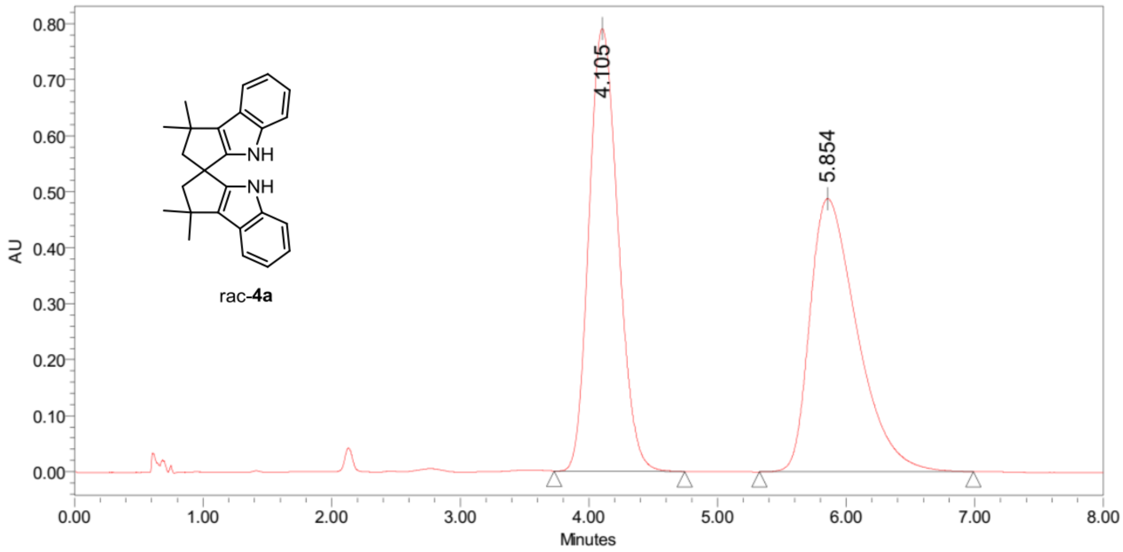

Peak Results

	Retention Time (min)	Area	% Area	Height	Int Type
1	4.105	12449100	50.02	790346	bb
2	5.854	12437047	49.98	488168	bb

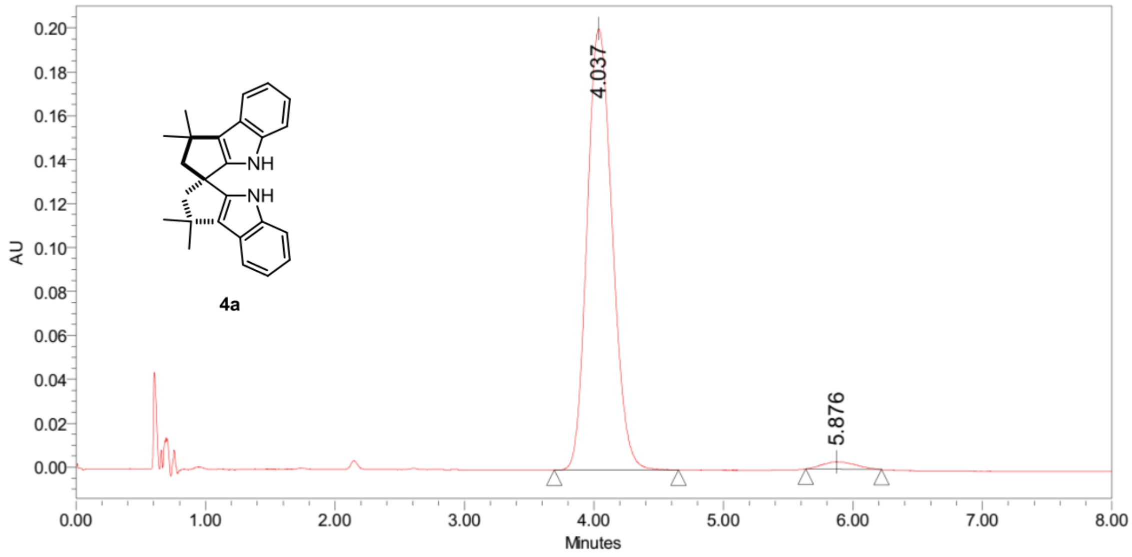

Peak Results

	Retention Time (min)	Area	% Area	Height	Int Type
1	4.037	2715763	97.85	200906	bb
2	5.876	59663	2.15	3333	bb

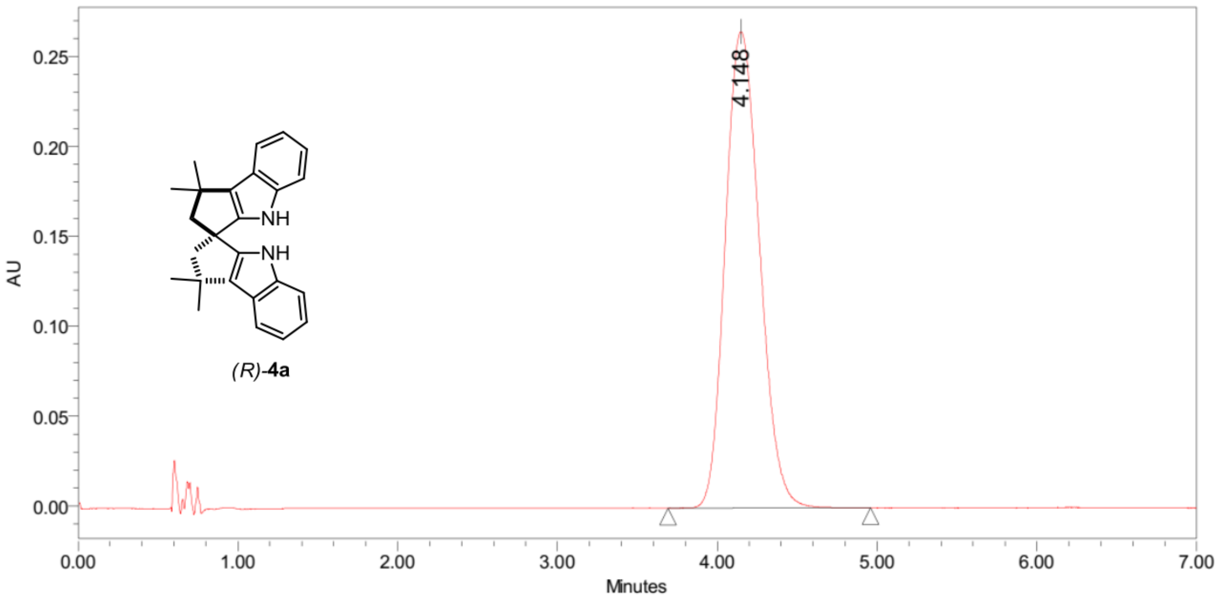

Peak Results

	Retention Time (min)	Area	% Area	Height	Int Type
1	4.148	3969020	100.00	265042	bb

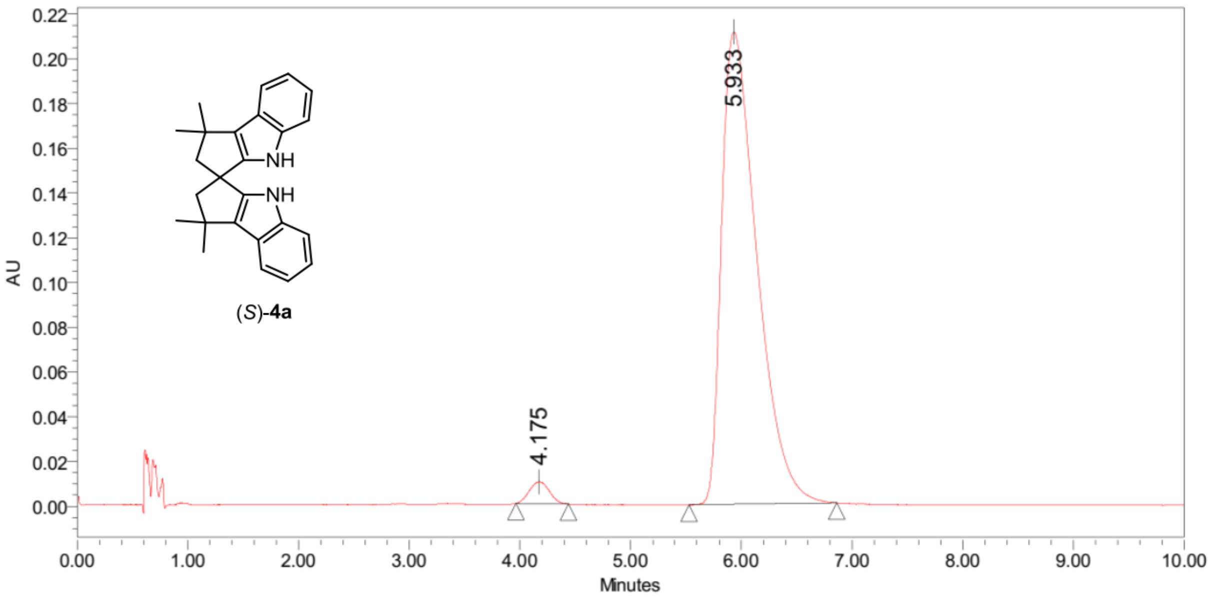

Peak Results

	Retention Time (min)	Area	% Area	Height	Int Type
1	4.175	124257	2.58	9809	bb
2	5.933	4687925	97.42	211021	bb

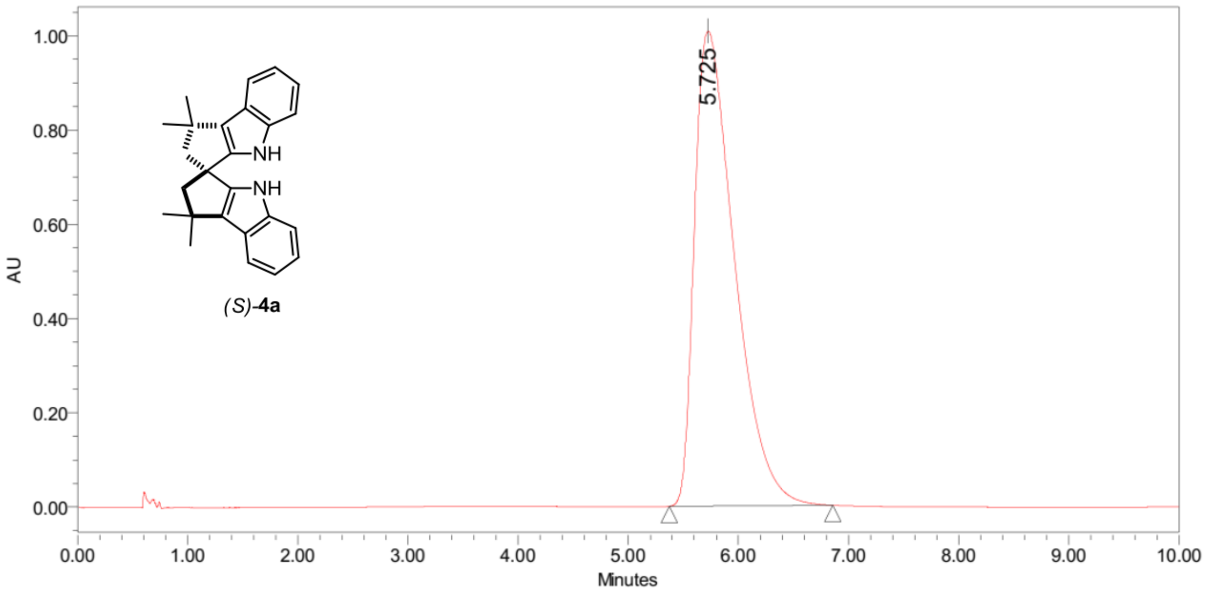

Peak Results

	Retention Time (min)	Area	% Area	Height	Int Type
1	5.725	25490161	100.00	1008039	bb

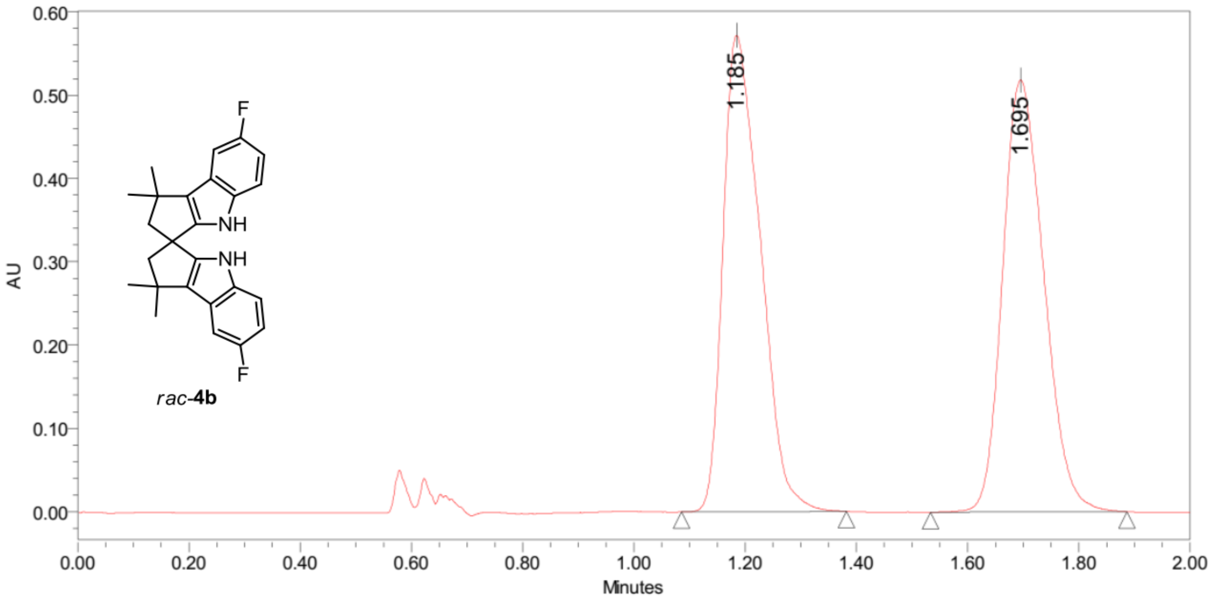

Peak Results

	Retention Time (min)	Area	% Area	Height	Int Type
1	1.185	2653168	49.92	571447	bb
2	1.695	2661622	50.08	518036	bb

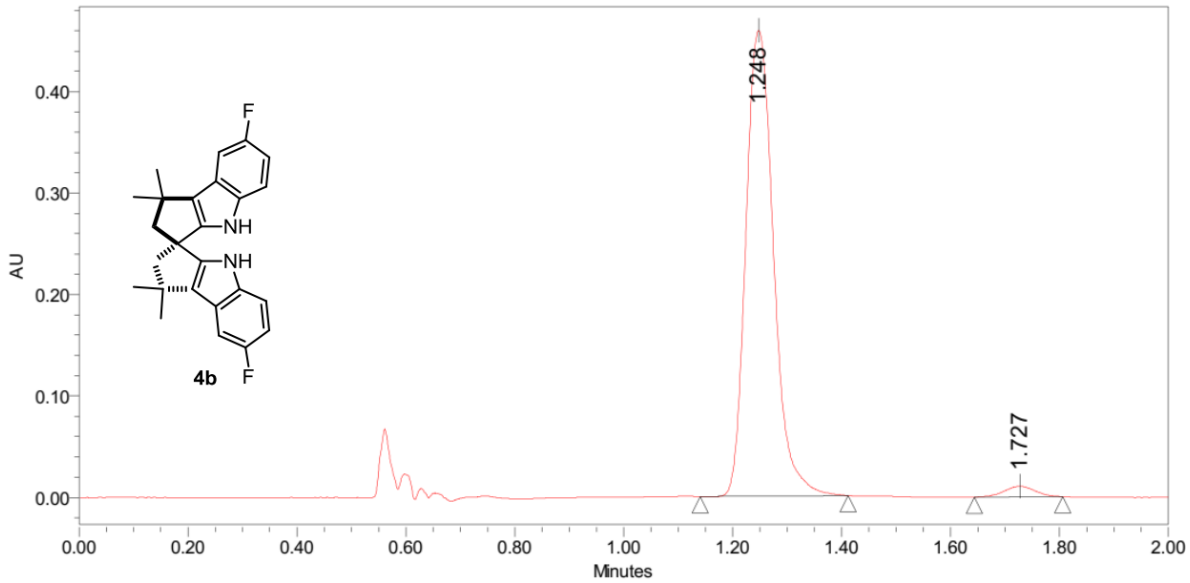

Peak Results

	Retention Time (min)	Area	% Area	Height	Int Type
1	1.248	1620013	97.51	459002	bb
2	1.727	41361	2.49	10427	bb

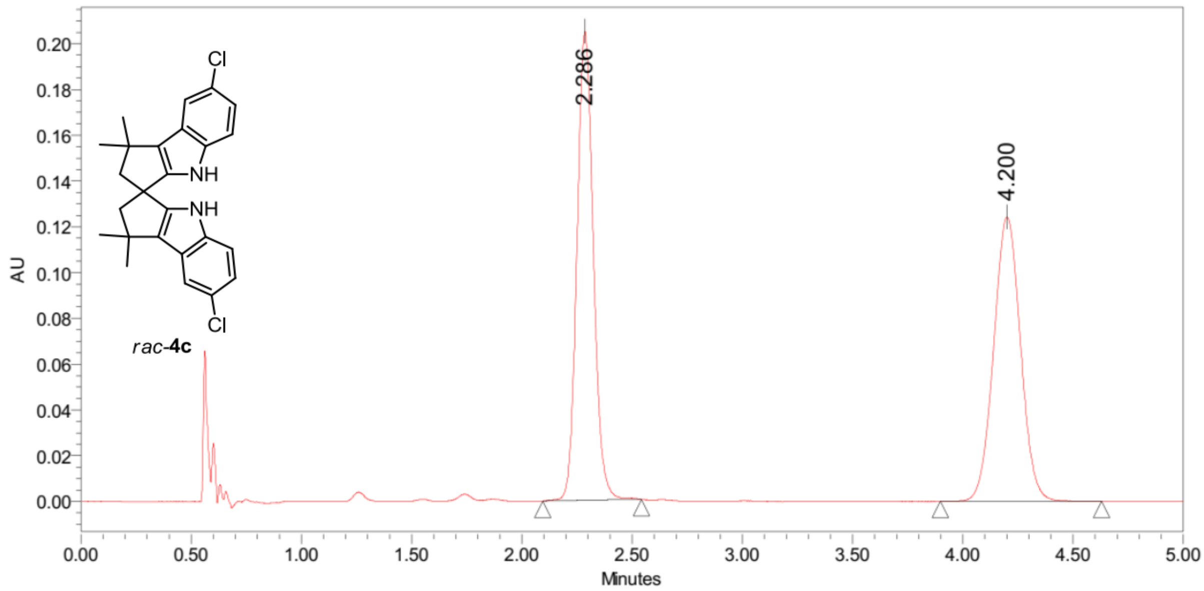

Peak Results

	Retention Time (min)	Area	% Area	Height	Int Type
1	2.286	1053894	49.99	204860	bb
2	4.200	1054274	50.01	124144	bb

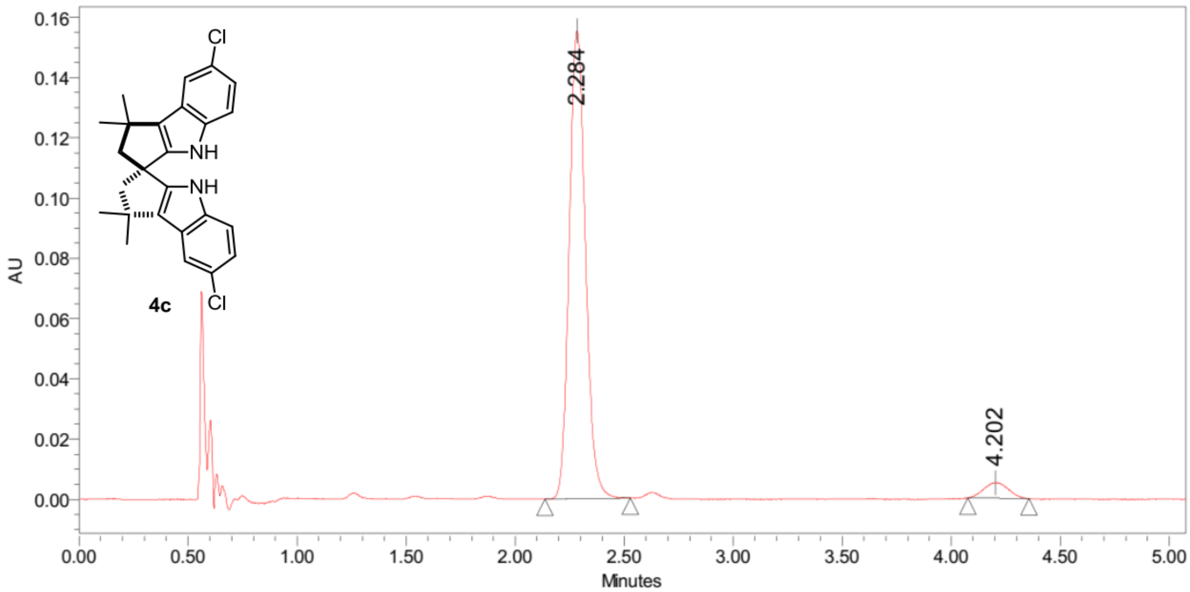

Peak Results

	Retention Time (min)	Area	% Area	Height	Int Type
1	2.284	797301	95.15	155207	bb
2	4.202	40633	4.85	5183	bb

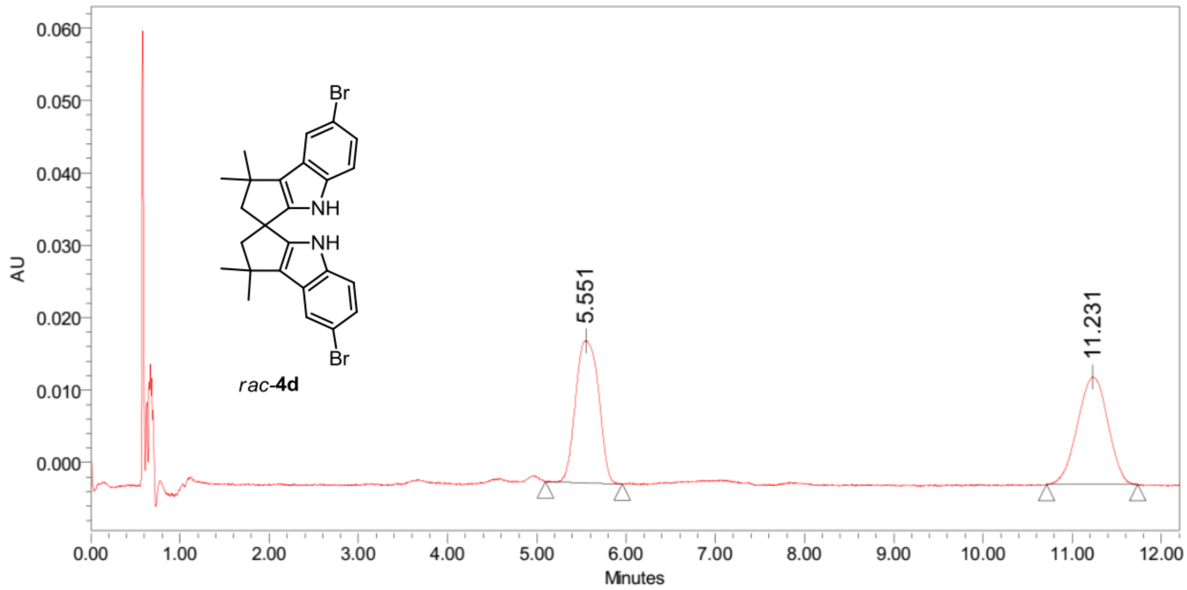

Peak Results

	Retention Time (min)	Area	% Area	Height	Int Type
1	5.551	352627	50.17	19654	bb
2	11.231	350171	49.83	14834	bb

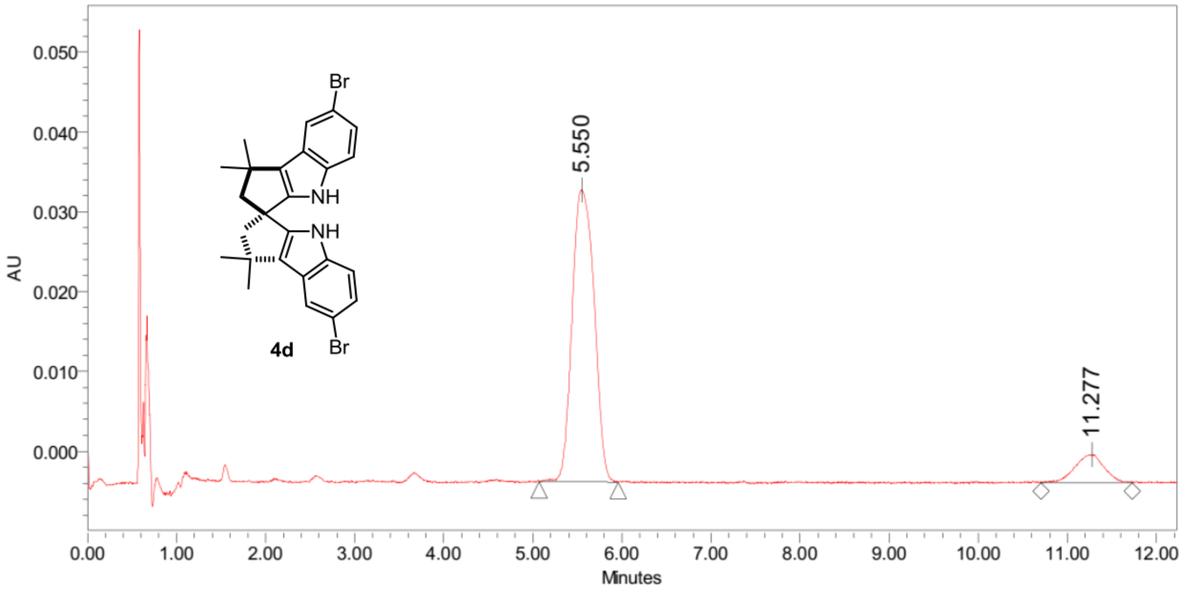

Peak Results

	Retention Time (min)	Area	% Area	Height	Int Type
1	5.550	627525	88.65	36575	BB
2	11.277	80356	11.35	3531	vv

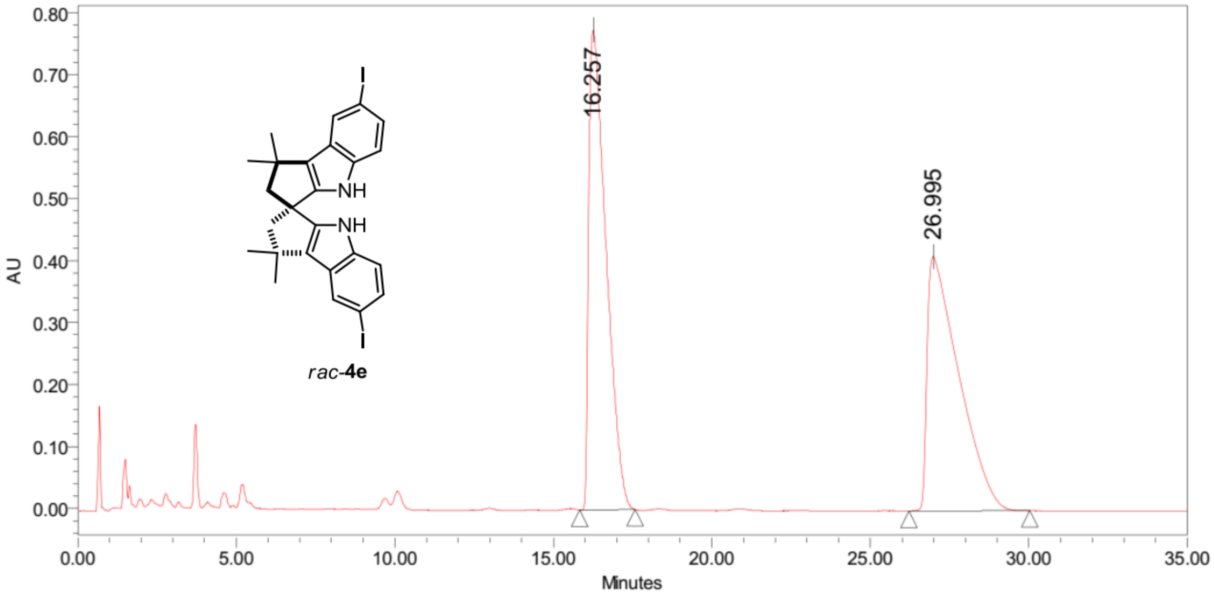

Peak Results

	Retention Time (min)	Area	% Area	Height	Int Type
1	16.257	29268603	50.67	774508	bb
2	26.995	28491204	49.33	410334	bb

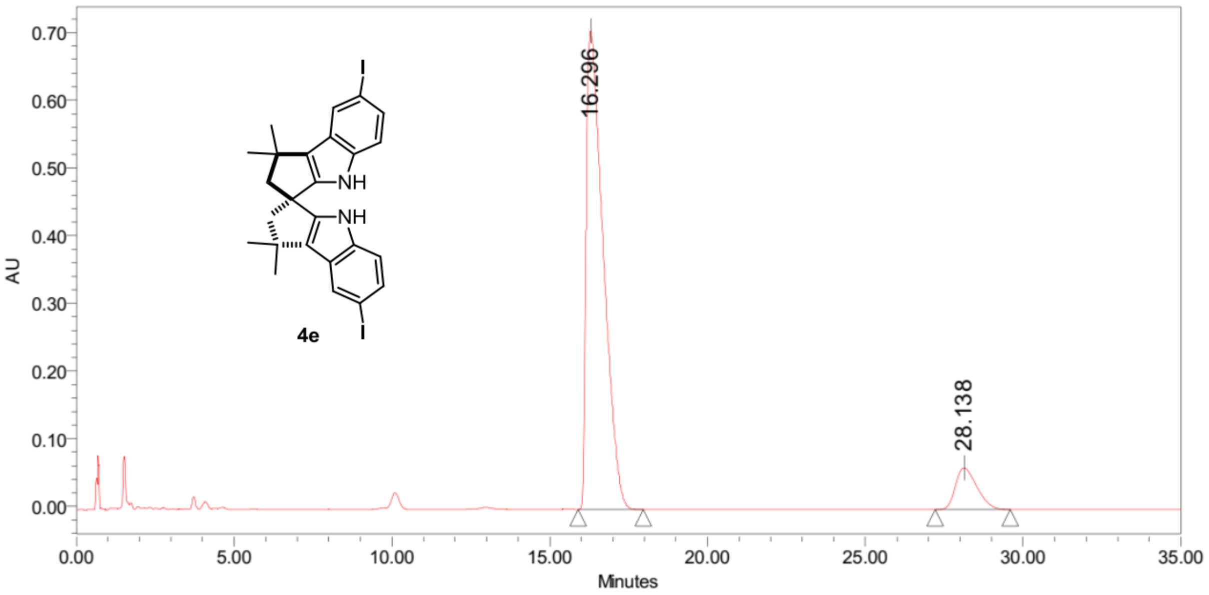

Peak Results

	Retention Time (min)	Area	% Area	Height	Int Type
1	16.296	26268785	90.01	706662	Bb
2	28.138	2916042	9.99	61577	bb

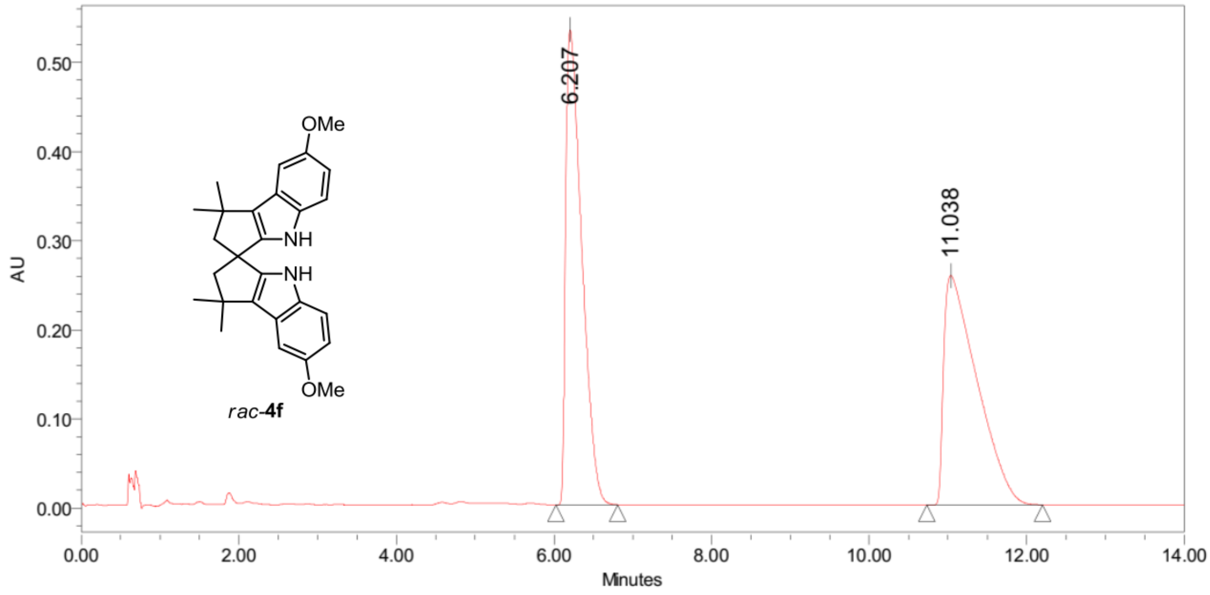

Peak Results

	Retention Time (min)	Area	% Area	Height	Int Type
1	6.207	7551949	50.05	533012	bb
2	11.038	7535908	49.95	257366	bb

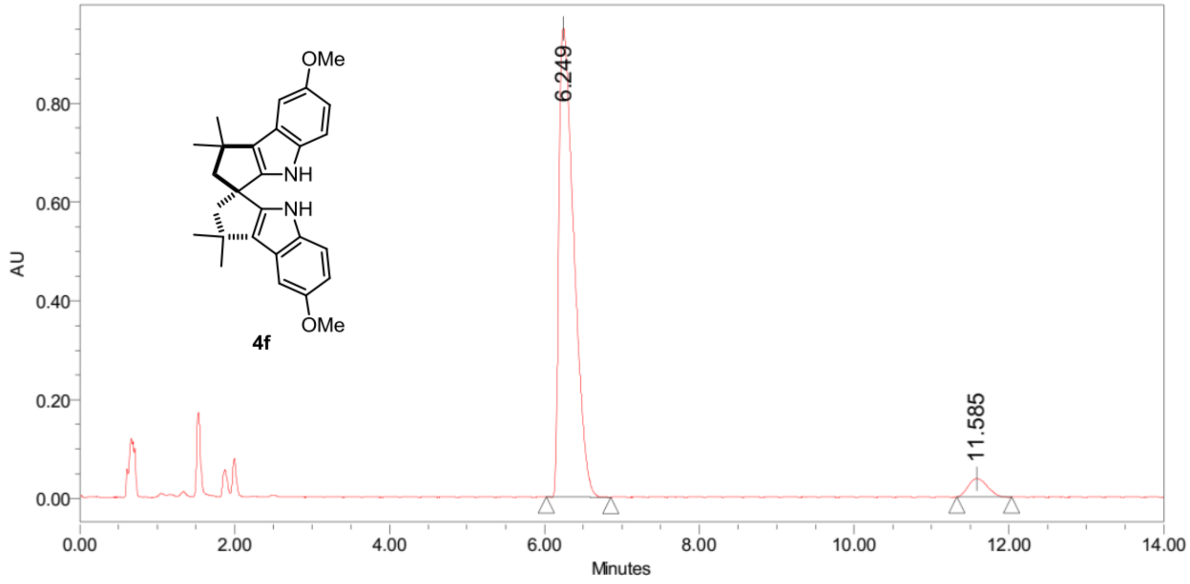

Peak Results

	Retention Time (min)	Area	% Area	Height	Int Type
1	6.249	12696162	95.09	948606	bb
2	11.585	655262	4.91	37731	bb

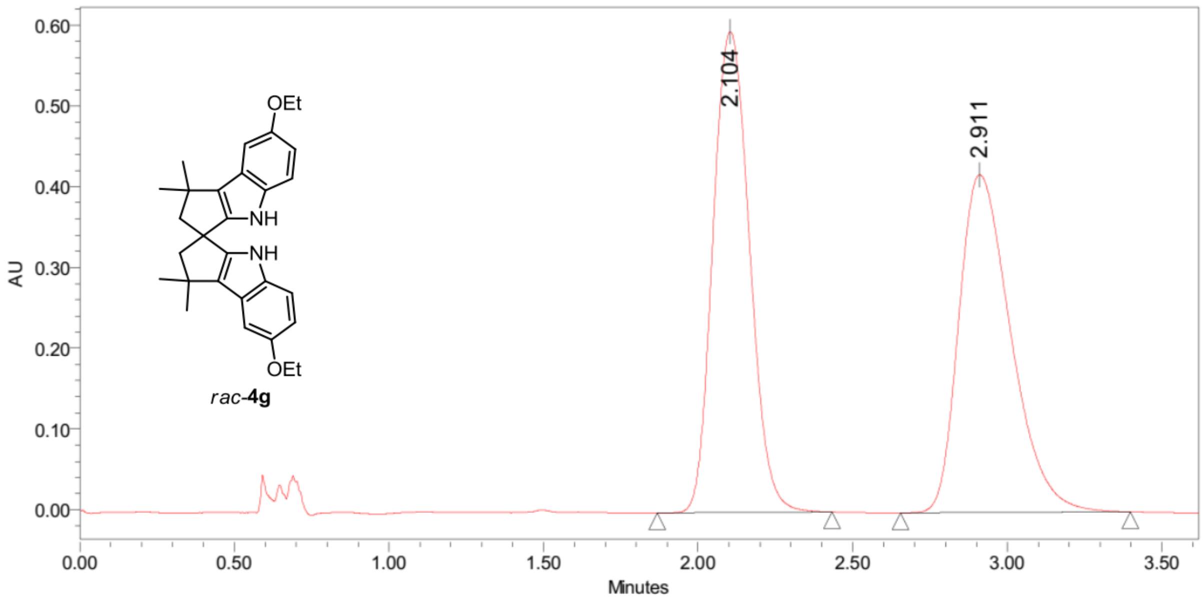

Peak Results

	Retention Time (min)	Area	% Area	Height	Int Type
1	2.104	4902988	50.11	594965	bb
2	2.911	4881758	49.89	417936	bb

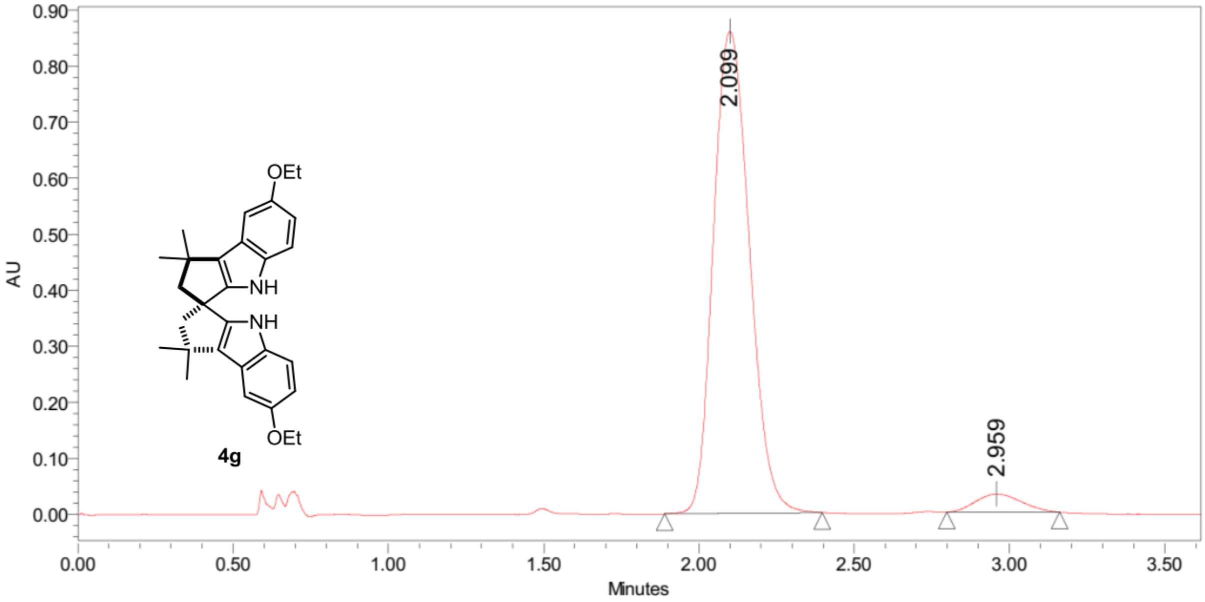

Peak Results

	Retention Time (min)	Area	% Area	Height	Int Type
1	2.099	6944781	95.46	860172	bb
2	2.959	329956	4.54	32209	bb

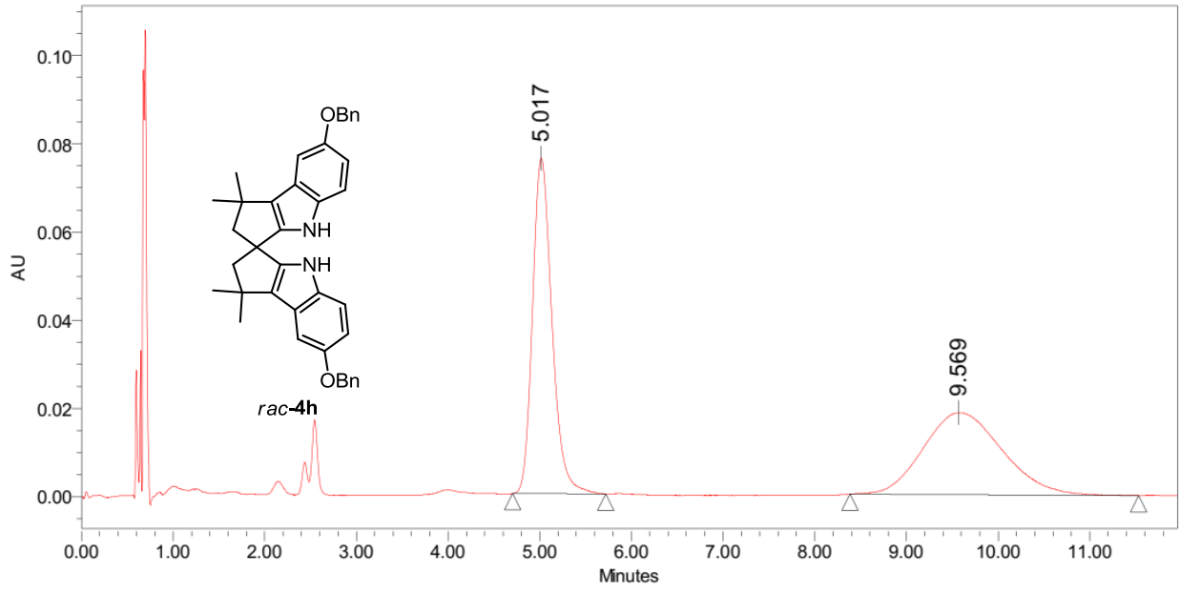

Peak Results

	Retention Time (min)	Area	% Area	Height	Int Type
1	5.017	1122211	50.21	76159	bb
2	9.569	1112706	49.79	18618	bb

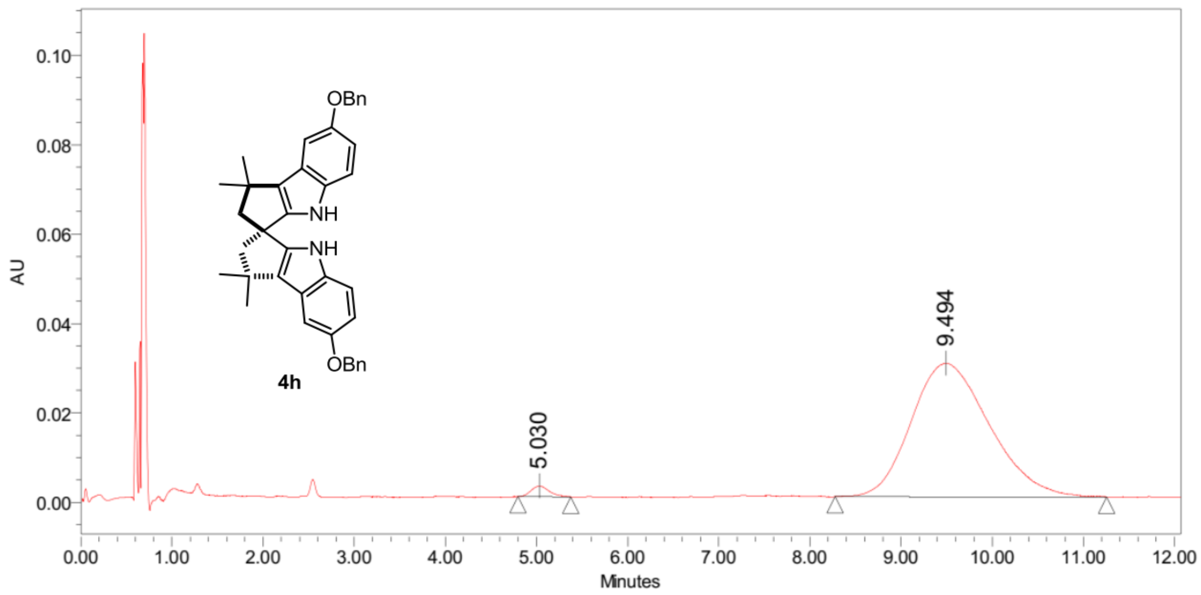

Peak Results

	Retention Time (min)	Area	% Area	Height	Int Type
1	5.030	33164	1.83	2404	bb
2	9.494	1779328	98.17	29894	bb

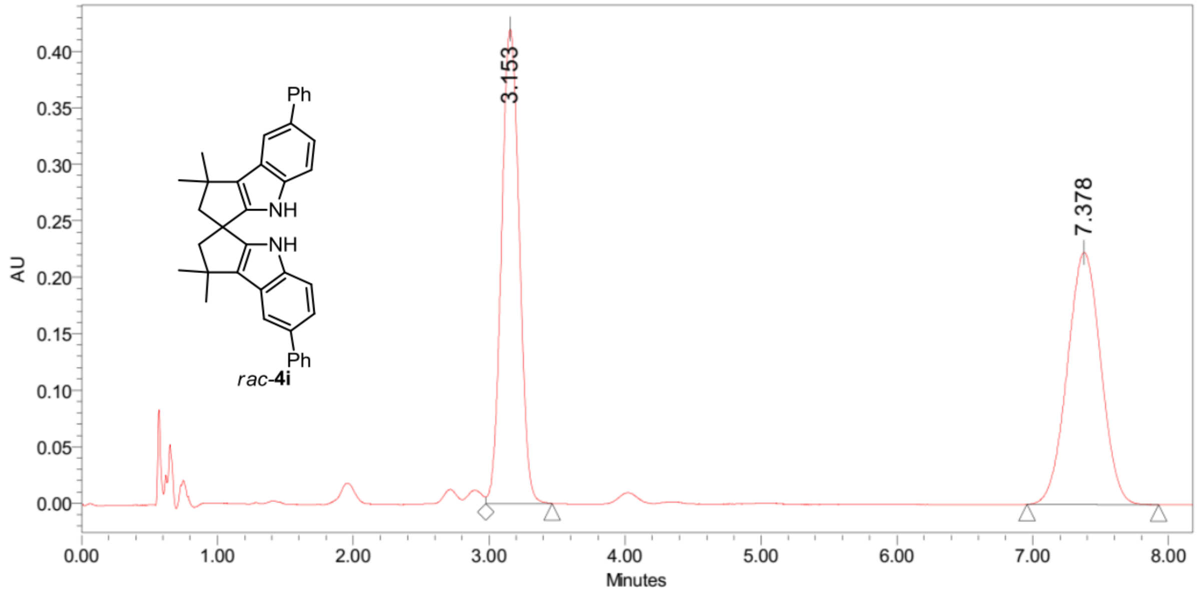

Peak Results

	Retention Time (min)	Area	% Area	Height	Int Type
1	3.153	3816170	50.01	419821	Vb
2	7.378	3814705	49.99	223074	bb

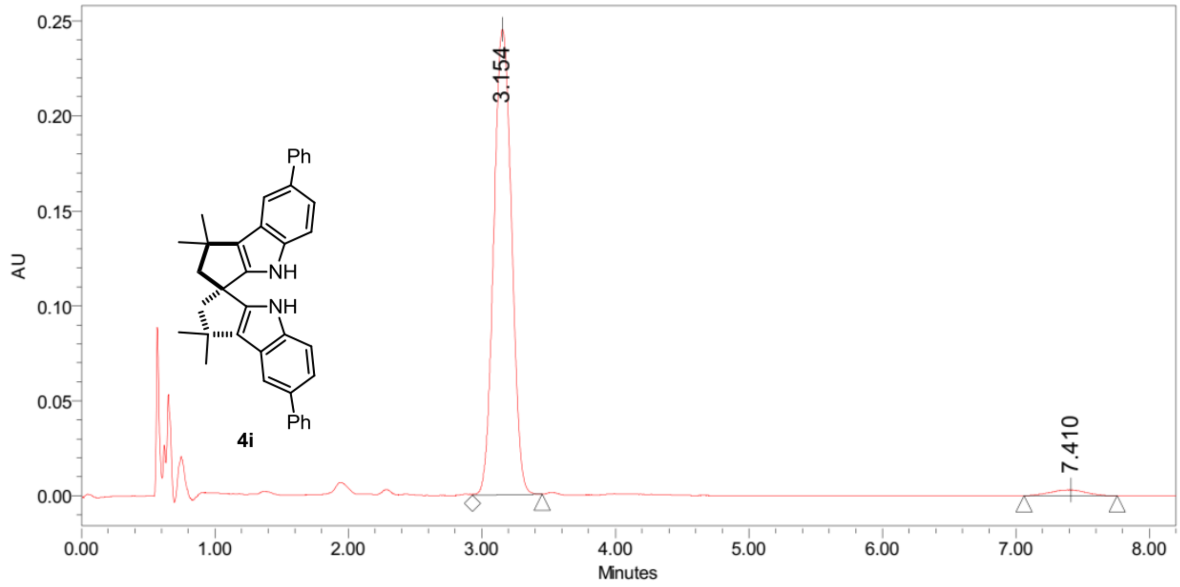

Peak Results

	Retention Time (min)	Area	% Area	Height	Int Type
1	3.154	2261189	97.64	244930	VB
2	7.410	54557	2.36	2982	bb

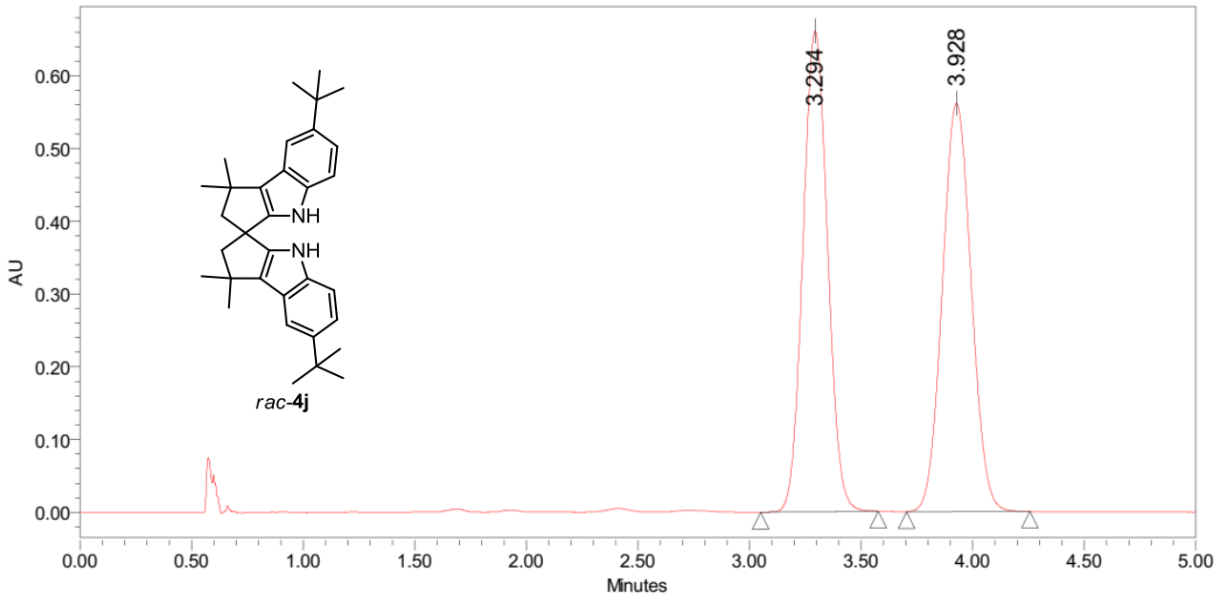

Peak Results

	Retention Time (min)	Area	% Area	Height	Int Type
1	3.294	5064160	50.05	660371	bb
2	3.928	5054365	49.95	561014	bb

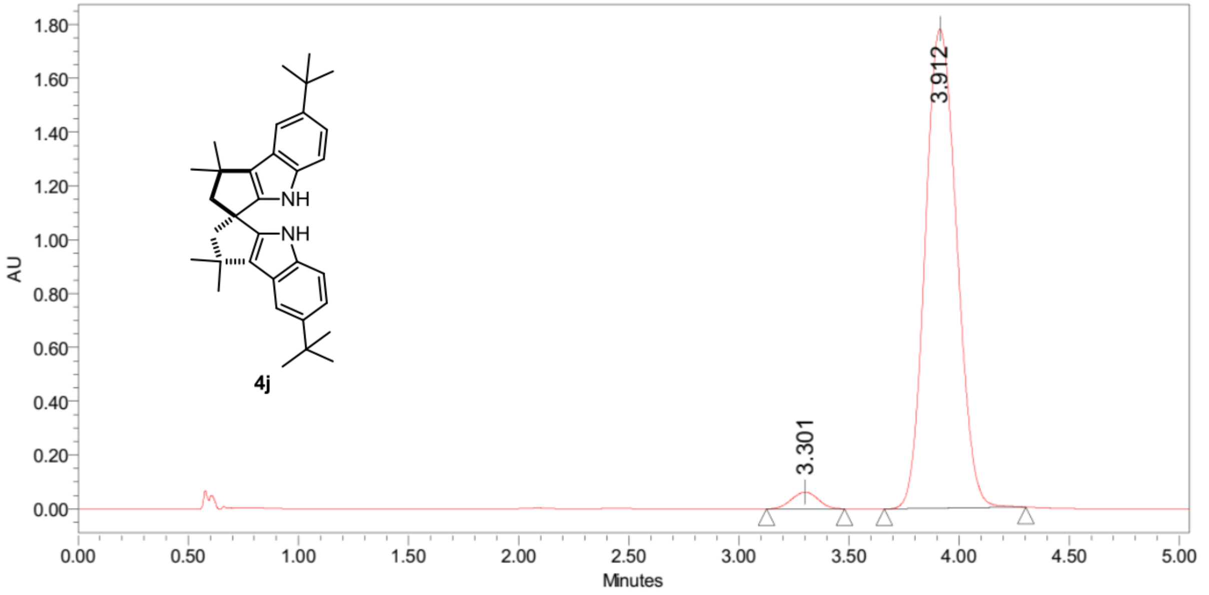

Peak Results

	Retention Time (min)	Area	% Area	Height	Int Type
1	3.301	515838	2.83	61668	bb
2	3.912	17743247	97.17	1781566	bb

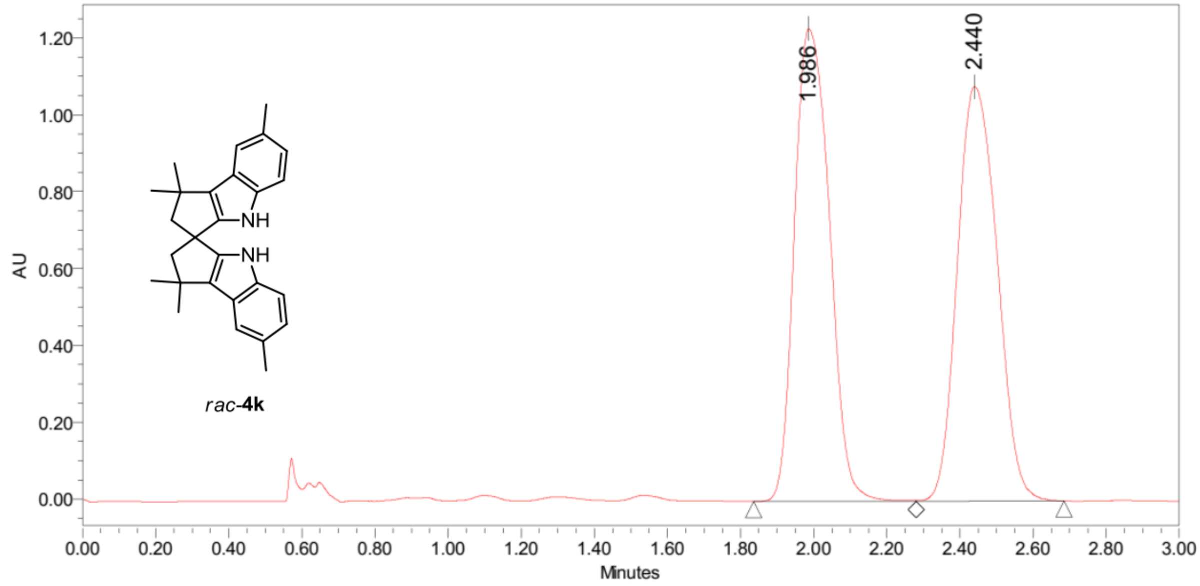

rac-**4k**

Peak Results

	Retention Time (min)	Area	% Area	Height	Int Type
1	1.986	8198338	50.09	1229821	bV
2	2.440	8169328	49.91	1077954	Vb

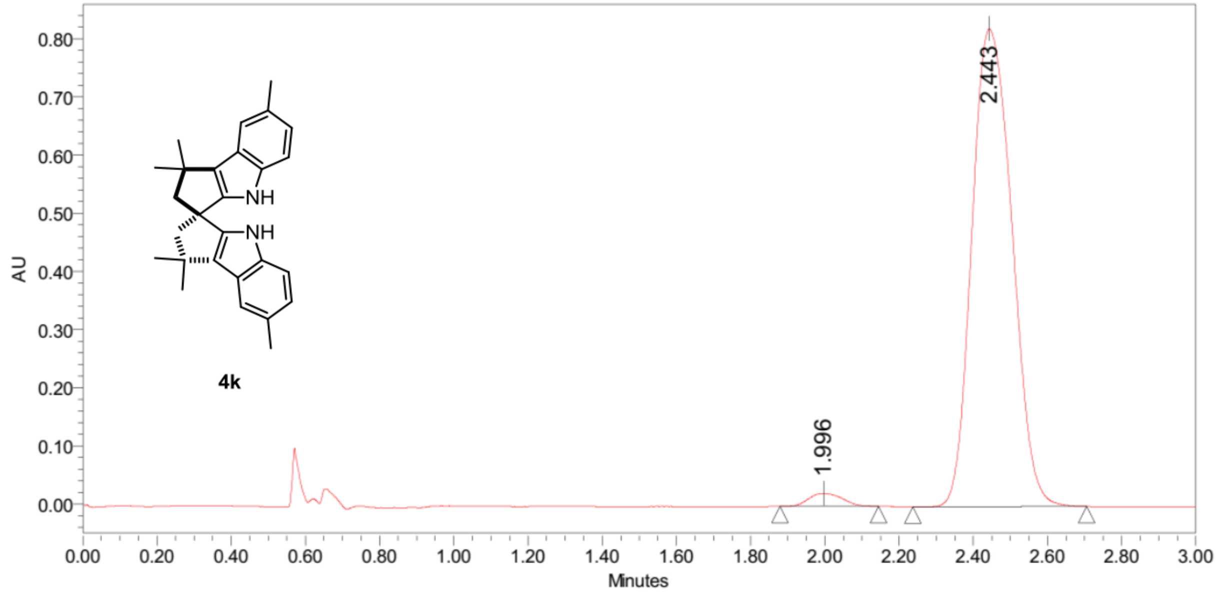

4k

Peak Results

	Retention Time (min)	Area	% Area	Height	Int Type
1	1.996	139903	2.22	22200	bb
2	2.443	6162822	97.78	821474	bb

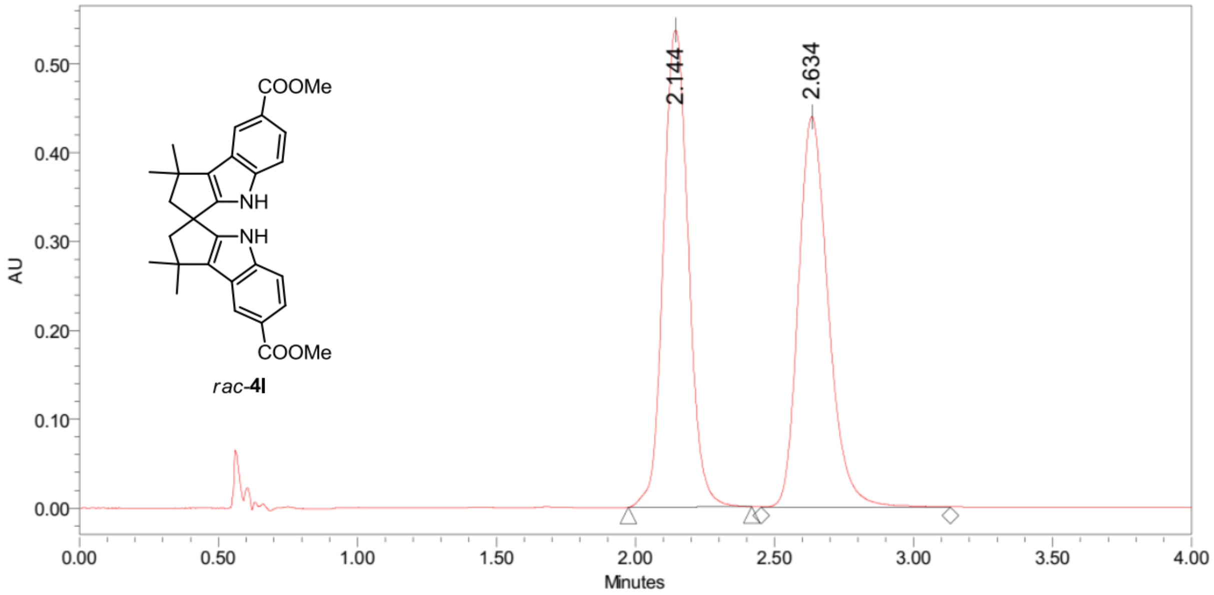

Peak Results

	Retention Time (min)	Area	% Area	Height	Int Type
1	2.144	3350439	50.40	536913	bb
2	2.634	3297497	49.60	439776	VV

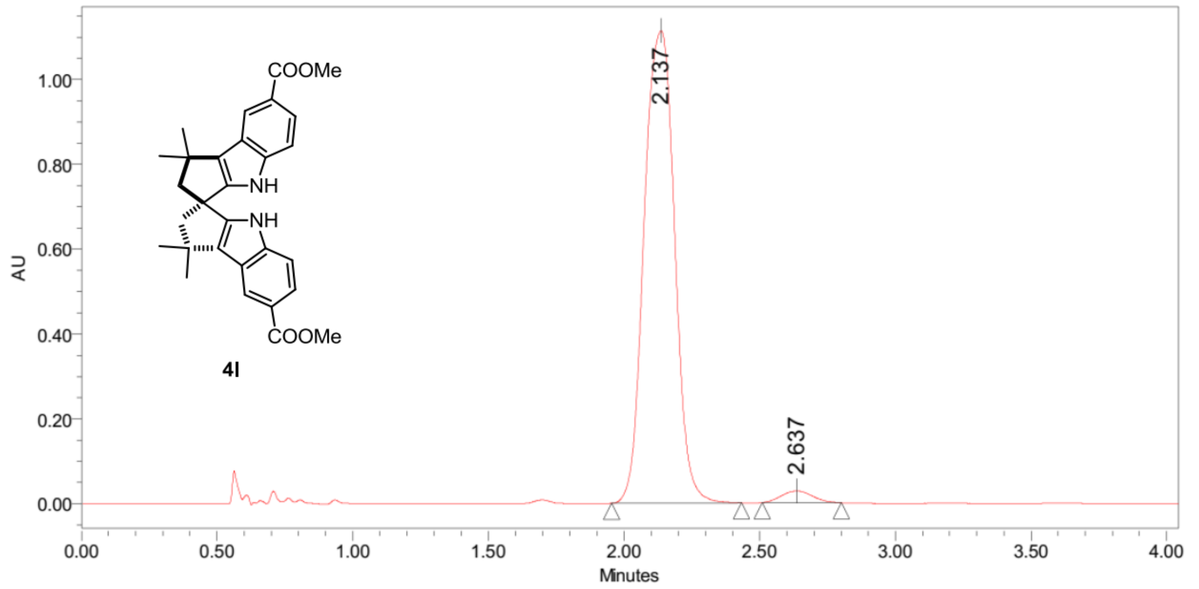

Peak Results

	Retention Time (min)	Area	% Area	Height	Int Type
1	2.137	8673177	97.54	1113475	bb
2	2.637	218356	2.46	27465	bb

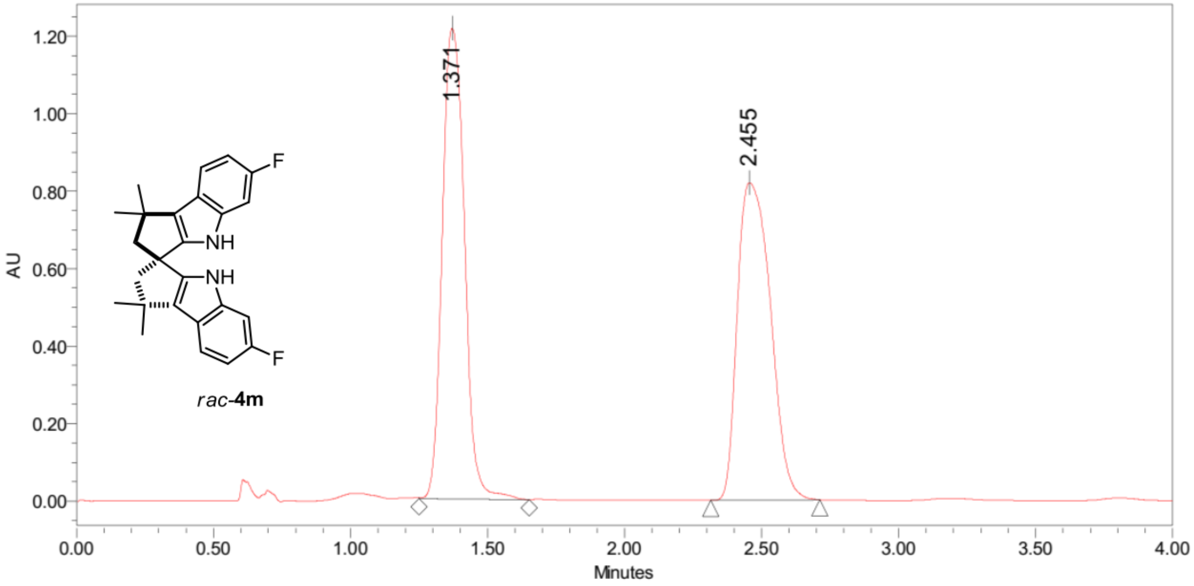

Peak Results

	Retention Time (min)	Area	% Area	Height	Int Type
1	1.371	6831717	49.87	1214793	VV
2	2.455	6866808	50.13	819430	bb

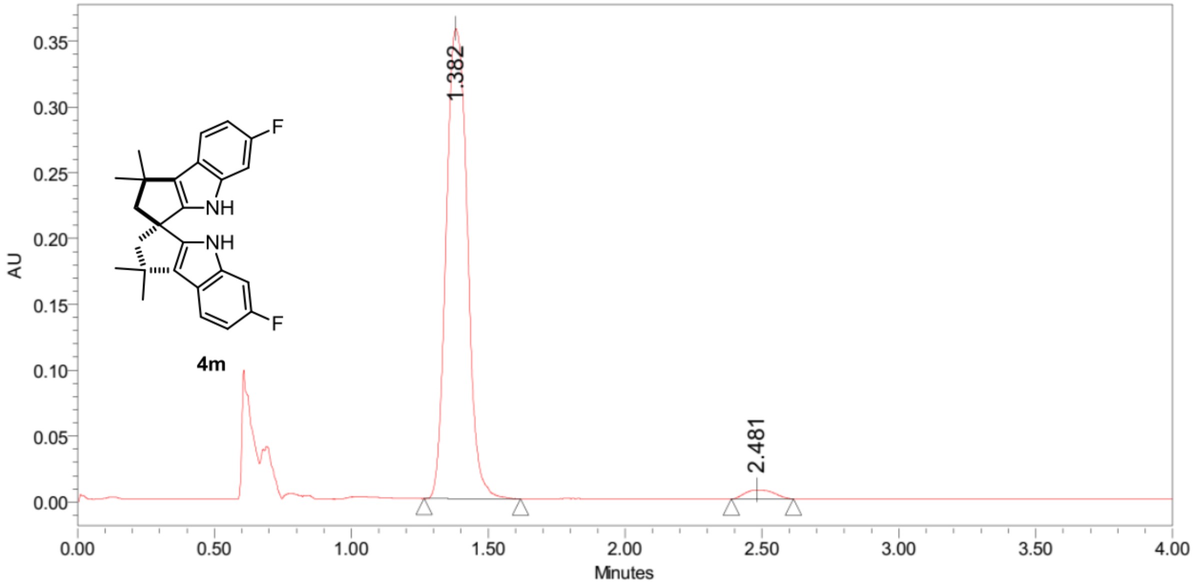

Peak Results

	Retention Time (min)	Area	% Area	Height	Int Type
1	1.382	1912827	97.50	356980	bb
2	2.481	48963	2.50	6708	bb

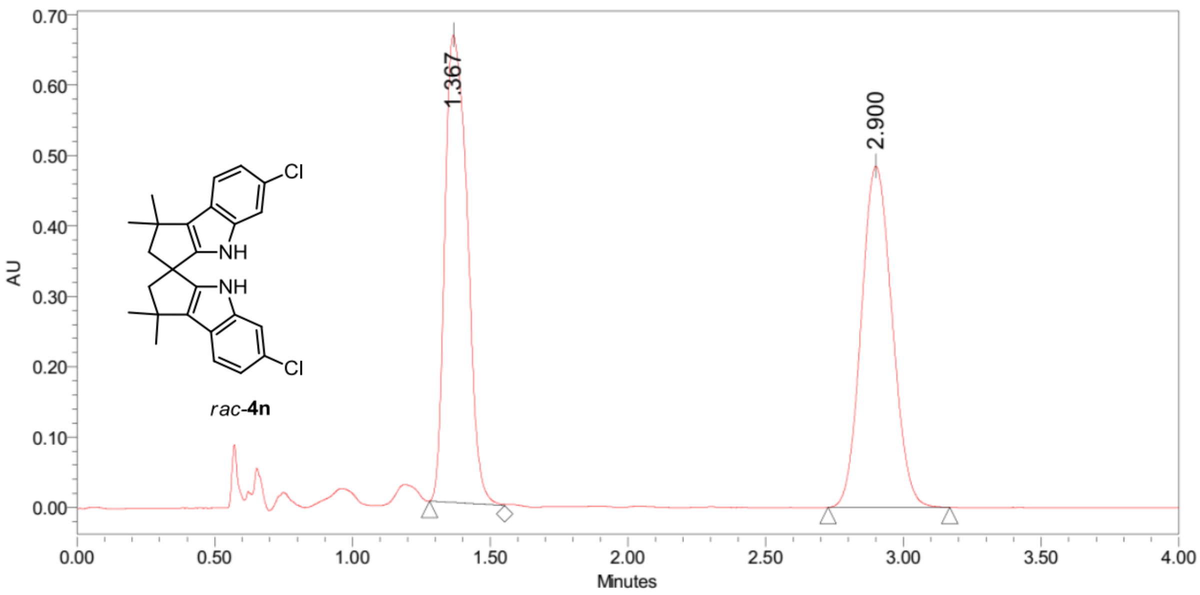

Peak Results

	Retention Time (min)	Area	% Area	Height	Int Type
1	1.367	3748892	49.90	664279	bV
2	2.900	3763373	50.10	485220	bb

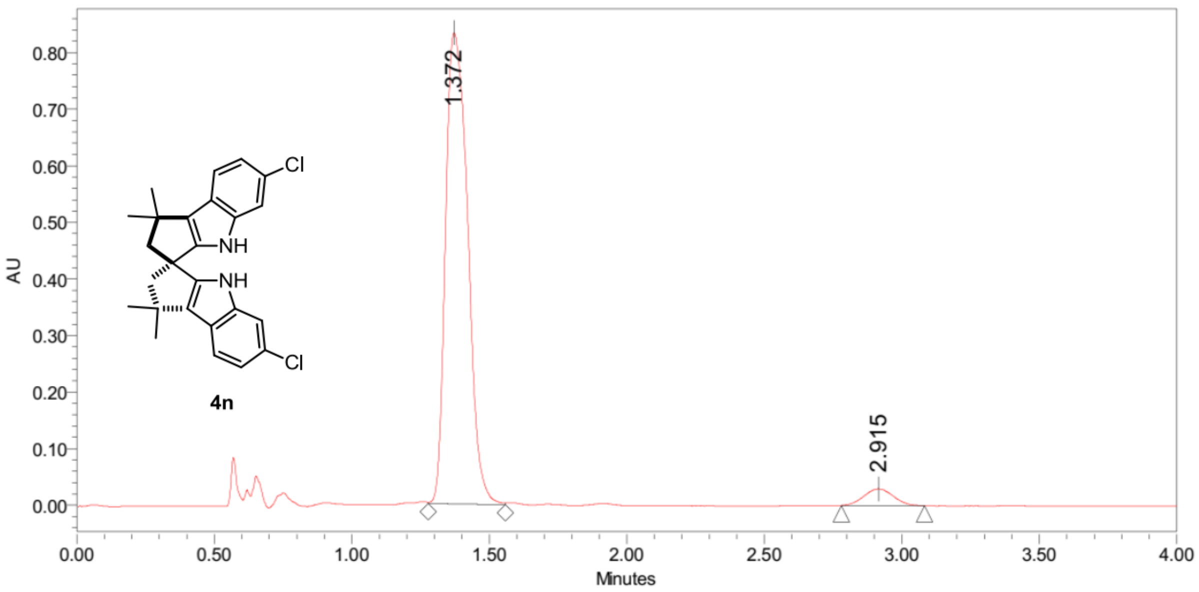

Peak Results

	Retention Time (min)	Area	% Area	Height	Int Type
1	1.372	4652876	95.51	832625	VV
2	2.915	218613	4.49	29440	bb

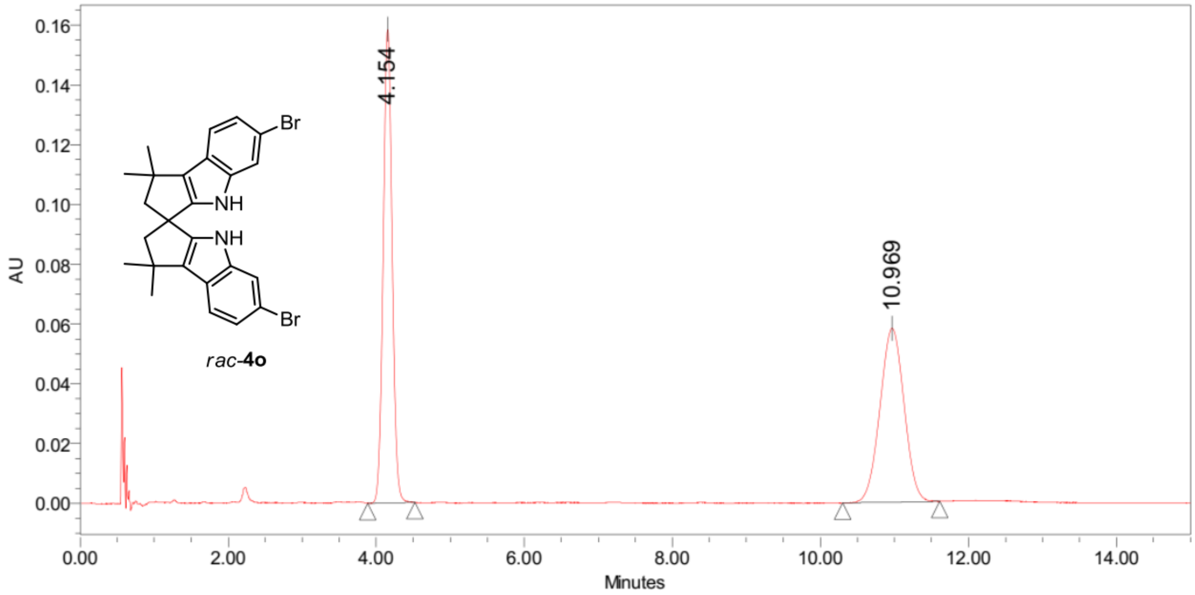

Peak Results

	Retention Time (min)	Area	% Area	Height	Int Type
1	4.154	1330204	50.26	158476	bb
2	10.969	1316382	49.74	58254	bb

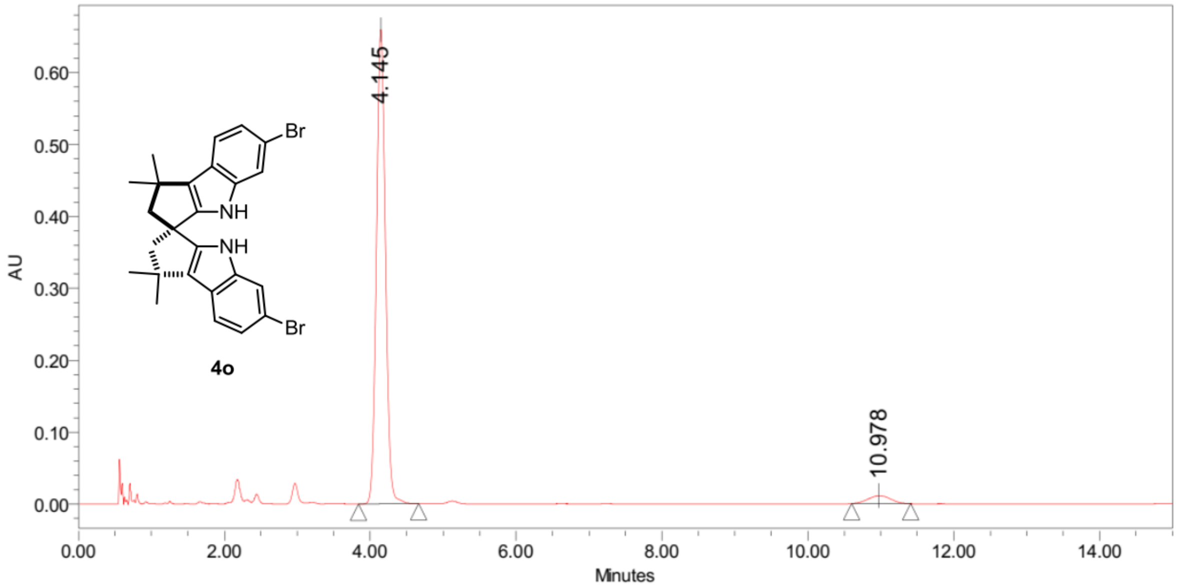

Peak Results

	Retention Time (min)	Area	% Area	Height	Int Type
1	4.145	5670539	95.91	659699	bb
2	10.978	241652	4.09	11122	bb

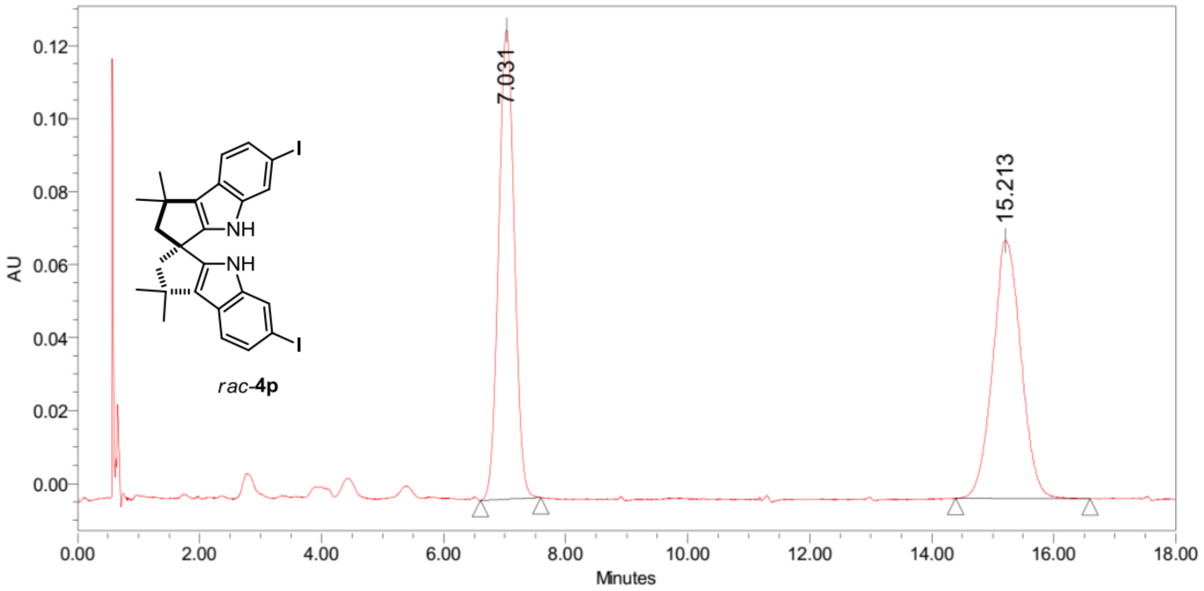

Peak Results

	Retention Time (min)	Area	% Area	Height	Int Type
1	7.031	2301397	50.36	128517	Bb
2	15.213	2268262	49.64	70732	bb

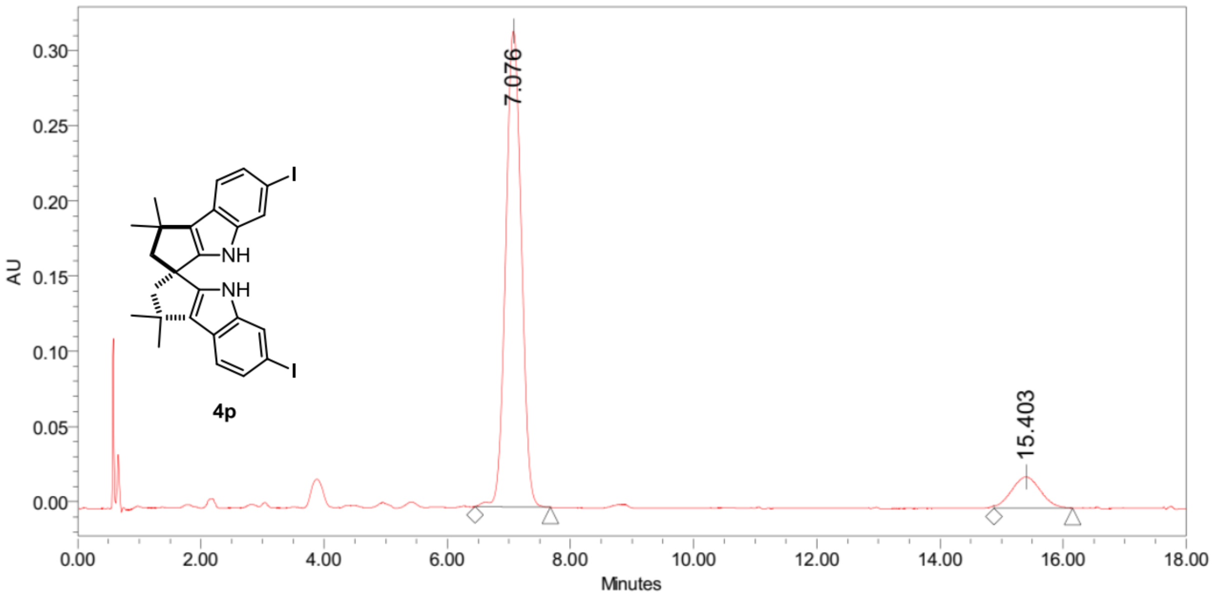

Peak Results

	Retention Time (min)	Area	% Area	Height	Int Type
1	7.076	5588872	89.28	316286	vb
2	15.403	671176	10.72	20597	Vb

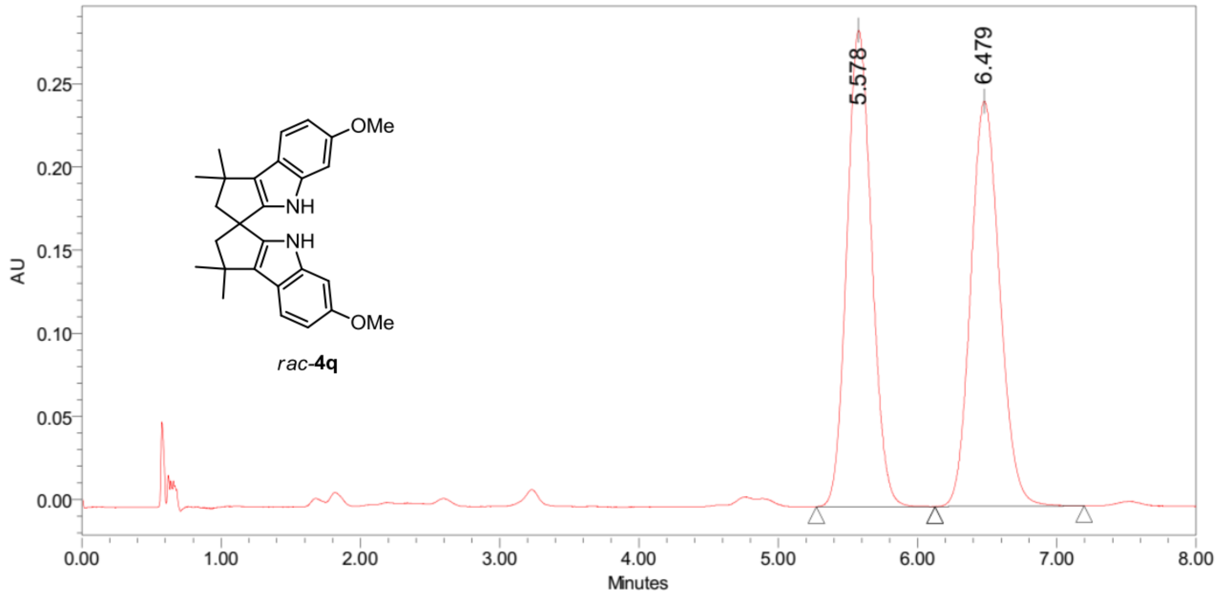

rac-**4q**

Peak Results

	Retention Time (min)	Area	% Area	Height	Int Type
1	5.578	3532047	50.22	285909	bB
2	6.479	3501426	49.78	243174	Bb

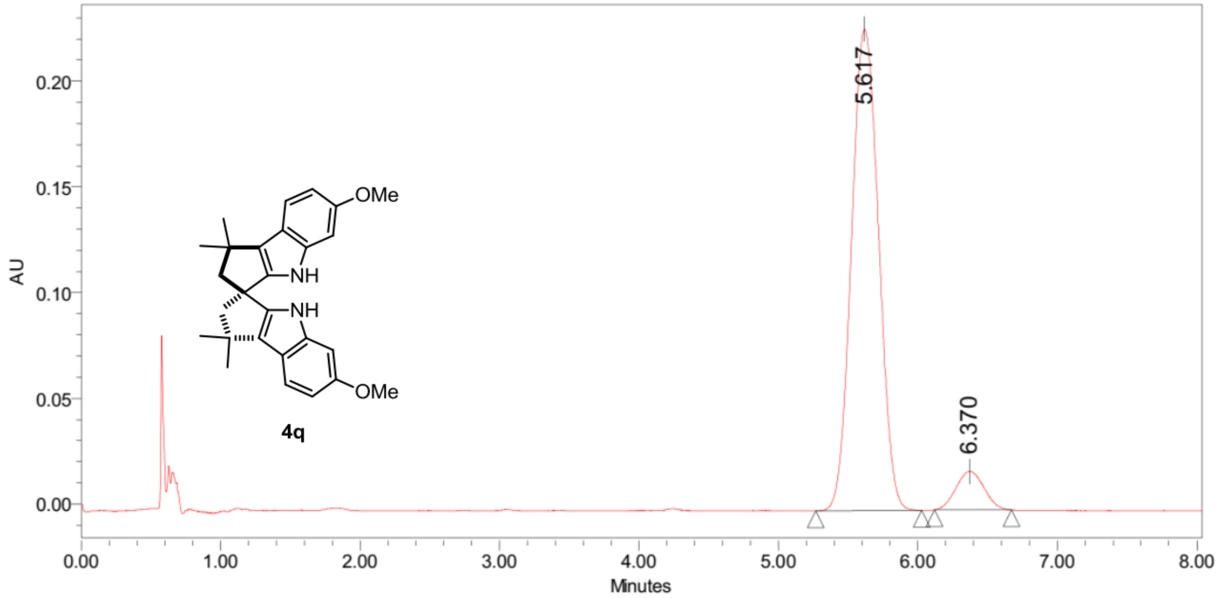

4q

Peak Results

	Retention Time (min)	Area	% Area	Height	Int Type
1	5.617	3073537	92.38	227797	bb
2	6.370	253537	7.62	18173	bb

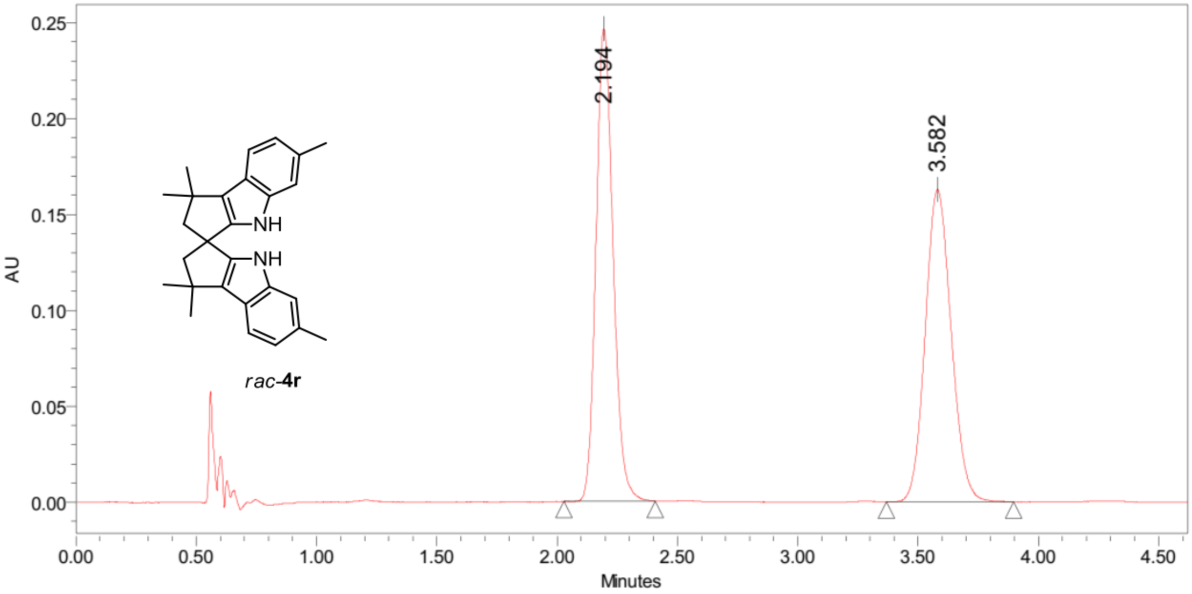

Peak Results

	Retention Time (min)	Area	% Area	Height	Int Type
1	2.194	1215448	49.85	246293	bb
2	3.582	1222563	50.15	162894	bb

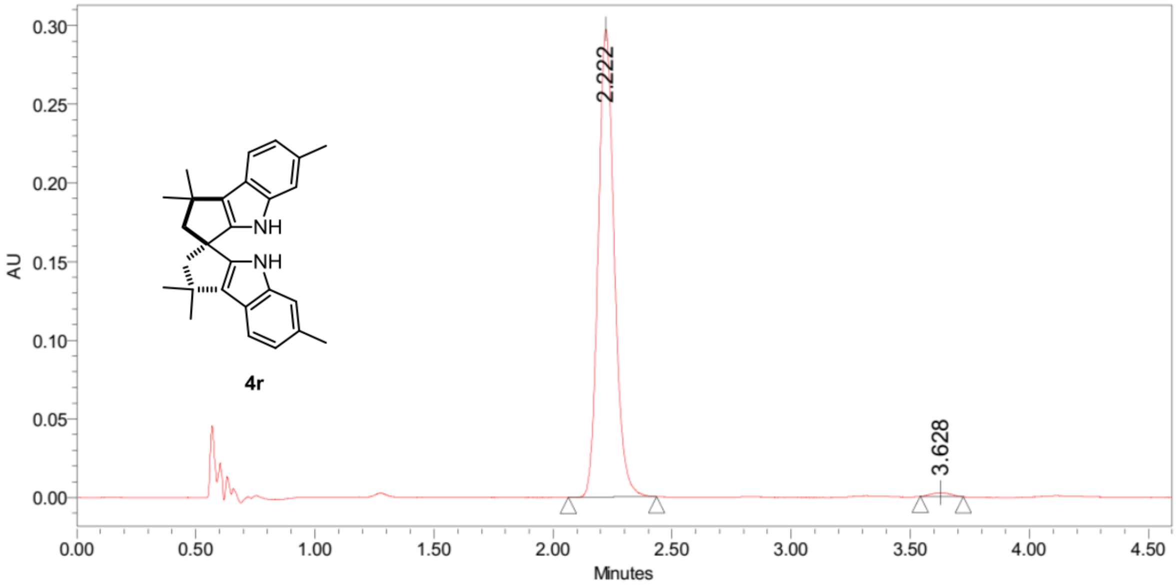

Peak Results

	Retention Time (min)	Area	% Area	Height	Int Type
1	2.222	1394844	98.99	297387	bb
2	3.628	14167	1.01	2507	bb

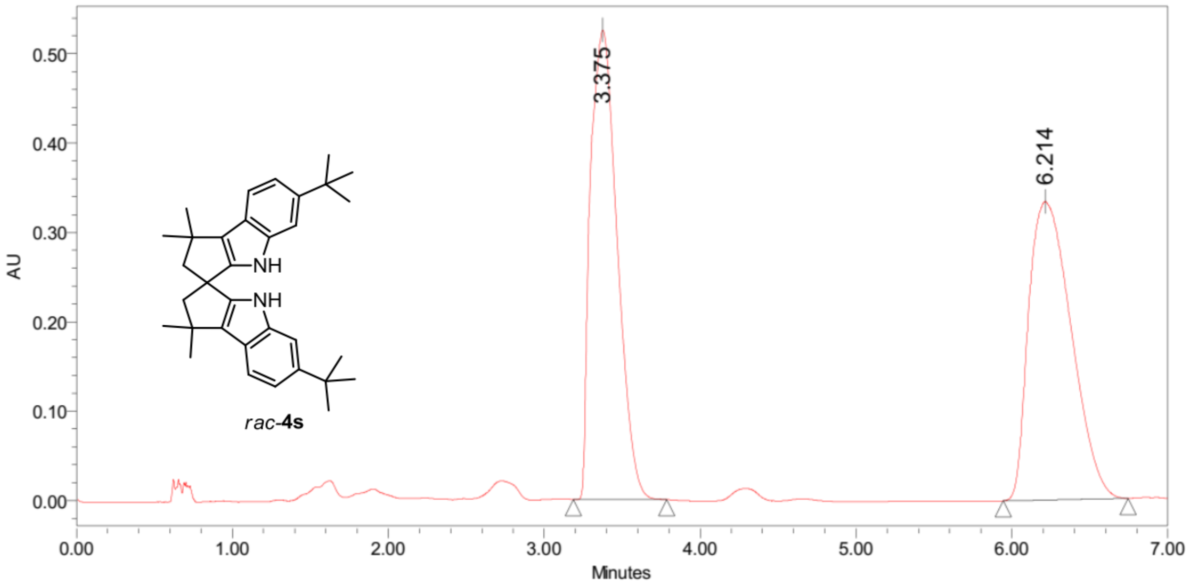

Peak Results

	Retention Time (min)	Area	% Area	Height	Int Type
1	3.375	6269713	49.75	525407	bb
2	6.214	6332907	50.25	334289	bb

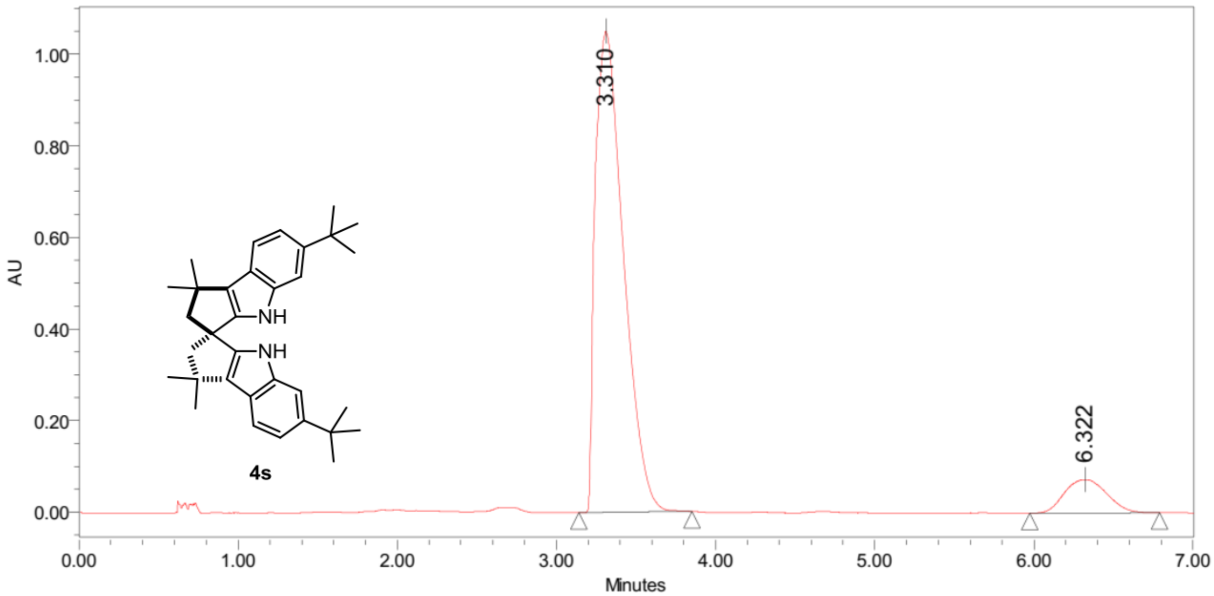

Peak Results

	Retention Time (min)	Area	% Area	Height	Int Type
1	3.310	12878381	90.56	1050535	bb
2	6.322	1342026	9.44	73032	bb

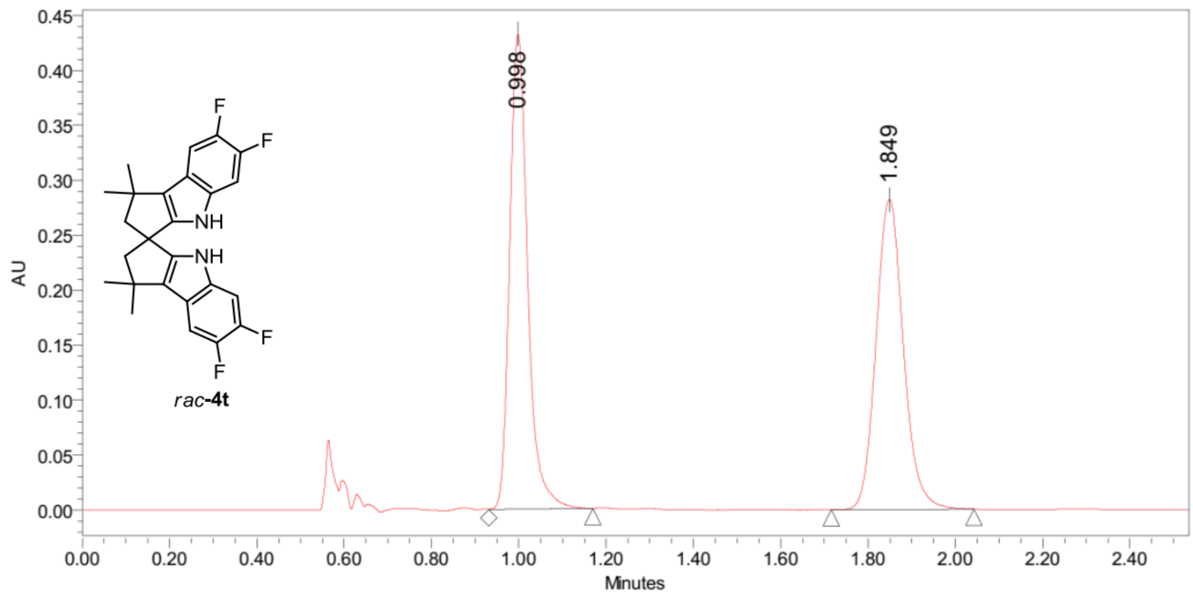

Peak Results

	Retention Time (min)	Area	% Area	Height	Int Type
1	0.998	1235169	50.07	432254	Vb
2	1.849	1231856	49.93	281915	bb

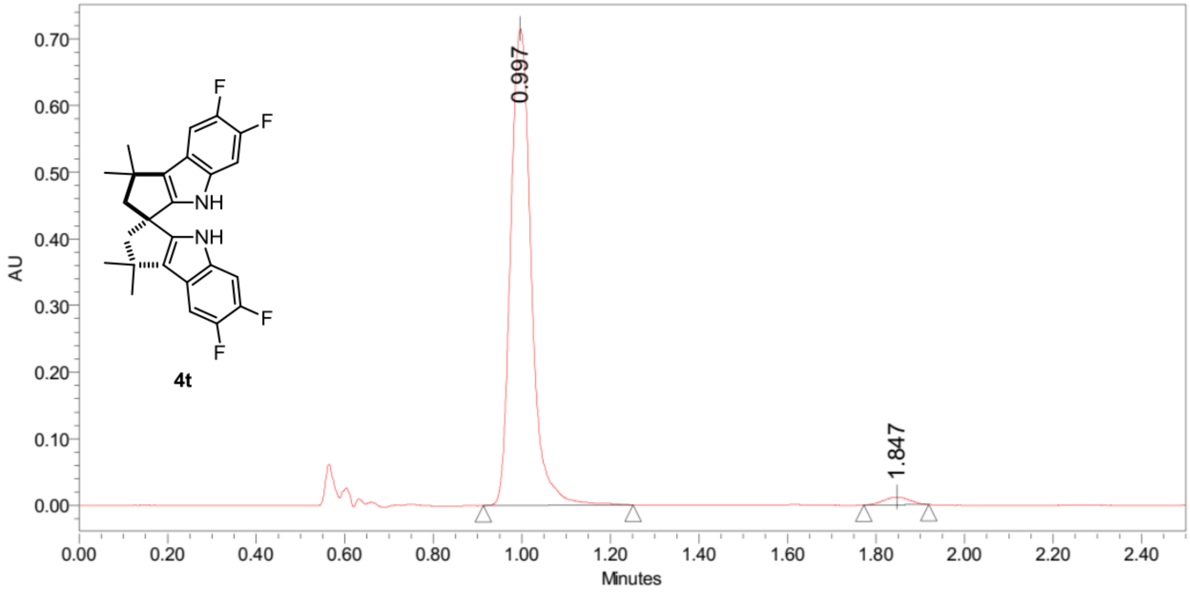

Peak Results

	Retention Time (min)	Area	% Area	Height	Int Type
1	0.997	2310698	98.00	715555	bb
2	1.847	47139	2.00	11501	bb

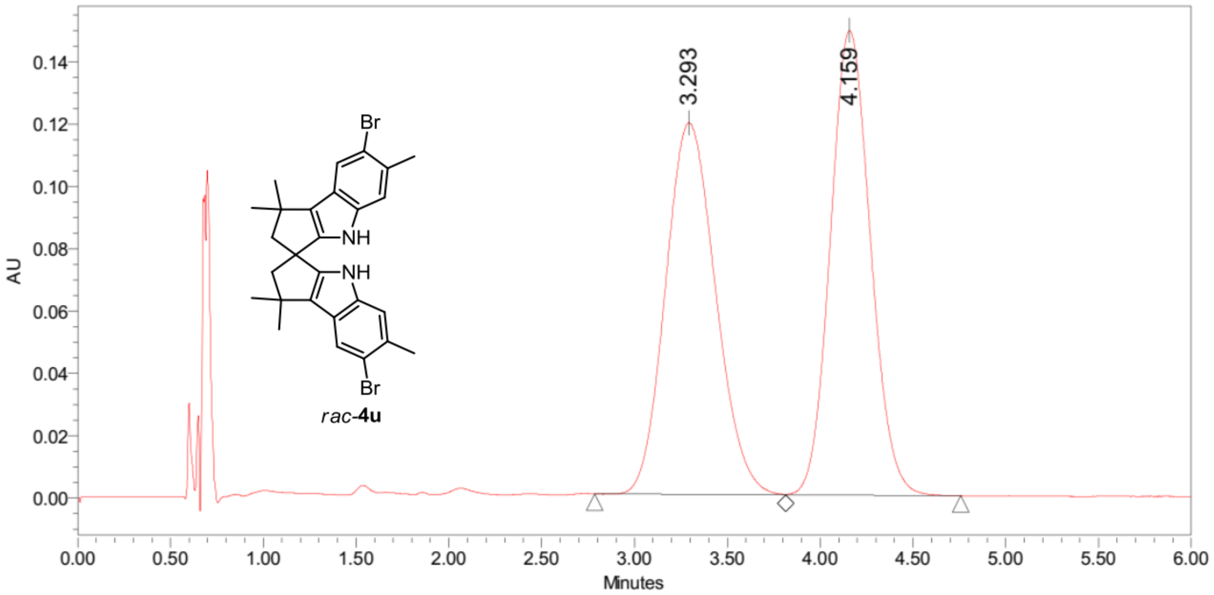

Peak Results

	Retention Time (min)	Area	% Area	Height	Int Type
1	3.293	2233454	49.89	119267	bV
2	4.159	2242914	50.11	149176	Vb

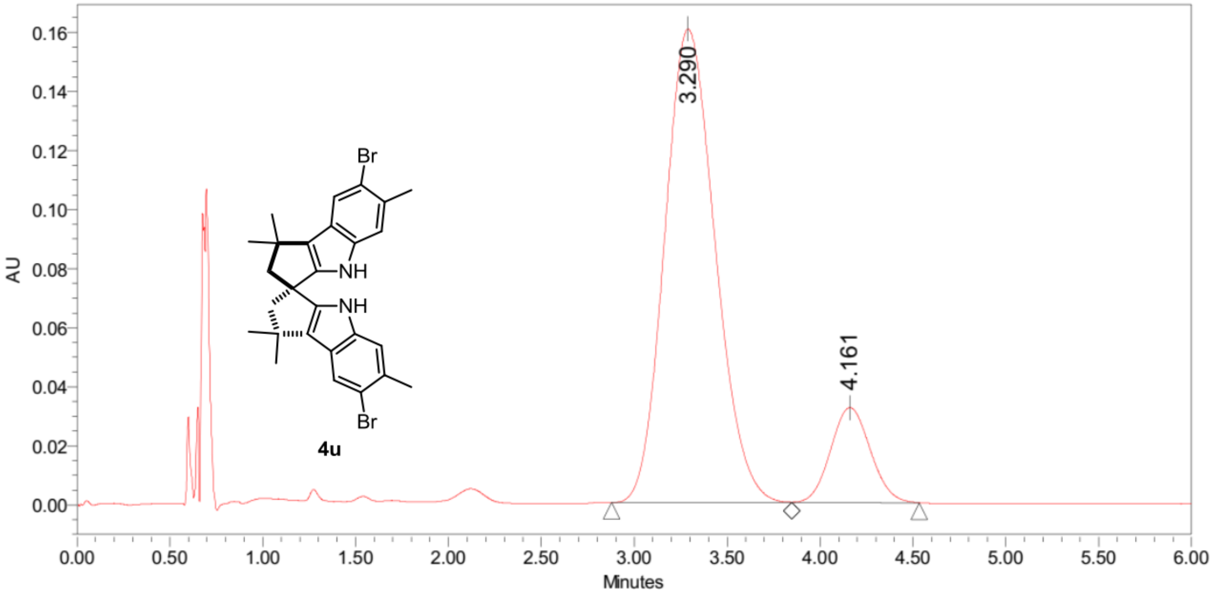

Peak Results

	Retention Time (min)	Area	% Area	Height	Int Type
1	3.290	3028140	86.34	160345	BV
2	4.161	479044	13.66	32206	Vb

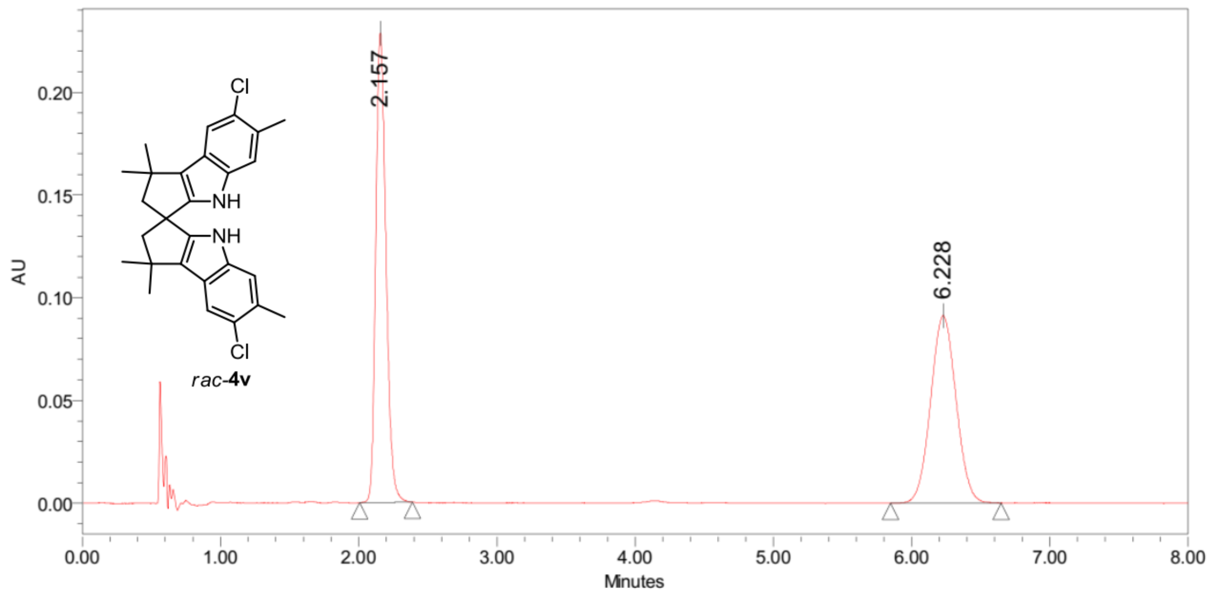

Peak Results

	Retention Time (min)	Area	% Area	Height	Int Type
1	2.157	1167345	50.06	228529	bb
2	6.228	1164633	49.94	91270	bb

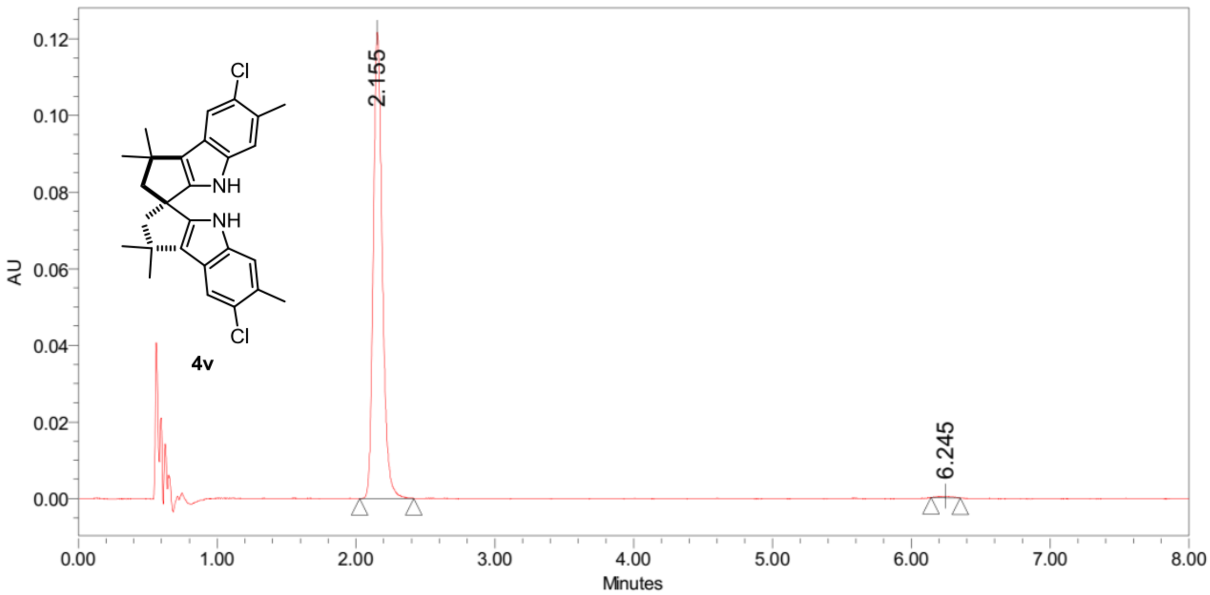

Peak Results

	Retention Time (min)	Area	% Area	Height	Int Type
1	2.155	546144	99.50	121717	bb
2	6.245	2765	0.50	412	bb

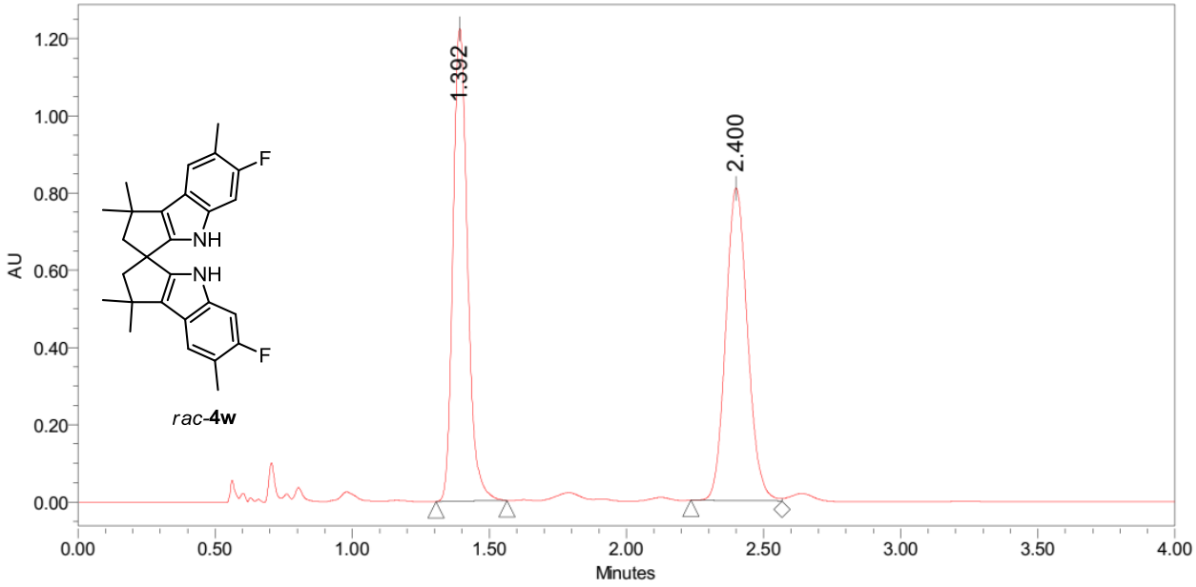

rac-**4w**

Peak Results

	Retention Time (min)	Area	% Area	Height	Int Type
1	1.392	4490810	49.99	1224269	bb
2	2.400	4492505	50.01	809191	bV

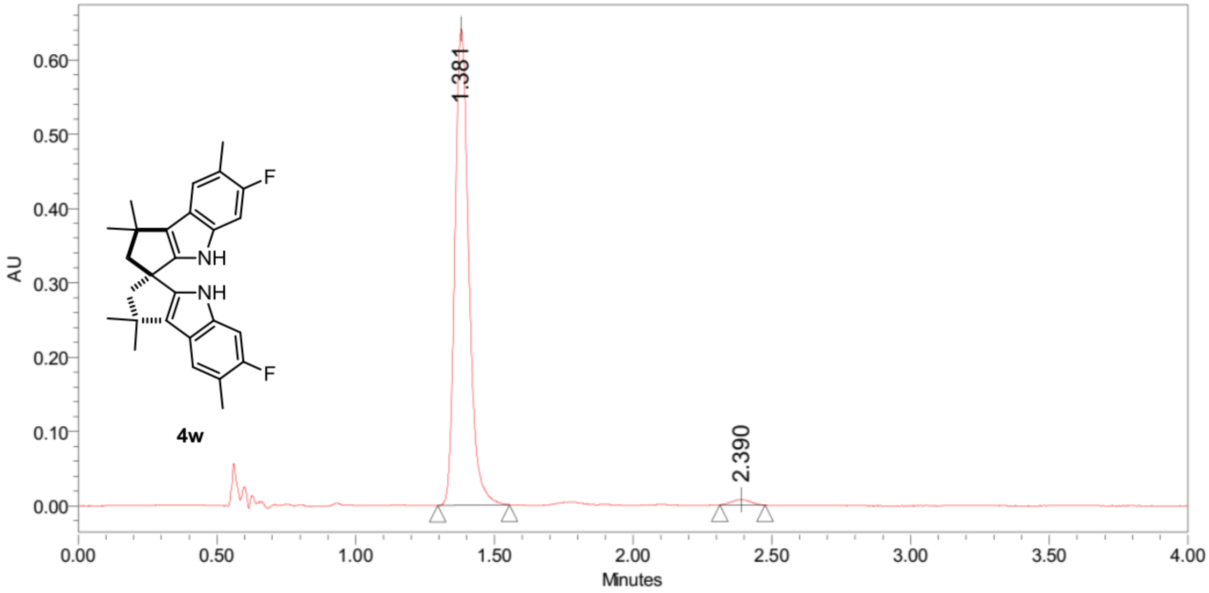

4w

Peak Results

	Retention Time (min)	Area	% Area	Height	Int Type
1	1.381	2228735	98.51	640688	bb
2	2.390	33791	1.49	7172	bb

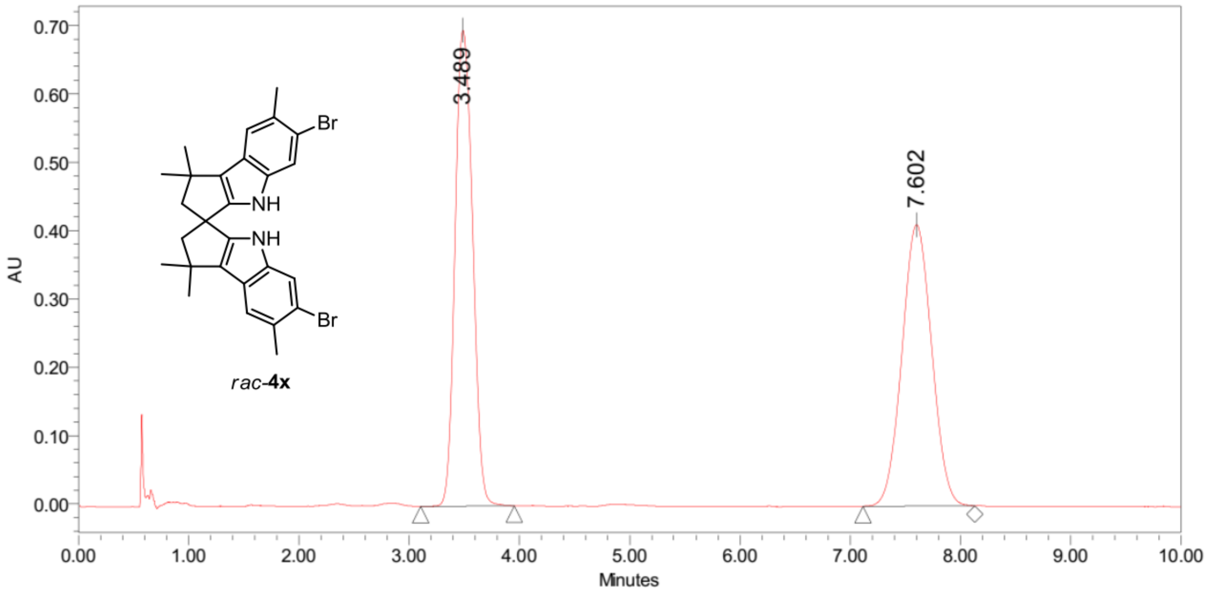

Peak Results

	Retention Time (min)	Area	% Area	Height	Int Type
1	3.489	7706288	49.88	696128	bb
2	7.602	7744396	50.12	411835	bV

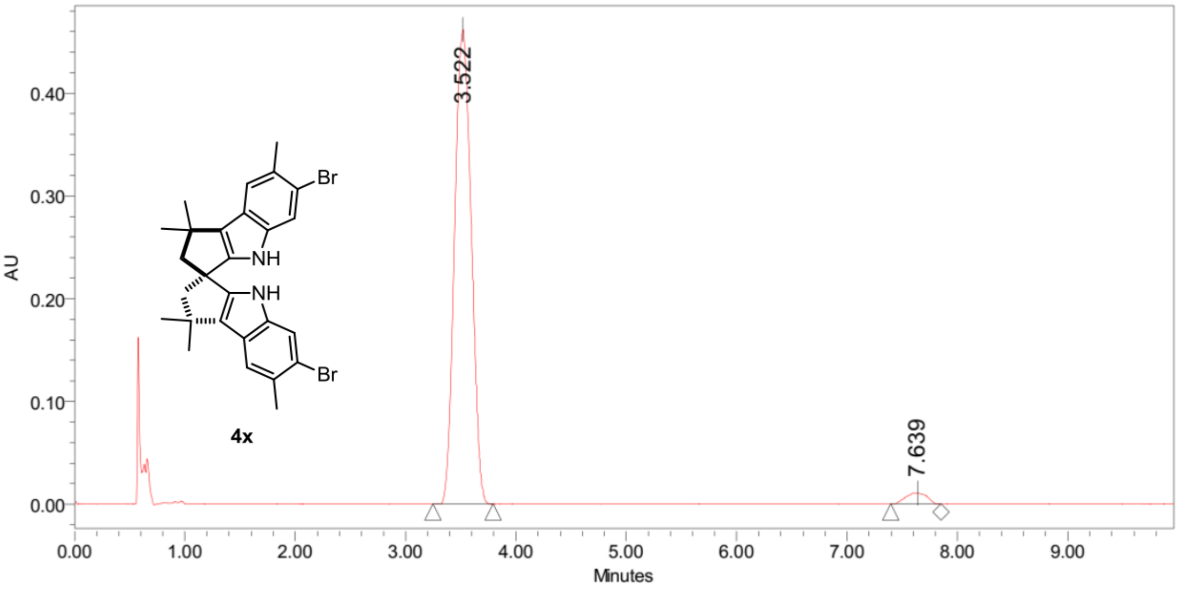

Peak Results

	Retention Time (min)	Area	% Area	Height	Int Type
1	3.522	4836892	97.10	461402	bb
2	7.639	144418	2.90	10424	bv

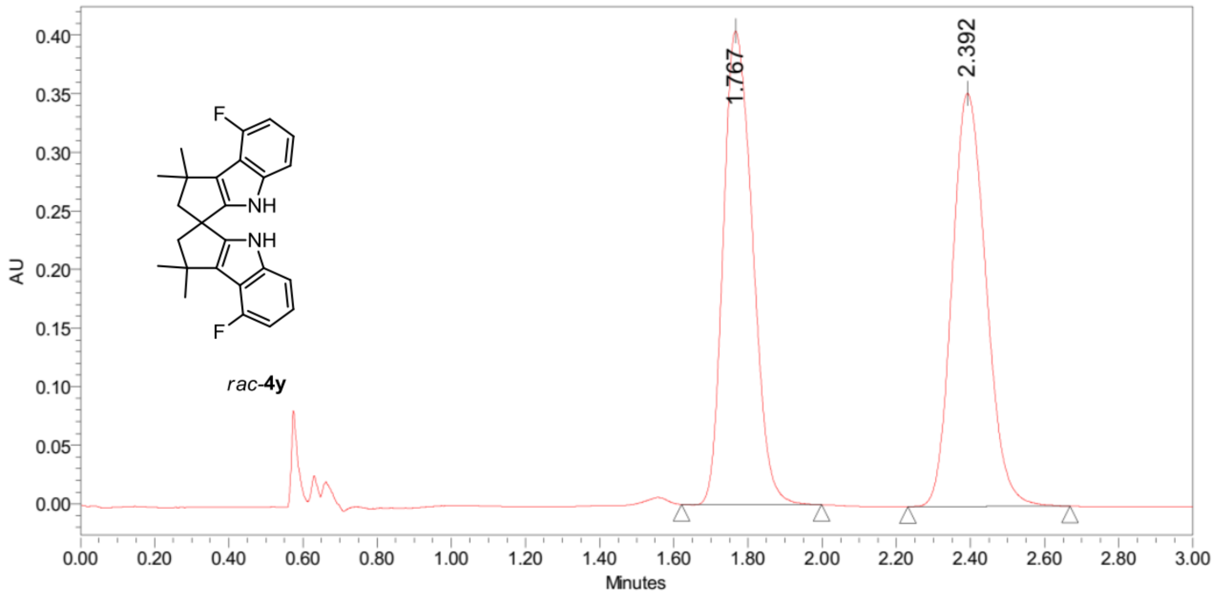

Peak Results

	Retention Time (min)	Area	% Area	Height	Int Type
1	1.767	2213998	49.67	404049	bb
2	2.392	2243193	50.33	352566	bb

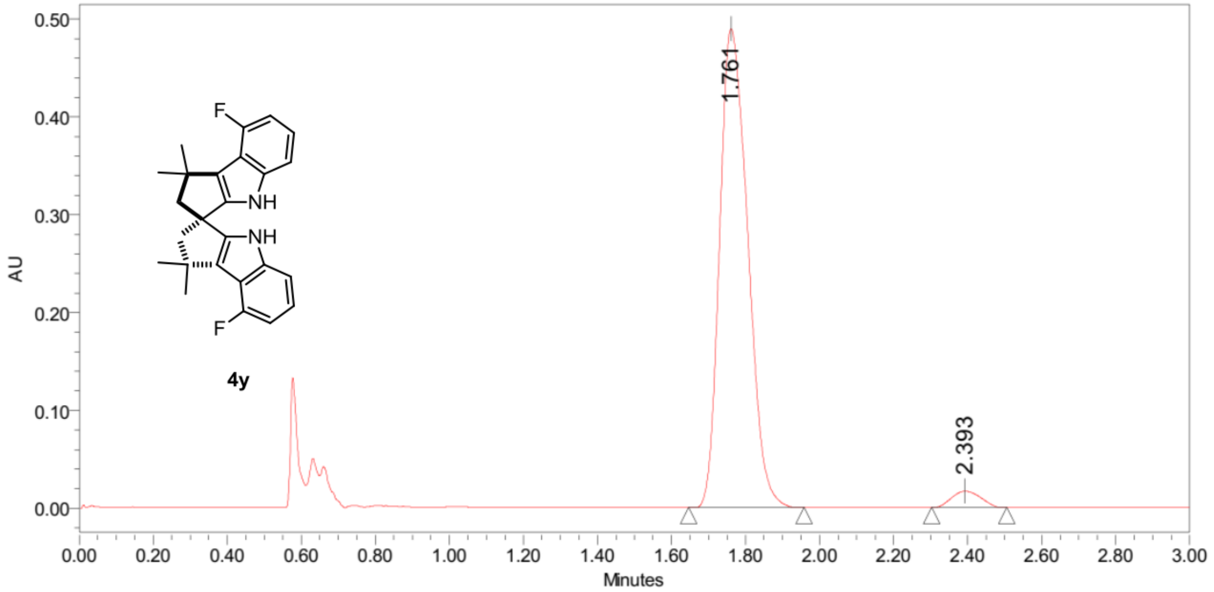

Peak Results

	Retention Time (min)	Area	% Area	Height	Int Type
1	1.761	2574265	96.69	489174	bb
2	2.393	88029	3.31	16462	bb

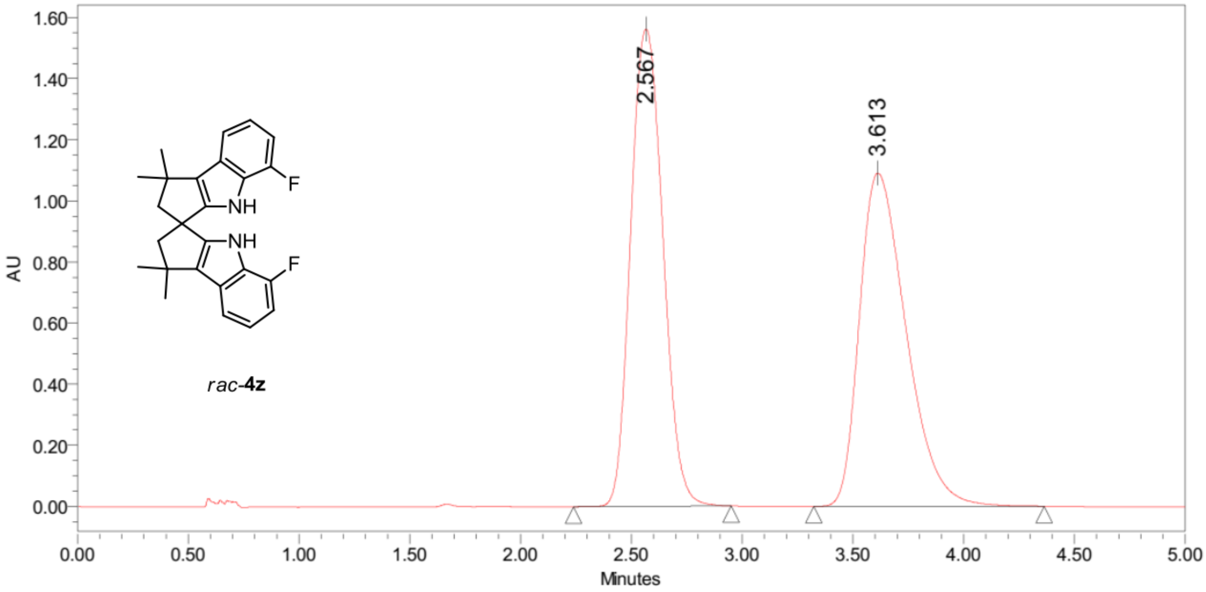

Peak Results

	Retention Time (min)	Area	% Area	Height	Int Type
1	2.567	15840466	49.82	1561739	bb
2	3.613	15956404	50.18	1091129	bb

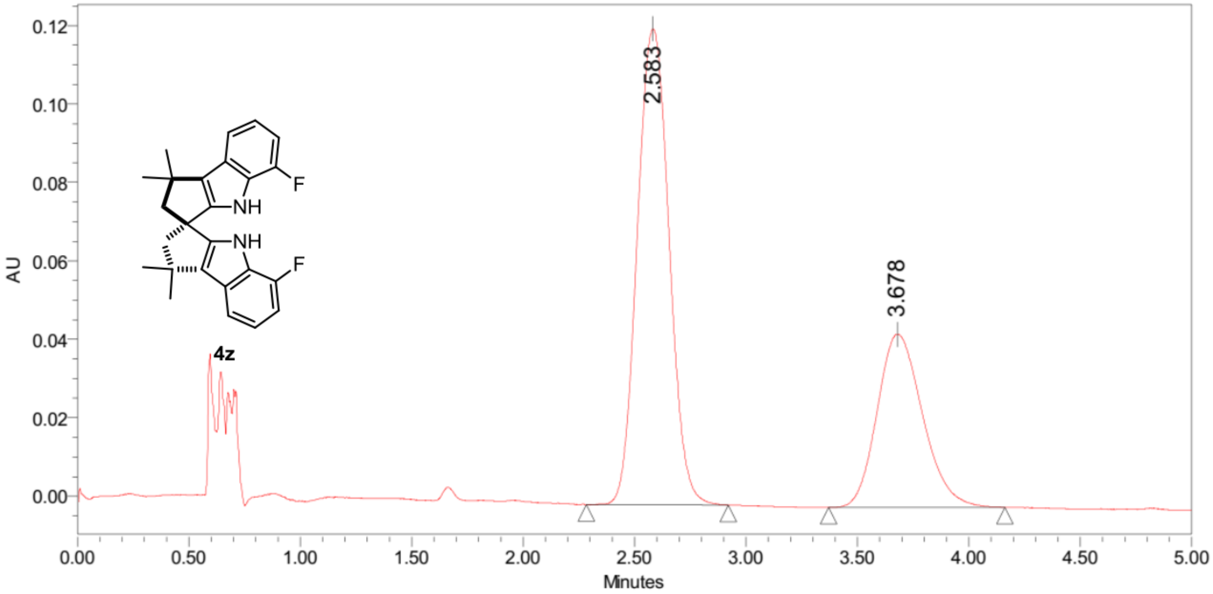

Peak Results

	Retention Time (min)	Area	% Area	Height	Int Type
1	2.583	1204855	65.81	121347	bb
2	3.678	625897	34.19	44184	bb

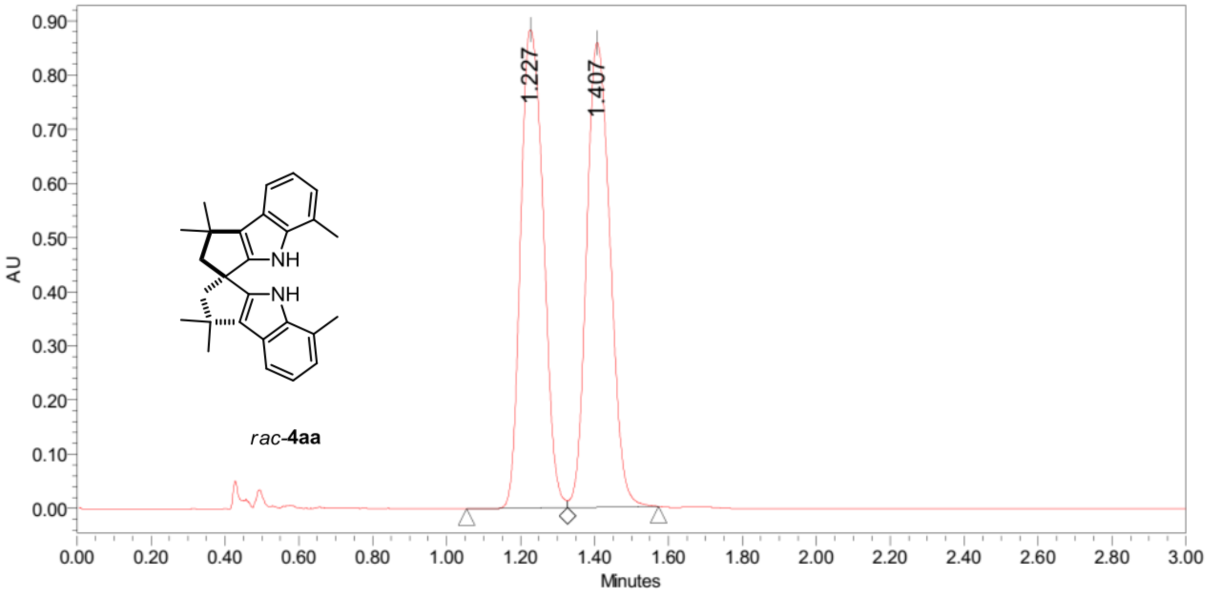

Peak Results

	Retention Time (min)	Area	% Area	Height	Int Type
1	1.227	3776227	49.83	883064	bV
2	1.407	3802351	50.17	857760	Vb

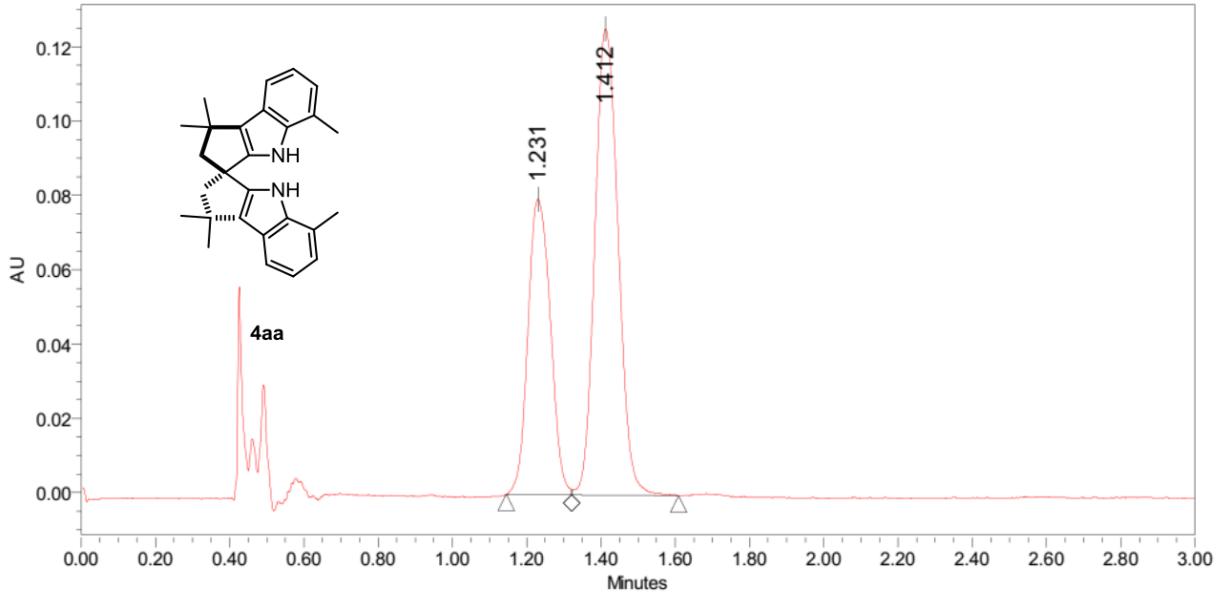

Peak Results

	Retention Time (min)	Area	% Area	Height	Int Type
1	1.231	334140	37.93	79474	bv
2	1.412	546716	62.07	125465	vb

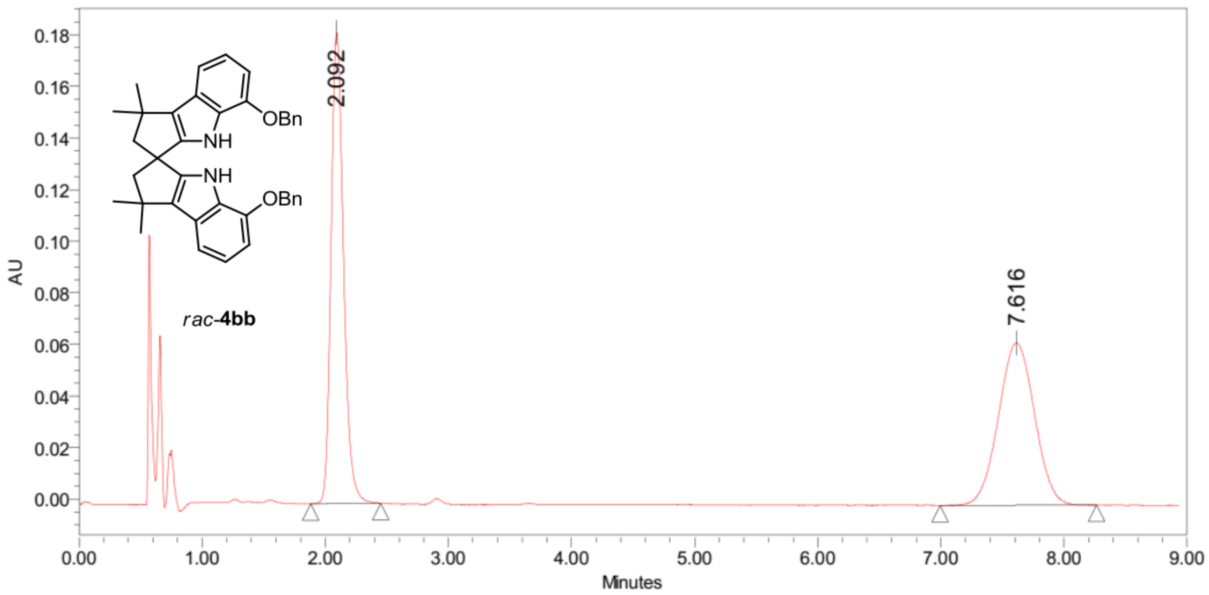

Peak Results

	Retention Time (min)	Area	% Area	Height	Int Type
1	2.092	1288440	50.02	182524	bb
2	7.616	1287497	49.98	62959	bb

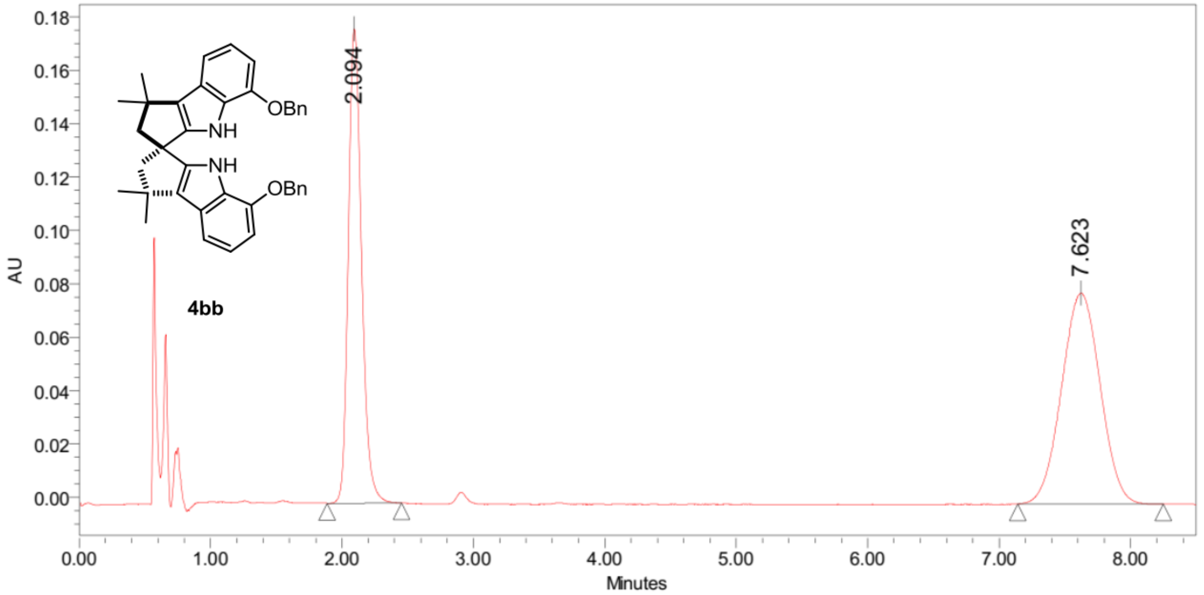

Peak Results

	Retention Time (min)	Area	% Area	Height	Int Type
1	2.094	1246438	43.63	177734	bb
2	7.623	1610530	56.37	79042	bb

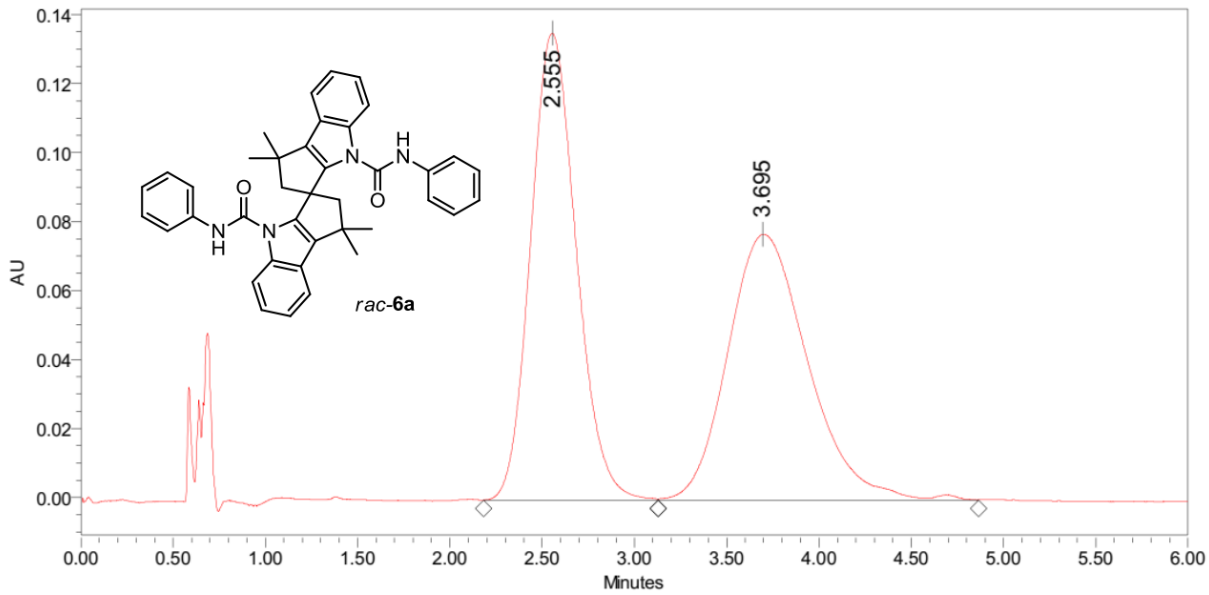

Peak Results

	Retention Time (min)	Area	% Area	Height	Int Type
1	2.555	2334259	50.15	135408	VV
2	3.695	2320721	49.85	77089	Vv

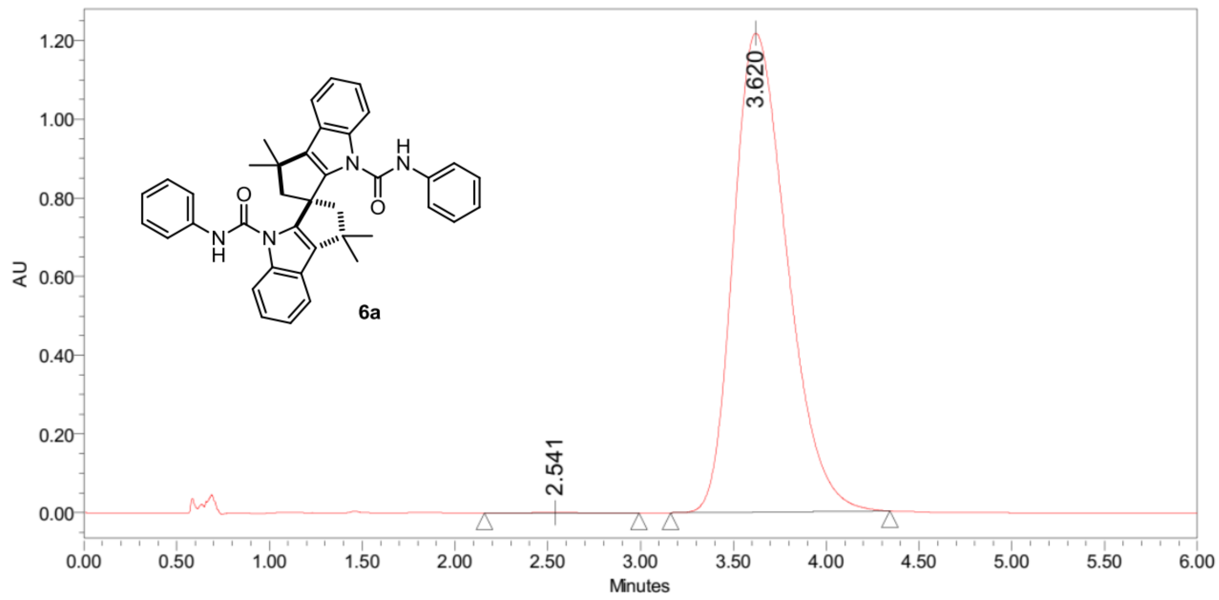

Peak Results

	Retention Time (min)	Area	% Area	Height	Int Type
1	2.541	16725	0.07	1215	bb
2	3.620	24439135	99.93	1216608	bb

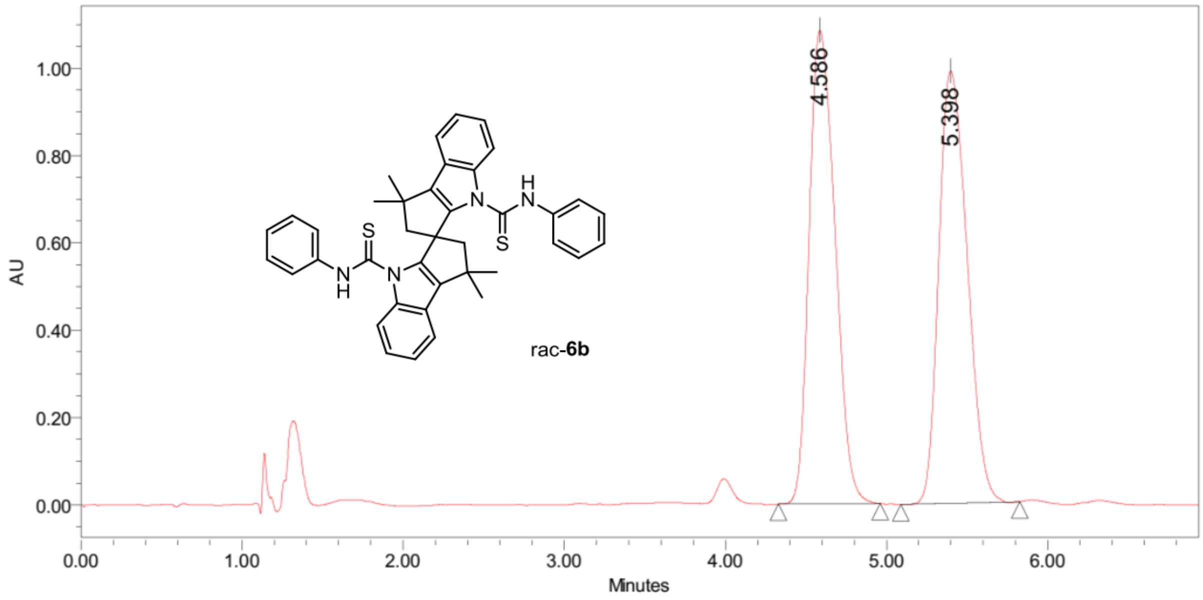

Peak Results

	Retention Time (min)	Area	% Area	Height	Int Type
1	4.586	12170416	50.10	1084652	bb
2	5.398	12122524	49.90	990237	bb

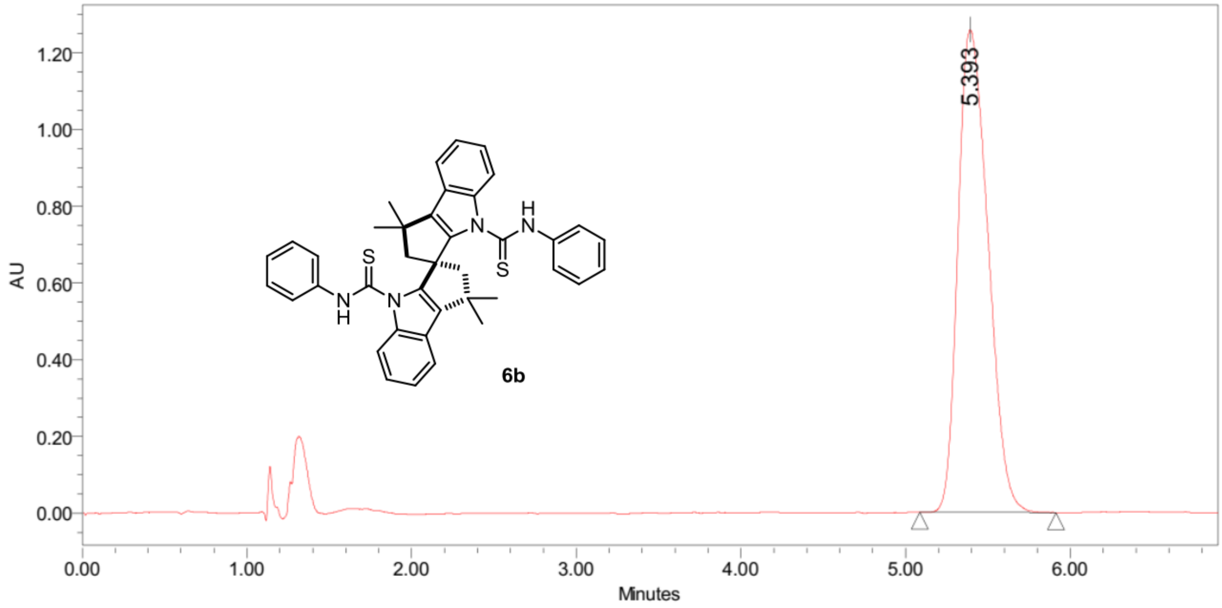

Peak Results

	Retention Time (min)	Area	% Area	Height	Int Type
1	5.393	15935839	100.00	1258606	bb

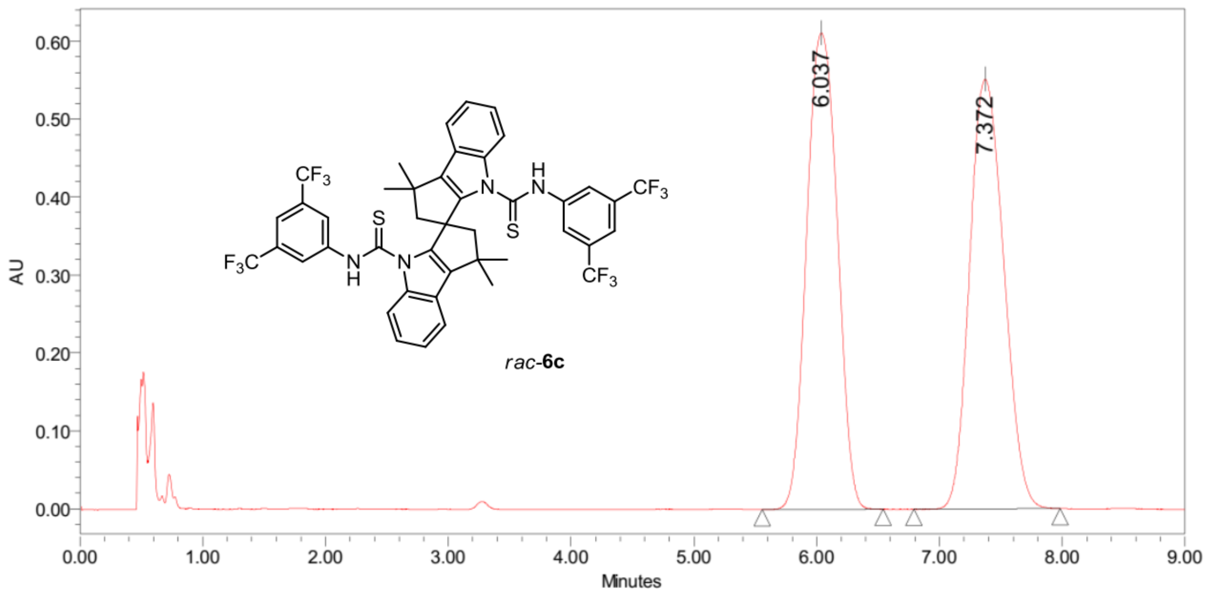

Peak Results

	Retention Time (min)	Area	% Area	Height	Int Type
1	6.037	10668648	50.01	611271	bb
2	7.372	10664323	49.99	551404	bb

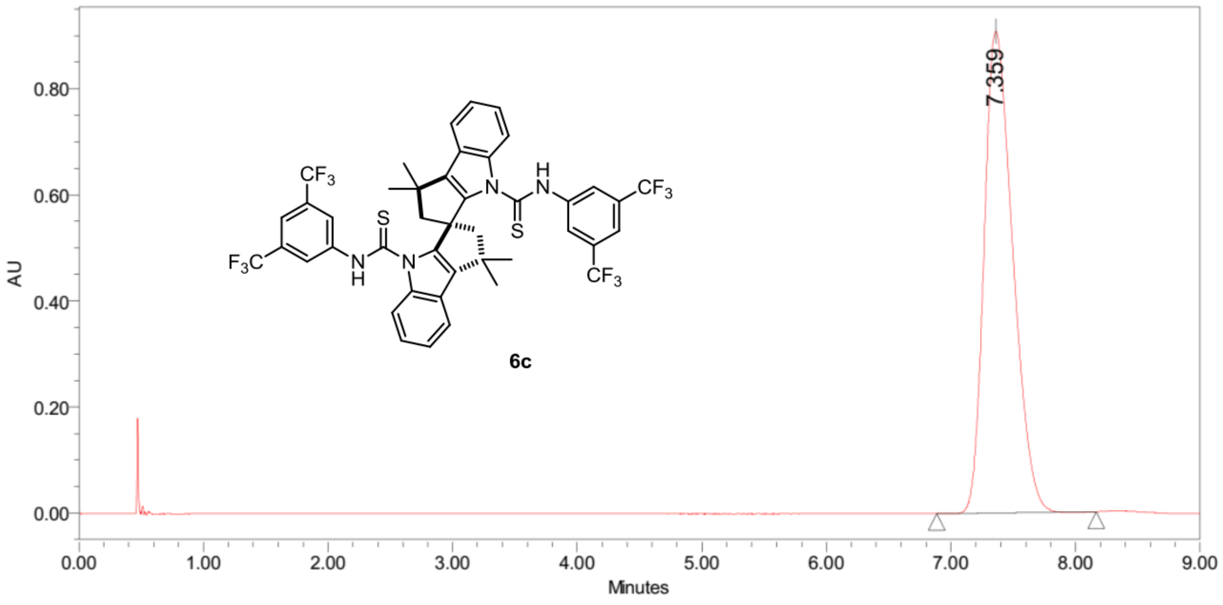

Peak Results

	Retention Time (min)	Area	% Area	Height	Int Type
1	7.359	14494716	100.00	908184	bb

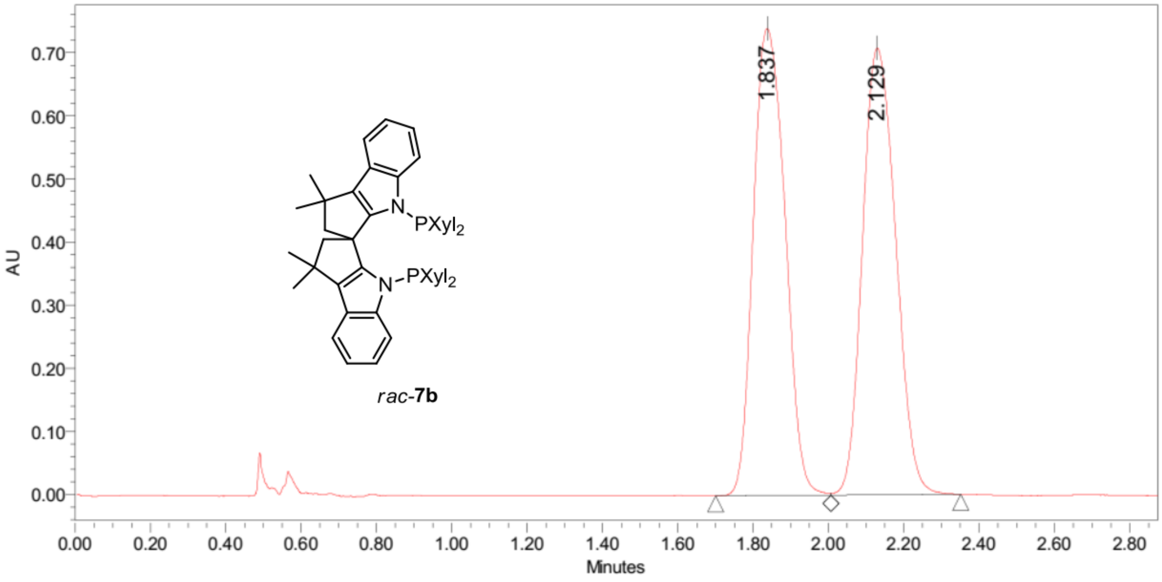

Peak Results

	Retention Time (min)	Area	% Area	Height	Int Type
1	1.837	4332003	49.96	739434	bV
2	2.129	4338122	50.04	707465	Vb

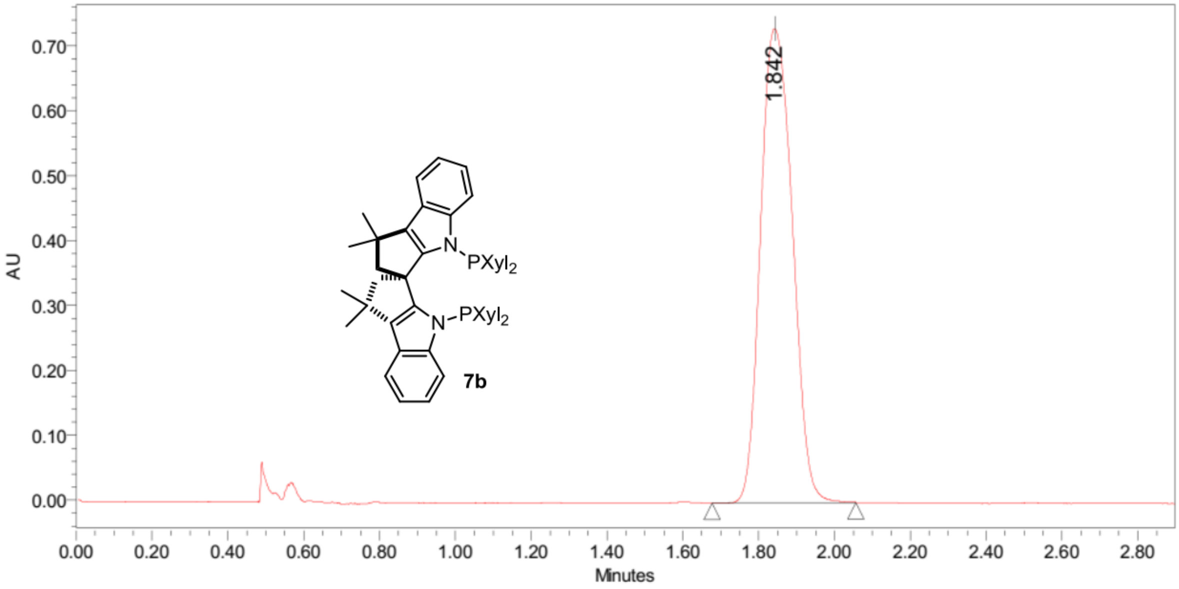

Peak Results

	Retention Time (min)	Area	% Area	Height	Int Type
1	1.842	4251432	100.00	730278	bb

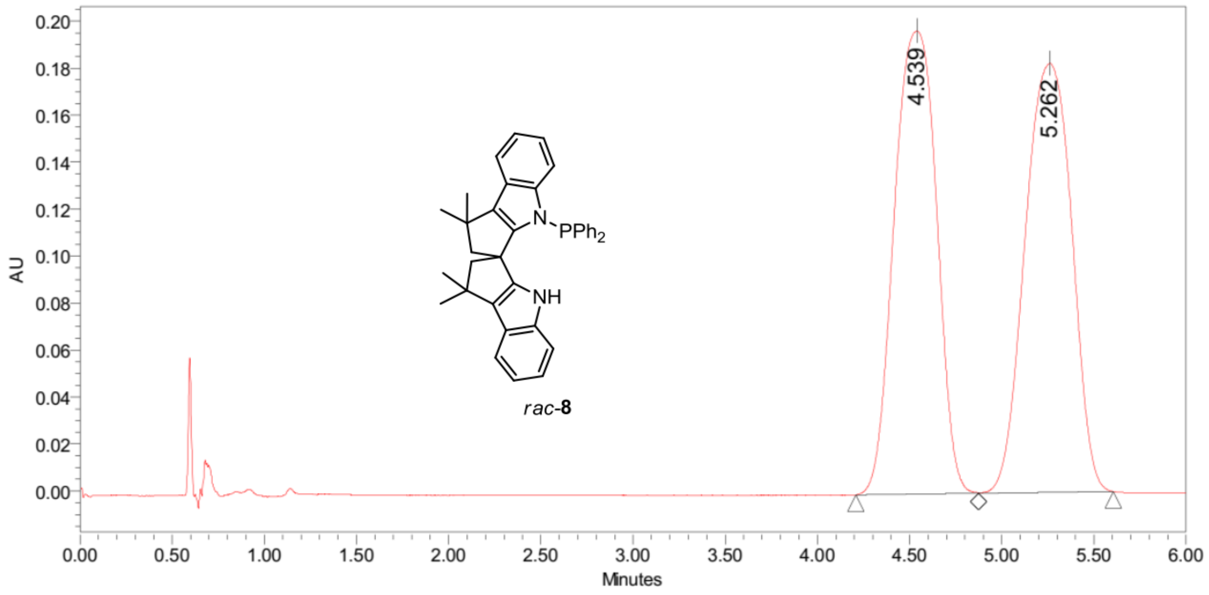

Peak Results

	Retention Time (min)	Area	% Area	Height	Int Type
1	4.539	3168764	49.58	197187	bv
2	5.262	3222137	50.42	182646	vb

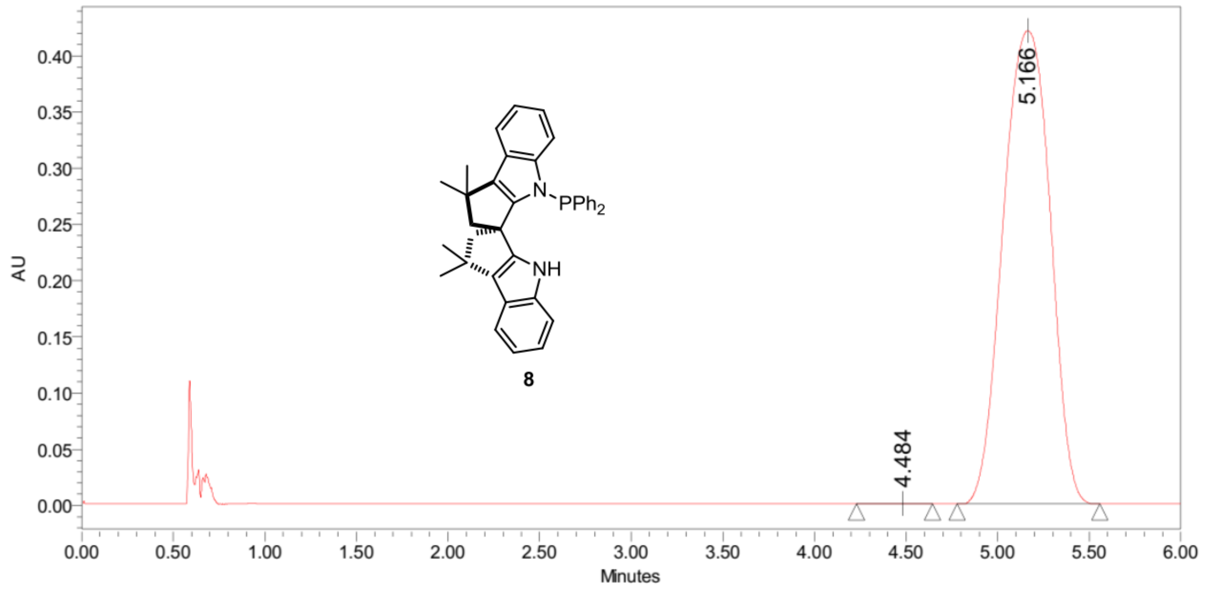

Peak Results

	Retention Time (min)	Area	% Area	Height	Int Type
1	4.484	1803	0.02	338	bb
2	5.166	7552655	99.98	420916	Bb

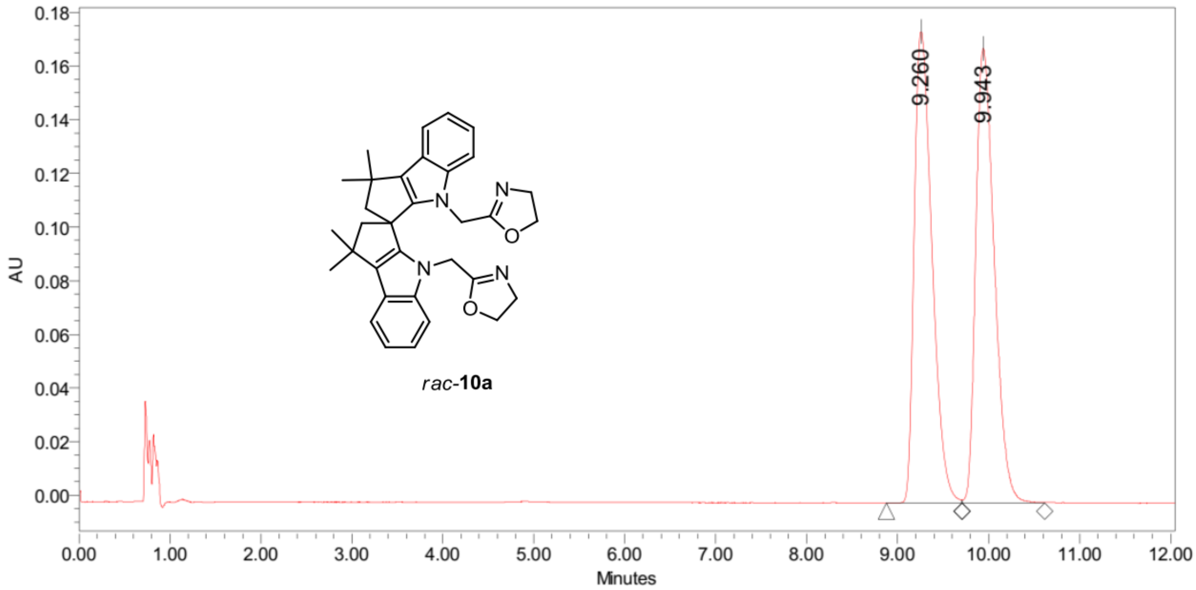

Peak Results

	Retention Time (min)	Area	% Area	Height	Int Type
1	9.260	2385044	49.88	175662	bV
2	9.943	2396896	50.12	169439	VV

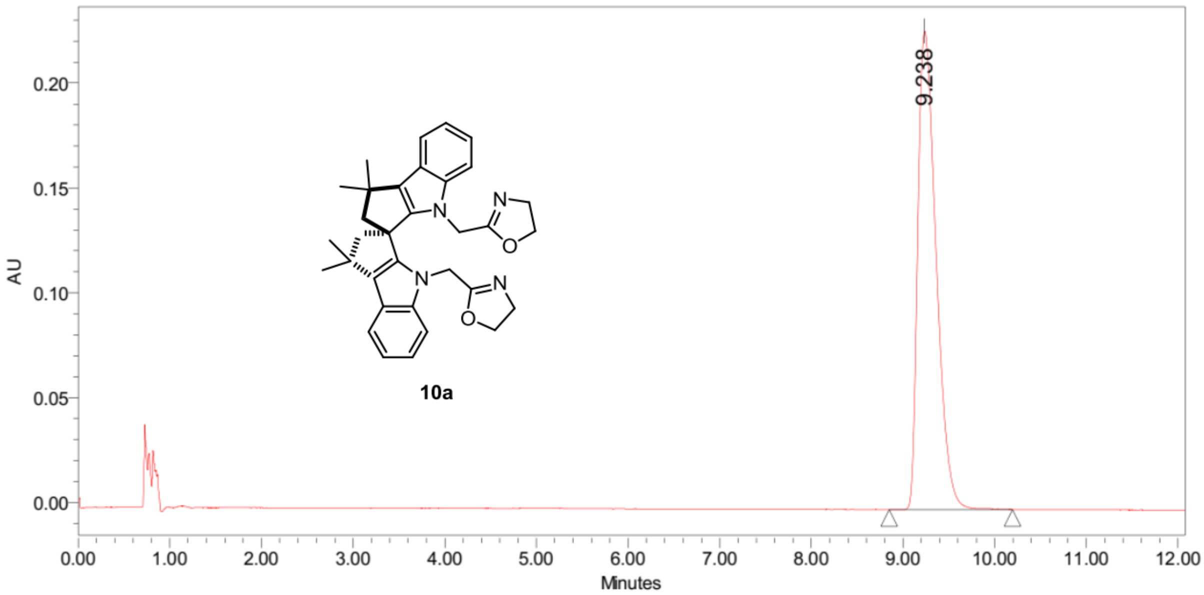

Peak Results

	Retention Time (min)	Area	% Area	Height	Int Type
1	9.238	3163956	100.00	228111	bb

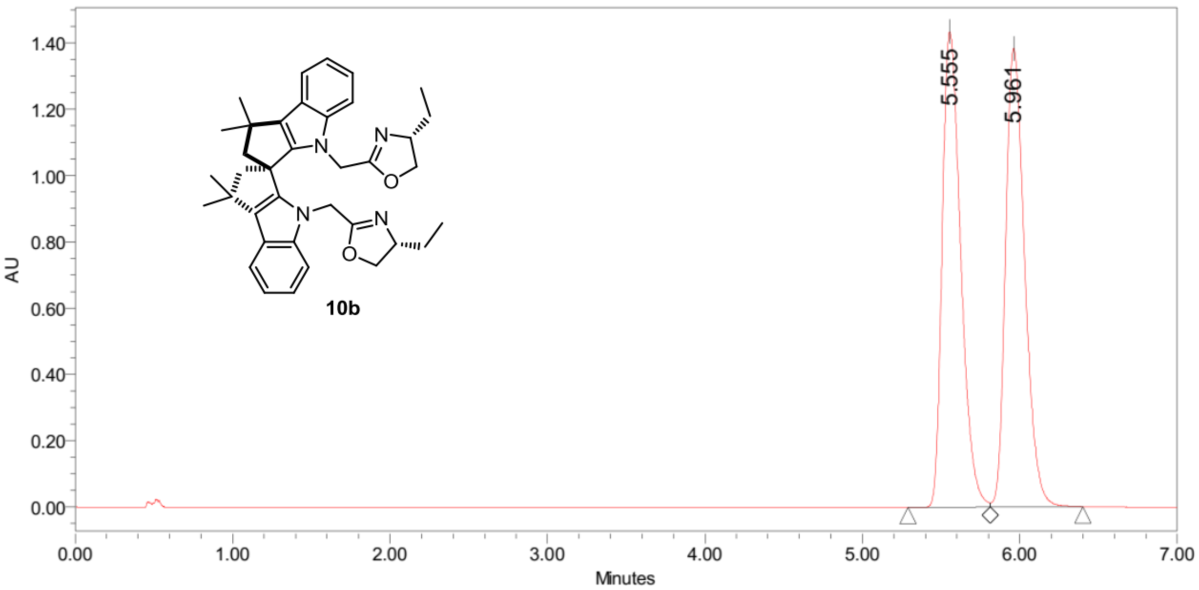

Peak Results

	Retention Time (min)	Area	% Area	Height	Int Type
1	5.555	11654778	49.84	1434650	bV
2	5.961	11730696	50.16	1383019	Vb

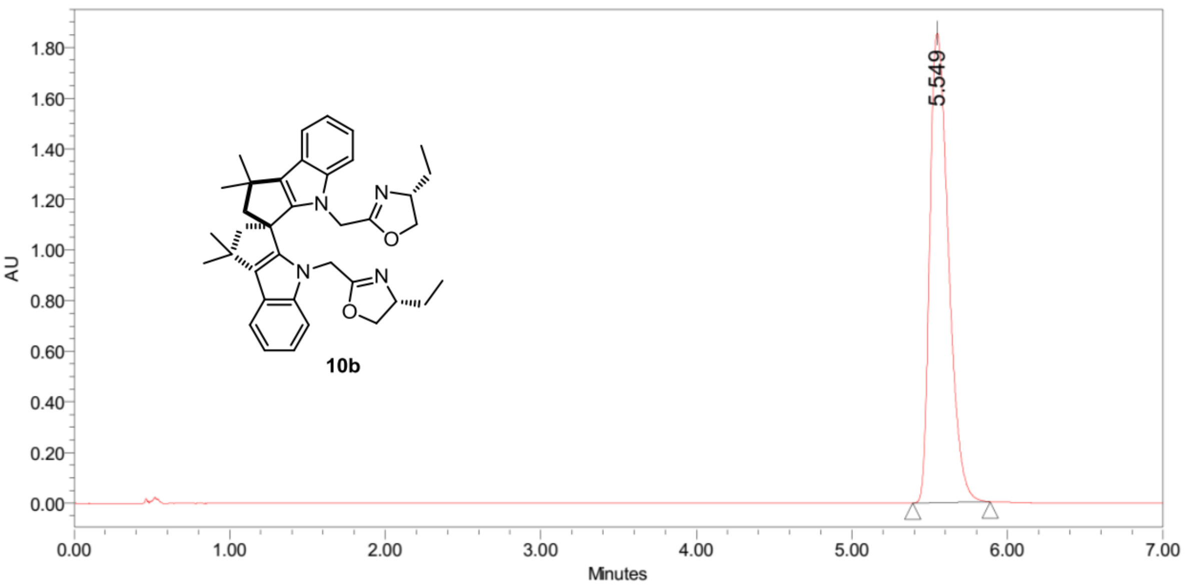

Peak Results

	Retention Time (min)	Area	% Area	Height	Int Type
1	5.549	15397068	100.00	1854434	bb

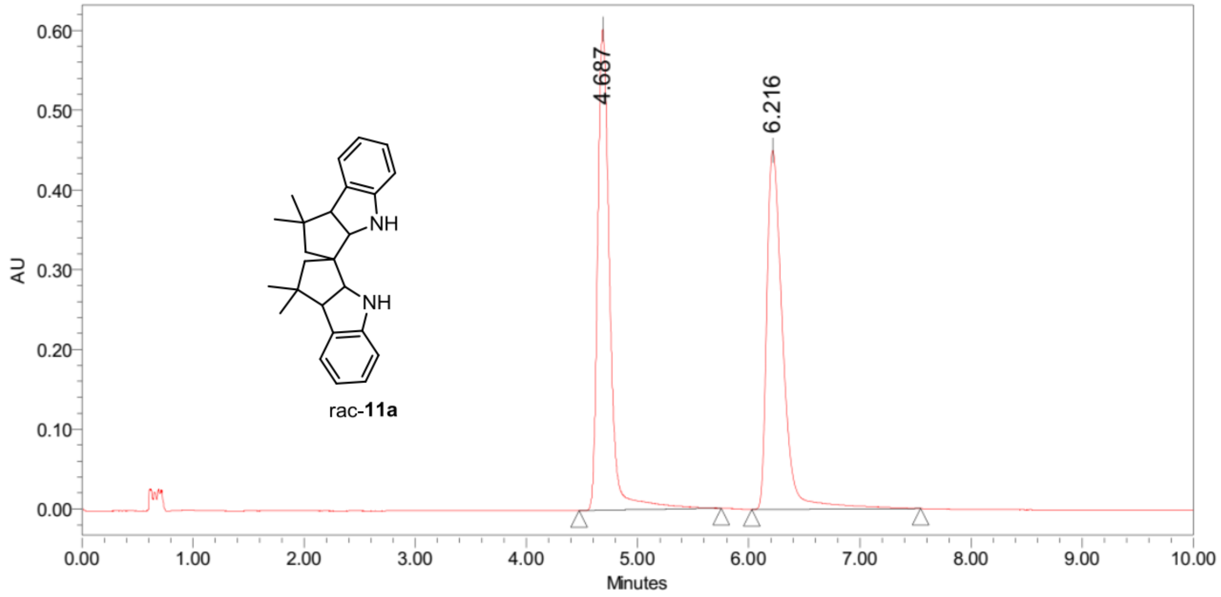

Peak Results

	Retention Time (min)	Area	% Area	Height	Int Type
1	4.687	4570824	50.30	602768	bb
2	6.216	4515429	49.70	449913	bb

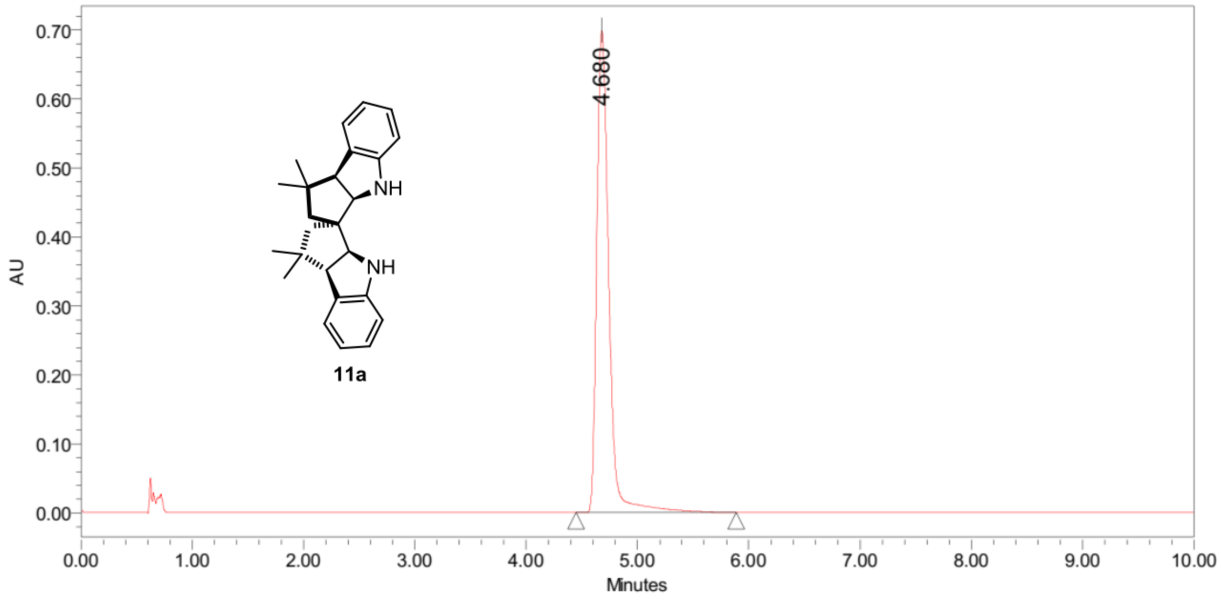

Peak Results

	Retention Time (min)	Area	% Area	Height	Int Type
1	4.680	5249010	100.00	698774	bb

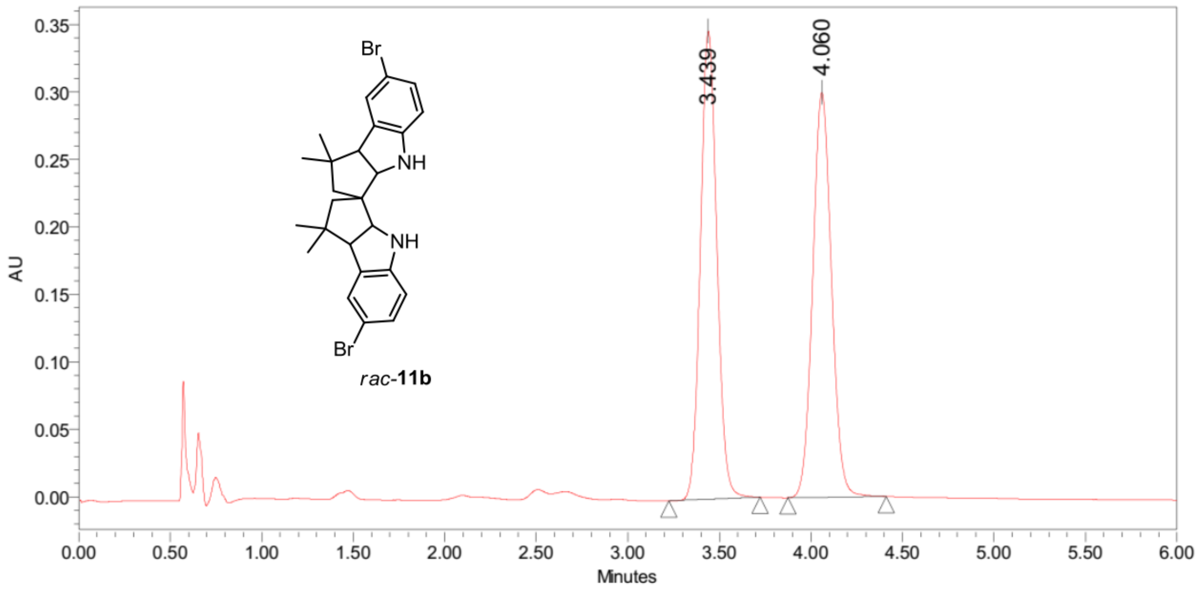

Peak Results

	Retention Time (min)	Area	% Area	Height	Int Type
1	3.439	2165280	50.18	346661	bb
2	4.060	2150147	49.82	300019	bb

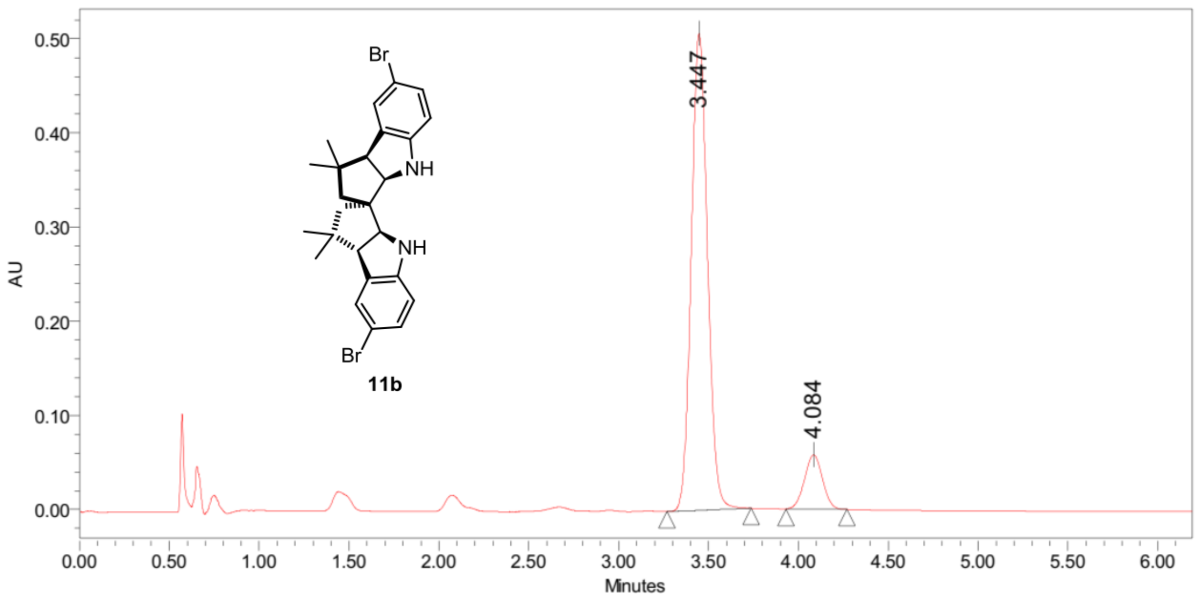

Peak Results

	Retention Time (min)	Area	% Area	Height	Int Type
1	3.447	3161311	88.70	506643	bb
2	4.084	402894	11.30	57758	bb

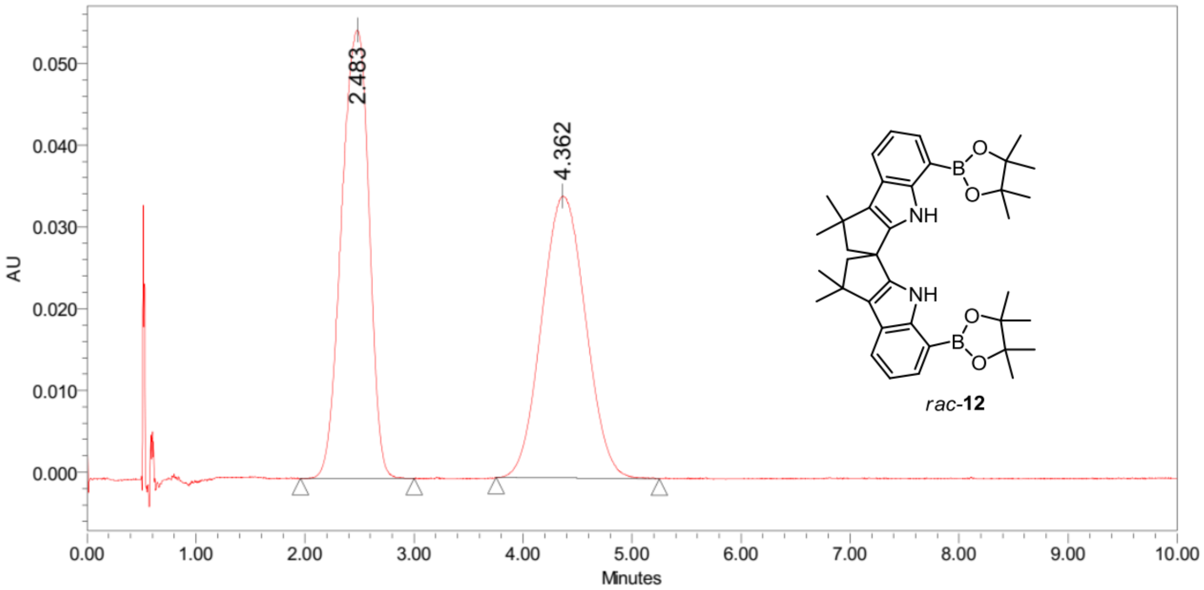

Peak Results

	Retention Time (min)	Area	% Area	Height	Int Type
1	2.483	968539	50.04	54814	bb
2	4.362	966896	49.96	34472	bb

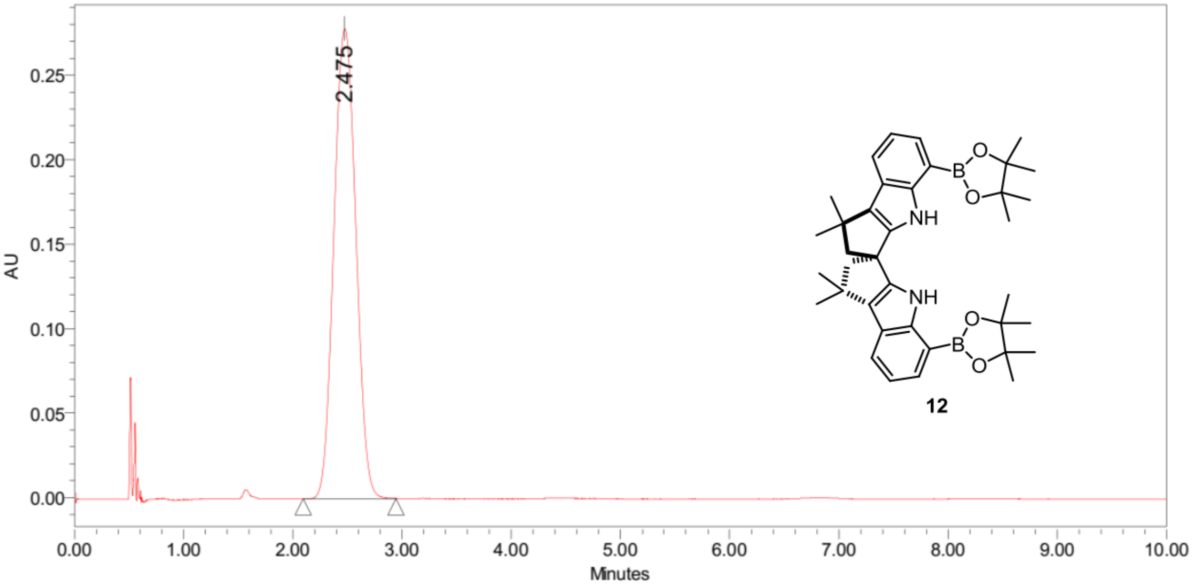

Peak Results

	Retention Time (min)	Area	% Area	Height	Int Type
1	2.475	3882242	100.00	278240	bb

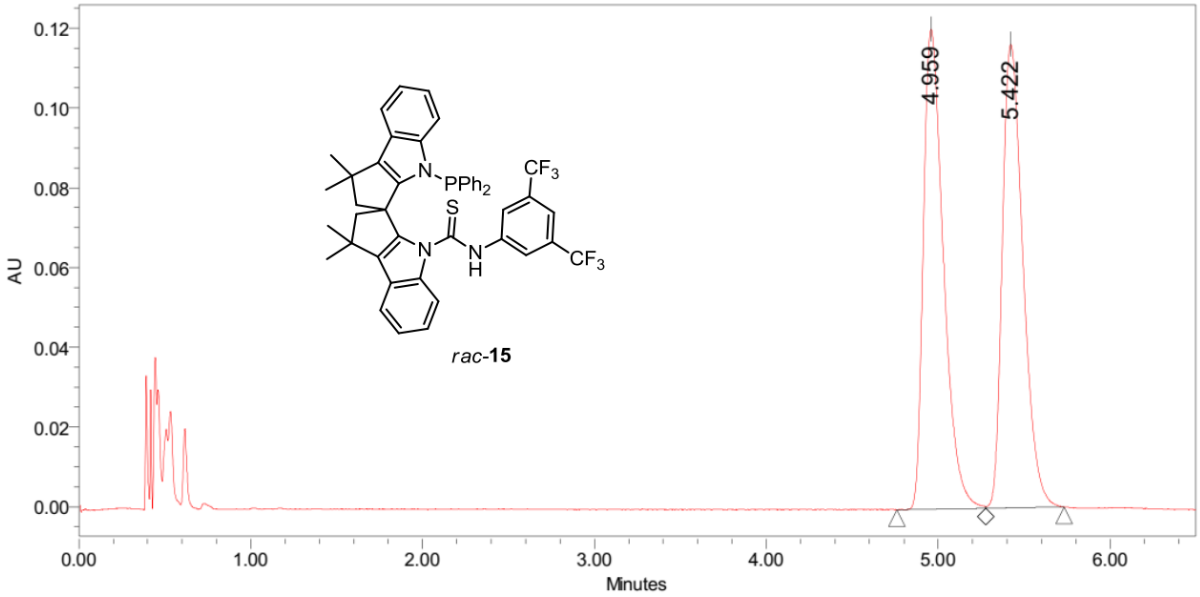

Peak Results

	Retention Time (min)	Area	% Area	Height	Int Type
1	4.959	978870	50.09	120324	bV
2	5.422	975280	49.91	116215	Vb

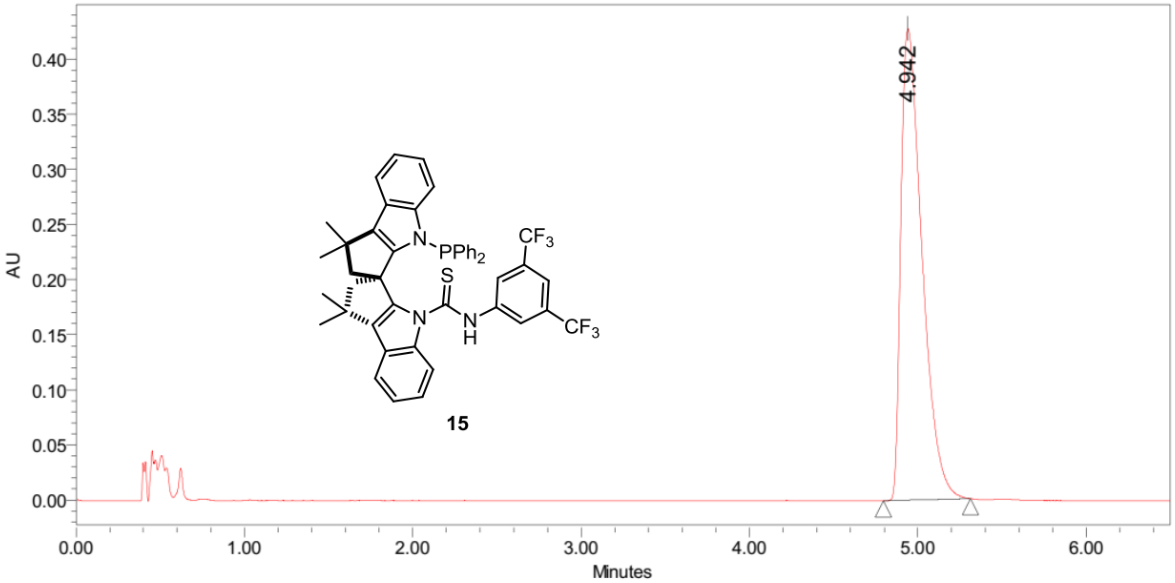

Peak Results

	Retention Time (min)	Area	% Area	Height	Int Type
1	4.942	3748241	100.00	427781	bb

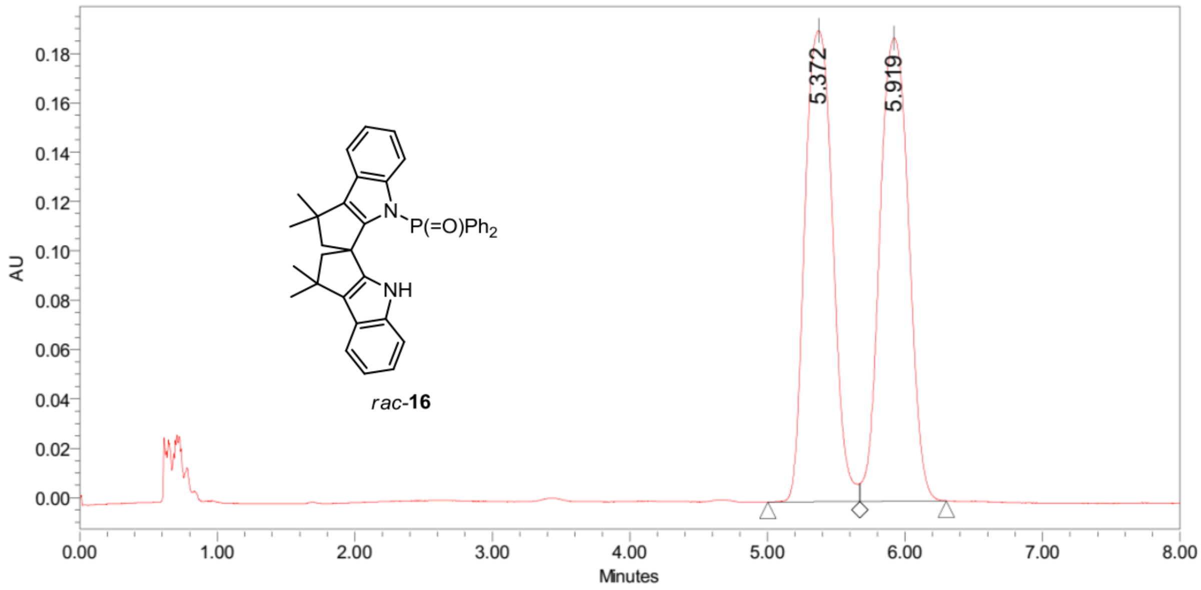

Peak Results

	Retention Time (min)	Area	% Area	Height	Int Type
1	5.372	2696295	49.50	190883	bv
2	5.919	2750974	50.50	187696	vb

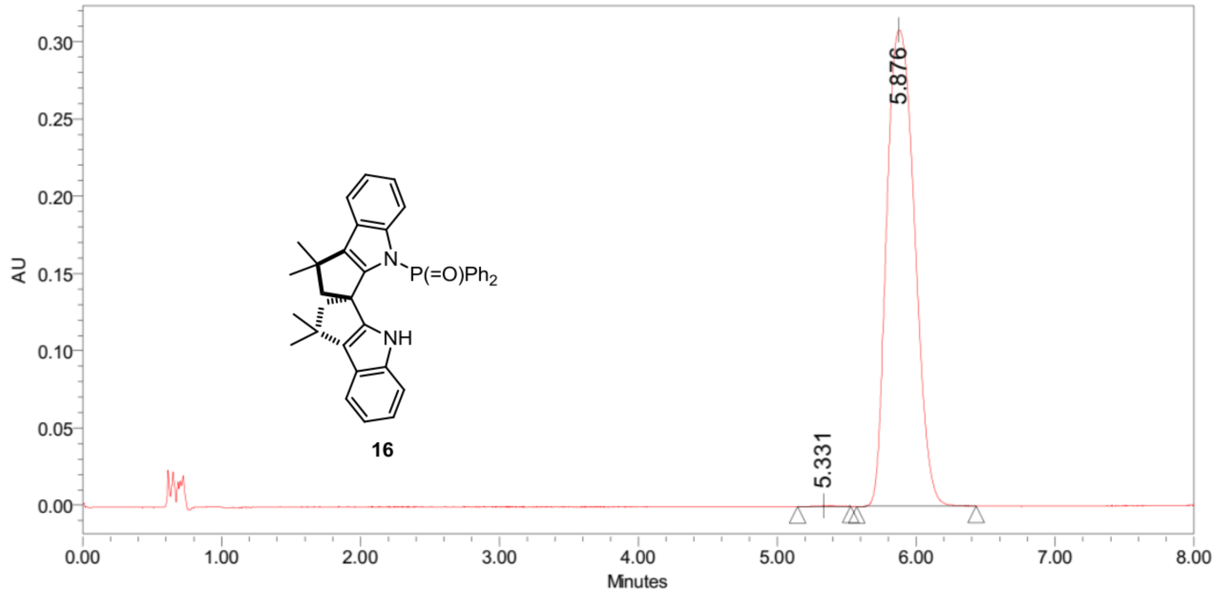

Peak Results

	Retention Time (min)	Area	% Area	Height	Int Type
1	5.331	4361	0.10	420	bb
2	5.876	4304670	99.90	308316	bb

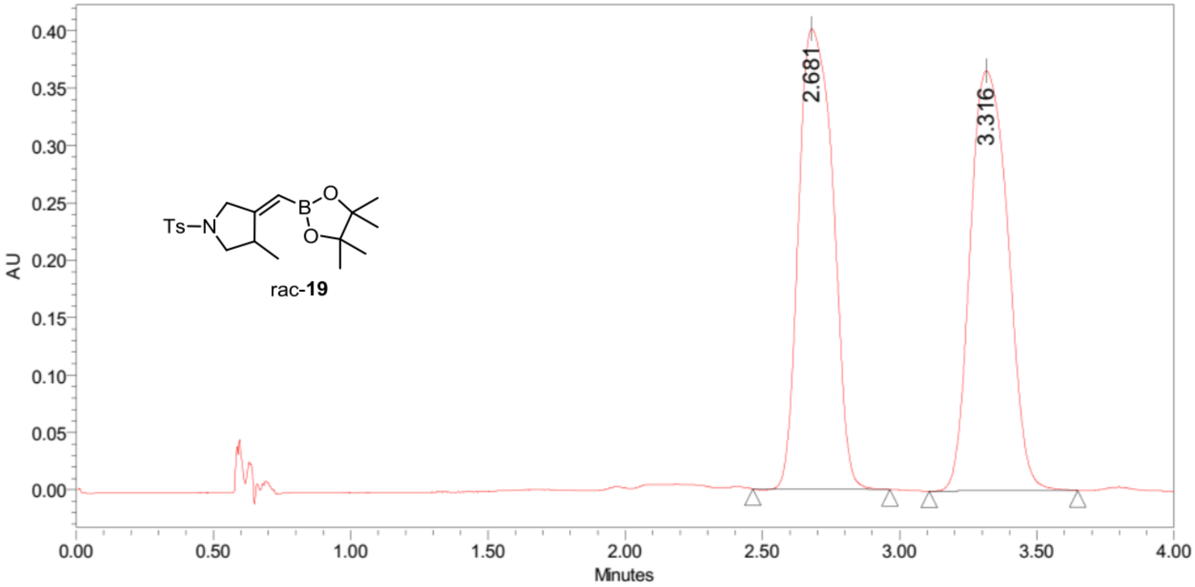

Peak Results

	Retention Time (min)	Area	% Area	Height	Int Type
1	2.681	3498867	49.94	401210	bb
2	3.316	3507760	50.06	365825	bb

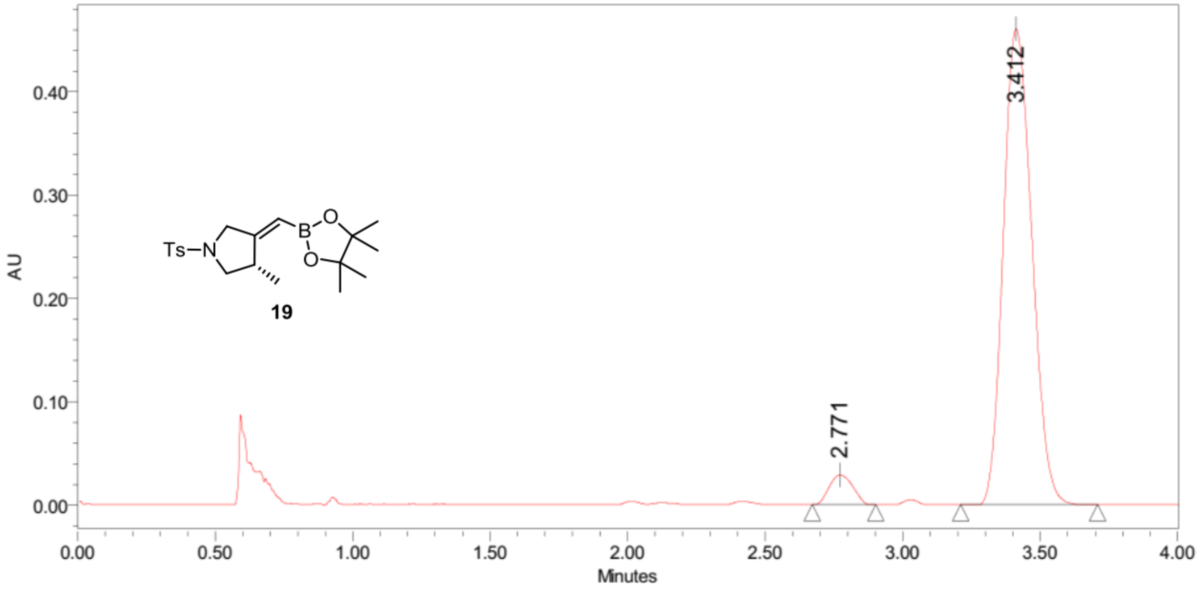

Peak Results

	Retention Time (min)	Area	% Area	Height	Int Type
1	2.771	166088	4.77	28214	bb
2	3.412	3314549	95.23	460161	bb

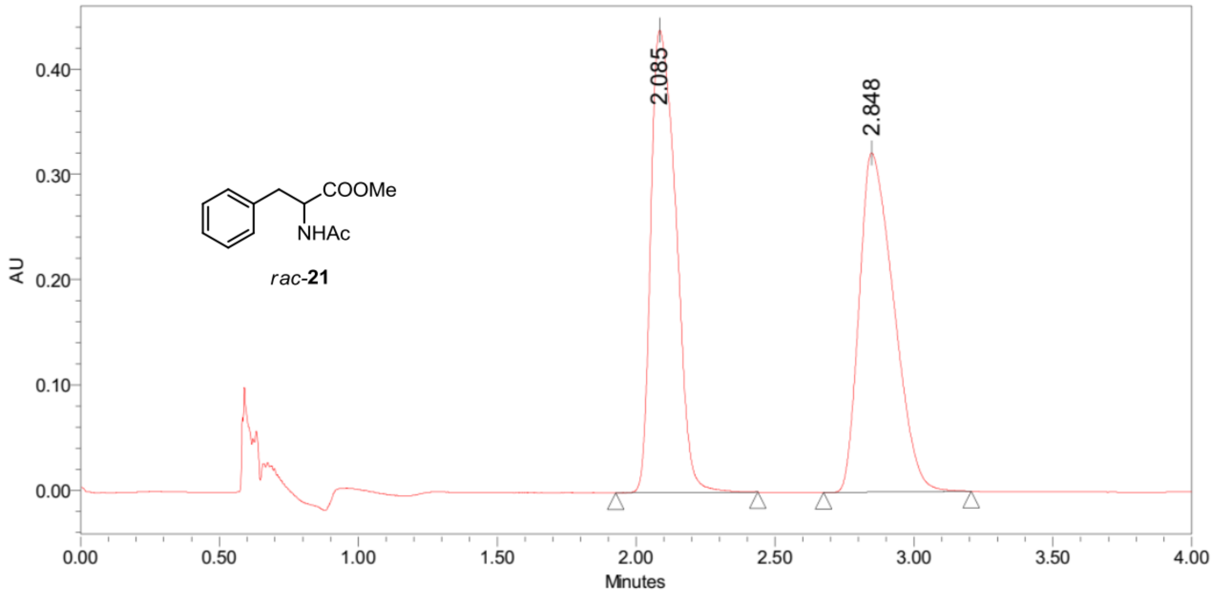

Peak Results

	Retention Time (min)	Area	% Area	Height	Int Type
1	2.085	2755142	49.82	438979	bb
2	2.848	2775590	50.18	321995	bb

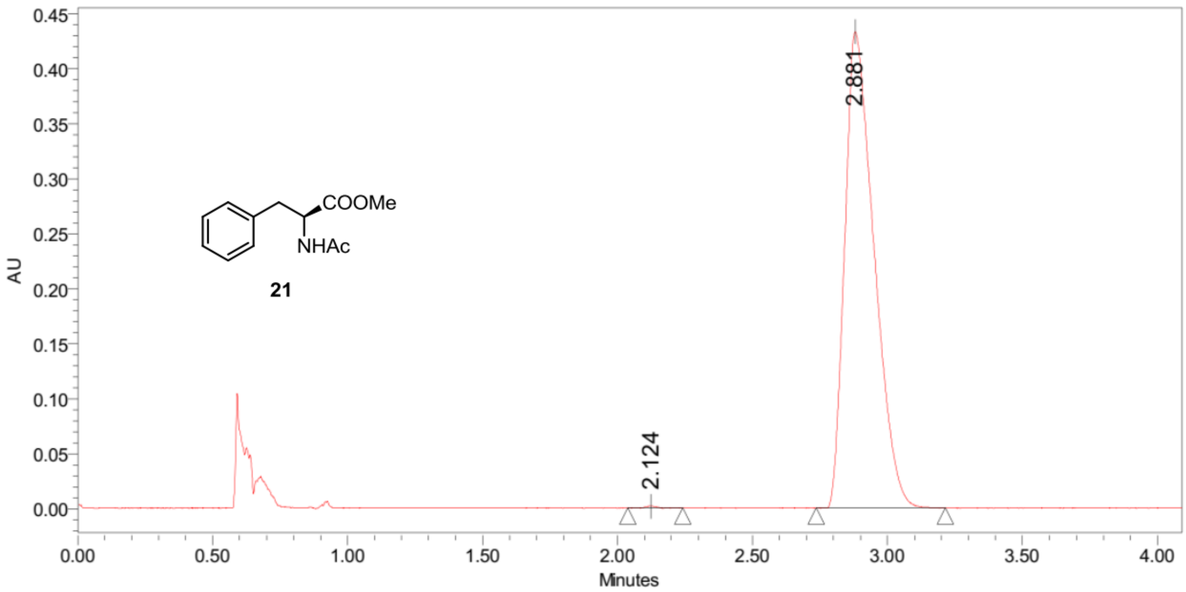

Peak Results

	Retention Time (min)	Area	% Area	Height	Int Type
1	2.124	4494	0.14	1603	bb
2	2.881	3256561	99.86	432717	bb

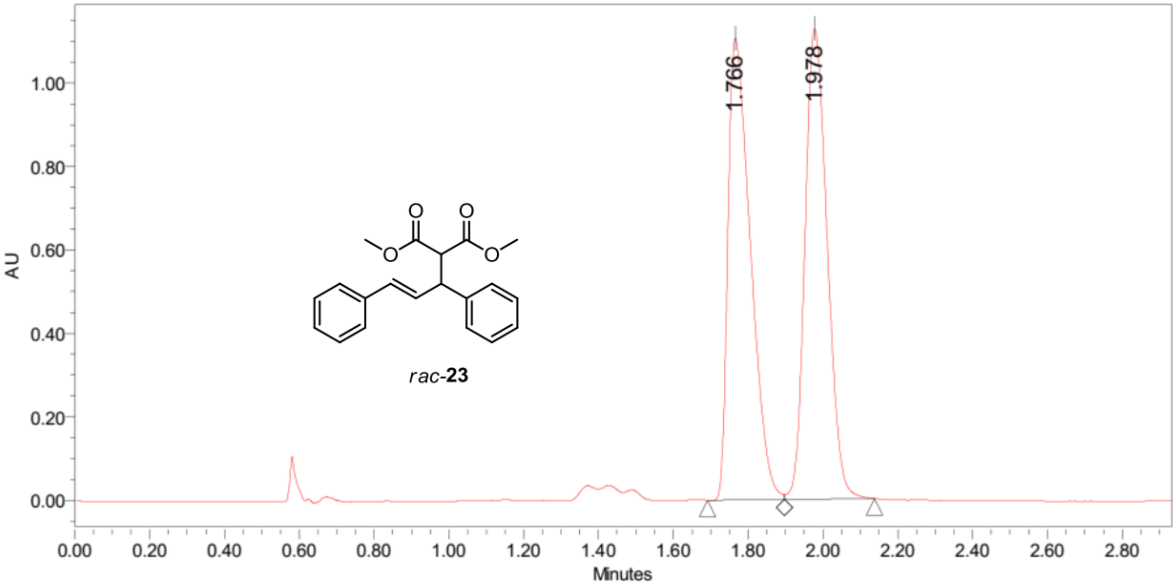

Peak Results

	Retention Time (min)	Area	% Area	Height	Int Type
1	1.766	4625258	49.89	1106941	bV
2	1.978	4644745	50.11	1127857	Vb

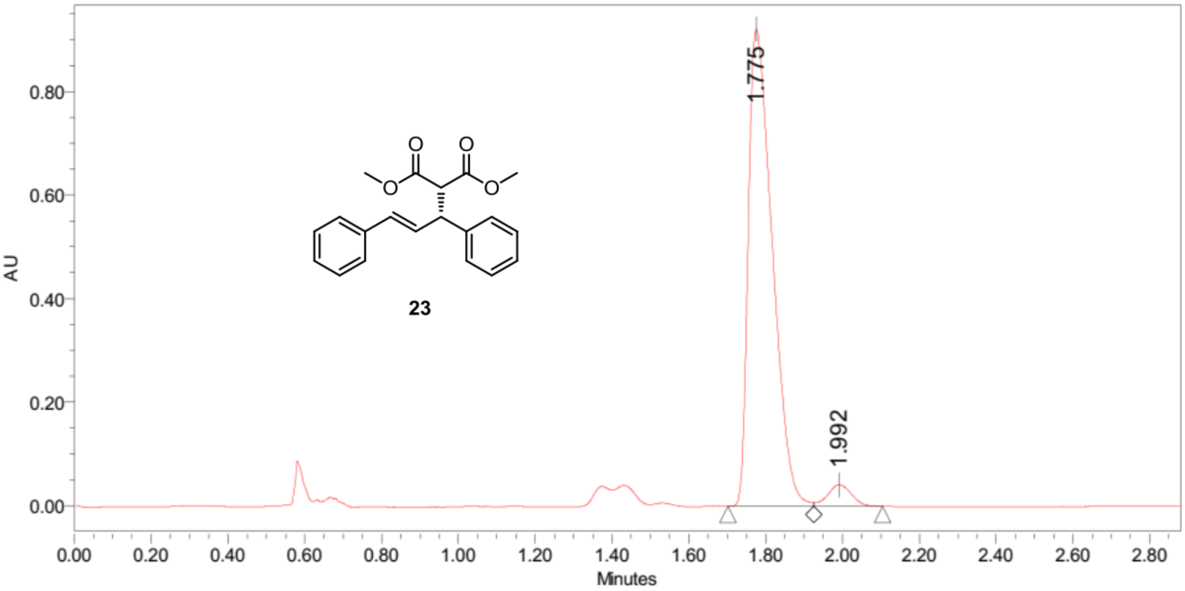

Peak Results

	Retention Time (min)	Area	% Area	Height	Int Type
1	1.775	3983098	95.65	921391	bV
2	1.992	180987	4.35	41631	Vb

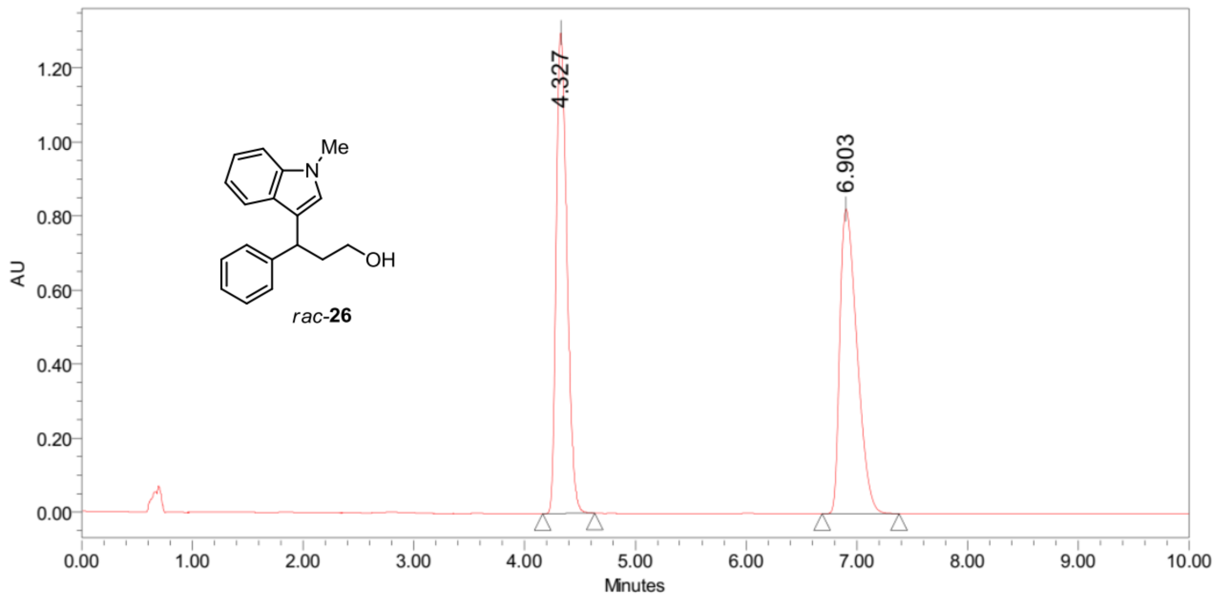

Peak Results

	Retention Time (min)	Area	% Area	Height	Int Type
1	4.327	8729050	49.63	1299064	bb
2	6.903	8858145	50.37	822879	bb

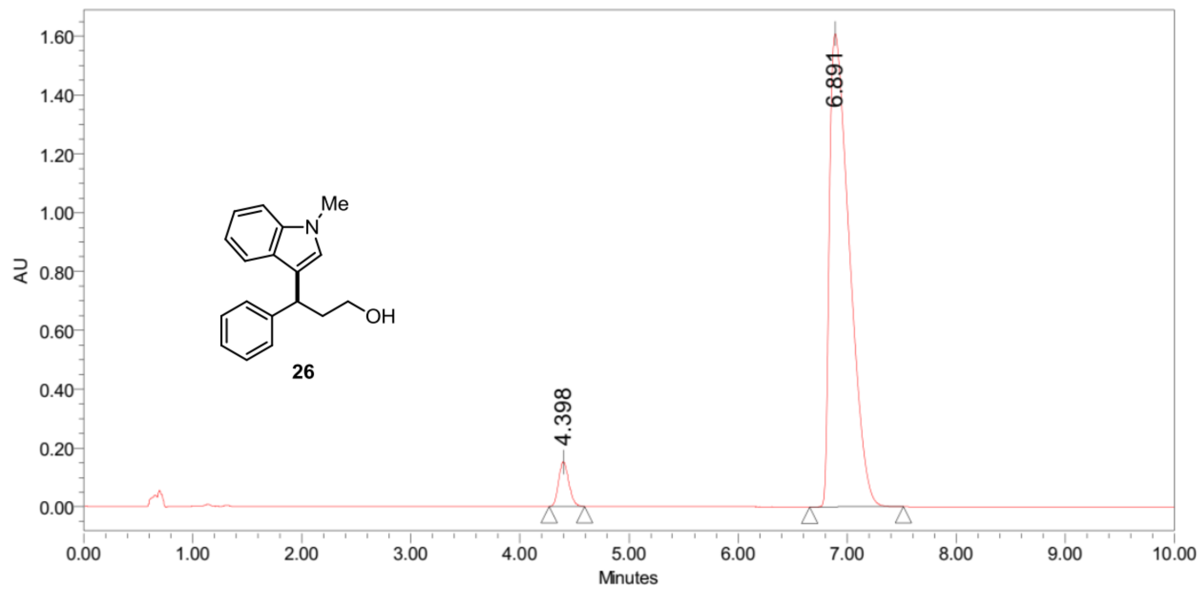

Peak Results

	Retention Time (min)	Area	% Area	Height	Int Type
1	4.398	987592	4.60	151980	bb
2	6.891	20473482	95.40	1608556	bb

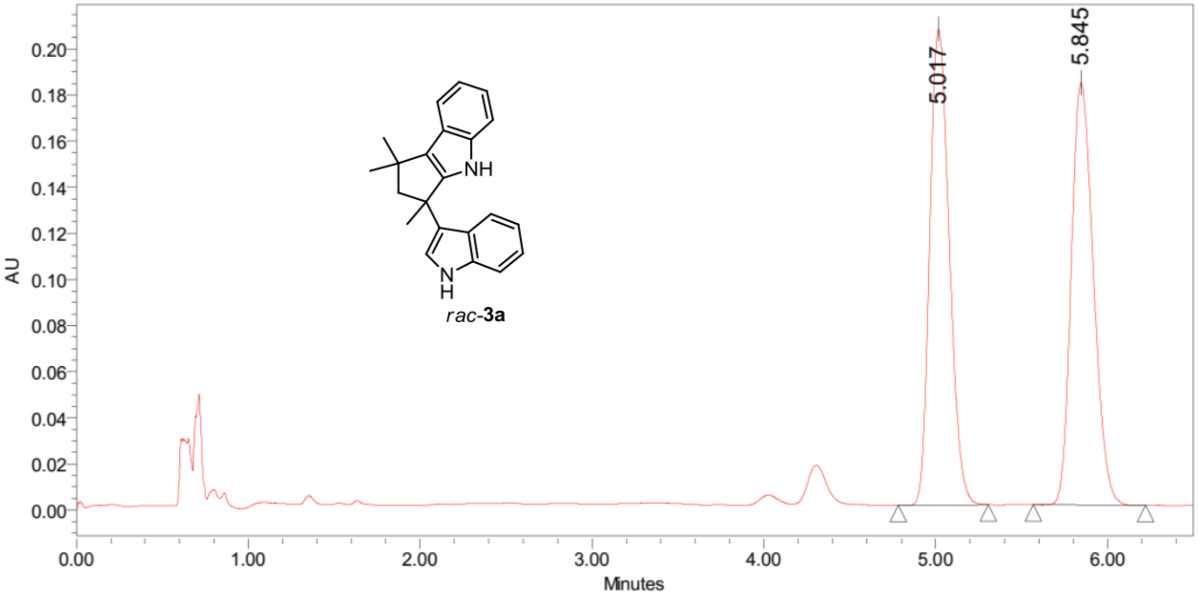

Peak Results

	Retention Time (min)	Area	% Area	Height	Int Type
1	5.017	1636184	50.04	206458	bb
2	5.845	1633504	49.96	183092	bb

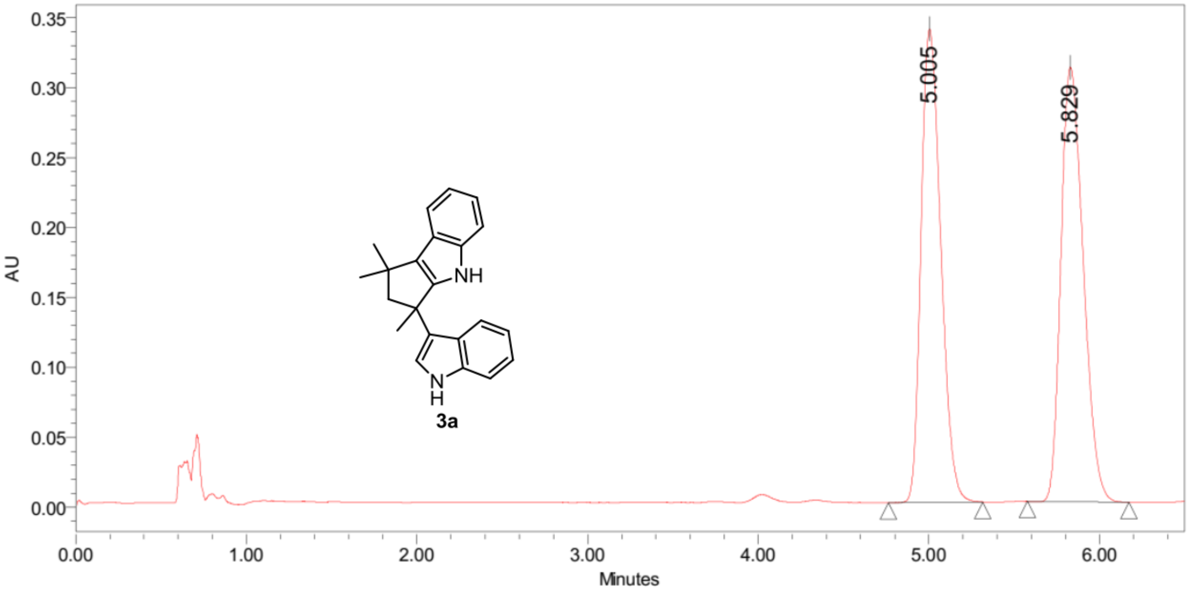

Peak Results

	Retention Time (min)	Area	% Area	Height	Int Type
1	5.005	2722209	49.12	338418	bb
2	5.829	2819379	50.88	310616	bb

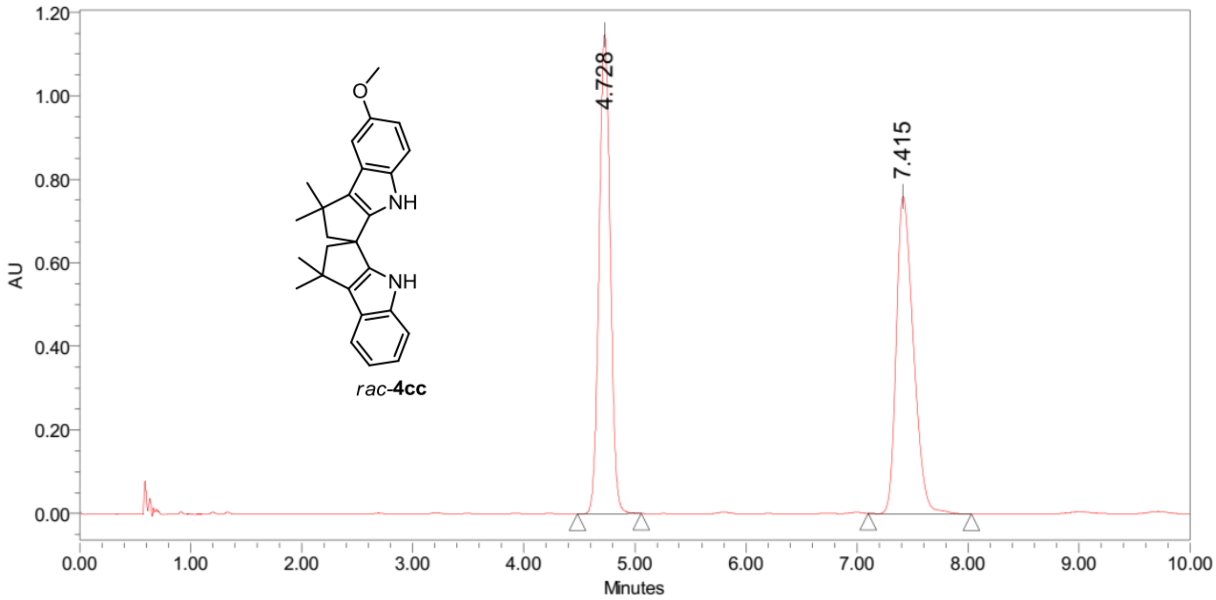

Peak Results

	Retention Time (min)	Area	% Area	Height	Int Type
1	4.728	8025406	49.69	1147730	bb
2	7.415	8124914	50.31	759766	bb

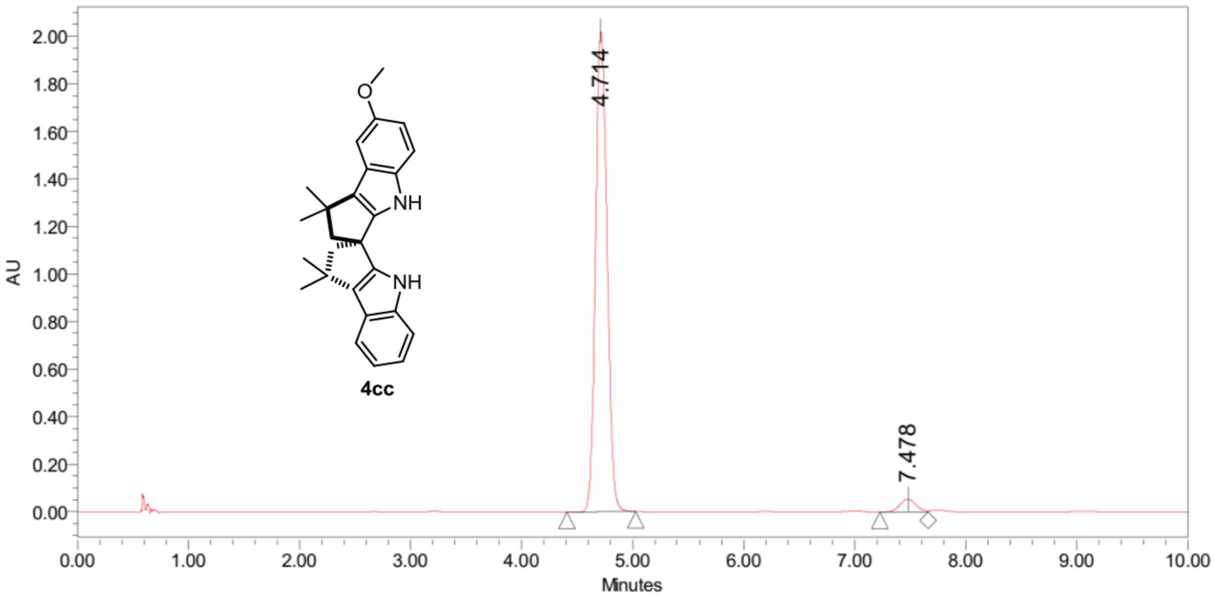

Peak Results

	Retention Time (min)	Area	% Area	Height	Int Type
1	4.714	14620464	96.41	2020600	bb
2	7.478	544393	3.59	53949	bV

13 References

1. a) Nakashima, D. & Yamamoto, H. Design of chiral N-triflyl phosphoramide as a strong chiral Brønsted acid and its application to asymmetric Diels– Alder reaction. *J. Am. Chem. Soc.* **128**, 9626–9627 (2006). b) Rueping, M., Nachtsheim, B. J., Koenigs, R. M. & Ieawsuwan, W. Synthesis and Structural Aspects of N‐Triflylphosphoramides and Their Calcium Salts—Highly Acidic and Effective Brønsted Acids. *Chem. Eur. J.* **16**, 13116–13126 (2010). c) Kong, L., Han, X. & Jiao, P. Catalytic asymmetric Diels–Alder reactions involving aryl vinyl ketones. *Chem. Commun.* **50**, 14113–14116 (2014).

2. a) Xing, C. H., Liao, Y. X., Ng, J. & Hu, Q. S. Optically active 1, 1′-spirobiindane-7, 7′-diol (SPINOL)-based phosphoric acids as highly enantioselective catalysts for asymmetric organocatalysis. *J. Org. Chem.* **76**, 4125–4131 (2011). b) Müller, S., Webber, M.J. & List, B. The catalytic asymmetric Fischer indolization. *J. Am. Chem. Soc.* **133**, 18534–18537 (2011). c) Xing, C. H., Liao, Y. X., Zhang, Y., Sabarova, D., Bassous, M. & Hu, Q.S. Asymmetric Allylboration of Aldehydes with Pinacol Allylboronates Catalyzed by 1, 1′‐Spirobiindane‐7, 7′‐diol (SPINOL) Based Phosphoric Acids. *Eur. J. Org. Chem.* **2012**, 1115–1118 (2012).

3. a) Nakashima, D. & Yamamoto, H. Design of chiral N-triflyl phosphoramide as a strong chiral Brønsted acid and its application to asymmetric Diels– Alder reaction. *J. Am. Chem. Soc.* **128**, 9626–9627 (2006). b) Rueping, M., Nachtsheim, B.J., Koenigs, R.M. & Ieawsuwan, W. Synthesis and Structural Aspects of N‐Triflylphosphoramides and Their Calcium Salts—Highly Acidic and Effective Brønsted Acids. *Chem. Eur. J.* **16**, 13116–13126 (2010).

4. a) Kaib, P. S., Schreyer, L., Lee, S., Properzi, R. & List, B. Extremely active organocatalysts enable a highly enantioselective addition of allyltrimethylsilane to aldehydes. *Angew. Chem. Int. Ed.* **55**, 13200–13203 (2016). b) Xie, Y., Cheng, G. J., Lee, S., Kaib, P. S., Thiel, W. & List, B. Catalytic asymmetric vinylogous Prins cyclization: a highly diastereo-and enantioselective entry to tetrahydrofurans. *J. Am. Chem. Soc.* **138**, 14538–14541 (2016). c) Lee, S., Kaib, P. S. & List, B. Asymmetric catalysis via cyclic, aliphatic oxocarbenium ions. *J. Am. Chem. Soc.* **139**, 2156–2159 (2017). d) Ghosh, S., Das, S., De, C. K., Yepes, D., Neese, F., Bistoni, G., Leutzsch, M. & List, B. Strong and confined acids control five stereogenic centers in catalytic asymmetric Diels–Alder reactions of cyclohexadienones with cyclopentadiene. *Angew. Chem. Int. Ed.* **59**, 12347–12351 (2020). e) Zhou, H., Bae, H. Y., Leutzsch, M., Kennemur, J. L., Bécart, D. & List, B. The

silicon–hydrogen exchange reaction: a catalytic σ-bond metathesis approach to the enantioselective synthesis of enol silanes. *J. Am. Chem. Soc.* **142**, 13695–13700 (2020). f) Kim, H., Gerosa, G., Aronow, J., Kasaplar, P., Ouyang, J., Lingnau, J. B., Guerry, P., Farès, C. & List, B. A multi-substrate screening approach for the identification of a broadly applicable Diels–Alder catalyst. *Nat. Commun.* **10**, 770 (2019). g) Properzi, R., Kaib, P. S., Leutzsch, M., Pupo, G., Mitra, R., De, C.K., Song, L., Schreiner, P. R. & List, B. Catalytic enantiocontrol over a non-classical carbocation. *Nature Chem.* **12**, 1174–1179 (2020). h) Ouyang, J., Bae, H., Jordi, S., Dao, Q. M., Dossenbach, S., Dehn, S., Lingnau, J.B., Kanta De, C., Kraft, P. & List, B. The smelling principle of vetiver oil, unveiled by chemical synthesis. *Angew. Chem. Int. Ed.* **60**, 5666–5672 (2021). i) Ouyang, J., Maji, R., Leutzsch, M., Mitschke, B. & List, B. Design of an organocatalytic asymmetric (4+ 3) cycloaddition of 2-indolylalcohols with dienolsilanes. *J. Am. Chem. Soc.* **144**, 8460–8466 (2022). j) Nistanaki, S. K., Williams, C. G., Wigman, B., Wong, J. J., Haas, B. C., Popov, S., Werth, J., Sigman, M. S., Houk, K. N. & Nelson, H. M. Catalytic asymmetric C–H insertion reactions of vinyl carbocations. *Science* **378**, 1085–1091 (2022). k) Grimm, J. A., Zhou, H., Properzi, R., Leutzsch, M., Bistoni, G., Nienhaus, J. & List, B. Catalytic asymmetric synthesis of cannabinoids and menthol from neral. *Nature* **615**, 634–639 (2023).

5. a) Liu, L., Kaib, P. S., Tap, A. & List, B. A general catalytic asymmetric Prins cyclization. *J. Am. Chem. Soc.* **138**, 10822–10825 (2016). b) Schwengers, S.A., De, C.K., Grossmann, O., Grimm, J. A., Sadlowski, N. R., Gerosa, G. G. & List, B. Unified approach to imidodiphosphate-type Brønsted acids with tunable confinement and acidity. *J. Am. Chem. Soc.* **143**, 14835–14844 (2021). c) Díaz-Oviedo, C.D., Maji, R. & List, B. The catalytic asymmetric intermolecular Prins reaction. *J. Am. Chem. Soc.* **143**, 20598–20604 (2021).

6. Kim, J. H., Coric, I., Palumbo, C. & List, B. Resolution of diols via catalytic asymmetric acetalization. *J. Am. Chem. Soc.* **137**, 1778–1781 (2015).

7. Ammar, H. B., Hassine, B. B., Fischmeister, C., Dixneuf, P. H. & Bruneau, C. Imidazolium - Oxazoline Salts in Ruthenium - Catalyzed Allylic Substitution and Cross Metathesis of Formed Branched Isomers. *Eur. J. Inorg. Chem.* **30**, 4752–4756 (2010).

8. Maji, K., Thorve, P.R., Rai, P. & Maji, B. Enantioselective C–H bond functionalization of aromatic ketones with 1, 6-enynes via photoredox/cobalt dual catalysis. *Chem. Commun.* **58**, 9516–9519 (2022).

9. Zheng, L., Zheng, D., Wang, Y., Yu, C., Zhang, K. & Jiang, H. Chiral bisphosphine ligands based on quinoline oligoamide foldamers: application in asymmetric hydrogenation. *Org. Biomol. Chem.* **17**, 9573–9577 (2019).

10. Singh, B., Bankar, S.K. & Ramasastry, S.S.V. Pd-Catalyzed Nazarov-Type Cyclization: Application in the Total Synthesis of β-Diasarone and Other Complex Cyclopentanoids. *Org. Lett.* **24**, 1043–1048 (2022).

11. Frisch, M. J. et al. Gaussian 16, Revision C.01, Gaussian, Inc.: Wallingford, CT 2016.

12. Chai, J. D. & Head-Gordon, M. Long-range corrected hybrid density functionals with damped atom–atom dispersion corrections. *Phys. Chem. Chem. Phys.* **10**, 6615-6620 (2008).

13. Krishnan, R.B.J.S., Binkley, J.S., Seeger, R. & Pople, J.A. Self-consistent molecular orbital methods. XX. A basis set for correlated wave functions.*J. Chem. Phys.* **72**, 650-654 (1980).

14 . Grimme, S. Supramolecular binding thermodynamics by dispersion-corrected density functional theory. *Chem. Eur. J.* **18**, 9955–9964 (2012).

15. Luchini, G., Alegre-Requena, J. V., Funes-Ardoiz, I. and Paton, R.S. GoodVibes: automated thermochemistry for heterogeneous computational chemistry data. *F1000Res.* **9**, 291 (2020).

16. MacroModel, version 11.7, Schrödinger, LLC, New York, NY, 2017.

17. Harder, E., Damm, W., Maple, J., Wu, C., Reboul, M., Xiang, J. Y., Wang, L., Lupyan, D., Dahlgren, M. K., Knight, J. L. & Kaus, J. W. OPLS3: a force field providing broad coverage of drug-like small molecules and proteins. *J. Chem. Theory Comput.* **12**, 281–296 (2016).

18. Shelke, G. M., Rao, V. K., Tiwari, R. K., Chhikara, B. S., Parang, K. & Kumar, A. Bismuth triflate-catalyzed condensation of indoles with acetone. *RSC Adv.* **3**, 22346–22352 (2013).

19. Bhattacharjee, P., Sarma, B. & Bora, U. Molecular-iodine catalyzed selective construction of cyclopenta [b] indoles from indoles and acetone: a green gateway to indole-fused cycles. *Org. Biomol. Chem.* **21**, 9275–9285 (2023).

20. Hou, F., Ning, Y., Song, L., Tan, Z., Yang, J., Liu, Z. & Chen, F. E. Rhodium-Catalyzed Asymmetric Hydroboration/Cyclization of 1, 6-Enynes Enabled by Spirosiladiphosphine Ligands: Constructing Chiral Five-Membered Rings with a Boron Handle. *Org. Lett.* **25**, 7810–7815 (2023).

21. Yang, L., Xu, W. Q., Liu, T., Wu, Y., Wang, B. & Wang, P. Concise synthesis and applications of enantiopure spirobiphenoxasilin-diol and its related chiral ligands. *Chem. Commun.* **57**, 13365-13368 (2021).

22. Xie, J. H., Duan, H. F., Fan, B. M., Cheng, X., Wang, L. X. & Zhou, Q. L. Application of SDP Ligands for Pd‐Catalyzed Allylic Alkylation. *Adv. Synth. Catal.* **346**, 625-632 (2004).

23. Holland, M. C., Metternich, J. B., Mück-Lichtenfeld, C. & Gilmour, R. Cation–π interactions in iminium ion activation: correlating quadrupole moment & enantioselectivity. *Chem. Commun.* **51**, 5322–5325 (2015).

24. Software for the Integration of CCD Detector System Bruker Analytical X-Ray Systems, Bruker AXS, Madison, WI (after 2013).

25. Sheldrick, G. M. Crystal structure refinement with SHELXL. *Acta Crystallogr. Sect. Found. Adv.* **71**, 3–8 (2015).

26. Dolomanov, O. V., Bourhis, L. J., Gildea, R. J., Howard, J. A. & Puschmann, H. OLEX2: a complete structure solution, refinement and analysis program. *J. Appl. Crystallogr.* **42**, 339–341 (2009).
